# Psychological factors associated with Long COVID: a systematic review and meta-analysis

**DOI:** 10.1016/j.eclinm.2024.102756

**Published:** 2024-07-26

**Authors:** Petra Engelmann, Max Reinke, Clara Stein, Stefan Salzmann, Bernd Löwe, Anne Toussaint, Meike Shedden-Mora

**Affiliations:** a Department of Psychosomatic Medicine and Psychotherapy, University Medical Centre Hamburg-Eppendorf, Hamburg, Germany; b Division of Clinical Psychology and Psychotherapy, Philipps-University Marburg, Marburg, Germany; c Medical Psychology, Health and Medical University Erfurt, Erfurt, Germany; d Department of Psychology, Medical School Hamburg, Hamburg, Germany

**Keywords:** Long COVID, Post COVID-19 condition, Persistent somatic symptoms, Biopsychosocial model, Psychological factors

## Abstract

**Background:**

Despite the immense impact of Long COVID on public health and those affected, its aetiology remains poorly understood. Findings suggest that psychological factors such as depression contribute to symptom persistence alongside pathophysiological mechanisms, but knowledge of their relative importance is limited. This study aimed to synthesise the current evidence on psychological factors potentially associated with Long COVID and condition-relevant outcomes like quality of life.

**Methods:**

In this systematic review and meta-analysis, MEDLINE, PsycINFO, and the Cochrane Database of Systematic Reviews were searched for peer-reviewed studies published in English from 2019 to January 2, 2024. Studies providing cross-sectional or longitudinal data on the association between at least one psychological variable and the presence of Long COVID (primary outcome) or condition-relevant secondary outcomes (symptom severity, impairment, quality of life, and healthcare utilisation) were included. Psychological constructs with at least five comparisons were pooled as odds ratio (OR) for categorical data and standardised mean difference (SMD) for continuous data in random-effects meta-analyses of cross-sectional studies with control groups. This review is registered with PROSPERO, CRD42023408320.

**Findings:**

113 studies (n = 312,831 patients with Long COVID) provided data on at least one psychological variable, 63 in cross-sectional group comparisons, 53 in cross-sectional associations, and 18 longitudinal. Most reported findings related to depression and anxiety, and — less frequently — to physical activity, posttraumatic stress disorder, stress, and history of mental illness. Depression (OR 2.35; 95% CI, 1.49–3.70) and anxiety (OR 2.53; 95% CI, 1.76–3.61) were significantly associated with Long COVID and higher in affected patients than controls (depression: SMD 0.88; 95% CI, 0.66–1.11; anxiety: SMD 0.74; 95% CI, 0.50–0.99), while results for physical activity and stress were non-significant. In most prospective studies, the investigated psychological constructs significantly predicted Long COVID.

**Interpretation:**

Evidence suggests depression and anxiety to be co-occurring phenomena and predictive factors of Long COVID. Future studies should prospectively investigate psychological constructs such as emotion regulation or dysfunctional symptom expectations, which are well-known risk factors and therapeutic targets of persistent somatic symptoms in other medical conditions, but are so far understudied in Long COVID.

**Funding:**

None.

**Research in context**

**Evidence before this study**

Besides pathophysiological mechanisms, a growing number of studies indicate the potential role of psychological factors in the development and maintenance of Long COVID. Previous reviews of risk factors for Long COVID confirmed the importance of depression and anxiety; however, a broad spectrum of further psychological variables possibly involved in the persistence of somatic symptoms, such as emotion regulation or dysfunctional symptom expectations, has been relatively overlooked. To improve the understanding of Long COVID and inform research into much-needed treatments, aggregating the available evidence on psychological factors associated with Long COVID is of high relevance. We searched MEDLINE, PsycINFO, and the Cochrane Database of Systematic Reviews for peer-reviewed studies published in English from 2019 to January 2, 2024. Studies were required to report a) cross-sectional comparisons on psychological factors between patients with Long COVID and controls, b) cross–sectional associations with Long COVID or condition-relevant outcomes (symptom severity, impairment, quality of life, and healthcare utilisation), or c) prospective relations between psychological factors and Long COVID. 113 eligible studies of mostly fair quality included 312,831 patients with Long COVID and provided data for 58 psychological constructs in total.

**Added value of this study**

Of all psychological variables investigated, this systematic review could only confirm higher levels of depression and anxiety in individuals with Long COVID compared to controls in meta-analyses. Both factors were also predictive of Long COVID in longitudinal studies. The scarce evidence base for all other psychological variables hinders reliable conclusions.

**Implications of all the available evidence**

The results of this review, together with previous studies, support that depression and anxiety should be considered in multidisciplinary Long COVID treatments. Future studies should examine psychological constructs such as emotion regulation or expectations, which are well-known risk factors and therapeutic targets of persistent somatic symptoms in other medical conditions, but are so far understudied in Long COVID. Large methodological heterogeneity of the identified studies points to the need of guidelines for research on Long COVID.

## Introduction

Five years after the outbreak of the COVID-19 pandemic, the aetiology of Long COVID^1^ is far from being clearly understood. Investigations into the pathophysiology of persistent somatic symptoms after SARS-CoV-2 infection have remained inconclusive so far, with no diagnostic markers known to fully explain them^2^ and several reports of objective clinical examinations discrepant with subjective symptom burden.^3^, ^4^, ^5^ While a growing body of research suggests that psychological factors like depression or anxiety contribute to the development and maintenance of Long COVID in addition to pathophysiological changes,^6^, ^7^, ^8^ the discussion about the involvement of psychological processes in its aetiology also raises critics who fear a “psychologisation” of the condition.^6^^,^^9^

Persistent somatic symptoms remain after many infectious diseases^10^ and are generally common in the general population.^11^ Research into persistent somatic symptoms and chronic medical diseases indicates accompanying psychological features such as health anxiety or negative expectations to be more crucial for impairment in quality of life or increase in healthcare utilisation of affected patients than the severity of the somatic symptoms per se.^12^ Accordingly, evidence-based etiological models clearly argue for the contribution of both biomedical and psychological mechanisms to the development and persistent course of somatic symptoms.^13^, ^14^, ^15^ Besides depression and anxiety,^16^^,^^17^ aggravating factors include cognitive-perceptual mechanisms such as selective attention, somatosensory amplification,^18^ and catastrophising.^19^^,^^20^ Additional assumed maintaining factors are unhelpful symptom-related behaviours like physical inactivity and dysfunctional healthcare use.^21^^,^^22^ Emotion regulation deficits, adverse childhood experiences, negative affectivity, or life stressors^20^^,^^23^, ^24^, ^25^ can predispose the development of persistent somatic symptoms. The same complex biopsychosocial interactions including a broad spectrum of psychological mechanisms can also be assumed for processes of symptom development and persistence in Long COVID.

While not accounting for non-affected control groups, van der Feltz-Cornelis et al.^26^ recently meta-analysed the all-time prevalence of any mental health condition in patients with Long COVID. They found a prevalence of 20.4%, with most studies on depression and anxiety, and the odds of mental health conditions significantly increasing over time after acute infection. Previous systematic reviews and meta-analyses of risk factors for Long COVID have largely neglected the role of psychological risk factors other than depression and anxiety.^7^^,^^27^

Regarding further potentially relevant psychological concepts, Hüsing et al.^28^ developed the PSY-PSS framework for systematic reviews to support the aggregation of empirical evidence on psychological (risk) factors in persistent somatic symptoms and related conditions. It provides the first comprehensive list of psychological variables to be considered in this field of research, which is constantly being expanded and has recently been applied in a systematic review of psychological risk factors for somatic symptom disorder.^29^ Considering Long COVID as dominated by persistent somatic symptoms, it is likely that the psychological constructs compiled in the PSY-PSS framework are also relevant with regard to this condition.

This study aimed to systematically review literature providing original data on psychological constructs associated with Long COVID and Long COVID-relevant outcomes by applying the PSY-PSS framework^28^ and, where possible, pool data using meta-analyses. For an individual psychological variable to be of relevance for Long COVID, it should either 1) be significantly higher in patients with Long COVID compared to controls, or 2) show a significant association with Long COVID or condition-relevant outcomes (symptom severity, impairment, quality of life, and healthcare utilisation), or 3) prove as a significant predictor of the development or maintenance of Long COVID. Consequently, three methodological approaches were followed and translated into the following three research questions (RQs):

1) In which psychological factors do patients with Long COVID report significantly higher values compared to control groups (cross-sectional group comparisons; RQ1)?
2) Which psychological factors are significantly associated with the presence of Long COVID or condition-relevant outcomes such as symptom severity, impairment, quality of life, and healthcare utilisation (cross-sectional associations; RQ2)?
3) Which psychological factors significantly predict the development or maintenance of Long COVID (longitudinal data; RQ3)?

## Methods

This systematic review and meta-analysis was preregistered on PROSPERO (CRD42023408320) and was conducted and reported in accordance with the PRISMA guidelines as well as the MOOSE reporting guidelines for meta-analyses of observational studies.^30^ It is the second review that emerged from a proposed framework of systematic reviews designed to facilitate research in the area of persistent somatic symptoms (PSY-PSS^28^) and follows the methodology outlined in the PROSPERO registration (CRD42022302014). Ethical approval was not sought or required, as the study involved no individual patient data.

### Eligibility criteria

Articles were eligible if written in English and published in a peer-reviewed journal since 2019, i.e., since the COVID-19 outbreak. Studies were required to report original, quantitative data on at least one of the pre-defined psychological variables from the comprehensive list of the PSY-PSS framework by any tool of measure in patients of any age group who self-report to suffer from or have received a clinical diagnosis of Long COVID according to the NICE guideline (i.e., somatic symptoms that develop during or after SARS-CoV-2 infection, continue for at least 4 weeks, and are not explained by an alternative diagnosis).^1^ Studies that referred to other conditions or used an unclear definition of Long COVID were excluded.

Studies needed to provide a) cross-sectional comparisons between patients with Long COVID and controls, b) cross-sectional associations, or c) longitudinal data on the relation between psychological variables and the presence of Long COVID (primary outcome) or condition-relevant secondary outcomes (i.e., symptom severity, impairment defined as impaired functioning in activities of daily living,^31^ quality of life, or healthcare utilisation). Preprints, case reports, purely qualitative studies, reviews and meta-analyses with no new data, study protocols, editorials, comments, letters, conference abstracts, and grey literature such as dissertations were excluded. Studies using results from previous publications (secondary analyses) were not included to prevent bias.

### Study selection

To ensure all relevant publications on Long COVID were found, an extensive list consisting of the 21 most commonly used terms for Long COVID in pertinent publications and guidelines^1^^,^^32^ in a variation of spellings was compiled. For psychological factors, the comprehensive list of the PSY-PSS framework^28^ was applied, which was generated to be used as search terms for literature reviews on persistent somatic symptoms. This list has a hierarchical structure, with seven overarching categories of psychological mechanisms (affective, cognitive, behavioural, psychophysiological, personality & interpersonal factors, prior experiences, and psychopathology) containing 120 psychological variables overall (e.g., emotion regulation, expectations, or illness behaviour; list and search terms can be found in the open science framework: https://osf.io/anbm6). All 120 terms from the list of potentially relevant psychological factors were crossed with all 21 Long COVID terms. The literature search was first run on 06/25/2023, and updated on 01/02/2024 using the bibliographic databases MEDLINE (via PubMed), PsycINFO (via OvidSP), and the Cochrane Database of Systematic Reviews (via Cochrane Library). The search syntax string including the precise search terms is available in eAppendix 1 in the Supplement. Due to the large number of hits, a manual search in the reference lists of obtained articles was not carried out.

Endnote^33^ was used to combine identified studies from all databases. Three authors (CS, MR, PE) removed duplicates and screened study titles and abstracts using the software Rayyan.^34^ Conflicts were resolved following discussion between the three authors. Studies identified for full-text review were independently reviewed twice against eligibility criteria by initially blinded authors (PE, MR, CS, SS, AT, MSM). Disagreements during full-text review were cleared through consultation of other authors (PE, MR, CS, SS, AT, MSM).

### Data extraction

Full-text data extraction was independently completed and summarised in one descriptive and three results tables (one for each RQ) by four authors (PE, MR, CS, AT). Descriptive data comprising study design and objectives, sample characteristics, diagnostic criteria used for Long COVID, and a short summary of key results were extracted for all studies. If control groups were described as individuals without prior SARS-CoV-2 infection and not explicitly labelled as diagnosed with another medical condition, they were categorised as “healthy controls without a history of COVID-19”. For cross-sectional associations and longitudinal evidence (RQ2 and RQ3), condition-relevant outcomes and their assessment along with effect sizes (if reported) were compiled. Besides the operationalisation of the respective construct, the following data were retrieved regarding psychological variables: raw measures of central tendency, frequencies, significance statistics, and effect sizes for each group in cross-sectional comparisons (RQ1), correlations and proportions for cross-sectional associations (RQ2), and predictive testing for longitudinal studies (RQ3). When reported data were inconsistent or incomplete, corresponding authors were contacted for clarification during the data extraction process.

### Statistics

The study quality assessment tools of the National Institutes of Health (https://www.nhlbi.nih.gov/health-topics/study-quality-assessment-tools) were employed to assess study quality and bias. Two authors (PE, AT) independently evaluated each study. Disparities were discussed between the two authors until reaching agreement.

To provide a concise overview of the available evidence for each psychological construct investigated so far in any of the included study designs, results were further summarised in a synthesis table. Meta-analyses were conducted if valid data was available for at least five studies per psychological variable, per type of data (psychological features in categorical vs. continuous format), and per outcome variable (for RQ2 and RQ3). The minimum number of studies for meta-analysis was increased from three to five compared to the predefined number stated in the PROSPERO registration (CRD42023408320) in favour of the robustness of findings given the heterogeneous assessment of psychological variables.

Meta-analyses were performed separately for psychological variables measured categorically (e.g., diagnosis of depression) and continuously (e.g., depression severity). If studies did not report the same central tendencies, corresponding authors were asked to provide mean values to integrate all continuous data. For studies that reported several follow-ups on persistent symptoms after SARS-CoV-2 infection, the first follow-up was used to be able to run analyses with the highest pooled sample size possible. Control groups of studies that examined both healthy controls without a history of SARS-CoV-2 infection and patients after SARS-CoV-2 infection without Long COVID were merged into one control group since no relevant differences between these two groups were assumed (i.e., healthy controls might have had a hidden infection).^35^ Studies with other control groups were excluded from meta-analyses due to an insufficient number of studies.

Due to the presumed heterogeneity between studies, random-effects models with raw data were employed. Effect sizes were estimated as odds ratio (OR) for categorical data and standardised mean difference (SMD) for continuous data. Heterogeneity between studies was quantified using *I*^2^. Risk for publication bias was assessed graphically through funnel plots and statistically with Egger's regression test. In case of significance, the trim-and-fill method was applied. Sensitivity analyses were conducted by first excluding studies with the greatest weight, then those with the smallest sample size (n < 20 per group), and again by excluding studies rated as poor in the quality assessment. All analyses were performed in RStudio version 4.2.3.^36^

### Role of the funding source

There was no funding source for this study.

## Results

**Fig. 1:**
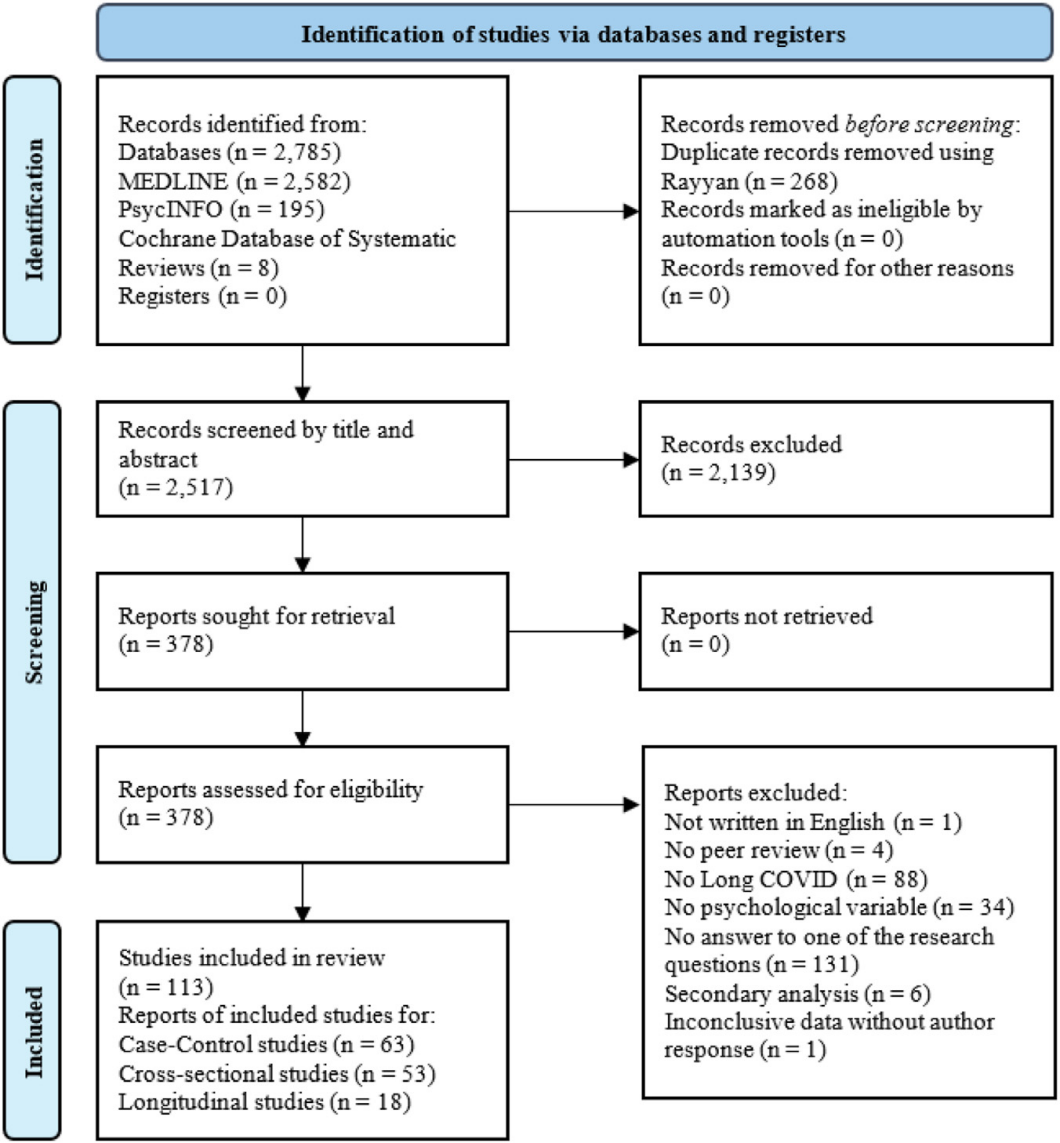
**PRISMA flow diagram**. Note. Some studies give information for more than one research question.

A PRISMA flow diagram of study selection is displayed in Fig. 1. Descriptive information of all included studies is shown in eTable 1 in the Supplement. Three results tables split by RQs 1–3 can be found in the Supplement (eTables 2–4).

### Study characteristics and overall results

A final of 113 studies (see eReferences) were included in the review, assessing 312,831 patients with Long COVID. Sixty-eight studies were conducted in Europe (most frequent: Germany), followed by Northern America (32; most frequent: USA), Asia (24; most frequent: China), Latin America (16; most frequent: Brazil), and Australia (1), with several studies involving multiple countries. 63 studies provided data on at least one psychological construct in cross-sectional group comparisons, 53 in cross-sectional associations, and 18 longitudinal format (eTable 1 in the Supplement). According to the categories of the PSY-PSS framework,^28^ most studies (20) investigated signs of psychopathology, followed by cognitive factors and personality & interpersonal factors (11 each), affective and behavioural factors (5 each), and psychophysiological factors (4). For an overview of all 58 psychological features examined in total, their respective category, and their number of investigations, separated by research question, see eTable 5 in the Supplement. The available evidence for each psychological concept assessed in patients with Long COVID in each included study design is synthesised in Table 1.

**Table 1:** Synthesis of evidence for all psychological variables by research question.

Psychological variable	RQ1 (cross-sectional group comparisons)	RQ2 (cross-sectional associations)		RQ3 (longitudinal data)	
		Sig. association	No sig. association	Sig. prediction	No sig. prediction
Depression	LC > CO (27)^37^, ^38^, ^39^, ^40^, ^41^, ^42^, ^43^, ^44^, ^45^, ^46^, ^47^, ^48^, ^49^, ^50^, ^51^, ^52^, ^53^, ^54^, ^55^, ^56^, ^57^, ^58^, ^59^, ^60^, ^61^, ^62^, ^63^/HC (17)^37^^,^^44^, ^45^, ^46^^,^^55^^,^^56^^,^^61^^,^^64^, ^65^, ^66^, ^67^, ^68^, ^69^, ^70^, ^71^, ^72^, ^73^ LC = CO (4)^37^^,^^38^^,^^74^^,^^75^/HC (4)^37^^,^^64^^,^^76^^,^^77^/FMS (1)^78^/CFS (1)^78^/PCS (1)^79^ LC < ME/CFS (1)^80^	QoL (9),^41^^,^^81^, ^82^, ^83^, ^84^, ^85^, ^86^, ^87^, ^88^ fatigue (6),^41^^,^^51^^,^^68^^,^^80^^,^^82^^,^^89^ impairment (5),^80^^,^^88^^,^^90^, ^91^, ^92^ LC (4),^54^^,^^59^^,^^93^^,^^94^ number of persistent symptoms (4),^40^^,^^84^^,^^95^^,^^96^ cognitive deficits (4),^97^, ^98^, ^99^, ^100^ pain (2),^80^^,^^83^ gastrointestinal symptoms (1)^95^	LC (3),^40^^,^^49^^,^^101^ number of persistent symptoms (1),^52^ cognitive deficits (1)^102^	LC (7),^6^^,^^8^^,^^67^^,^^103^, ^104^, ^105^, ^106^ impairment (2),^6^^,^^106^ fatigue (2)^39^^,^^107^	LC (2),^50^^,^^108^ fatigue (1),^109^ pain (1),^109^ respiratory symptoms (1)^109^
Anxiety	LC > CO (18)^37^^,^^38^^,^^40^^,^^42^, ^43^, ^44^, ^45^^,^^47^^,^^48^^,^^52^^,^^54^^,^^55^^,^^57^^,^^58^^,^^61^, ^62^, ^63^^,^^110^/HC (13)^37^^,^^45^^,^^46^^,^^55^^,^^61^^,^^64^, ^65^, ^66^, ^67^, ^68^^,^^71^, ^72^, ^73^ LC = CO (6)^37^^,^^38^^,^^50^^,^^74^^,^^75^^,^^111^/HC (4)^37^^,^^44^^,^^64^^,^^77^/ME/CFS (1)^80^/FMS (1)^78^/CFS (1)^78^/PCS (1)^79^ LC < ME/CFS (1)^80^	QoL (5),^81^^,^^84^^,^^86^, ^87^, ^88^ number of persistent symptoms (5),^40^^,^^52^^,^^84^^,^^95^^,^^96^ fatigue (4),^68^^,^^80^^,^^82^^,^^89^ cognitive deficits (4),^97^, ^98^, ^99^, ^100^ LC (3),^40^^,^^54^^,^^93^ impairment (2),^91^^,^^92^ pain (2),^80^^,^^83^ gastrointestinal symptoms (1)^95^	QoL (4),^82^^,^^83^^,^^85^^,^^112^ impairment (1),^80^ fatigue (1),^80^ pain (1)^80^	LC (3),^6^^,^^8^^,^^110^ impairment (1),^6^ cognitive deficits (1)^107^	LC (3),^50^^,^^67^^,^^108^ fatigue (1),^109^ pain (1),^109^ respiratory symptoms (1)^109^
Physical activity	LC = CO (2)^53^^,^^113^/HC (1)^114^ LC < CO (3)^45^^,^^56^^,^^115^/HC (4)^45^^,^^56^^,^^67^^,^^76^	LC (1),^116^ symptom severity (1),^56^ impairment (1),^56^ QoL (1),^117^ overall health (1)^56^	LC (3),^113^^,^^116^^,^^118^ QoL (2)^86^^,^^117^	LC (1)^119^	–
Depression/Anxiety	LC > CO (6)^44^^,^^63^^,^^120^, ^121^, ^122^, ^123^/HC (2)^44^^,^^124^ LC = CO (1)^125^/HC (1)^114^	LC (2),^126^^,^^127^ fatigue (2),^51^^,^^128^ impairment (1),^126^ QoL (1)^117^	–	LC (1),^121^ respiratory symptoms (1)^129^	LC (1)^125^
PTSD	LC > CO (4)^44^^,^^47^^,^^63^^,^^122^/HC (2)^44^^,^^73^ LC = CO (2)^48^^,^^75^	LC (1),^93^ number of persistent symptoms (1),^95^ gastrointestinal symptoms (1)^95^	fatigue (1)^130^	–	–
Stress	LC > CO (2)^45^^,^^58^/HC (2)^45^^,^^64^ LC = CO (3)^52^^,^^75^^,^^113^/HC (1)^77^/PCS (1)^79^	QoL (1)^81^	QoL (1)^81^	LC (2),^6^^,^^106^ impairment (2)^6^^,^^106^	–
History of mental health disorders	LC > CO (3)^39^^,^^131^^,^^132^ LC = CO (2)^39^^,^^74^ LC < CO (1)^115^	LC (3)^131^, ^132^, ^133^	LC (1)^131^		fatigue (1)^39^
Alcohol abuse/dependence	LC = CO (2)^60^^,^^134^/PCS (1)^79^	–	–	–	–
Suicidal ideation	LC > CO (1)^47^ LC = PCS (1)^79^ LC < ME/CFS (1)^80^	impairment (1),^80^ fatigue (1),^80^ pain (1)^80^	–	–	–
Mood disorders	LC > CO (1)^37^/HC (2)^37^^,^^76^	–	cognitive deficits (1)^135^	dizziness (1),^136^ change in appetite (1)^136^	fatigue (1),^136^ cognitive deficits (1),^136^ sleep disturbance (1)^136^
Reaction to severe stress and adjustment disorders	LC > HC (1)^72^ LC = CO (1)^110^	–	–	LC (1)^108^	LC (1)^110^
Mania	LC = PCS (1)^79^	–	–	–	–
Schizophrenia/Psychosis/Paranoia	LC < PCS (1)^79^	–	–	LC (1)^103^	–
ADHD	–	–	–	–	cognitive deficits (1)^136^
Disorders of psychological development	LC = CO (1)^110^	–	–	–	LC (1)^110^
Nicotine abuse/dependence	–	–	–	LC (1)^103^	LC (1)^108^
Drug problems/substance abuse	LC = PCS (1)^79^	–	–	LC (1)^103^	–
Borderline features	LC = PCS (1)^79^	–	–	–	–
Antisocial features	LC = PCS (1)^79^	–	–	–	–
Thought disorder	LC > CO (1)^37^/HC (1)^37^	–	–	–	–
Depression medications	LC = HC (1)^76^	–	LC (1)^137^	–	–
Anxiety medications	–	LC (1)^137^	–	–	–
Loneliness	–	–	–	LC (2)^6^^,^^119^	impairment (1)^6^
Anger	LC = HC (1)^76^	–	–	–	–
Positive trait affect	–	–	–	LC (1),^106^ impairment (1)^106^	–
Fear of COVID-19	LC > CO (1)^62^ LC = CO (1)^138^ LC < HC (1)^138^	LC (1)^139^	LC (1)^137^	LC (1),^106^ impairment (1)^106^	–
Maternal health anxiety	LC > CO (1)^138^/HC (1)^138^	–	–	–	–
Worry about COVID-19	–	–	–	LC (1),^6^ impairment (1)^6^	–
Catastrophising	LC > CO (1)^45^/HC (1)^45^ LC = FMS (1)^78^ LC < CFS (1)^78^	QoL (1)^83^	pain (1)^83^	–	–
Negative cognitions	LC = HC (1)^114^	–	–	–	–
Personal control	–	fatigue (1)^82^	QoL (1)^82^	–	–
Treatment control	–	fatigue (1)^82^	QoL (1)^82^	–	–
Treatment rejection	LC = PCS (1)^79^	–	–	–	–
Coherence	–	fatigue (1)^82^	QoL (1)^82^	–	–
Self-compassion	–	symptom severity (1),^140^ psychosocial impact (1)^140^	–	–	–
Emotional representation	–	fatigue (1)^82^	QoL (1)^82^	–	–
Illness identity	–	QoL (1),^82^ fatigue (1)^82^	–	–	–
Walking self-efficacy	LC = HC (1)^76^	–	–	–	–
Fear avoidance/Kinesiophobia	LC > CO (1)^45^/HC (1)^45^ LC = CFS (1)^78^ LC < FMS (1)^78^	–	QoL (1),^83^ pain (1)^83^	–	–
Avoidance	LC = HC (1)^114^	–	–	–	–
Connection with friends	LC = HC (1)^114^	–	LC (1),^114^ impairment (1)^114^	–	–
Sedentary behaviour	–	LC (1),^118^ fatigue (1)^118^	–	–	–
Neuroticism/emotional instability	LC > CO (1)^141^/HC (1)^142^	–	fatigue (1)^142^	–	–
Extraversion	LC < HC (1)^142^	–	fatigue (1)^142^	–	–
Openness	LC = HC (1)^142^	–	fatigue (1)^142^	–	–
Conscientiousness	LC = HC (1)^142^	–	fatigue (1)^142^	–	–
Agreeableness	LC < HC (1)^142^	–	fatigue (1)^142^	–	–
Resilience	LC = CO (1)^75^ LC < CO (1)^141^	–	LC (2)^137^^,^^143^	–	–
Psychological flexibility	–	symptom severity (1),^140^ psychosocial impact (1)^140^	–	–	–
Aggression	LC = PCS (1)^79^	–	–	–	–
Dominance	LC = PCS (1)^79^	–	–	–	–
Warmth	LC = PCS (1)^79^	–	–	–	–
Stigma	LC > CO (1)^144^	–	–	–	–
Life events	LC > CO (1)^141^ LC = CO (1)^141^	–	–	fatigue (1)^119^	–
Nonsupport	LC = PCS (1)^79^	–	–	–	–
Psychological distress	LC > CO (1)^132^ LC = HC (1)^145^	cognitive deficits (1)^146^	–	–	–
Central sensitisation	LC > CO (1)^45^/HC (1)^45^	QoL (2),^83^^,^^85^ pain (1)^83^	–	–	–
Multisensory sensitivity/somatosensory amplification	LC = CFS (1)^78^ LC < FMS (1)^78^	–	–	–	–

*Note*. Numbers in parentheses refer to the frequency of studies. For RQ1, directions of effect are shown. Some studies provide results for more than one comparison group or outcome. RQ, research question; LC, patients with Long COVID; CO, patients after COVID-19 without Long COVID; HC, healthy controls without a history of COVID-19; ME/CFS, patients with myalgic encephalomyelitis/chronic fatigue syndrome; FMS, patients with fibromyalgia syndrome; PCS, patients with post-concussion syndrome; QoL, quality of life; sig., significant; threshold for statistical significance *p* < 0.05.

For RQ1, meta-analyses could be calculated for depression (17 categorical and 24 continuous comparisons), anxiety (15 categorical and 16 continuous comparisons), physical activity (6 continuous comparisons), and stress (5 continuous comparisons). The variable “depression/anxiety”, which comprises measures of either “depression or anxiety” or “depression and anxiety” as reported in the included studies, was not meta-analysed due to the higher informative value of depression and anxiety alone. For RQ2 and RQ3, it was not possible to conduct meta-analyses for any psychological factor due to highly heterogeneous outcome variables and statistical coefficients for both categorical and continuous data. The diverse operationalisation of psychological variables can be viewed for those with at least five observations, along with the respective control groups (for RQ1) or outcomes of the studies (for RQ2 and RQ3), in eTables 6–17 in the Supplement.

### Research question 1: In which psychological factors do patients with Long COVID report significantly higher values compared to control groups (cross-sectional group comparisons)?

Patients with Long COVID were compared to patients after SARS-CoV-2 infection but without Long COVID in 44 studies, to healthy controls without a history of COVID-19 in 24 studies, to patients with chronic fatigue syndrome in two studies, and to patients with fibromyalgia and post-concussion syndrome in one study respectively. The most studied psychological factors within any control group were depression and anxiety, followed by physical activity, a combination of depression and/or anxiety, posttraumatic stress disorder, stress, and history of mental health disorders. For all other searched psychological constructs, evidence was very limited, with a maximum of three studies per variable (eTable 2 in the Supplement). The majority of studies found almost all maladaptive psychological factors to be significantly higher and more frequent in patients with Long COVID than in both patients after SARS-CoV-2 infection who did not develop Long COVID and in healthy controls without a history of COVID-19, with no study reporting the opposite direction. Lower scores for depression, anxiety, history of mental health disorders, paranoia, catastrophising, kinesiophobia, somatosensory amplification, and suicidal ideation were reported in patients with Long COVID compared to controls in one study each, all but one of which referred to comparisons to a control group with other persistent somatic symptoms (chronic fatigue, fibromyalgia, post-concussion syndrome). Solely no significant differences between patients with Long COVID and controls were found for alcohol abuse, drug problems, disorders of psychological development, mania, borderline features, depression medications, walking self-efficacy (confidence in the ability to walk at a certain pace for a certain time without stopping), openness, conscientiousness, connection with friends, warmth, antisocial features, anger, aggression, dominance, negative cognitions, avoidance, nonsupport, and treatment rejection. However, it should be noted that, with the exception of alcohol abuse, all of these factors were only examined in one study each. Some studies reported both significant and non-significant differences between groups for the same psychological variable depending on measures (psychometric questionnaire data vs. clinical diagnosis of depression and anxiety^37^^,^^64^; adverse life events in the past year vs. earlier),^141^ follow-up time point on symptoms after SARS-CoV-2 infection,^38^^,^^39^ and different aspects of a construct (trait vs. state anxiety; Table 1: RQ1).^80^

### Meta-analysis of cross-sectional studies with control groups

**Fig. 2:**
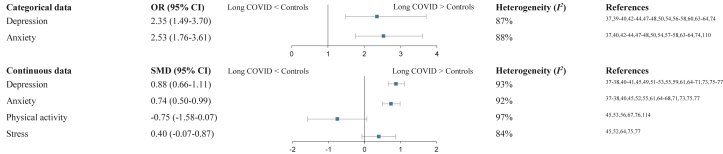
**Pooled data from random-effects meta-analyses of cross-sectional studies with control groups**. Note. OR, odds ratio; SMD, standardised mean difference; CI, confidence interval.

Meeting the required criteria (≥5 studies with valid data in the same format) allowed meta-analyses to be calculated for depression, anxiety (both categorical and continuous comparisons), physical activity, and stress (continuous comparisons). Pooled meta-analytic evidence for each construct is presented in Fig. 2. Sensitivity analyses led to almost identical results for all the meta-analyses executed, demonstrating model robustness (see eTable 18 in the Supplement). For all forest and funnel plots, see eFigures 1–12 in the Supplement.

#### Depression

Meta-analysis of continuous data revealed depression to be significantly higher in patients with Long COVID compared to controls (*z* = 7.73; *p* < 0.001; eFigure 1 in the Supplement). High between-study heterogeneity (*I*^*2*^ = 93%, *p* < 0.001) and a significant risk for publication bias were found (*p* < 0.01). After applying the trim-and-fill method, the model remained significant with a SMD of 0.37 (95% CI, 0.07–0.67). Meta-analysis of categorical data indicated a significant association between Long COVID and depression (*z* = 3.69; *p* < 0.001; eFigure 2 in the Supplement). Again, heterogeneity between studies was high (*I*^*2*^ = 87%, *p* < 0.001). Publication bias risk was not significant (*p* > 0.05).

#### Anxiety

Meta-analysis of continuous data showed significantly higher anxiety in patients with Long COVID compared to controls (*z* = 5.98; *p* < 0.001; eFigure 3 in the Supplement). Heterogeneity testing yielded high between-study variability (*I*^*2*^ = 92%, *p* < 0.001). Risk for publication bias was significant (*p* < 0.01) and the model remained significant after the trim-and-fill method (SMD = 0.32; 95% CI, 0.002–0.63). Meta-analysis of categorical data resulted in a significant association between Long COVID and anxiety (*z* = 5.07; *p* < 0.001; eFigure 4 in the Supplement). High heterogeneity between studies (*I*^*2*^ = 88%, *p* < 0.001) was found. Risk for publication bias was not significant (*p* > 0.05).

#### Physical activity

The model's overall effect was not significant (*p* = 0.07; eFigure 5 in the Supplement). Heterogeneity between studies was high (*I*^*2*^ = 97%, *p* < 0.001). No risk for publication bias was found (*p* > 0.05).

#### Stress

The model's overall effect was not significant (*p* = 0.10; eFigure 6 in the Supplement). Heterogeneity between studies was high (*I*^*2*^ = 84%, *p* < 0.001). No risk for publication bias was found (*p* > 0.05).

### Research question 2: Which psychological factors are significantly associated with the presence of Long COVID or condition-relevant outcomes such as symptom severity, impairment, quality of life, and healthcare utilisation (cross-sectional associations)?

Cross-sectional associations with psychological variables were investigated in 16 studies for presence of Long COVID, in 11 for quality of life, in 8 for impairment, in 4 for number of persistent somatic symptoms, and 2 for symptom severity. In other studies, single Long COVID symptoms such as fatigue were used as outcomes. No study examined healthcare utilisation. Depression and anxiety were by far the most frequently investigated factors, followed by physical activity and a combination of depression and/or anxiety. All other constructs were examined in no more than three studies each (eTable 3 in the Supplement). In general, most studies demonstrated significant associations between maladaptive psychological factors and Long COVID or condition-relevant outcomes. Significant negative associations between psychological factors and condition-relevant outcomes were found in only three studies in total, with these relating to depression, anxiety, suicidal ideation, and illness identity. Solely no significant associations were reported in two studies for resilience and in one study each for the personality factors extraversion, openness, conscientiousness, agreeableness, and neuroticism as well as for connection with friends, kinesiophobia, depression medications, and mood disorders. Some studies reported both significant and non-significant results depending on measures (depression reported as mean vs. cut-off),^40^ outcomes (mental vs. physical quality of life),^81^^,^^117^ acute COVID-19 severity,^131^ and aspects of a construct (state vs. trait anxiety^80^; physical activity vs. inactivity; Table 1: RQ2).^116^

### Research question 3: Which psychological factors significantly predict the development or maintenance of Long COVID (longitudinal data)?

Prospective relations with psychological factors were assessed in 16 studies for Long COVID and in two for impairment. Other outcomes were individual Long COVID symptoms. No study investigated symptom severity, quality of life, or healthcare utilisation. Again, depression and anxiety were assessed most frequently. A maximum of three longitudinal studies each were conducted on other variables (eTable 4 in the Supplement). The majority of studies showed maladaptive psychological factors to be significant predictors of Long COVID or impairment. The only exception was one study in which psychosis, tobacco smoking, and substance abuse significantly predicted a lower risk of Long COVID. For disorders of psychological development and Attention-Deficit Hyperactivity Disorder (ADHD), only non-significant results were reported (in one study each). Results for all psychological factors can be found in Table 1, RQ3.

### Risk of bias of included studies

Twelve studies providing psychological data for cross-sectional group comparisons (e.g., in case–control studies) were of good quality, 44 were fair, and seven were poor. For data on cross–sectional associations, 11 studies were of good quality, 41 were fair, and one was poor. Regarding longitudinal data, 13 studies were of good quality, five were fair, and none was poor. For individual quality ratings by study design, see eAppendix 2 in the Supplement.

## Discussion

This is the first systematic review and meta-analysis of a comprehensive range of pre-defined psychological factors with potential relevance for the development and/or maintenance of Long COVID and associated symptom severity, impairment, quality of life, and healthcare utilisation. Consequently, this study aimed to identify modifiable variables that could improve the understanding of Long COVID and have therapeutic relevance for affected patients. Overall, 113 studies of varying methodology provided original data for 58 psychological constructs in total (eTable 1 in the Supplement). Findings highlight above all the importance of depression and anxiety, which could be confirmed by meta-analyses of cross-sectional studies providing comparisons between patients with Long COVID and controls. Meta-analyses of physical activity and stress with only a small number of studies yielded no mean difference between patients with Long COVID and controls. The sound evidence found for depression and anxiety is in line with earlier systematic reviews and meta-analyses on Long COVID^7^^,^^26^^,^^27^ and also corroborates a biopsychosocial model of persistent somatic symptoms in general which proposes both to be important maintaining and aggravating factors.^13^, ^14^, ^15^ However, the evidence base for psychological features other than depression and anxiety is scarce, which hinders conclusions to be drawn regarding their relevance for Long COVID.

A narrative synthesis across all study designs indicates the investigated psychological constructs to be associated with and predictive of Long COVID, with only seven studies reporting contrary results.^41^^,^^78^, ^79^, ^80^^,^^82^^,^^103^^,^^115^ The latter mainly concern mental health differences between patients with Long COVID and those with other persistent somatic symptoms (chronic fatigue,^78^^,^^80^ fibromyalgia,^78^ and post-concussion syndrome).^79^ Considering the phenotypic resemblance between Long COVID and chronic fatigue syndrome in particular,^147^ studies with control groups should include individuals with fatigue but no history of COVID-19 to shed further light on the role of SARS-CoV-2 infection.^119^^,^^148^ Due to the high infection rates worldwide, such comparisons are becoming increasingly difficult though.

The vast majority of the evidence available to date relates to the role of depression and anxiety in Long COVID. However, the spectrum of psychological criteria known to be involved in chronic somatic symptoms is broader.^149^ Also with regard to other post-acute infection syndromes, findings point to the importance of various psychological constructs like neuroticism or attributional styles.^150^^,^^151^ In the course of this review, no studies were found on potentially relevant psychological variables such as alexithymia, emotion regulation, illness behaviour, symptom perception, and expectations.^28^^,^^152^ In contrast to biomedical and sociodemographic risk factors for Long COVID,^7^ psychological factors offer the advantage of being modifiable through interventions. Therefore, diversity of assessed constructs should be increased in Long COVID research, as is already the case with other forms of persistent somatic symptoms,^153^ to establish further evidence-based treatment targets and inform guidelines.^1^ A more solid foundation of robust research findings leading to thorough explanation models could help alleviate skepticism about the importance of psychological processes and disputes about their legitimacy in the context of Long COVID.^6^^,^^9^

In this review, high heterogeneity between publications and, in some cases, methodological shortcomings (e.g., insufficient reporting of statistical parameters such as sum scores or mean values) were observed. In addition to the monthly volume of new publications on the topic, widely varying methodologies (e.g., measures of psychological variables and outcomes such as impairment, methods of analysis, and reported statistical parameters) complicate the consolidation of evidence in this rapidly growing field of research and question the validity of results. Guidelines for research in the field of Long COVID, including harmonisation of data collection, could improve methodological quality and comparability of future studies and pooling of data. A core battery of instruments to standardise the assessment of psychological aspects in Long COVID, for instance, would not only facilitate conclusions on their contribution to symptom persistence in the interplay between biomedical, psychological, and social factors, but also on the effectiveness of therapies.^154^ Beyond that, more prospective analyses of representative cohorts are greatly needed to determine the predictive value of psychological features for trajectories of somatic symptoms after COVID-19, thereby improving our etiological understanding of Long COVID and the long-term impact of psychological burden on symptom severity. Fortunately, an increasing number of studies that strengthen our findings are currently being published.^148^^,^^155^

In contrast to previous reviews on Long COVID, we included a wide range of psychological factors in our literature search by applying the PSY-PSS framework,^28^ which provides the first comprehensive list of potentially relevant psychological features in persistent somatic symptoms and related conditions such as Long COVID. One limitation concerns the scarcity of studies on Long COVID investigating psychological constructs other than depression and anxiety. While there is no doubt about their relevance in mental health research,^156^ exclusively focusing on depression and anxiety prevents informed conclusions about the relative importance of other psychological risk factors for Long COVID. This disparity may also be due to the fact that valid self-report measures are widely available for depression and anxiety,^157^^,^^158^ whereas clear recommendations on which other psychological variables should be assessed are still lacking for Long COVID.^31^ Great methodological heterogeneity between studies further hindered the meta-analytical synthesis of existing data. In some studies, only significant effects were reported. Such publication bias was discovered in two of our meta-analyses. Individual studies employed innovative methods, for instance allowing for intra-individual trajectories over time, which unfortunately could not be meta-analysed.^159^ Based on the results of our meta-analyses, no inferences can be made as to causal relations between maladaptive psychological factors and Long COVID. This requires further prospective studies. In this review, it was also not possible to investigate any interactions between psychological constructs — such as depression and anxiety — and biological factors in the aetiology of Long COVID, e.g., via inflammatory processes,^160^^,^^161^ which could inform the further development of a biopsychosocial explanatory model for Long COVID. Severity of SARS-Cov-2 infection, virus variant, vaccination status, pre-existing or comorbid somatic and/or mental diseases, and duration of Long COVID are important factors to be considered in the interpretation of our results. However, many studies did not provide any information on these factors. Accounting for these parameters also exceeded the scope of our study where we defined Long COVID as per NICE guideline, i.e., purely symptom-based, which does not take any of the above listed factors into account.^1^ Females have a higher risk of developing Long COVID^7^; however, we did not consider sex and gender-specific differences regarding psychological variables in this review. Overall, the ratio of female and male patients was relatively balanced across all included studies (58% females). In addition, we did not account for race or ethnicity of patients in the interpretation of our results and therefore cannot draw any conclusions regarding the racial or ethnic representativeness of study populations. We extracted data reported in manuscripts or supplements. If values were inconsistent or incorrect and we did not receive an author response, the corresponding studies were excluded. It should also be noted that we only included English-language studies and only studies up to January 2024, but new studies on Long COVID are constantly being published. We extracted additional psychological constructs that were examined in the identified studies but are not included in the PSY-PSS framework list (e.g., anger, self-compassion). Our database search, however, was based on the original list of psychological variables^28^ and did not cover features such as psychodynamic factors added in a newer version.^162^ Regarding the definition of Long COVID, we used the NICE guideline,^1^ i.e., symptom duration of at least 4 weeks, instead of the stricter WHO criteria of post-COVID-19 condition,^32^ i.e., symptom duration of at least 2 months, to take more findings into account. In the majority of identified studies, the time criterion of the WHO criteria was met (eTable 1 in the Supplement). However, no distinction was made in our interpretation of the results.

This review summarises the current evidence for psychological factors of importance for the understanding and tailored treatment of Long COVID. There is sufficient evidence for depression and anxiety to consider these variables in interventions; however, many other relevant features either miss empirical investigation or have hardly been tested in prospective designs. Psychological factors, even depression and anxiety, have insufficiently been addressed in the current mechanistic understanding of Long COVID pathophysiolog.^161^ While this review does not allow causal inferences regarding the role of psychological factors for Long COVID, it provides a starting point for further research. More longitudinal studies and experimental research using harmonised methods are needed to advance etiologic models of Long COVID and help differentiate between patient subtypes.^163^ In light of the great burden on affected patients,^164^ driving forward multidisciplinary treatment for Long COVID based on a biopsychosocial perspective is of high clinical and societal relevance.

## Contributors

MSM and AT conceptualised and supervised the study. PE run the database search. PE, MR, and CS screened titles and abstracts. Full texts were reviewed by PE, MR, CS, SS, AT, and MSM. PE, MR, CS, and AT extracted the data. PE and AT assessed study quality and bias. PE performed the meta-analysis and drafted the manuscript. AT, MSM, BL, SS, MR, CS, and PE revised and approved the final manuscript. All authors had full access to all the data in the study and had final responsibility for the decision to submit for publication.

## Data sharing statement

The authors confirm that the data supporting the findings of this study are available within the article and its Supplementary materials.

## Declaration of interests

PE reports research funding (no personal honoraria) from the German Research Foundation. SS reports research funding (no personal honoraria) from the German Research Foundation and the German Heart Foundation/German Foundation of Heart Research. BL reports research funding (no personal honoraria) from the German Research Foundation, the German Federal Ministry of Education and Research, the German Innovation Committee at the Joint Federal Committee, the European Commission's Horizon 2020 Framework Programme, the European Joint Programme for Rare Diseases (EJP), the Ministry of Science, Research and Equality of the Free and Hanseatic City of Hamburg, Germany, and the Foundation Psychosomatics of Spinal Diseases, Stuttgart, Germany. He received remunerations for several scientific book articles from various book publishers, from the Norddeutscher Rundfunk (NDR) for interviews in medical knowledge programmes on public television, and as a committee member from Aarhus University, Denmark. He received travel expenses from the European Association of Psychosomatic Medicine (EAPM), and accommodation and meals from the Societatea de Medicina Biopsyhosociala, Romania, for a presentation at the EAPM Academy at the Conferința Națională de Psihosomatică, Cluj-Napoca, Romania, October 2023. He received remuneration and travel expenses for lecture at the Lindauer Psychotherapiewochen, April 2024. He is President of the German College of Psychosomatic Medicine (DKPM) (unpaid) since March 2024 and was a member of the Board of the European Association of Psychosomatic Medicine (EAPM) (unpaid) until 2022. He is member of the EIFFEL Study Oversight Committee (unpaid). AT reports research funding (no personal honoraria) from the German Research Foundation. She received remunerations for scientific book articles. MSM reports research funding (no personal honoraria) from the German Research Foundation and the German Academic Research Service. She received remunerations for scientific book articles, from the Norddeutscher Rundfunk (NDR) for interviews in medical knowledge programmes on public television, for post graduate training for psychotherapy, and for the review of a grant proposal at the University of Toledo, USA. She is member of the Scientific Advisory Board of PKD Cure e.V. (unpaid). She is Executive Board Member and Vice-Treasurer of the European Association of Psychosomatic Medicine (EAPM) (unpaid).

## Appendix A Supplementary data

Supplement

eAppendix 2
