# Supplementary material for: Psychological factors associated with Long COVID: a systematic review and meta-analysis
Source: eClinicalMedicine. 2024 Jul 26;74:102756. doi: 10.1016/j.eclinm.2024.102756 (PMC11701445; doi:10.1016/j.eclinm.2024.102756)
Supplement: Supplement [file mmc1.docx]

**Supplemental Online Content**

Psychological Factors Associated With Long COVID: A Systematic Review and Meta-Analysis

Engelmann, P., Reinke, M., Stein, C., Salzmann, S., Löwe, B., Toussaint, A., &
Shedden-Mora, M.

2024

[**eAppendix 1. Search paradigm** 2](#_Toc169091082)

[**eAppendix 2. Quality rating (see Excel file)** 7](#_Toc169091083)

[**eTable 1. Descriptive information for included studies** 7](#_Toc169091084)

[**eTable 2. Overview of included studies providing cross-sectional group comparisons on psychological variables (RQ1)** 80](#_Toc169091085)

[**eTable 3. Overview of included studies providing cross-sectional associations with psychological variables (RQ2)** 117](#_Toc169091086)

[**eTable 4. Overview of included studies providing longitudinal data on psychological variables (RQ3)** 155](#_Toc169091087)

[**eTable 5. Psychological variables, respective category, and number of studies in which they were investigated by RQ** 169](#_Toc169091088)

[**eTables 6-12. Operationalisation of psychological variables with at least five observations in studies answering RQ1 and respective control groups** 173](#_Toc169091089)

[**eTables 13-15. Operationalisation of psychological variables with at least five observations in studies answering RQ2 and respective outcomes variables** 184](#_Toc169091090)

[**eTables 16-17. Operationalisation of psychological variables with at least five observations in studies answering RQ3 and respective outcomes variables** 190](#_Toc169091091)

[**eTable 18. Sensitivity analyses for meta-analyses of cross-sectional studies with control groups** 192](#_Toc169091092)

[**eFigure 1. Forest plot depicting meta-analysis of cross-sectional studies with control groups investigating depression continuously** 194](#_Toc169091093)

[**eFigure 2. Forest plot depicting meta-analysis of cross-sectional studies with control groups investigating depression categorically** 195](#_Toc169091094)

[**eFigure 3. Forest plot depicting meta-analysis of cross-sectional studies with control groups investigating anxiety continuously** 196](#_Toc169091095)

[**eFigure 4. Forest plot depicting meta-analysis of cross-sectional studies with control groups investigating anxiety categorically** 197](#_Toc169091096)

[**eFigure 5. Forest plot depicting meta-analysis of cross-sectional studies with control groups investigating physical activity** 198](#_Toc169091097)

[**eFigure 6. Forest plot depicting meta-analysis of cross-sectional studies with control groups investigating stress** 198](#_Toc169091098)

[**eFigures 7-12. Funnel plots** 199](#_Toc169091099)

[**eReferences** 205](#_Toc169091100)

## **eAppendix 1. Search paradigm**

| **Database** | **Search string** |
| --- | --- |
| MEDLINE (via PubMed) | ("Long COVID"[Title/Abstract] OR "Long-COVID"[Title/Abstract] OR "Post COVID-19 condition"[Title/Abstract] OR "Post-COVID-19 condition"[Title/Abstract] OR "Post COVID conditions"[Title/Abstract] OR "Post-COVID conditions"[Title/Abstract] OR "Post COVID syndrome"[Title/Abstract] OR "Post-COVID syndrome"[Title/Abstract] OR "Post COVID-19 syndrome"[Title/Abstract] OR "Post-COVID-19 syndrome"[Title/Abstract] OR "Post-acute COVID-19 syndrome"[Title/Abstract] OR "Post-acute COVID syndrome"[Title/Abstract] OR "Post-acute sequelae of SARS-CoV-2 infection"[Title/Abstract] OR "Post-acute sequelae of COVID-19"[Title/Abstract] OR "Chronic COVID"[Title/Abstract] OR "Chronic-COVID"[Title/Abstract] OR "Chronic COVID syndrome"[Title/Abstract] OR "Chronic-COVID syndrome"[Title/Abstract] OR "Long-haul COVID"[Title/Abstract] OR "Long-haul COVID-19"[Title/Abstract] OR "Persistent COVID-19"[Title/Abstract]) AND ("alexithymia"[Title/Abstract] OR ("difficulty"[Title/Abstract] AND ("feeling*"[Title/Abstract] OR "emotion*"[Title/Abstract])) OR "emotion expression"[Title/Abstract] OR ("emotion"[Title/Abstract] AND ("regulation"[Title/Abstract] OR "control"[Title/Abstract])) OR "psychological conflict"[Title/Abstract] OR ("supression*"[Title/Abstract] AND "emotion*"[Title/Abstract]) OR "health anxiety"[Title/Abstract] OR "hypochondri*"[Title/Abstract] OR "health worr*"[Title/Abstract] OR ("health"[Title/Abstract] AND ("concern"[Title/Abstract] OR "concerns"[Title/Abstract])) OR "illness anxiety"[Title/Abstract] OR "illness worr*"[Title/Abstract] OR "illness concern*"[Title/Abstract] OR "loneliness"[Title/Abstract] OR "lonely"[Title/Abstract] OR "isolat*"[Title/Abstract] OR "negative affect*"[Title/Abstract] OR "positive affect*"[Title/Abstract] OR ("avoidance"[Title/Abstract] AND "activit*"[Title/Abstract]) OR ("avoiding"[Title/Abstract] AND "activit*"[Title/Abstract]) OR ("avoid"[Title/Abstract] AND "activit*"[Title/Abstract]) OR "fear-avoidance"[Title/Abstract] OR "fear avoidance"[Title/Abstract] OR "fear of movement"[Title/Abstract] OR "kinesiophobi*"[Title/Abstract] OR "physical activit*"[Title/Abstract] OR "exercis*"[Title/Abstract] OR "physical exertion"[Title/Abstract] OR "workout"[Title/Abstract] OR "work-out"[Title/Abstract] OR "physical effort"[Title/Abstract] OR "physical performance"[Title/Abstract] OR "reduced activity"[Title/Abstract] OR "inactivity"[Title/Abstract] OR "social withdrawal"[Title/Abstract] OR "lack social relationship*"[Title/Abstract] OR "lacking social relationship*"[Title/Abstract] OR "lacks social relationship*"[Title/Abstract] OR ("lack"[Title/Abstract] AND "interpersonal relation*"[Title/Abstract]) OR "body check*"[Title/Abstract] OR "body-check*"[Title/Abstract] OR "checking behavior*"[Title/Abstract] OR "checking behaviour*"[Title/Abstract] OR "self observation*"[Title/Abstract] OR "self-observation*"[Title/Abstract] OR "body observation*"[Title/Abstract] OR "body scan*"[Title/Abstract] OR "body-scan*"[Title/Abstract] OR "scanning behavior*"[Title/Abstract] OR "scanning behaviour*"[Title/Abstract] OR "self-inspection"[Title/Abstract] OR "self inspection"[Title/Abstract] OR "illness behaviour"[Title/Abstract] OR "help seeking"[Title/Abstract] OR "help-seeking"[Title/Abstract] OR "health seeking"[Title/Abstract] OR "health-seeking"[Title/Abstract] OR "seek medical help"[Title/Abstract] OR "seeking medical help"[Title/Abstract] OR "seeks medical help"[Title/Abstract] OR "seeks medical attention"[Title/Abstract] OR "seeking medical attention"[Title/Abstract] OR "seek medical attention"[Title/Abstract] OR "requesting diagnosis reevaluation"[Title/Abstract] OR "requesting diagnoses reevaluation"[Title/Abstract] OR "requesting diagnostic reevaluation"[Title/Abstract] OR "medical reassessment"[Title/Abstract] OR "reassurance"[Title/Abstract] OR "medical certainty"[Title/Abstract] OR "seek reassurance"[Title/Abstract] OR "reassurance-seeking"[Title/Abstract] OR "seeking reassurance"[Title/Abstract] OR "verify diagnos*"[Title/Abstract] OR "verified diagnos*"[Title/Abstract] OR " verifying diagnos*"[Title/Abstract] OR "valid diagnos*"[Title/Abstract] OR "validate diagnos*"[Title/Abstract] OR "validity diagnos*"[Title/Abstract] OR "confirm diagnos*"[Title/Abstract] OR "time symptom*"[Title/Abstract] OR "time complaint*"[Title/Abstract] OR "energy complaint*"[Title/Abstract] OR "energy symptom*"[Title/Abstract] OR "health-care utilization"[Title/Abstract] OR "health care utilization"[Title/Abstract] OR "health-care use"[Title/Abstract] OR "healthcare use"[Title/Abstract] OR "medication"[Title/Abstract] OR "doctor-hopping"[Title/Abstract] OR "doctor hopping"[Title/Abstract] OR "repeated doctor visits"[Title/Abstract] OR "repeated medical visits"[Title/Abstract] OR "media use"[Title/Abstract] OR "news use"[Title/Abstract] OR "social media"[Title/Abstract] OR ("information"[Title/Abstract] AND ("search*"[Title/Abstract] OR "seek*"[Title/Abstract])) OR "cyberchondria"[Title/Abstract] OR "self monitor*"[Title/Abstract] OR "self-monitor*"[Title/Abstract] OR "attention"[Title/Abstract] OR "attention* bias*"[Title/Abstract] OR "cognitive bias*"[Title/Abstract] OR "distraction"[Title/Abstract] OR "disturbance"[Title/Abstract] OR "interference"[Title/Abstract] OR "interruption"[Title/Abstract] OR "attention"[Title/Abstract] OR "sensory filtering"[Title/Abstract] OR "stimulus filtering"[Title/Abstract] OR "sensory gating"[Title/Abstract] OR "sensory processing"[Title/Abstract] OR "peripheral filtering"[Title/Abstract] OR "central filtering"[Title/Abstract] OR "body vigilance"[Title/Abstract] OR "internal cues"[Title/Abstract] OR "bod* sensation"[Title/Abstract] OR "*vigilan*"[Title/Abstract] OR "selective recall"[Title/Abstract] OR "memory bias*"[Title/Abstract] OR "locus of control"[Title/Abstract] OR "bodily weakness"[Title/Abstract] OR ("trust"[Title/Abstract] AND "body"[Title/Abstract]) OR "catastrophi*"[Title/Abstract] OR ("overestimat*"[Title/Abstract] AND "negative consequences"[Title/Abstract]) OR "attribution*"[Title/Abstract] OR ("causal"[Title/Abstract] AND "attribution*"[Title/Abstract]) OR ("attribution*"[Title/Abstract] AND ("organic"[Title/Abstract] OR "somatic"[Title/Abstract])) OR (("cause"[Title/Abstract] OR "causation"[Title/Abstract]) AND ("biomedical"[Title/Abstract] OR "organic"[Title/Abstract])) OR ("somatic"[Title/Abstract] AND "illness belief"[Title/Abstract]) OR ("expect*"[Title/Abstract] AND "side effect*"[Title/Abstract]) OR ("expect*"[Title/Abstract] AND "side-effect*"[Title/Abstract]) OR "nocebo"[Title/Abstract] OR "optimism"[Title/Abstract] OR "positive thinking"[Title/Abstract] OR "hope"[Title/Abstract] OR "self-efficacy"[Title/Abstract] OR ("treatment"[Title/Abstract] AND "expectation*"[Title/Abstract]) OR "expect*"[Title/Abstract] OR "anticipat*"[Title/Abstract] OR ("symptom*"[Title/Abstract] AND "expectation"[Title/Abstract]) OR ("health"[Title/Abstract] AND ("worr*"[Title/Abstract] OR "concern"[Title/Abstract])) OR "hypochondri*"[Title/Abstract] OR ("illness"[Title/Abstract] AND ("worr*"[Title/Abstract] OR "concern*"[Title/Abstract])) OR "demoralisation"[Title/Abstract] OR "demoralization"[Title/Abstract] OR "desperation"[Title/Abstract] OR "despair"[Title/Abstract] OR "loss of hope"[Title/Abstract] OR "hopeless*"[Title/Abstract] OR "helpless*"[Title/Abstract] OR "illness belief*"[Title/Abstract] OR "symptom belief*"[Title/Abstract] OR "symptom perception"[Title/Abstract] OR "illness perception"[Title/Abstract] OR "illness representation"[Title/Abstract] OR "symptom representation"[Title/Abstract] OR "mindful*"[Title/Abstract] OR "accept*"[Title/Abstract] OR ("symptom*"[Title/Abstract] AND "intolera*"[Title/Abstract]) OR "secondary gain"[Title/Abstract] OR "agreeable*"[Title/Abstract] OR "conscientious*"[Title/Abstract] OR "extraver*"[Title/Abstract] OR "sociabl*"[Title/Abstract] OR "neurotic*"[Title/Abstract] OR "openness"[Title/Abstract] OR "complain*"[Title/Abstract] OR "moan"[Title/Abstract] OR "attachment"[Title/Abstract] "communication behavior"[Title/Abstract] OR "communication behaviour"[Title/Abstract] OR "doubt"[Title/Abstract] OR "scepti*"[Title/Abstract] OR "distrust"[Title/Abstract] OR "injustice"[Title/Abstract] OR "interpersonal"[Title/Abstract] OR "relation*"[Title/Abstract] OR ("confidence"[Title/Abstract] NOT "confidence interval*"[Title/Abstract]) OR "stigma*"[Title/Abstract] OR "discriminat*"[Title/Abstract] OR "reject*"[Title/Abstract] OR "perfectionis*"[Title/Abstract] OR "absorption"[Title/Abstract] OR "absorbing"[Title/Abstract] OR "suggestib*"[Title/Abstract] OR (("negative"[Title/Abstract] OR "advers*"[Title/Abstract] OR "stressful"[Title/Abstract]) AND "event*"[Title/Abstract]) OR (("early"[Title/Abstract] OR "childhood"[Title/Abstract]) AND ("advers*"[Title/Abstract] OR "negative"[Title/Abstract] OR "stress*"[Title/Abstract])) OR "parent-child relationship"[Title/Abstract] OR "child-parent relationship"[Title/Abstract] OR "emotional neglect*"[Title/Abstract] OR "isolat*"[Title/Abstract] OR "trauma*"[Title/Abstract] OR ("trauma*"[Title/Abstract] AND ("early"[Title/Abstract] OR "childhood"[Title/Abstract])) OR ("adverse"[Title/Abstract] AND ("childhood"[Title/Abstract] OR "early"[Title/Abstract])) OR ("sexual*"[Title/Abstract] AND ("child*"[Title/Abstract] OR "early"[Title/Abstract]) AND ("abuse"[Title/Abstract] OR "violence"[Title/Abstract])) OR ("parent*"[Title/Abstract] AND "illness"[Title/Abstract]) OR ("family history"[Title/Abstract] AND "illness"[Title/Abstract]) OR (("previous"[Title/Abstract] OR "past"[Title/Abstract] OR "history"[Title/Abstract] OR "earlier"[Title/Abstract] OR "repeat*"[Title/Abstract] OR "experience"[Title/Abstract]) AND ("illness"[Title/Abstract] OR "disease"[Title/Abstract])) OR "role model"[Title/Abstract] OR "role-model"[Title/Abstract] OR "sick role"[Title/Abstract] OR ("symptom*"[Title/Abstract] AND "reinforc*"[Title/Abstract] AND ("relative*"[Title/Abstract] OR "family"[Title/Abstract])) OR (("dissatisfaction"[Title/Abstract] OR "disapprov*"[Title/Abstract] OR "frustrat*"[Title/Abstract] OR "unhapp*"[Title/Abstract]) AND ("care"[Title/Abstract] OR "treatment"[Title/Abstract])) OR (("previous"[Title/Abstract] OR "early"[Title/Abstract] OR "experience"[Title/Abstract]) AND ("treatment"[Title/Abstract] OR "therapy"[Title/Abstract])) OR "anxiety"[Title/Abstract] OR "anxious"[Title/Abstract] OR "fear"[Title/Abstract] OR "worr*"[Title/Abstract] OR "panic"[Title/Abstract] OR "comorbid*"[Title/Abstract] OR "depress*"[Title/Abstract] OR "OCD"[Title/Abstract] OR "obsessive-compulsive behavior"[Title/Abstract] OR "obsessive-compulsive-behavior"[Title/Abstract] OR "obsessive-compulsive behaviour"[Title/Abstract] OR "obsessive-compulsive-behaviour"[Title/Abstract] OR "obsessive-compulsive disorder"[Title/Abstract] OR "obsessive-compulsive-disorder"[Title/Abstract] OR "PTSD"[Title/Abstract] OR "posttraumatic stress disorder"[Title/Abstract] OR "post-traumatic stress disorder"[Title/Abstract] OR "substance abuse"[Title/Abstract] OR "drug use"[Title/Abstract] OR "addict*"[Title/Abstract] OR "suicid*"[Title/Abstract] OR "hyperarousal"[Title/Abstract] OR "hyper-arousal"[Title/Abstract] OR "interoception"[Title/Abstract] OR "interoceptive"[Title/Abstract] OR "proprioception"[Title/Abstract] OR "kinesthesia"[Title/Abstract] OR "sensitisation"[Title/Abstract] OR "sensitization"[Title/Abstract] OR "sensitize"[Title/Abstract] OR "sensitise"[Title/Abstract] OR "emotion arous*"[Title/Abstract] OR "emotion activat*"[Title/Abstract] OR "affective arousal"[Title/Abstract] OR ("stress*"[Title/Abstract] AND ("chronic"[Title/Abstract] OR "constant"[Title/Abstract] OR "continuous"[Title/Abstract] OR "persist*"[Title/Abstract])) OR ("intolerance"[Title/Abstract] AND "stress"[Title/Abstract]) OR "stress-intolerance"[Title/Abstract] OR "psychological distress"[Title/Abstract] OR "symptom perception"[Title/Abstract] OR "perceptual processing"[Title/Abstract] OR ("pain"[Title/Abstract] AND "perception"[Title/Abstract]) OR ("pain"[Title/Abstract] AND "threshold"[Title/Abstract]) OR "sensitiv*"[Title/Abstract] OR "tactile sensitiv*"[Title/Abstract] OR "autonomic sensation*"[Title/Abstract] OR ("amplif*"[Title/Abstract] AND "symptom*"[Title/Abstract] AND ("perceive"[Title/Abstract] OR "perception"[Title/Abstract])) OR "somatic amplification"[Title/Abstract] OR "somatosensory amplification"[Title/Abstract]) |
| PsycINFO (via OvidSP) and Cochrane Database of Systematic Reviews (via Cochrane Library) | ("Long COVID" OR "Long-COVID" OR "Post COVID-19 condition" OR "Post-COVID-19 condition" OR "Post COVID conditions" OR "Post-COVID conditions" OR "Post COVID syndrome" OR "Post-COVID syndrome" OR "Post COVID-19 syndrome" OR "Post-COVID-19 syndrome" OR "Post-acute COVID-19 syndrome" OR "Post-acute COVID syndrome" OR "Post-acute sequelae of SARS-CoV-2 infection" OR "Post-acute sequelae of COVID-19" OR "Chronic COVID" OR "Chronic-COVID" OR "Chronic COVID syndrome" OR "Chronic-COVID syndrome" OR "Long-haul COVID" OR "Long-haul COVID-19" OR "Persistent COVID-19") AND ("alexithymia" OR ("difficulty" AND ("feeling*" OR "emotion*")) OR "emotion expression" OR ("emotion" AND ("regulation" OR "control")) OR "psychological conflict" OR ("supression*" AND "emotion*") OR "health anxiety" OR "hypochondri*" OR "health worr*" OR ("health" AND ("concern" OR "concerns")) OR "illness anxiety" OR "illness worr*" OR "illness concern*" OR "loneliness" OR "lonely" OR "isolat*" OR "negative affect*" OR "positive affect*" OR ("avoidance" AND "activit*") OR ("avoiding" AND "activit*") OR ("avoid" AND "activit*") OR "fear-avoidance" OR "fear avoidance" OR "fear of movement" OR "kinesiophobi*" OR "physical activit*" OR "exercis*" OR "physical exertion" OR "workout" OR "work-out" OR "physical effort" OR "physical performance" OR "reduced activity" OR "inactivity" OR "social withdrawal" OR "lack social relationship*" OR "lacking social relationship*" OR "lacks social relationship*" OR ("lack" AND "interpersonal relation*") OR "body check*" OR "body-check*" OR "checking behavior*" OR "checking behaviour*" OR "self observation*" OR "self-observation*" OR "body observation*" OR "body scan*" OR "body-scan*" OR "scanning behavior*" OR "scanning behaviour*" OR "self-inspection" OR "self inspection" OR "illness behaviour" OR "help seeking" OR "help-seeking" OR "health seeking" OR "health-seeking" OR "seek medical help" OR "seeking medical help" OR "seeks medical help" OR "seeks medical attention" OR "seeking medical attention" OR "seek medical attention" OR "requesting diagnosis reevaluation" OR "requesting diagnoses reevaluation" OR "requesting diagnostic reevaluation" OR "medical reassessment" OR "reassurance" OR "medical certainty" OR "seek reassurance" OR "reassurance-seeking" OR "seeking reassurance" OR "verify diagnos*" OR "verified diagnos*" OR " verifying diagnos*" OR "valid diagnos*" OR "validate diagnos*" OR "validity diagnos*" OR "confirm diagnos*" OR "time symptom*" OR "time complaint*" OR "energy complaint*" OR "energy symptom*" OR "health-care utilization" OR "health care utilization" OR "health-care use" OR "healthcare use" OR "medication" OR "doctor-hopping" OR "doctor hopping" OR "repeated doctor visits" OR "repeated medical visits" OR "media use" OR "news use" OR "social media" OR ("information" AND ("search*" OR "seek*")) OR "cyberchondria" OR "self monitor*" OR "self-monitor*" OR "attention" OR "attention* bias*" OR "cognitive bias*" OR "distraction" OR "disturbance" OR "interference" OR "interruption" OR "attention" OR "sensory filtering" OR "stimulus filtering" OR "sensory gating" OR "sensory processing" OR "peripheral filtering" OR "central filtering" OR "body vigilance" OR "internal cues" OR "bod* sensation" OR "*vigilan*" OR "selective recall" OR "memory bias*" OR "locus of control" OR "bodily weakness" OR ("trust" AND "body") OR "catastrophi*" OR ("overestimat*" AND "negative consequences") OR "attribution*" OR ("causal" AND "attribution*") OR ("attribution*" AND ("organic" OR "somatic")) OR (("cause" OR "causation") AND ("biomedical" OR "organic")) OR ("somatic" AND "illness belief") OR ("expect*" AND "side effect*") OR ("expect*" AND "side-effect*") OR "nocebo" OR "optimism" OR "positive thinking" OR "hope" OR "self-efficacy" OR ("treatment" AND "expectation*") OR "expect*" OR "anticipat*" OR ("symptom*" AND "expectation") OR ("health" AND ("worr*" OR "concern")) OR "hypochondri*" OR ("illness" AND ("worr*" OR "concern*")) OR "demoralisation" OR "demoralization" OR "desperation" OR "despair" OR "loss of hope" OR "hopeless*" OR "helpless*" OR "illness belief*" OR "symptom belief*" OR "symptom perception" OR "illness perception" OR "illness representation" OR "symptom representation" OR "mindful*" OR "accept*" OR ("symptom*" AND "intolera*") OR "secondary gain" OR "agreeable*" OR "conscientious*" OR "extraver*" OR "sociabl*" OR "neurotic*" OR "openness" OR "complain*" OR "moan" OR "attachment" "communication behavior" OR "communication behaviour" OR "doubt" OR "scepti*" OR "distrust" OR "injustice" OR "interpersonal" OR "relation*" OR ("confidence" NOT "confidence interval*") OR "stigma*" OR "discriminat*" OR "reject*" OR "perfectionis*" OR "absorption" OR "absorbing" OR "suggestib*" OR (("negative" OR "advers*" OR "stressful") AND "event*") OR (("early" OR "childhood") AND ("advers*" OR "negative" OR "stress*")) OR "parent-child relationship" OR "child-parent relationship" OR "emotional neglect*" OR "isolat*" OR "trauma*" OR ("trauma*" AND ("early" OR "childhood")) OR ("adverse" AND ("childhood" OR "early")) OR ("sexual*" AND ("child*" OR "early") AND ("abuse" OR "violence")) OR ("parent*" AND "illness") OR ("family history" AND "illness") OR (("previous" OR "past" OR "history" OR "earlier" OR "repeat*" OR "experience") AND ("illness" OR "disease")) OR "role model" OR "role-model" OR "sick role" OR ("symptom*" AND "reinforc*" AND ("relative*" OR "family")) OR (("dissatisfaction" OR "disapprov*" OR "frustrat*" OR "unhapp*") AND ("care" OR "treatment")) OR (("previous" OR "early" OR "experience") AND ("treatment" OR "therapy")) OR "anxiety" OR "anxious" OR "fear" OR "worr*" OR "panic" OR "comorbid*" OR "depress*" OR "OCD" OR "obsessive-compulsive behavior" OR "obsessive-compulsive-behavior" OR "obsessive-compulsive behaviour" OR "obsessive-compulsive-behaviour" OR "obsessive-compulsive disorder" OR "obsessive-compulsive-disorder" OR "PTSD" OR "posttraumatic stress disorder" OR "post-traumatic stress disorder" OR "substance abuse" OR "drug use" OR "addict*" OR "suicid*" OR "hyperarousal" OR "hyper-arousal" OR "interoception" OR "interoceptive" OR "proprioception" OR "kinesthesia" OR "sensitisation" OR "sensitization" OR "sensitize" OR "sensitise" OR "emotion arous*" OR "emotion activat*" OR "affective arousal" OR ("stress*" AND ("chronic" OR "constant" OR "continuous" OR "persist*")) OR ("intolerance" AND "stress") OR "stress-intolerance" OR "psychological distress" OR "symptom perception" OR "perceptual processing" OR ("pain" AND "perception") OR ("pain" AND "threshold") OR "sensitiv*" OR "tactile sensitiv*" OR "autonomic sensation*" OR ("amplif*" AND "symptom*" AND ("perceive" OR "perception")) OR "somatic amplification" OR "somatosensory amplification") |

^1-113^

## **eAppendix 2. Quality rating (see Excel file)**

## **eTable 1. Descriptive information for included studies**

| **Author (year of publication)** | **Type and aim of the study** | **Sample characteristics (sample size; population; setting; age; sex (female))** | **Diagnostic criteria of Long COVID** | **Psychological variable(s) (measuring instrument)** | **Key results** | **Research question(s)** |
| --- | --- | --- | --- | --- | --- | --- |
| Al-Hadrawi et al. (2023) | Cross-sectional study aiming to delineate the effects of oxygen saturation (SpO2) and body temperature during the acute phase on Long COVID symptoms and providing data on the comparison of Long COVID patients and healthy controls along clinical and psychological characteristics | *n* = 120 patients with Long COVID and *n* = 36 healthy controls without a history of COVID-19; patients formerly hospitalised in Iraq;  *M*(age Long COVID) = 31.27, *SD* = 8.36; 29% female  *M*(age no Long COVID) = 30.9, *SD* = 8.3; 17% female | According to the WHO definition | Anxiety symptoms (HAMA)  Depressive symptoms (HAMD) | Individuals with Long COVID showed higher rates of depression and anxiety than healthy controls | RQ1 |
| Alghamdi et al. (2022) | Retrospective study aiming to assess the incidence, risk factors and most common persisting symptoms of Long COVID | *n* = 277 patients with Long COVID and *n* = 227 patients after COVID-19 without Long COVID; all adult patients formerly hospitalised for confirmed COVID-19 in a tertiary-care hospital in Saudi Arabia;  Age groups(Long COVID)  18-30 y: 14.1%  > 30-50 y: 46.3%  > 50-70 y: 33.0%  > 70 y: 6.6%; 56.8% female  Age groups(no Long COVID)  18-30 y: 16.6%  > 30-50 y: 49.8%  > 50-70 y: 28.2%  > 70 y: 5.4%; 48.5% female | Presence of one or more symptoms beyond 12 weeks from the onset of the illness | Depressive symptoms (yes/no)  Anxiety symptoms (yes/no) | Individuals with depressive and anxiety symptoms were significantly more likely to suffer from Long COVID than controls | RQ1 |
| Alkwai et al. (2022) | Case-control study aiming to describe and characterise the prevalence of persistent COVID-19 symptoms and to evaluate risk factors for the delayed return to the usual state of health | *n* = 108 patients with Long COVID and *n* = 105 patients after COVID-19 without Long COVID; users of social media platforms in Saudi Arabia;  Age groups(Long COVID)  18-24 y: 25.9%  25-34 y: 37.0%  35-44 y: 24.1%  45-54 y: 8.3%  55-64 y: 4.6%  65+ y: 0.0%; 78.7% female  Age groups(no Long COVID)  18-24 y: 34.3%  25-34 y: 40.0%  35-44 y: 19.0%  45-54 y: 4.8%  55-64 y: 1.0%  65+ y: 1.0%; 73.3% female | According to the NICE guideline (> 12 weeks) | Anxiety (as a chronic health condition; self-report via one item)  Depression (as a chronic health condition; self-report via one item)  Other psychological illness (as a chronic health condition; self-report via one item) | There was no significant difference between patients with Long COVID and controls in terms of anxiety, depression, and other psychological illnesses | RQ1 |
| Antony et al. (2023) | Predictive study using electronic health record data to predict the incidence of Long COVID with trained machine learning models | *n* = 2,190,579 patients after COVID-19, including *n* = 17,036 patients with Long COVID and n = 2,173,543 patients after COVID-19 without Long COVID; adult sample from the National COVID Cohort Collaborative in the US;  Age groups(whole sample)  ≤ 10 y: 8.45%  11-20 y: 10.81%  21-30 y: 15.85%  31-40 y: 16.02%  41-50 y: 14.44%  51-60 y: 14.22%  61-70 y: 11.02%  71-80 y: 6.22%  81-90 y: 2.03%  ≥ 91 y: 0.00%; 55.82% female | Post-COVID-19 condition diagnosis (ICD-10: U09.9) | Pre-existing depression (diagnosis obtained from electronic health records) | When predicting the future occurrence of Long COVID with machine learning models trained using information from the electronic health records of COVID-19 patients during the acute infection (logistic regression and random forest models), a significant contribution of depression to the development of Long COVID was found | RQ3 |
| Ariza et al. (2022) | Cross-sectional study aiming to describe cognitive dysfunction in Long COVID and providing data on the comparison of Long COVID patients and HC along psychological variables | *n* = 319 patients with Long COVID and *n* = 109 healthy controls without a history of COVID-19; participants evaluated in 16 hospitals in Catalonia, Madrid and Andorra;  *M*(age Long COVID) = 49.1, *SD* = 9.12; 77.7% female  *M*(age no Long COVID) = 46.1, *SD* = 9.31; 62.4% female | Signs and symptoms of the acute disease at least 12 weeks after infection | Depression (PHQ-9)  Anxiety (GAD-7) | Depression and anxiety explained part of the sample’s variance in cognitive performance, as evidenced by the reduction of cognitive differences between the Long COVID and controls after controlling for these factors | RQ1 |
| Azcue et al. (2022) | Case-control study aiming to describe and compare the cognitive impairment and neuropsychiatric symptoms in Long COVID and ME/CFS along clinical and psychological characteristics | *n* = 73 patients with Long COVID and *n* = 42 patients with ME/CFS; patients recruited in the Neurology department at a University Hospital in Spain;  *M*(age Long COVID) = 44.36, *SD* = 9.47; 69.9% female  *M*(age ME/CFS) = 43.5, *SD* = 8.24; 92.9% female | Criteria proposed by the NICE guidelines; symptoms had to be present at least 3 months after the infection and persisting for at least 2 months | Depression (GDS)  Trait-Anxiety (STAI-T)  State-Anxiety (STAI-S)  Suicidal ideation (C-SSRS) | Individuals with Long COVID and ME/CFS shared common symptomatology and cognitive patterns with greater impairment in ME/CFS; significant negative correlations between the psychological variables depression, anxiety, and suicidal ideation with several quality of life indices and positive correlations with fatigue were found | RQ1; RQ2 |
| Becker et al. (2021) | Prospective study investigating long-term outcomes regarding residual symptoms and psychological distress in hospitalised patients 1 year after COVID-19 | *n* = 63 patients with Long COVID and *n* = 27 patients after COVID-19 without Long COVID; all adult patients hospitalised for confirmed COVID-19 in two Swiss tertiary-care hospitals;  *M*(age Long COVID) = 59.41, *SD* = 14.3; 33% female  *M*(age no Long COVID) = 61.7, *SD* = 14.3; 48% female | Presence of at least one persisting symptom assessed 12 months after the initial infection with SARS-CoV-2 which newly  occurred during or after the infection | Anxiety symptoms (HADS-A)  Depressive symptoms (HADS-D)  PTSD symptoms 30 (IES-R)  Perceived stress during illness (PSS)  Resilience (CD-RISC) | There were no significant differences regarding psychological variables assessed at 30-day follow-up and the prevalence of Long COVID at 1 year between the Long COVID group and the control group | RQ1 |
| Bellan et al. (2022) | Prospective study aiming to identify risk factors for the development of Long COVID in a cohort of subjects hospitalised for SARS-CoV-2 infection | *n* = 93 patients with Long COVID and *n* = 154 patients after COVID-19 without Long COVID; patients formerly hospitalised for COVID-19 in a University hospital in Novara, Italy;  $\tilde{X}$(age Long COVID) = 59  (IQR 51-68); 31% female  $\tilde{X}$(age no Long COVID) = 61 (IQR 50-70); 52% female | Presence of at least one residual symptom assessed 12 months after hospital discharge due to confirmed COVID-19 | Anxiety symptoms (yes/no; MINI)  Depressive symptoms (yes/no; MINI) | Anxiety and depression symptoms at 1YFU were more frequent in patients with Long COVID than in controls at the same time | RQ1 |
| Benoit-Piau et al. (2023) | Multicentre population-based cohort study aiming to assess acute COVID-19 symptoms and their evolution for up to 9 months following the infection and providing data on the comparison between Long COVID patients and controls | *n* = 1,349 patients after COVID-19, including *n* = 622 patients with Long COVID and *n* = 727 patients after COVID-19 without Long COVID; adult population-based sample recruited in three Canadian regions;  *M*(age whole sample) = 46.7, *SD* = 16.1; 58.1% female | Reporting one  symptom or more at least 3 months after COVID-19 | Pre-existing mental health disorders (self-report yes/no)  Psychological  distress (K6), cut-off score ≥ 14 | Patients with Long COVID showed a higher prevalence of mental health conditions and reported higher psychological distress than controls; having a diagnosis of mental health disorder was significantly associated with Long COVID | RQ1; RQ2 |
| Beyer et al. (2023) | Cross-sectional study aiming to investigate exercise capacity as well as markers of subjective wellbeing and their independent relation to post-COVID-19 syndrome | *n* = 69 patients with Long COVID; adult patients recruited at the pneumological post-COVID-19 outpatient clinic of a German university medical hospital;  *M*(age Long COVID) = 46, *SD* = 12; 66.6% female | 3-month continuing impairment of capability after COVID-19 with (FAS) score ≥ 22 points | Depressive symptoms (HADS)  Anxiety symptoms (HADS) | The study showed a significant correlation of Depression and Anxiety with fatigue | RQ2 |
| Bierbauer et al. (2022) | Cross-sectional study assessing psychological factors and their relationship with health-related outcomes in Long COVID patients | *n* = 246 patients with Long COVID recruited via online platforms in Switzerland;    *M*(age Long COVID) = 45.29, *SD* = 12.12; 89% female | Experiencing persistent symptoms ≥ 12 weeks past SARS-CoV-2 infection | Personal control (IPQ-R)  Treatment control (IPQ-R)  Coherence (IPQ-R)  Emotional representation (IPQ-R)  Illness identity (IPQ-R)  Depressive symptoms (HADS)  Anxiety (HADS) | Significant correlations between aspects of illness identity, depression, and anxiety with quality of life and fatigue were found | RQ2 |
| Binka et al. (2022) | Cross-sectional study aiming to develop an algorithm to identify individuals with Long COVID using population-level health administrative data | *n* = 2,430 patients with Long COVID and *n* = 24,300 patients without Long COVID from the BC COVID-19 Cohort in Canada;  Age groups(Long COVID)  18-29 y: 8.8%  30-39 y: 14.9%  40-49 y: 21.8%  50-59 y: 23.2%  60-69 y: 17.9%  70-79 y: 9.6%  80+ y: 3.8%; 53.9% female  Age groups(no Long COVID)  18-29 y: 32.3%  30-39 y: 23.2%  40-49 y: 17.7%  50-59 y: 13.1%  60-69 y: 8.0%  70-79 y: 3.7%  80+ y: 1.9%; 48.4% female | Either ICD-10 code U09.9 or diagnosis in a Post-COVID-19 Recovery Clinic; in the latter case criteria were persistent symptoms for at least 12 weeks after acute symptom onset and ≥ 1 abnormal patient-reported outcome measure score (cough, dyspnoea, fatigue, anxiety, depression, PTSD) | Depressive disorders (diagnosis) | Depressive disorders were identified as a relevant predictor of a Long COVID diagnosis | RQ3 |
| Bonner et al. (2024) | Cross-sectional study aiming to examine relationships between Long COVID, chronic conditions, and work outcomes | *n* = 18,816 participants, including 17.8% (*n* = 3,349) with Long COVID and *n* = 15,467 healthy controls without a history of COVID-19; working aged adult sample from the US National Health Interview Survey;  Age groups(whole sample)  18-24 y: 2,783 (14.8%)  25-34 y: 4,186 (22.3%)  35-44 y: 4,069 (21.6%)  45-54 y: 3,791 (20.2%)  55-64 y: 3,988 (21.2%); 49.5% female | Long-term symptoms lasting 3 months or longer that emerged after having COVID-19 | Anxiety disorder or Depression (self-report yes/no) | The study showed a significant association of Anxiety/Depression with Long COVID and disability | RQ2 |
| Bottemanne et al. (2021) | Prospective study investigating risk factors for persistent physical symptoms following COVID-19. | *n* = 84 patients after COVID-19; patients previously hospitalised for COVID-19 in a hospital in France;  $\tilde{X}$(age all patients) = 60 (IQR 50.5-67.5) | Presence of fatigue, dyspnoea or pain complaints assessed 3 months after hospital discharge | Depression (HADS-D)  Anxiety (HADS-A) | Anxiety and Depression at 1-month FU did not show to be significant predictors of fatigue, dyspnoea and pain complaints at 3-month FU | RQ3 |
| Bungenberg et al. (2022) | Cross sectional study aiming to objectify and compare persisting self-reported symptoms in hospitalised and non-hospitalised patients after COVID-19 and providing data on the association between psychological variables and Long COVID symptoms | *n* = 50 patients with Long COVID from Germany;  $\tilde{X}$(age Long COVID) = 50.5 (IQR 41-61) | Participants with persisting symptoms for at last 4 weeks after SARS-CoV-2 infection were included | Affective Symptoms (HADS) | The severity of affective symptoms was positively associated with fatigue severity | RQ2 |
| Buonsenso et al. (2023) | Cross-sectional study aiming to investigate if children with Long COVID experience stigma | *n* = 224 patients after COVID-19, including *n* = 40 patients with Long COVID; paediatric sample evaluated at 3MFU at a paediatric post-COVID clinic in Rome, Italy;  $\tilde{X}$(age Long COVID) = 114.0 months (IQR 90.0-156.0); 57.5% female  $\tilde{X}$(age no Long COVID) = 72.0 months (IQR 36.0-108.0); 43.7% female | According to the WHO definition of paediatric Long COVID | Stigma (adapted LCSS survey) | Children with Long COVID significantly more frequently felt stigmatised than controls | RQ1 |
| Burton et al. (2023) | Longitudinal study (ecological momentary assessment) aiming to describe the real-time associations between different symptoms and between symptoms and physical activity at the individual patient level | *n* = 74 patients with Long COVID; patients from the UK were recruited via the RICOVER database (Sheffield Hallam University) for people living with Long COVID as well as via a Facebook self-help-group;  $\tilde{X}$(age Long COVID) = 50 (IQR 42-54); 85.1% female | Presence of ongoing physical symptoms which the individual attributed to Long COVID and which followed (by at least 3 months) a recognizable acute infection during the COVID-19 pandemic (positive test result was not mandatory) | Anxiousness (VAS)  Physical activity (measured with accelerometer) | Pooled within-subject correlations showed anxiety correlated with cognitive difficulty, but not with fatigue | RQ2 |
| Busatto et al. (2022) | Cohort study aiming to investigate the patterns of co-occurrence of multiple symptoms in Long COVID | *n* = 749 patients with Long COVID; patients formerly admitted to a hospital due to COVID-19 in Sao-Paulo, Brazil;  *M*(age Long COVID) = 55, *SD* = 14; 47% female | Persistent symptoms reported at follow-up (median 212 days after hospitalisation) | PTSD (PCL), cut-off score ≥ 45  Anxiety (HADS-A), cut-off score ≥ 8  Depression (HADS-D), cut-off score ≥ 8 | Different symptoms that persist for several months after moderate or severe COVID-19 were dominated by fatigue and psychiatric symptoms | RQ2 |
| Cai et al. (2023) | Longitudinal cohort study aiming to study overall short- and long-term influences of Long COVID among a large population in Asia and providing data on mental health differences between patients with Long COVID and controls | 6MFU:  *n* = 450 patients with Long COVID, *n* = 75 patients after COVID-19 without Long COVID, and *n* = 979 healthy controls without a history of COVID-19; patients from a hospital in China;  *M*(age Long COVID) = 42.32, *SD* = 12.67; 43.33% female  *M*(age no Long COVID) = 39.77, *SD* = 14.83; 37.33% female  *M*(age HC) = 43.51, *SD* = 10.70; 36.26% female  12MFU:  *n* = 93 patients with Long COVID | Conditions that occur in individuals with a history of probable or confirmed SARS-CoV-2 infection 3 months from the onset of COVID-19 that last for 2 months and cannot be explained by an alternative diagnosis | Anxiety (GAD-7), cut-off score ≥ 5  Depression (PHQ-9), cut-off score ≥ 5  PTSD (PCL-C), cut-off score ≥ 38  Anxiety/Depression (EQ-5D-5L) | Patients with Long COVID reported a higher proportion of abnormal mental status than controls | RQ1 |
| Calvache-Mateo et al. (2023) | Case-control study aiming to identify the clinical and psychosocial profile associated with pain in non-hospitalised patients with Long COVID | *n* = 55 patients with Long COVID, *n* = 57 patients after COVID-19 without Long COVID and *n* = 58 healthy controls without a history of COVID-19; Long COVID patients were recruited from the "Covid Persistente Andalucía" association and HC by word-of-mouth in Spain;  *M*(age Long COVID) = 45.51, *SD* = 3.28; 70.9% female  *M*(age no Long COVID) = 44.74, *SD* = 3.04; 66.7% female  *M*(age HC) = 45.43, *SD* = 3.63; 69% female | According to the WHO definition | Physical activity (IPAQ-SF)  Central sensitisation (CSI)  Kinesiophobia (TSK)  Catastrophising (PCS)  Depression (DASS-21)  Anxiety (DASS-21)  Stress (DASS-21)  Fear avoidance (FABQ) | The Long COVID group obtained significantly worse punctuation in psychosocial variables than the other two groups | RQ1 |
| Carazo et al. (2022) | Cross-sectional study aiming to assess the prevalence of Long COVID and associated physical, psychological, and cognitive symptoms | *n* = 2,836 patients with Long COVID and *n* = 4,390 healthy controls without a history of COVID-19; participants recruited from the province of Quebec, Canada;  Age groups(Long COVID)  18-29 y: 540  30-39 y: 686  40-49 y: 835  50-59 y: 601  60-80 y: 174; 82.79% female  (no data for HC) | Persistent symptoms 4 weeks after an acute SARS-CoV-2 infection | Psychological distress (K6), cut-off score: 7-12 high distress, > 12 very high distress | Psychological distress was similarly reported as high or very high by Long COVID cases as by cases without Long COVID | RQ1 |
| Carter et al. (2022) | Case-control study aiming to investigate effects of Long COVID on functional status, mood state and physical activity | *n* = 17 patients with Long COVID and *n* = 15 matched healthy controls without a history of COVID-19; participants from the US recruited via social media, radio advertisement and the Indiana Clinical and Translational Institute’s web-portal;  *M*(age Long COVID) = 55, *SD* = 11  *M*(age no Long COVID) = 54, *SD* = 14; 100% female | One or more symptoms beyond 3-4 weeks from initial illness onset (SARS-CoV-2 infection); one patient reported “loss of smell” lasting 20 days and was included in the analysis | Anger (POMS)  Depression (POMS)  TMD (adding the  negative mood variables and subtracting the positive variable of vigour)  Physical activity (GSLTPAQ)  Depression medications  Walking self-efficacy (8-item scale) | Significant between-group differences were detected for TMD revealing elevated values among Long COVID patients and Long COVID patients exhibited reduced physical activity compared to controls | RQ1 |
| Chen et al. (2023a) | Single-centre retrospective study aiming to explore the quality of life in children with Long COVID and how pre-existing conditions affect symptoms and quality of life | *n* = 93 patients with Long COVID; paediatric sample (< 21 y) treated at a Paediatric Post-COVID-19 Rehabilitation Clinic in the US;  *M*(age Long COVID) = 13.7, *SD* = 4.3; 63% female | Clinical diagnosis of Long COVID using the Health and Human Services definition of signs, symptoms, and conditions that continue or develop after initial COVID-19 or SARS-CoV-2 infection and are present four weeks or more after the initial phase of infection | Pre-existing history of Depression (diagnosis obtained from electronic medical records)  Pre-existing history of Anxiety (diagnosis obtained from electronic medical records)  Pre-existing history of OCD (diagnosis obtained from electronic medical records)  Pre-existing history of ADHD (diagnosis obtained from electronic medical records) | Pre-existing mood disorders were significantly associated with change in appetite and dizziness/light-headedness | RQ3 |
| Chen et al. (2023b) | Cross-sectional study aiming to investigate whether COVID-19 patients with pre-pandemic insomnia have a greater risk of developing Long COVID and providing data on differences in mental health between patients with and without Long COVID | *n* = 1,403 patients after COVID-19, including *n* = 904 patients with Long COVID and *n* = 499 patients after COVID-19 without Long COVID; participants of an international collaborative study from 16 countries (Austria, Brazil, Bulgaria, Canada, China, Croatia, Finland, France, Germany, Israel, Italy, Japan, Norway, Portugal, Sweden, and the United States);  *M*(age Long COVID) = 44.8, *SD* = 16.3; 60.6% female  *M*(age no Long COVID) = 37.4, *SD* = 18.5; 41.1% female | According to the WHO definition | Pre-existing Depression and/or Anxiety (self-reported diagnosis yes/no) | Compared to controls, Long COVID cases were significantly more likely to have a diagnosis of Depression and/or Anxiety before the pandemic | RQ1 |
| Cuschieri et al. (2023) | Observational, cross-sectional study exploring Long COVID characteristics and associated mental health impact | *n* = 50 patients with Long COVID, *n* = 72 patients after COVID-19 without Long COVID and *n* = 489 healthy controls without a history of COVID-19; participants from Malta recruited via an anonymous online survey disseminated through social media;  Age groups(Long COVID)  18-19 y: 0%  20-29 y: 10%  30-39 y: 62%  40-49 y: 16%  50-59 y: 10%  60-69 y: 2%  70-79 y: 0%; 82% female  Age groups(recovered)  18-19 y: 1.4%  20-29 y: 25%  30-39 y: 33.3%  40-49 y: 23.6%  50-59 y: 6.9%  60-69 y: 8.3%  70-79 y: 1.4%; 63.9% female | Persistent symptoms post-acute infection that  lasted beyond two months | Anxiety (GAD-7), cut-off scores: 0-4 no symptoms, 5-9 mild symptoms, 10-14 moderate symptoms > 15 severe symptoms  Depression (PHQ-9), cut-off scores: 0-4 mild depression, 10-14 moderate depression, 15-19 moderate severe depression, 20-27 severe depression | Patients with Long COVID had a significantly higher anxiety score than those never acquiring COVID-19 and the Long COVID cohort had significantly higher moderate and severe depression scores when compared to both those acquiring COVID-19 with no persistent symptoms and those never acquiring COVID-19 | RQ1 |
| de Oliviera et al. (2022) | Observational, cross-sectional study aiming to investigate Long COVID in a cohort of hospitalised patients in Brazil | *n* = 369 patients with Long COVID and *n* = 70 healthy controls without a history of COVID-19; participants from Brazil recruited after discharge from a public hospital in Belo Horizonte;  $\tilde{X}$(age Long COVID) = 57 (IQR 46-66); 51% female  $\tilde{X}$(age no Long COVID) = 62 (IQR 49-71) | Persistence of at least one physical and/or mental health symptom 4 or more weeks after disease onset | Depression and anxiety (questionnaire on Long COVID symptoms)  Depression and anxiety (EQ-5D-3L) | Patients with Long COVID showed higher levels of depression/anxiety scores than controls | RQ1 |
| Delgado-Alonso et al. (2022a) | Cross-sectional study aiming to evaluate personality traits in patients with Long COVID as well as the association with neuropsychiatric symptoms | *n* = 93 patients with Long COVID and *n* = 93 matched healthy controls without a history of COVID-19; patients recruited from the neurology department of a hospital in Madrid, Spain;  *M*(age Long COVID) = 50.39, *SD* = 11.26; 71% female  *M*(age no Long COVID) = 50.28, *SD* = 13.50; 71% female | According to the WHO definition | Depression (BDI-II)  Trait-Anxiety (STAI-T)  State-Anxiety (STAI-S)  Personality (emotional stability, extraversion, openness, conscientiousness, agreeableness; BFSI) | The study showed higher levels of neuroticism (= lower emotional stability), lower agreeableness and lower extraversion in patients with Long COVID compared to controls; no statistically significant correlations were identified between the five personality factors and fatigue | RQ1; RQ2 |
| Delgado-Alonso et al. (2022b) | Cross-sectional study aiming to determine the characteristics of cognitive dysfunction in patients reporting cognitive complaints after COVID-19 and to evaluate the correlation between cognitive function and anxiety, depression, sleep, and olfactory function | *n* = 50 patients with Long COVID and *n* = 50 matched healthy controls without a history of COVID-19; adult sample from Spain recruited from patients consulting due to cognitive issues after COVID-19;  *M*(age Long COVID) = 51.06, *SD* = 11.65; 74% female | Cognitive complaints at least three months after the onset of SARS-CoV-2 infection | Trait-Anxiety (STAI-T)  State-Anxiety (STAI-S)  Depression (BDI-II) | Anxiety, but not depression, significantly correlated with the global cognitive composite score | RQ2 |
| Delgado-Alonso et al. (2023) | Cross-sectional study aiming to examine the characteristics of brain fog and to understand how fatigue, cognitive performance, and neuropsychiatric symptoms and the mutual relationships among these variables influence subjective cognitive complaints | *n* = 170 patients with Long COVID cognitive complaints; adult sample from Spain;  *M*(age Long COVID) = 49.37, *SD* = 10.96; 73.1% female | Diagnosis of post-COVID-19 condition according to the WHO definition | Depressive symptoms (BDI-II)  Trait-Anxiety (STAI-T)  State-Anxiety (STAI-S) | Depression, Trait-Anxiety and State-Anxiety significantly correlated with brain fog | RQ2 |
| Durstenfeld et al. (2023) | Online cohort study aiming to determine whether sociodemographic factors, lifestyle, or medical history preceding COVID-19 or characteristics of acute SARS-CoV-2 infection are associated with Long COVID | *n* = 476 patients with Long COVID and *n* = 1,004 healthy controls without a history of COVID-19;  participants from the US recruited through email invitations, press releases, word-of-mouth, and by partner organisation;  *M*(age Long COVID) = 53.13, *SD* = 13.27; 75.11% female  *M*(age no Long COVID) = 52.5, *SD* = 14.13; 65.9% female | At least one Long COVID symptom 30 days post infection | Anxiety (GAD-7)  Depression (PHQ-8)  Physical activity (average days/week) | Patients with Long COVID showed significantly higher depression and anxiety scores as well as lower physical activity compared to controls and pre-existing depression was a significant predictor of Long COVID symptoms, while anxiety was not | RQ1; RQ3 |
| Elmunzer et al. (2023) | Follow-up study of patients enrolled in an observational cohort study aiming to examine the long-term gastrointestinal impact of hospitalisation with COVID-19 | *n* = 116 patients after COVID-19, including *n* = 104 patients with Long COVID; patients formerly hospitalised with COVID-19 across the US and Canada;  *M*(age whole sample) = 55.2, *SD* = 14.3; 52.6% female | At least one persistent COVID-19-related symptom over the past month 12–18 months after COVID-19 hospitalisation | PTSD (IES-R), cut-off score ≥ 33  Depressive symptoms (PHQ-9)  Anxiety symptoms (GAD-7) | Higher degree of psychological trauma related to the illness experience, depressive symptoms and anxiety symptoms significantly correlated with the number of persistent COVID-related symptoms and with gastrointestinal symptom severity; a higher psychological trauma score was most strongly associated with the number of moderate or severe persistent COVID-related symptoms and with gastrointestinal symptom severity | RQ2 |
| Fernández-Alonso et al. (2023) | Descriptive cross-sectional study aiming to examine the relationship between resilience and health-related quality of life in patients following COVID-19 among those with and without lingering symptoms | 3MFU:  *n* = 32 patients with Long COVID and *n* = 65 patients after COVID-19 without Long COVID;  6MFU:  *n* = 54 patients with Long COVID and *n* = 43 patients after COVID-19 without Long COVID; patients recruited from a hospital in Spain;  *M*(age whole sample at 3MFU) = 62.67, *SD* = 15.10; 25.8% female | At least one symptom at 3 months/6 months post infection | Depression (HADS-D)  Anxiety (HADS-A) | Patients with Long COVID had higher depression scores at 3MFU and higher anxiety scores at 6MFU than controls | RQ1 |
| Fernández-de-Las-Peñas et al. (2022) | Observational, cross-sectional study aiming to analyse correlations between neuropathic symptoms and pain with proxies of sensitisation, pain-related, or psychological/cognitive variables in COVID-19 survivors exhibiting post-COVID pain | *n* = 146 patients with Long COVID; patients recruited from three urban hospitals in Spain;  *M*(age Long COVID) = 57.5, *SD* = 11.8; 53.4% female | New-onset pain symptoms starting after the infection, lasting for three months (pain as main Long COVID symptom) | Sensitisation (CSI)  Anxiety (HADS-A)  Depression (HADS-D)  Kinesiophobia (TSK-11)  Pain Catastrophising (PCS) | The study revealed significant associations between psychological variables and quality of life (sensitisation, depression and catastrophising) and sensitisation, anxiety and depression were significantly associated with pain intensity | RQ2 |
| Freda et al. (2024) | Cross-sectional study aiming to determine psychosocial differences between children with Long COVID and two control groups from a bio-psycho-social and psychosomatic perspective | *n* = 29 patients with Long COVID, *n* = 76 patients after COVID-19 without Long COVID and *n* = 93 healthy controls without a history of COVID-19; data were collected from 198 Italian mothers of children aged 4 to 13 years recruited at the University Hospital of Naples, Italy;  Children:  *M*(age Long COVID) = 7.8, *SD* = 2.8; 41% female  *M*(age no Long COVID) = 7.7, *SD* = 2.8; 55% female  *M*(age HC) = 8.5, *SD* = 2.8; 47% female  Mothers:  *M*(age Long COVID) = 39.7, *SD* = 4.7  *M*(age no Long COVID) = 40.8, *SD* = 5.0  *M*(age HC) = 43.0, *SD* = 4.8 | At least one symptom lasting at least 12 weeks from SARS-CoV-2 positivity | Maternal health anxiety (HAPYS); subscale “Thoughts”  Child health anxiety (SAI); subscale “Health Anxiety Symptoms”  COVID-19 anxiety (FIVE); subscales “Fear of SARS-CoV-2 Infection and Illness” and “Fears about  Social Distancing”  Child’s mental health (SDQ) | In contrast to child health anxiety, fear of distancing, and adjustment problems, for which no significant differences were found, differences in maternal health anxiety and fear of SARS-CoV-2 infection were statistically significant; maternal health anxiety was significantly higher in mothers of children with Long COVID than in the two control groups and children with Long COVID had significantly lower scores on fear of SARS-CoV-2 infection than healthy controls | RQ1 |
| Frontera et al. (2021) | Cross-sectional study aiming to quantitatively measure self-reported metrics of fatigue, cognitive dysfunction, anxiety, depression, and sleep and identify factors associated with these metrics among United States residents with or without COVID-19 | *n* = 19 patients with Long COVID, *n* = 57 patients after COVID-19 without Long COVID and *n* = 923 healthy controls without a history of COVID-19; participants from the US recruited via an online survey utilising a commercial crowdsourcing community research platform;  $\tilde{X}$(age Long COVID) = 32 (IQR 22-51); 32% female  $\tilde{X}$(age no Long COVID) = 44 (IQR 28-56); 65% female  $\tilde{X}$(age HC) = 45 (IQR 31-60); 48% female | “Prolonged COVID-19” was self-reported among COVID-19 participants who continued to have symptoms ≥ 1 month after initial diagnosis | Anxiety (Neuro-QOL)  Depression (Neuro-QOL)  History of  Depression/Anxiety/Other mood disorder/Thought disorder  Depression as Long COVID symptom  Anxiety as Long COVID symptom | Prolonged COVID-19 participants more often had a history of depression/mood/thought disorder and those with prolonged COVID-19 had significantly worse anxiety scores | RQ1 |
| Fry et al. (2023) | Cross-sectional study aiming to compare psychological functioning in a Long COVID group to a PCS group, a syndrome with a significant psychological component | *n* = 201 patients with Long COVID and *n* = 102 patients with PCS; Long COVID patients recruited at the University of Minnesota and PCS patients recruited from a separate academic medical centre and the University of Minnesota, USA;  *M*(age Long COVID) = 48.87, *SD* = 13.5; 71% female  *M*(age PCS) = 46.08, *SD* = 16.97; 63.7% female | According to the WHO definition; including (1) COVID-19 as confirmed by a positive PCR or antibody test, and (2) duration of symptoms lasting more than 30 days following the onset of COVID-19; all participants had symptoms that persisted at least 6 months following acute COVID-19 | Personality (PAI scales) | There was no significant difference between patients with Long COVID and patients with PCS regarding personality scores except for paranoia which was higher in patients with PCS | RQ1 |
| Fujita et al. (2023) | Retrospective cross-sectional study aiming to elucidate the characteristics of Long COVID patients with headaches and giving cross-sectional data on the association between | *n* = 482 patients with Long COVID; adult patients visiting a Long COVID outpatient clinic who were initially hospitalised for COVID-19 in a university hospital in Japan;  $\tilde{X}$(age 369 Long COVID patients without headache) = 42 (IQR 28-52)  $\tilde{X}$(age 113 Long COVID patients with headache) = 37 (IQR 22-45)  Whole sample: 54.35% female | Long COVID was defined in the present study as symptoms that persist for more than four weeks after the onset of COVID-19 | Anxiety symptoms (yes/no; assessed in medical interviews) | Self-reported anxiety after COVID-19 was not significantly related to quality of life | RQ2 |
| Gaspar et al. (2023) | Prospective cohort study aiming to describe and prospectively assess the prevalence and risk factors for Long COVID three, six and nine months after hospital discharge, and to evaluate its impact on patients’ health-related quality of life | At 9MFU:  *n* = 59 patients with Long COVID and *n* = 51 patients after COVID-19 without Long COVID; adult patients formerly admitted to a COVID-19 dedicated ward at a hospital in Portugal;  $\tilde{X}$(age Long COVID) = 58 (IQR 71-51); 54.2% female  $\tilde{X}$(age no Long COVID) = 59 (IQR 73-48); 37.2% female | Presence of persistent signs and/or symptoms occurring three months after the acute SARS-Cov-2 infection, lasting for at least two months, and not otherwise explained by alternative causes | Pre-existing Depression/Anxiety (use of anti-depressive and/or anxiolytic drugs and/or medical record of diagnosis) | There was no significant difference between patients with and without Long COVID in terms of pre-existing Depression/Anxiety and pre-existing Depression/Anxiety was not associated with the presence of persisting symptoms at any time point | RQ1; RQ3 |
| Giurgi-Oncu et al. (2021) | Cross-sectional study investigating, among others, the impact of mental health impairments on the quality of life in subjects who were hospitalised or treated as outpatients for a mild/moderate SARS-CoV-2 pulmonary infection and are currently suffering from Long COVID | *n* = 143 patients with Long COVID; all adult patients attending a specialised outpatient clinic of a hospital in Romania;  *M*(age Long COVID) = 44.06,  *SD* = 9.12; 54.5% female | Persisting symptoms 3 to 12 weeks after acute COVID-19 | Depressive symptoms (HADS-D)  Anxiety symptoms (HADS-A) | Mental health difficulties (Depressive and Anxiety symptoms) significantly correlated with the number and intensity of persisting symptoms and reduced quality of life scores | RQ2 |
| Goodman et al. (2023) | Cross-sectional, prospectively sampled study reporting data on mental health differences between patients with and without Long COVID | *n* = 655 patients after COVID-19, including 20.3% (*n* = 126) with self-reported Long COVID and 79.7% (*n* = 495) without Long COVID; self-referred population-based adult sample from Texas, USA;  *M*(age Long COVID) = 36.9; 82.6% female  *M*(age no Long COVID) = 34.5; 73.0% female | Long COVID was defined as reporting any current symptom that developed within 3 months of a confirmed positive COVID-19 test that could not be explained by another diagnosis (assessed in a semi-structured questionnaire) | Depressive symptoms (PHQ-2), cut-off score for depression ≥ 3  Anxiety symptoms (GAD-2), cut-off score for anxiety ≥ 3  PTSD (PC- PTSD)  Suicide ideation (MSSI) | Depression, Anxiety, PTSD, and suicide ideation were higher in Long COVID patients compared to patients after COVID-19 without Long COVID | RQ1 |
| Gouraud et al. (2021) | Cross-sectional study with the aim to explore the associations of objective cognitive performances and psychological distress with cognitive complaints in COVID-19 survivors | *n* = 100 patients after COVID-19, including *n* = 29 patients with Long COVID; adult patients previously hospitalised for COVID-19 in a university hospital in France;  $\tilde{X}$(age all patients) = 60 (IQR 49.5-71.5); 29% female | Cognitive complaints at 1 month after hospital admission | Psychological distress (HADS) | Greater psychological distress was significantly associated with the presence of cognitive complaints at 1-month | RQ2 |
| Haider et al. (2023) | Cross-sectional study aiming to characterise multiple symptom domains in individuals reporting post-COVID-19 and compare its clinical phenotype with those with FMS and CFS | *n* = 707 patients, including *n* = 203 patients with Long COVID only and *n* = 186 with FMS or CFS only; adult sample recruited online by the University of Iowa, USA, using an anonymous survey link;  *M*(age Long COVID) = 35.1, *SD* = 10.5; 57.1% female  *M*(age FMS) = 41.7, *SD* = 15.0; 71.7% female  *M*(age CFS) = 35.5, *SD* = 13.5; 63.2% female | Self-reported physician diagnosis of post-COVID-19 according to the WHO clinical case definition | Depressive symptoms (PHQ-2)  Depressive symptoms (HADS)  Anxiety symptoms (HADS)  Catastrophising (CSQ-CAT)  Kinesiophobia (TSK-11)  Multisensory sensitivity/Somatosensory amplification (MSAS-7) | Patients with Long COVID reported lower kinesiophobia and multisensory sensitivity/somatosensory amplification than patients with FMS as well as less catastrophising than patients with CFS | RQ1 |
| Harenwall et al. (2022) | Prospective cohort study aiming to explore whether post-traumatic stress symptoms and breathlessness are related to fatigue severity among individuals with post-COVID-19 syndrome fatigue giving baseline cross-sectional data on the association between PTSD symptoms and persistent fatigue | *n* = 154 patients with Long COVID (persistent fatigue); adult health care professionals enrolled in a 7-week multidisciplinary rehabilitation course in the UK;  *M*(age Long COVID) = 47.29, *SD* = 10.44; 87% female | Persistent symptoms lasting for more than 12 weeks after infection | PTSD symptoms (3-item scale of the C19-YRS) | Baseline PTSD symptoms were not significantly associated with baseline persistent fatigue | RQ2 |
| Hastie et al. (2022) | Prospective cohort study aiming to determine the frequency, nature, determinants and impact of Long COVID in the general population at 6, 12 and 18 months and giving cross-sectional data on the association between no recovery after COVID-19 and a pre-existing diagnosis of Depression or Anxiety | *n* = 31,486 patients after symptomatic COVID-19, including *n* = 15,206 patients with Long COVID; adult (> 16 years) Scottish population cohort;  $\tilde{X}$(age whole sample) = 45 (IQR 31-56); 61% female | Symptoms that last for at least 2 months after probable or confirmed SARS-CoV-2 infection and cannot be explained by an alternative diagnosis according to the WHO clinical case definition (assessed via self-completed online questionnaire) | Pre-existing Depression/Anxiety (ICD-10 codes F30-F33, or anti-depressant, hypnotic or anxiolytic (BNF 4.1;4.3) in electronic health records, or self-report) | Following previous symptomatic infection, pre-existing Depression or Anxiety was associated with lack of complete recovery | RQ2 |
| Hedberg et al. (2023) | Cross-sectional study with the aim to investigate the occurrence and healthcare use trajectory of PCC in SARS-CoV-2-positive individuals with and without a PCC diagnosis | *n* = 204,805 patients after COVID-19, including *n* = 3,066 patients with Long and *n* = 201,739 patients without Long COVID; register- and population-based adult sample from Sweden;  Long COVID:  $\tilde{X}$(age non-hospitalised) = 48.0 (IQR 40.0-56.0); 71.3% female  $\tilde{X}$(age hospitalised) = 60.0 (IQR 52.0-70.0); 46.5% female  $\tilde{X}$(age ICU-treated) = 59.0 (IQR 51.0-66.0); 33.4% female  No Long COVID:  $\tilde{X}$(age non-hospitalised) = 42.0 (IQR 31.0-53.0); 52.8% female  $\tilde{X}$(age hospitalised) = 65.0 (IQR 52.0-78.0); 42.9% female  $\tilde{X}$(age ICU-treated) = 62.0 (IQR 53.0-69.0); 28.9% female | Post COVID-19 condition diagnosis (ICD-10: U09.9) registered any time from 90 to 360 days after first positive SARS-CoV-2 test, given by any healthcare professional in primary care, outpatient specialist care or inpatient care (obtained from health records) | Pre-existing mental health disorders (ICD-10: F20-F29, F30-F39, F40-F48; health records) | Pre-existing mental health disorders were more common in patients with Long COVID than patients without Long COVID and pre-existing mental health disorders were significantly associated with a PCC diagnosis among non-hospitalised and hospitalised individuals | RQ1; RQ2 |
| Heine et al. (2023) | Cross-sectional study aiming to explore the clinical characteristics of post-COVID fatigue, describe associated structural imaging changes, and determine what influences fatigue severity | *n* = 47 adult patients with Long COVID and *n* = 47 matched healthy controls without a history of COVID-19; adult sample prospectively recruited from the neurological post-COVID outpatient clinic of a German university hospital;  *M*(age Long COVID) = 43.4, *SD* = 11.9; 82.9% female  *M*(age no Long COVID) = 44.5, *SD* = 14.1; 82.9% female | History of confirmed SARS-CoV-2 infection (i.e., positive RT-PCR test) with postinfectious neurological symptoms for at least 3 months and no history of relevant neurological disease prior to COVID-19 (assessed at outpatient clinic) | Depressive symptoms (BDI-II)  Anxiety symptoms (BAI) | Patients with Long COVID had increased levels of depressive and anxiety symptoms compared to healthy controls; higher levels of depressiveness and anxiety significantly correlated with fatigue severity and post-acute depressiveness was significantly associated with post-COVID fatigue | RQ1; RQ2 |
| Hill et al. (2023) | Large retrospective study aiming to identify risk factors associated with a Long COVID diagnosis | *n* = 7,512 patients with Long COVID and *n* = 37,560 patients after COVID-19 without Long COVID; adult sample from 31 health systems in the US;  Age groups(Long COVID)  18-29 y: 7.7%  30-39 y: 14.5%  40-49 y: 21.0%  50-59 y: 23.1%  60-69 y: 19.3%  70-79 y: 10.2%  80-89 y: 3.6%  90+ y: 0.6 %; 63.3% female  Age groups(no Long COVID)  18-29 y: 18.3%  30-39 y: 17.8%  40-49 y: 17.3%  50-59 y: 17.4%  60-69 y: 15.5%  70-79 y: 9.2%  80-89 y: 3.7%  90+ y: 0.8; 59.9% female | Clinical diagnosis of Long COVID (ICD-10: U09.9) | Depression (diagnosis)  Psychosis (diagnosis)  Substance abuse (diagnosis)  Tobacco smoker (diagnosis) | A pre-existing diagnosis of depression significantly predicted a higher risk of subsequent Long COVID whereas other mental health conditions (psychosis, substance abuse, and tobacco smoker) predicted a lower risk | RQ3 |
| Hirahata et al. (2022) | Cross-sectional study aiming to examine sociodemographic and clinical characteristics of Long COVID in Japan | *n* = 1,891 patients with Long COVID; adult sample collected in an outpatient clinic in Japan;  *M*(age Long COVID) = 37.8, *SD* = 12.2; 59.7% female | Long COVID patients were defined as those who were symptomatic after 28 days from COVID-19 onset in accordance with the Centres for Disease Control and Prevention | Depressive symptoms (yes/no; self-report obtained from electronic medical records) | Depressive symptoms were significantly associated with lower performance status | RQ2 |
| Hirschtick et al. (2023) | Cross-sectional study aiming to examine Long COVID symptoms in a US population-based probability sample | *n* = 1,913 patients after COVID-19, including 40.0% (*n* = 765) patients with Long COVID; adult sample from the US;  Age groups(Long COVID)  18-24 y: 16.8%  25-34 y: 20.0%  35-44 y: 16.6%  45-54 y: 17.1%  55-64 y: 16.4%  65+ y: 13.0%; 55.0% female | Presence of any symptom for at least 90 days post COVID-19 onset | Pre-existing psychological condition (self-report yes/no) | A pre-existing psychological condition was significantly associated with Long COVID | RQ2 |
| Huang et al. (2022) | Prospective cohort study aiming to characterise the longitudinal evolution of health outcomes in hospital survivors with different initial disease severity up to 2 years after acute COVID-19 infection and to determine their recovery status and giving data on differences in depressive, anxiety and PTSD symptoms between patients with and without Long COVID at 2 years | *n* = 1,190 patients after COVID-19, including *n* = 650 patients with Long COVID and *n* = 540 patients without Long COVID; adult patients previously hospitalised for COVID-19 in a hospital in China;  $\tilde{X}$(age all patients) = 57.0 (IQR 48.0-65.0); 46% female | Long-term effects after COVID-19 assessed at 6 months, 1 year and 2 years; COVID-19 survivors with Long COVID symptoms were defined as having at least one sequelae symptom (i.e., newly occurring and persistent, or worse than the status before getting COVID-19, and not explained by an alternative disease) at follow-up | Depressive symptoms (PHQ-9), cut-off score for depression ≥ 5  Anxiety symptoms (GAD-7), cut-off score for anxiety ≥ 5  PTSD symptoms (PCL-C), cut-off score for PTSD ≥ 38 | COVID-19 survivors with Long COVID symptoms at 2 years had more mental health abnormality than survivors without Long COVID symptoms (significantly higher percentage of individuals with depression and anxiety in sample with Long COVID compared to without) | RQ1 |
| Jacobs et al. (2023) | Prospective cohort study aiming to investigate comorbid conditions associated with PASC | *n* = 1,224 patients after COVID-19, including *n* = 518 patients with Long COVID and *n* = 706 patients without Long COVID; self-referred population-based adult sample from Arizona, USA;  $\tilde{X}$(age Long COVID) = 49 (IQR 36-60); 45.6% female  $\tilde{X}$(age no Long COVID) = 47 (IQR 33-60); 54.4% female | Self-reported continuing or new symptoms 28 days or more following the test date for the acute SARS-CoV-2 infection in accordance with the case definition for PASC from the Centres for Disease Control and Prevention | Pre-existing Depression/Anxiety (self-reported information provided in electronic survey) | Pre-existing Depression/Anxiety was significantly more frequent in patients with Long COVID compared to patients without Long COVID and pre-existing Depression/Anxiety was significantly associated with the development of PASC | RQ1; RQ3 |
| Jung et al. (2022) | Retrospective cross-sectional study aiming to compare the clinical characteristics and subjectively reported symptoms of the acute coronavirus disease (COVID) phase and those of the post-acute COVID phase to examine varying factors that affect the number of persistent symptoms and their categories | *n* = 1,122 patients after COVID-19, including *n* = 447 patients with Long COVID and *n* = 675 patients < 4 weeks after COVID-19; adult outpatients visiting a Post COVID-19 clinic in South Korea;  *M*(age Long COVID) = 52.1, *SD* = 14.4; 66.0% female  *M*(age no Long COVID) = 50.9, *SD* = 15.9; 66.1% female | Persistent symptoms > 4 weeks since the onset of COVID-19 condition | Depressive symptoms (HADS)  Anxiety symptoms (HADS)  Depression (self-developed questionnaire)  Anxiety (self-developed questionnaire) | Depressive and Anxiety symptoms assessed with the HADS were significantly associated with the number of Long COVID symptoms | RQ2 |
| Jung et al. (2023) | Retrospective cross-sectional study aiming to investigate the factors associated with neurological manifestations of post-coronavirus disease 2019 (COVID-19) conditions | *n* = 440 patients with Long COVID; adult outpatients visiting a Post COVID-19 clinic in South Korea;  *M*(age Long COVID) = 51.9, *SD* = 14.3; 66.4% female | Persistent symptoms > 4 weeks since the onset of COVID-19 condition | Depressive symptoms (HADS), cut-off score for depression ≥ 8, cut-off score for severe depression ≥ 11  Anxiety symptoms (HADS), cut-off score for anxiety ≥ 8, cut-off score for severe anxiety ≥ 11 | Anxiety symptoms assessed with the HADS were significantly associated with brain fog; Anxiety (HADS-Anxiety score ≥ 8), severe Anxiety (HADS-Anxiety score ≥ 11), Depression (HADS-Depression score ≥ 8) and severe Depression (HADS- Depression score ≥ 11) were significantly associated with brain fog, headache, dizziness and memory impairment | RQ2 |
| Kim et al. (2022) | Prospective cohort study aiming to investigate  the impact of long-term consequences of COVID-19 on Korean patients to determine the characteristics of persistent symptoms, quality of life, lifestyle, and mental health and giving data on differences in depressive, anxiety and PTSD symptoms between patients with and without Long COVID after 12 months from acute SARS-CoV-2 infection | *n* = 170 patients after COVID-19, including *n* = 129 patients with Long COVID and *n* = 41 patients without Long COVID; adult patients recruited at a single tertiary university hospital in South Korea and followed at 6 and 12 months after acute COVID-19 symptom onset or diagnosis;  $\tilde{X}$(age Long COVID) = 52 (IQR 39-61); 64.3% female  $\tilde{X}$(age no Long COVID) = 44 (IQR 30-54); 46.3% female | The symptom persistent group was defined as patients with at least one of 38 categorised persistent symptoms after acute COVID-19 infection | Depressive symptoms (PHQ-9)  Anxiety symptoms (GAD-7)  Anxiety/Depression (EQ5D-5L)  PTSD symptoms (PCL-5-K) | Anxiety/Depression assessed with the EQ5D-5L was more frequent and PTSD symptoms were higher in patients with Long COVID than in patients without Long COVID | RQ1 |
| König et al. (2023) | Prospective cohort study aiming to investigate the prevalence of persistent fatigue after COVID-19 in patients with mild infection (managed in primary care) during the first wave of the pandemic and to determine prognostic factors for persistent fatigue and providing data on differences in neuroticism between patients with and without Long COVID | *n* = 179 non-hospitalised patients after mild COVID-19 (including *n* = 49 patients with persistent fatigue) and *n* = 122 matched controls without a history of COVID-19; adult patients recruited from four Dutch GP practices at 3, 6, and 15 months after contacting their GP;  *M*(age COVID-19) = 47.3, *SD* = 12.9; 65% female  *M*(age no COVID-19) = 48.6, *SD* = 13.2; 59% female | “Persistent fatigue”: “fatigue” (CIS8R ≥ 27) scored on all three follow-ups or at two follow-ups with missing data from the other occasion | Neuroticism (EPQR-S)  Life events (LTE-Q)  Resilience (SOC-13) | Patients with a higher neuroticism score, more life events in the previous year and a lower resilience score had greater odds of persistent fatigue (Long COVID) after COVID-19 | RQ1 |
| Kostev et al. (2022a) | Retrospective cohort study aiming to investigate the prevalence of and the factors associated with post-coronavirus disease 2019 (COVID-19) condition (PCC) in the 12 months after the diagnosis of COVID-19 in patients followed in general practices in Germany | *n* = 51,630 patients after COVID-19, including 8.3% (*n* = 4,286) patients with Long COVID; adult patients from 855 general practices in Germany;  *M*(age whole sample) = 47.1, *SD* = 19.8; 54.3% female | Post-COVID-19 condition diagnosis (ICD-10: U09.9) 91 to 365 days after the COVID-19 index date (visit date on which COVID-19 was diagnosed for the first time) | Pre-existing Depression (ICD-10: F32 and F33 documented in a clinical database in the 12 months before the index date)  Pre-existing Anxiety disorders (ICD-10: F41 documented in a clinical database in the 12 months before the index date)  Pre-existing reaction to severe stress and adjustment disorders (ICD-10: F43 documented in a clinical database in the 12 months before the index date)  Pre-existing nicotine dependence (ICD-10: F17 documented in a clinical database in the 12 months before the index date) | Pre-existing reaction to severe stress and adjustment disorders were positively and significantly associated with post-COVID-19 condition, while depression, anxiety and nicotine dependence were not | RQ3 |
| Kostev et al. (2022b) | Retrospective cohort study aiming to investigate the prevalence of and the factors associated with post-COVID-2019 in patients aged < 18 years who were diagnosed with COVID-19 in general and pediatric practices in Germany at least 3 months after COVID-19 diagnosis | *n* = 6,568 patients after COVID-19, including 1.7% (*n* = 114) patients with Long COVID; children and adolescents (age < 18 years) from 524 general and 81 pediatric practices in Germany;  *M*(age Long COVID) = 12.1, *SD* = 4.7; 54.4% female  *M*(age no Long COVID) = 10.0, *SD* = 4.9; 49.1% female | Post-COVID-19 condition diagnosis (ICD-10: U09.9) | Pre-existing Anxiety disorders (ICD-10: F41 documented in a clinical database in the 12 months before the visit date on which COVID-19 was diagnosed for the first time)  Pre-existing reaction to severe stress and adjustment disorders (ICD-10: F43 documented in a clinical database in the 12 months before the visit date on which COVID-19 was diagnosed for the first time)  Pre-existing disorders of psychological development (ICD-10: F80-F89 documented in a clinical database in the 12 months before the visit date on which COVID-19 was diagnosed for the first time) | Pre-existing Anxiety disorders were more frequent in patients with post-COVID-19 condition than in patients without post-COVID-19 condition and were significantly associated with post-COVID-19 condition | RQ1; RQ3 |
| Kozik et al. (2023) | Cross-sectional study aiming to examine patient-reported and clinical predictors of cognitive deficits in patients with Long COVID and socio-demographically comparable healthy controls | *n* = 282 patients with Long COVID and *n* = 52 healthy controls without a history of COVID-19; patients presenting to a post-COVID-19 outpatient clinic at a University Hospital in Germany;  *M*(age Long COVID) = 46.84, *SD* = 11.30; 66% female  *M*(age no Long COVID) = 45.62, *SD* = 10.15; 60% female | Manifestation of symptoms during or after SARS-CoV-2 that persist for over 4 weeks without explanation by another diagnosis | Depression (PHQ-9) | Patients with Long COVID showed significantly higher depression scores than controls | RQ1 |
| Lhuillier et al. (2022) | Prospective cohort study of first responders after the 9/11 attacks providing cross-sectional and data on differences in depressive symptoms between patients with and without Long COVID and on the association between depressive symptoms and Long COVID | *n* = 1,280 patients after COVID-19, including *n* = 366 patients with Long COVID and *n* = 853 patients without Long COVID; individuals enrolled in a health program for World Trade Centre first responders in the US;  *M*(age Long COVID) = 56.9, *SD* = 7.41; 10.1% female  *M*(age no Long COVID) = 56.9, *SD* = 7.27; 7.9% female | PASC was defined as any COVID-19-related symptoms that lasted at least 4 weeks after symptom onset | Depressive symptoms (PHQ-9) | Patients with Long COVID had significantly higher depressive symptoms than patients without but depressive symptoms were not significantly associated with Long COVID | RQ1; RQ2 |
| Liang et al. (2023) | Cross-sectional study aiming to compare cognitive performance, psychiatric symptoms and DTI metrics between Long COVID patients and controls | *n* = 23 patients with Long COVID and *n* = 24 healthy controls without a history of COVID-19; community sample from the US;  *M*(age Long COVID) = 44.1, *SD* = 12.2; 65.2% female  *M*(age no Long COVID = 44.3, *SD* = 12.5; 54.2% female | Documented history of COVID-19 at least 6 weeks earlier and at least one new cognitive or neuropsychiatric symptom after COVID-19 | History of depression prior to COVID-19 (self-report yes/no)  History of anxiety disorders prior to COVID-19 (self-report yes/no)  Self-reported depression (PROMIS)  Self-reported anxiety (PROMIS)  Perceived stress (NIHTB-EB) | Compared to controls, patients with Long COVID had greater psychiatric symptoms and perceived stress | RQ1 |
| Lier et al. (2022) | Cross-sectional study aiming to better understand which neurological, cognitive, psychiatric, and psychosomatic symptoms mostly affect the functional long-term outcome of patients with SARS-CoV-2 infection | *n* = 219 patients with Long COVID; patients from an interdisciplinary outpatient clinic in Germany;  $\tilde{X}$(age Long COVID) = 49 (IQR 36.75-58.25); 64.5% female | Constellation of symptoms which develops following a severe acute SARS-CoV-2 infection and persists for more than 12 weeks, while not being explained by an alternative diagnosis | Depression (PHQ-9)  Anxiety (GAD-7) | There was a strong significant association of depression and anxiety with functional impairment | RQ2 |
| Liu et al. (2023) | Prospective cohort study aiming to describe the characteristics of patients with perceived cognitive deficits within the first 4 weeks of SARS-CoV-2 infection and the association of those deficits with Long COVID symptoms in 30, 60 and 90 days FU | *n* = 766 patients after COVID-19, including *n* = 223 patients with Long COVID and *n* = 543 patients after COVID-19 without Long COVID; adults from a SARS-CoV-2 Ambulatory Program in California, USA;  Age groups(Long COVID)  18-39 y: 12.6%  40-59 y: 34.5%  ≥ 60 y: 52.9%; 50.2% female  Age groups(no Long COVID)  18-39 y: 4.2%  40-59 y: 3.1%  ≥ 60 y: 52.7%; 47.0% female | Persistent SARS-CoV-2 symptoms among 9 symptom clusters on the 90-day survey (or the 60-day survey if the 90-day survey was incomplete) | History of depression (ICD-10 code F32)  History of anxiety (ICD-10  code F41) | A history of depressive disorder was significantly more frequent in patients with Long COVID than controls while there was no significant difference between groups regarding a history of anxiety disorder and both conditions did not predict the development of Long COVID | RQ1; RQ3 |
| Loosen et al. (2022) | Cross-sectional study aiming to determine risk factors for the development of Long COVID | *n* = 50,402 patients after COVID-19, including *n* = 1,708 patients with Long COVID; data from 1,056 general practices in Germany;  Age groups(Long COVID)  ≤ 30 y: 2.0%  31-45 y: 2.9%  46-60 y: 4.6%  > 60 y: 3.6%; 61.8% female | Long COVID was identified based on the original diagnosis text of the physicians (“long COVID syndrome”, “post COVID syndrome, “post COVID complications”); the following ICD-10 diagnoses were additionally used as surrogates for Long COVID if one or more of these diagnoses were documented within the time period between 90 and 183 days after the diagnosis of COVID-19: chronic fatigue (ICD-10: G93.3), abnormalities of breathing (ICD-10: R06), disturbances of smell and taste (ICD-10: R43), malaise and fatigue (ICD-10: R53, disturbances in attention (ICD-10: R41.8) | Depression (ICD-10 code F32 or F33) | Depression in women was significantly associated with an increased likelihood of developing Long COVID | RQ2 |
| Luedke et al. (2023) | Retrospective study aiming to explore the relationships between cognitive difficulties, mood, and functional impairment in paediatric patients with Long COVID | *n* = 34 patients with Long COVID; paediatric sample from the US;  *M*(age Long COVID) = 14.06, *SD* = 2.85; 64.7% female | Lingering or emergent neurologic, cognitive, and/or behavioural symptoms for at least 4 weeks following initial COVID-19 diagnosis or symptom onset | Depressive symptoms (Conners CBRS-Parent upsetting thoughts subscale used as proxy measure)  Anxiety symptoms (Conners CBRS-Parent worrying subscale used as proxy measure) | In children with Long COVID, low mood and anxiety significantly correlated with functional impairment and anxiety was significantly associated with functional impairment, while low mood was not | RQ2 |
| Magel et al. (2023) | Prospective cohort study aiming to examine the prevalence and outcome predictors of post-viral fatigue and related symptoms 3 and 6 months following symptom onset | *n* = 88 patients after COVID-19, including *n* = 58 patients with Long COVID at 3MFU and *n* = 47 patients with Long COVID at 6MFU; patients formerly hospitalised with COVID-19 and recruited from a Post-COVID-19 Respiratory Clinic in Canada;  *M*(age whole sample) = 61.1, *SD* = 16.2; 36.4% female  *M*(age Long COVID fatigue at 3MFU) = 59.5, *SD* = 17.7; 39.7% female  *M*(age COVID-19 without Long COVID fatigue at 3MFU) = 65.1, *SD* = 11.7; 31% female  *M*(age Long COVID fatigue at 6MFU) = 59.8, *SD*= 17.9; 38.3 % female  *M*(age COVID-19 without Long COVID fatigue at 6MFU) = 62.5, *SD* = 14.8; 37.5% female | Confirmed SARS-CoV-2 infection and 3- and 6-months post-symptom of fatigue | Depression (PHQ-9)  Pre-existing anxiety/depression (yes/no) | Patients with Long COVID showed significantly higher depression scores than controls and there were significant associations between depression as well as pre-existing anxiety/depression with fatigue at 3-month follow-up | RQ1; RQ2 |
| Magnavita et al. (2023) | Cross-sectional retrospective study aiming to evaluate the incidence of SARS-CoV-2 infection in workers and to determine whether protracted symptoms were associated with occupational stress, sleep disturbances or mental health | *n* = 115 patients with Long COVID and *n* = 49 patients after COVID-19 without Long COVID; occupational cohort consisting of adult workers from Italy;  *M*(age Long COVID) = 49.98, *SD* = 9.93; 75.6% female  *M*(age no Long COVID) = 49.16, *SD* = 12.34 | Post-COVID  syndrome cases = workers who had symptoms 4 weeks after the acute phase | Stress (ERI-Short Version)  Anxiety (GADS)  Depression (GADS) | Patients with Long COVID were found to have increased anxiety and depression scores compared to controls and anxiety was significantly associated with the number of post-COVID symptoms, while depression was not | RQ1; RQ2 |
| Margalit et al. (2022) | Nested case-control study aiming to assess risk factors for Long COVID fatigue | *n* = 66 patients with Long COVID and *n* = 75 patients after COVID-19 without Long COVID; patients recruited from a COVID recovery clinic in Israel;  *M*(age Long COVID) = 48.85, *SD* = 11.51; 60.6% female  *M*(age no Long COVID) = 45.21, *SD* = 14.45; 57.3% female | Fatigue at least 2 months following a PCR-proven diagnosis of COVID-19 | Recreational physical activity prior to COVID-19 and following COVID-19 (Mean and *SD* minutes/week)  Depression (PHQ-9) | Patients with Long COVID had significantly higher depression scores than controls and there was no significant difference between groups regarding physical activity | RQ1 |
| Marinkovic et al. (2023) | Cross-sectional study aiming to understand the neural mechanisms underlying Long COVID in a group of young, previously healthy adults | *n* = 18 patients with Long COVID and *n* = 20 healthy controls without a history of COVID-19; adult participants recruited from a local community in the US;  *M*(age Long COVID) = 24.4, *SD* = 5.2; 61% female  *M*(age no Long COVID) = 23.35, *SD* = 3.66; 60% female | Persistent symptoms for two months or longer | Stress (PSS)    Generalised Anxiety Disorder (GAD-7)  Depression (PHQ-9) | There were no significant group differences regarding perceived stress, anxiety, and depression | RQ1 |
| Martin et al. (2023) | Cross-sectional study aiming to assess whether tonic alertness as a neurocognitive index of arousal is reduced in patients with Long COVID and providing data on differences between patients with Long COVID and healthy controls in terms of depression | *n* = 40 patients with Long COVID and *n* = 40 healthy controls without a history of COVID-19; adult sample from Germany;  *M*(age Long COVID) = 47.95, *SD* = 8.44; 80% female  *M*(age no Long COVID) = 44.05, *SD* = 12.25; 65% female | According to the NICE guidelines | Depression (HADS-D) | Patients with Long COVID reported significantly higher levels of depression than healthy controls | RQ1 |
| Mazza et al. (2022) | Longitudinal study with 1MFU, 3MFU, 6MFU, and 12MFU aiming to investigate  the prevalence,  the course over  time, and the  risk factors  of post-COVID  fatigue | *n* = 265 patients after COVID-19, including *n* = 59 patients with Long COVID at 1MFU; patients formerly hospitalised for clinical and radiological findings suggestive of COVID-19 pneumonia in Milan, Italy;  *M*(age Long COVID) = 56.88, *SD* = 9.62; 49.2% female  *M*(age no Long COVID) = 56.79, *SD* = 10.98; 32.5% female | Fatigue at 1-month follow-up (or longer) | Depression (BDI-13), cut-off score ≥ 9 = yes  Depression (ZSDS), cut-off score ≥ 50 = yes  Positive psychiatric history (self-report yes/no) | Patients with Long COVID more often had a positive psychiatric history and depression than controls (except for positive psychiatric history at 3MFU, 6MFU and 12MFU as well as depression measured with the ZSDS index at 12MFU); only depressive symptomatology at one month predicted the presence of post-COVID-19 long-term fatigue | RQ1; RQ3 |
| Messin et al. (2021) | Retrospective observational descriptive study aiming to describe the natural course of COVID-19 symptoms at 6 months and to identify possible factors favouring the resurgence or persistence of these symptoms | *n* = 53 patients with Long COVID and *n* = 21 patients after COVID-19 without Long COVID; patients treated at a hospital in France;  *M*(age Long COVID) = 54.7, *SD* = 16.9; 56.6% female  *M*(age no Long COVID) = 46.1, *SD* = 19.6; 66.7% female | Presence of persistent symptoms at 6 months (+/-2 months) after SARS-CoV-2 infection | Anxiety (ordinal scale: 0 = not anxious, 1 = slightly anxious, 2 = moderately anxious, 3 = highly anxious, 4 = very highly anxious) | There was no significant difference between patients with Long COVID and controls regarding anxiety | RQ1 |
| Meza-Torres et al. (2022) | Cross-sectional study aiming to determine which symptoms people present to primary care after COVID-19 infection and compare Long COVID cases vs. cases with infection only | *n* = 416,505 patients after COVID-19, including *n* = 7,623 patients with Long COVID and *n* = 408,882 patients after COVID-19 without Long COVID; routine data from nationally representative primary care cohort from the UK;  *M*(age Long COVID) = 47.7, *SD* = 14.82; 64.6% female  *M*(age no Long COVID) = 44.5, *SD* = 21.77; 55.7% female | Long COVID cases were defined using a phenotype, with Long COVID cases being defined by a clinical term for a case, referral to an Long COVID service, or a symptom score suggestive of Long COVID based on the ONS set of possible Long COVID symptoms | History of depression (yes/no)  History of anxiety (yes/no) | Patients with Long COVID had significantly more often a history of depression or anxiety and depression and anxiety were significantly associated with Long COVID | RQ1; RQ2 |
| Mikuteit et al. (2023) | Cross-sectional study aiming to investigate different potential biomarkers of oxidative stress in serum and urine samples of Long COVID patients and providing data on differences between patients with Long COVID and healthy controls in terms of anxiety and depression | *n* = 124 patients with Long COVID and *n* = 24 healthy controls without a history of COVID-19; adult sample from Germany;  *M*(age Long COVID) = 42.7, *SD* = 11.8; 75.7% females  *M*(age no Long COVID) = 44.8, *SD* = 12.8; 54.2% females | Sequelae after COVID-19 with SARS-CoV-2 infection longer than 4 weeks ago | Depression (self-perceived intensity)  Anxiety (self-perceived intensity) | Long COVID patients reported significantly higher levels of depression and anxiety than healthy controls | RQ1 |
| Milde et al. (2023) | Prospective study in acute, subacute (3 months after symptom onset) and chronic phases (6 months after symptom onset) of COVID-19 aiming to investigate the role of psychological factors in the development of Long COVID | *n* = 91 patients with Long COVID (subacute or chronic); patients recruited via German media;  *M*(age Long COVID) = 41.6, *SD* = 13.6; 61.7% female | Impairment by COVID symptoms measured by the PHQ-15 plus 9 specific symptoms 3 or 6 months after symptom onset after acute infection | Fear of COVID-related health consequences (FCV-19S)  Positive and negative trait affect (PANAS)  Anxiety and depression (STADI)  Chronic stress (TICS) | Fear of COVID-related health consequences predicted higher odds of reporting any COVID symptoms in the subacute and chronic phases, whereas it only predicted a higher magnitude of COVID-related symptom impairment in the subacute phase; in subsequent exploratory analyses, other psychological factors were associated with an overall increase (i.e., chronic stress and depression) or decrease (i.e., trait positive affect) in the odds and magnitude of COVID-related symptom impairment | RQ3 |
| Mora et al. (2023) | Cross-sectional study aiming to determine the prevalence of Long COVID and identify its clinical manifestations among farmworkers in California and providing data on differences between patients with Long COVID and controls in terms of depression and anxiety | *n* = 94 patients with Long COVID, *n* = 58 patients after COVID-19 without Long COVID, *n* = 41 patients after covert COVID-19 without Long COVID, and *n* = 104 healthy controls without a history of COVID-19; adult sample from California, USA;  *M*(age Long COVID) = 43.8, *SD* = 9.9; 70.2% female  *M*(age COVID-19 without Long COVID) = 41.9, *SD* = 13.4; 56.9% female  *M*(age covert COVID-19 without Long COVID) = 42.6, *SD* = 12.7; 53.7% female  *M*(age HC) = 44.8, *SD*= 12.3; 52.9% female | Persistent symptoms > 28 days | Depression (PHQ-2)  Anxiety (GAD-2) | Individuals with Long COVID had higher adverse estimated mean scores for depression and anxiety compared to controls | RQ1 |
| Navas-Otero et al. (2023) | Cross-sectional study aiming to compare the prevalence of frailty-related factors between perimenopausal women with Long COVID, women who had successfully recovered from COVID-19, and controls | *n* = 68 patients with Long COVID, *n* = 68 patients after COVID-19 without Long COVID and *n* = 68 healthy controls without a history of COVID-19; Long COVID patients were recruited from the Long COVID Regional Association, recovered patients and HC were recruited from the community in Spain;  *M*(age Long COVID) = 42.62, *SD* = 9.65  *M*(age recovered) = 44.58, *SD* = 16.12  *M*(age HC) = 43.62, *SD* = 19.92 | According to the NICE guideline | Physical activity (IPAQ, METs)  Depressed mood (no information regarding assessment) | Statistically significant differences were found between the Long COVID group and the other two groups for physical activity as well as depressed mood and significant correlations between physical activity and symptom severity, functional disability, and overall health were found | RQ1; RQ2 |
| Nishimi et al. (2023) | Longitudinal cohort study aiming to examine psychological resilience to lifetime potentially traumatic events early in the pandemic and the risk of SARS-CoV-2 infection and somatic symptoms across 2 years of follow-up | *n* = 528 patients after COVID-19, including *n* = 81 (15.3%) patients with Long COVID; participants from the US who were re-invited in 2020 and 2021 after completing a screening questionnaire on trauma and stress in 2017-2018;  *M*(whole sample) = 37.8, *SD* = 11.2; 80.5% female | Presence of one or more symptoms more than 4 weeks after SARS-CoV-2 infection | Psychological resilience (factor considering lifetime exposure to potentially traumatic events and current psychological functioning) | Resilience was not significantly associated with the risk for reporting Long COVID | RQ2 |
| O’Sullivan et al. (2023) | Cross-sectional observational study aiming to describe cardiopulmonary, functional, and neurocognitive outcomes five months post-illness and comparing post-COVID-19 groups with a matched comparison group | *n* = 34 patients with Long COVID and *n* = 26 healthy controls without a history of COVID-19; exposed patients were recruited from the UK Defence COVID-19 Recovery Service; recovered patients and controls were recruited from local military units in the UK;  *M*(age Long COVID) = 37, *SD* = 10  *M*(age HC) = 38, *SD* = 8 | Continued presence of one or more post-COVID-19 symptoms more than 4 weeks after infection | Depression (PHQ-9)  Anxiety (GAD-7)  PTSD (PCL-5) | Patients with Long COVID showed significantly higher values for anxiety, depression, and PTSD compared to healthy controls | RQ1 |
| Pacho-Hernández et al. (2022) | Cross-sectional study aiming to identify potential direct and indirect effects on the relationships between sensitisation-associated symptoms, mood disorders such as anxiety/depressive levels, and sleep quality on health-related quality of life in people suffering from Long COVID pain | *n* = 146 patients with Long COVID pain; patients formerly hospitalised due to COVID-19 from three urban hospitals in Madrid, Spain;  *M*(age) = 57.5, *SD* = 12; 53.4% female | Post-COVID-19 pain symptoms starting no later than two months after hospitalisation and lasting for at least three months | Anxiety (HADS-A)  Depression (HADS-D)  Sensitisation (CSI) | Sleep quality mediated the relationship between sensitisation-associated symptoms and depressive levels with health-related quality of life | RQ2 |
| Pérez-López et al. (2023) | Cross-sectional study aiming to investigate sociodemographic and clinical factors associated with Long COVID among women living in Latin American countries | *n* = 164 patients with Long COVID and *n* = 183 patients after COVID-19 without Long COVID; female sample from nine Latin American countries;  Age groups(Long COVID)  40-52 y: 49.4%  53-64 y: 50.6%; 100% female  Age groups(no Long COVID)  40-52 y: 50.3%  53-64 y: 49.7%; 100% female | Long COVID symptoms persisting for more than four weeks | Use of antidepressants (yes/no)  Use of anxiolytics (yes/no)  Resilience (CD-RISC; dichotomised as higher score/lower score)  Fear of COVID-19 (FCV-19S; dichotomised as higher score/lower score) | Use of anxiolytics showed to be significantly associated with Long COVID whereas use of antidepressants, resilience, and fear of COVID-19 did not | RQ2 |
| Peters et al. (2022) | Cross-sectional, baseline analyses of a longitudinal study aiming to identify risk factors for persistent symptoms and the impact of COVID-19 on health-related quality of life | *n* = 1,406 patients with Long COVID and *n* = 524 patients after COVID-19 without Long COVID; workers in health and social services in Germany;  Age groups(Long COVID)  < 35 y: 16.3%  35-49 y: 25.7%  > 49 y: 58.0%; 84.9% female  Age groups(no Long COVID)  < 35 y: 25.2%  35-49 y: 30.0%  > 49 y: 44.8%; 73.5% female | Symptoms persisting beyond 12 weeks after the acute phase of SARS-CoV-2 infection | Depressive symptoms (PHQ-2), reported based on cut-off score  Anxiety symptoms (GAD-2), reported based on cut-off score | Patients with Long COVID reported higher levels of depression and anxiety than controls | RQ1 |
| Phu et al. (2023) | Cross-sectional study aiming to investigate the prevalence of Long COVID and mental health status among Thai  adults who had recovered from COVID-19 | *n* = 745 patients with Long COVID and *n* = 194 patients after COVID-19 without Long COVID; sample from the databases of a secondary care hospital and three field hospitals in southern Thailand;  Age groups(Long COVID)  19-59 y: 84.8%  > 59 y: 15.2%; 80.3% female  Age groups(no Long COVID)  19-59 y: 84.0%  > 59 y: 16.0%; 66.5% female | According to the WHO definition | Depression (DASS-21), cut-off score > 9  Anxiety (DASS-21), cut-off score > 9  Stress (DASS-21), cut-off score > 14 | Participants experiencing Long COVID symptoms were at higher risk of depression, anxiety, and stress than controls | RQ1 |
| Pływaczewska-Jakubowska et al. (2022) | Cross-sectional study aiming to assess factors, including lifestyle variables, related to the course of SARS-CoV-2 infection and to assess their impact on prolonged symptoms | *n* = 1,013 patients with Long COVID and *n* = 504 patients after COVID-19 without Long COVID; non-hospitalised participants of the STOP-COVID registry of the PoLoCOV-Study who, following COVID-19, underwent check-up examinations at a cardiology outpatient clinic in Poland;  $\tilde{X}$(age Long COVID) = 52 (IQR 42-62); 68.0% female  $\tilde{X}$(age no Long COVID) = 51 (IQR 41-61); 60.3% female | New or ongoing signs or symptoms associated with a SARS-CoV-2 infection persisting for more than 12 weeks | Physical activity (regular physical activity was defined as at least 150–300 min per week of moderate-intensity activity or 75–150 min per week of high-intensity activity during at least 3 months preceding COVID-19)  Stress (anxious, on edge, not being able to stop or control worrying more than half a day during 4 weeks preceding COVID-19) | There were no significant differences between the groups in terms of physical activity and stress and there was no significant relation between physical activity and Long COVID | RQ1; RQ2 |
| Rastogi et al. (2023) | Cross-sectional study investigating associations between post-acute sequelae of COVID-19 (PASC) and serious psychological distress during young adulthood | *n* = 19,862 patients with Long COVID and *n* = 44,652 patients after COVID-19 without Long COVID; sample of students of higher education institutions in the US;  Age groups(Long COVID)  18-24 y: 25.8%  25-30 y: 38.7%; 29.5% female  Age groups(no Long COVID)  18-24 y: 44.3%  25-30 y: 49.0%; 46.1% female | Single self-report item asking whether participants experienced symptoms continuing for more than four weeks post COVID-19 onset which they had not experienced beforehand | Prior mental health diagnosis (self-report yes/no)  Physical activity level not meeting guidelines (self-report yes/no) | Prior experience with mental illness and physical activity were lower in patients with Long COVID than in controls | RQ1 |
| Rocha et al. (2023) | Cross-sectional study aiming to investigate the relationship between physical activity before and after acute SARS-CoV-2 infection and the presence of Long COVID symptoms in adults | *n* = 2,919 patients after COVID-19, including *n =* 1,410 patients with Long COVID and *n* = 1,509 patients after COVID-19 without Long COVID; adult patients who received medical care in Rio Grande, Brazil;  Age groups(whole sample)  18-59 y: 48.6%  > 60 y: 46.6%; 58.9% female | Ongoing symptoms after SARS-CoV-2 infection at the time of the survey, at least 6 months after the SARS-CoV-2 infection | Physical activity (categories: remained inactive, became inactive, remained active) | The likelihood of experiencing Long COVID symptoms was significantly reduced in those patients who remained active | RQ2 |
| Roessler et al. (2022) | Cross-sectional study aiming to investigate post-COVID-19-associated morbidity and using 1:5 exact matching on age and sex, and propensity score matching on preexisting medical conditions (group with PCR-confirmed COVID-19 versus no COVID-19) | *n* = 11,950 children/adolescents after COVID-19, *n* = 145,184 adults after COVID-19, *n* = 59,750 cases from 51,825 healthy children/adolescents without a history of COVID-19 as controls and *n* = 723,165 cases from 557,287 healthy adults without a history of COVID-19 as controls; comprehensive health insurance data on 46% of the German population (children/adolescents and adults);    Age groups(children/adolescents after COVID-19)  0-11 y: 67.2%  12-17 y: 32.8%; 48.1% female  Age groups(adults after COVID-19)  18-24 y: 8.8% 25-39 y = 25.2%  40-49 y: 17.1%  50-54 y: 11.2%  55-59 y: 11.2%  60-64 y: 8.0%  65-69 y: 4.2%  70-74 y: 3.2%  75-79 y: 3.2%  80-plus y: 7.9%; 60.2% female  Age groups(healthy controls): same as COVID-19 groups | NICE guideline on Long COVID and the clinical case definition of post-COVID-19 condition proposed by the WHO, i.e., individuals were considered to enter the post-COVID-19 phase 3 months after diagnosis of COVID-19 | Anxiety disorders (as defined by health insurance records)  Depression (as defined by health insurance records)  Adjustment disorders (as defined by health insurance records) | Children/adolescents with COVID-19 diagnosis in the past had higher incidence rate ratios of anxiety disorders and depression than children/adolescents without past SARS-CoV-2 infection | RQ1 |
| Román-Montes et al. (2023) | Cross-sectional study aiming to describe the prevalence, characteristics, and impact on the quality of life of post-COVID-19 syndrome | *n* = 187 patients with Long COVID and *n* = 59 patients after COVID-19 without Long COVID; patients from Mexico with a history of hospitalisation for COVID-19;  $\tilde{X}$(age Long COVID) = 55 (IQR 41-63); 46% female  $\tilde{X}$(age no Long COVID) = 50 (IQR 39-69); 42% female | According to the WHO definition | Anxiety/depression (EQ-5D-5L), reported based on cut-off score | Anxiety/depression was higher in patients with Long COVID than in controls | RQ1 |
| Ruggeri et al. (2023) | Cross-sectional study aiming to investigate cognition of patients with  post-COVID-19 syndrome and to find clinical factors predicting cognitive and memory impairments | *n* = 18 patients with Long COVID; individuals attending a rehabilitation in Rome, Italy;  *M*(age Long COVID) = 55.06, *SD* = 8.67; 66.7% female | Cognitive difficulties 2-3 months after recovery from acute COVID-19 | Mood disorders (DASS-21) | Mood disorders were not significantly associated with cognitive dysfunctions | RQ2 |
| Salci et al. (2024) | Cross-sectional survey aiming to evaluate perceived quality of life and determine its association with personal, sociodemographic, behavioural, clinical and social variables; and assess significant predictors of high perceived quality of life in Long COVID | *n* = 403 patients with Long COVID; older Brazilian residents with clinical evidence of Long COVID;  Age groups(Long COVID)  60-74 y: 82.6%  ≥ 75 y: 17.4%; 54.1% female | Temporal criterion of 12 weeks after an acute phase or hospital discharge | Practice of physical activity (yes/no)  Depressive mood after 12 weeks up to 12 months (yes/no)  Anxiety symptoms after 12 weeks up to 12 months (yes/no) | Individuals with depressive mood and anxiety symptoms were more likely to show lower quality of life | RQ2 |
| Salve et al. (2023) | Cross-sectional study aiming to estimate the prevalence of Long COVID and its determinants | *n* = 77 patients with Long COVID and *n* = 133 patients after COVID-19 without Long COVID; patients from a subdistrict hospital in India;  Age groups(Long COVID)  < 40 y: 39.2%  > 40 y: 60.8%; 51.9% female  *M*(age whole sample) = 43.2, *SD* = 15.2; 47% female | Persistent symptoms at least 28 days after the onset of COVID-19 | Depression (PHQ-9) | Depression was significantly higher in patients with Long COVID than in controls and in the multivariate model, depression showed to be significantly associated with Long COVID | RQ1; RQ2 |
| Samper-Pardo et al. (2022) | Secondary data analysis from a randomised clinical trial aiming to identify factors associated with quality of life in Long COVID | *n* = 100 patients with Long COVID; patients from Spain treated by primary health care;  *M*(age Long COVID) = 48.28, *SD* = 9.27; 80% female | Ongoing COVID-19 symptoms (median time since infection = 18 months) | Physical activity (IPAQ-SF)  Depression and anxiety (HADS) | Physical activity significantly correlated with mental quality of life and depression and anxiety significantly correlated with mental and physical quality of life | RQ2 |
| Satar et al. (2023) | Cross-sectional study aiming to determine which patients’ quality of life was most compromised | *n* = 173 patients with Long COVID, patients from a pulmonary rehabilitation centre in Ankara, Turkey.  *M*(age Long COVID) = 56.21, *SD* = 10.64; 31.2% female | Prolonged COVID-19 symptoms at least three months after the end of isolation or hospital ward or intensive care unit discharge | Depression (HADS-D)  Anxiety (HADS-A) | Depression and anxiety showed to be significantly related to quality of life | RQ2 |
| Scholz et al. (2023) | Cross-sectional online survey aiming to examine experiences of social stigma in people with long COVID and their associations with perceived stress, depressive symptoms, anxiety, and mental and physical health-related quality of life | *n* = 253 patients with Long COVID; patients recruited via social media in Switzerland, Germany, and Austria;  *M*(age Long COVID) = 45.49, *SD* = 12.03; 88.5% female | Acute SARS-CoV-2 infection 12 weeks before or longer and persistent symptoms at the time of recruitment | Depressive symptoms (HADS)  Anxiety (HADS)  Perceived stress (PSS-4) | Depressive symptoms were significantly correlated with physical quality of life and mental quality of life, anxiety was significantly correlated with physical quality of life and mental quality of life, and stress was significantly correlated with mental quality of life | RQ2 |
| Selvakumar et al. (2023) | Prospective cohort study of adolescents and young adults aiming to determine the point prevalence of Long COVID 6 months after the acute infection, to determine the risk of development of Long COVID and to explore a broad range of potential risk factors | *n* = 382 patients after COVID-19, including *n* = 191 patients with post-COVID-19 condition and *n* = 48 with postinfective fatigue syndrome, and *n* = 85 healthy controls without a history of COVID-19 at 6-month follow-up; non-hospitalised individuals from Norway;  Age groups(patients after COVID-19)  12-15 y: 25.7%  15-18 y: 27.2%  18-21 y: 20.9%  21-25 y: 26.2%; 60.2% female  Age groups(healthy controls)  12-15 y: 21.2%  15-18 y: 36.5%  18-21 y: 24.7%  21-25 y: 17.6%; 63.5% female | According to the WHO definition of post-COVID-19 condition and the case definition for postinfective fatigue syndrome at 6 months; all participants were classified as cases or non-cases according to both definitions | Physical activity prior to infection (single questionnaire item)  Loneliness (single questionnaire item)  Negative life events prior to last year (life events checklist impact score) | Low physical activity and loneliness were risk factors for post-COVID-19 condition and negative life events prior to last year were a significant risk factor for postinfective fatigue syndrome | RQ3 |
| Shachar-Lavie et al. (2023) | Cross-sectional study aiming to compare mental health aspects in children with Long COVID to a control group of uninfected children | *n* = 103 patients with Long COVID and *n* = 113 healthy controls without a history of COVID-19; paediatric sample including children with Long COVID recruited from a designated multidisciplinary clinic for Long COVID at a paediatric tertiary centre in Israel;  *M*(age Long COVID) = 13.50, *SD* = 4.01; 54.4% females  *M*(age no Long COVID) = 10.14, *SD* = 2.80; 48.7% female | Symptoms suggestive of Long COVID > 4 weeks from acute COVID-19 that cannot be explained  by an alternative diagnosis | Connection with friends (1 item)  Physical activity (1 item)  Depression and anxiety (PSC internalising subscale)  Avoidance (CATS)  Negative cognitions (CATS) | No significant group differences emerged regarding all psychological variables and connection with friends was not significantly associated with Long COVID or functional impairment | RQ1; RQ2 |
| Song et al. (2023) | Large-scale study aiming to compare clinical characteristics of people who suffer from Long COVID and individuals who were infected with SARS-CoV-2 at the same time without Long COVID | *n* = 8,329 patients with Long COVID and *n* = 600,161 patients after COVID-19 without Long COVID; nationwide sample of insurance data from the US;  *M*(age Long COVID) = 56.6, *SD* = 17.0; 58% female  *M*(age no Long COVID) = 46.4, *SD* = 21.6; 50.9 female | Clinical diagnosis of Long COVID (ICD-10: U09.9) | Depression (diagnosis; Elixhauser Comorbidity Index)  Alcohol abuse (diagnosis; Elixhauser Comorbidity Index) | Patients with Long COVID had a higher prevalence of depression than controls and there was no significant difference between groups regarding alcohol abuse | RQ1 |
| Staples et al. (2023) | Cross-sectional study aiming to assess the prevalence and predictors of self-reported Long COVID in Australian adults | *n* = 1,873 patients with Long COVID, *n* = 6,151 healthy controls without a history of COVID-19, *n* = 2,417 patients with acute COVID-19, and *n* = 7,468 patients after COVID-19 without Long COVID; adult sample from Australia;  *M*(age Long COVID) = 35.8, *SD* = 13.0; 77.8% female  *M*(age HC) = 38.0, *SD* = 15.5; 71.2% female  *M*(age acute COVID-19) = 35.1, *SD* = 13.4; 77.2% female  *M*(age COVID-19 without Long COVID) = 33.2, *SD* = 12.1; 76.2% female | Continuing symptoms that patients associated with a previous SARS-CoV-2 infection over three months ago | Depression (PHQ-9)  Anxiety (GAD-7) | Patients reporting symptoms associated with Long COVID had higher depression and anxiety scores than controls | RQ1 |
| Subramanian et al. (2022) | Retrospective cohort study aiming to determine symptoms that are associated with confirmed SARS-CoV-2 infection beyond 12 weeks in non-hospitalised adults and the risk factors associated with developing persistent symptoms | *n* = 384,137 patients after COVID-19, including *n* = 29,869 patients with Long COVID; primary care data from the UK;  Age groups(Long COVID)  18-29 y: 23.2%  30-39 y: 19.4%  40-49 y: 19.4%  50-59 y: 18.4%  60-69 y: 9.3%  70+ y: 10.3%; 69.6% female  Age groups(whole sample)  18-29 y: 25.0%  30-39 y: 20.4%  40-49 y: 19.6%  50-59 y: 19.1%  60-69 y: 9.4%  70+ y: 6.6%; 55.3% female | According to the WHO definition | Depression (as a comorbidity)  Anxiety (as a comorbidity) | In the risk factor analysis, a Long COVID diagnosis was more likely for people with depression and anxiety 3-12 months prior to SARS-CoV-2 infection | RQ3 |
| Tavares-Júnior et al. (2022) | Cross-sectional study aiming to determine the relationship between cognitive impairment and COVID-19 after the acute phase of the disease and providing data on the association between depression and Long COVID | *n* = 141 patients with Long COVID (neurological symptoms); adult patients recruited in an outpatient clinic in Brazil;  *M*(age Long COVID) = 48, *SD* = 14; 89% female | Neurological symptoms that persisted for more than 3 months from COVID-19 onset | Depression (yes/no), measured depending on the age of the participants (BDI; cut-off score ≥ 10) or (GDS; cut-off score ≥ 3) | There was a significant difference regarding the proportions of depression between patients with subjective cognitive decline, cognitive impairment, and unimpaired cognition | RQ2 |
| Tebeka et al. (2023) | Cross-sectional study aiming to assess the associations between anxiety and depressive symptoms and post-COVID-19 condition | *n* = 1,095 patients with Long COVID and *n* = 1,021 patients after COVID-19 without Long COVID; French adults participating in a nationwide survey;  Age groups(Long COVID)  18-24 y: 9.8%  25-34 y: 27.8%  35-44 y: 19.5%  45-54 y: 17.7%  55-64 y: 14.0%  ≥ 65 y: 11.3%; 60.6 % female  Age groups(no Long COVID)  18-24 y: 9.2%  25-34 y: 25.7%  35-44 y: 17.1%  45-54 y: 17.9%  55-64 y: 15.8%  ≥ 65 y: 14.4%; 51.8 % female | According to the WHO definition | Depressive symptoms (PHQ-2); cut-off score for depression ≥ 3  Anxiety symptoms (GAD-2), cut-off score for anxiety ≥ 3  Chronic depression (self-report yes/no)  Chronic anxiety (self-report yes/no) | Depression and anxiety were higher in patients with Long COVID than controls; levels of depression and anxiety were both strongly correlated with the number of Long COVID symptoms and measured anxiety and chronic anxiety were significantly associated with Long COVID, while measured depression and chronic depression were not | RQ1; RQ2 |
| Terai et al. (2023) | Multicentre prospective cohort study aiming to assess the distribution of Long COVID in Japanese patients | *n =* 433 patients with Long COVID and *n* = 502 patients after COVID-19 without Long COVID at 3MFU; *n =* 350 patients with Long COVID and *n* = 515 patients after COVID-19 without Long COVID at 6MFU; *n =* 239 patients with Long COVID and *n* = 485 patients after COVID-19 without Long COVID at 12MFU; adult patients formerly hospitalised for confirmed COVID-19 in 26 medical institutions in Japan;  *M*(age whole sample at baseline) = 56.1, *SD* = 16.8; 36.3% female | Presence of one or more symptoms for at least three months after initial onset | Anxiety (HADS-A)  Depression (HADS-D)  Fear of COVID-19 (FCV-19S) | Patients with at least one Long COVID symptom showed lower quality of life and scored significantly higher on assessments for anxiety, depression, and fear of COVID-19 | RQ1 |
| Thronicke et al. (2022) | Cross-sectional study aiming to evaluate factors associated with Long COVID using national online survey data | *n* = 99 patients with Long COVID and *n* = 174 patients after COVID-19 without Long COVID; participants of an online survey from Germany;  $\tilde{X}$(age Long COVID) = 51.5 (IQR 39-60); 77.8% female  $\tilde{X}$(age no Long COVID): no data | Self-reported persisting symptoms after COVID-19 | COVID-19 Anxiety (yes/no) | Individuals reporting COVID-19 related anxiety were more likely to develop Long COVID | RQ2 |
| Tudor et al. (2023) | Cross-sectional online study aiming to investigate the association between Long COVID symptoms and self-compassion/psychological flexibility | *n* = 105 patients with Long COVID; users of social media platforms and members of self-help groups in the UK;  *M*(age Long COVID) = 45.14, *SD* = 10.85; 87% female) | According to the NICE guideline | Self-compassion (SCS-SF)  Psychological flexibility (Psy-Flex) | Self-compassion and psychological flexibility were negatively associated with symptom load and psychosocial impact | RQ2 |
| Uniyal et al. (2022) | Cross-sectional study aiming to investigate the prevalence of post-acute COVID-19 symptoms as well as the effect of demographic and clinical characteristics on the persistence of Long COVID | *n* = 18 patients with Long COVID and *n* = 226 patients after COVID-19 without Long COVID; adult patients from a tertiary care centre in northern India;  *M*(age Long COVID) = 43.94, *SD* = 11.37; 39% female  *M*(age no Long COVID) = 43.32, *SD* = 12.85; 40% female | Persistence of symptoms beyond 6 weeks after COVID-19 | Alcoholism (yes/no) | There was no significant difference between patients with and without Long COVID regarding alcoholism | RQ1 |
| Walker et al. (2023) | Cross-sectional single-arm study assessing the impact of symptoms as well as psychological characteristics on health-related quality of life, ability to work and undertake activities of daily living | *n* = 3,754 patients diagnosed with Long COVID using a digital health intervention; patients from Great Britain;  *M*(age Long COVID) = 47.7, *SD* = 12.3; 71% female | Patients experiencing post-COVID-19 symptoms for 12 weeks or more | Depression (PHQ-8)  Anxiety (GAD-7) | There was a significant association between depression and functional limitation as well as significant associations of depression and anxiety with health-related quality of life | RQ2 |
| Wang et al. (2022) | Prospective cohort study aiming to determine whether high levels of psychological distress before SARS-CoV-2 infection, characterised by depression, anxiety, worry, perceived stress, and loneliness, are prospectively associated with an increased risk of developing Long COVID | *n* = 3,193 patients after COVID-19, including *n* = 1,403 patients with Long COVID; participants from three large, predominantly female cohort studies from the US;  *M*(age Long COVID): no data; 97.5% female  *M*(age patients after COVID-19) = 55.3, *SD* = 13.8; 96.4% female | Participants experiencing long-term COVID-19 symptoms for more than 4 weeks after acute COVID-19 | Probable depression (PHQ-2)  Probable anxiety (GAD-2)  Worry about COVID-19 (single item)  Perceived stress (PSS-4)  Loneliness (UCLA Loneliness Scale) | The findings of this study suggest that preinfectional psychological distress may be a risk factor for post-COVID-19 conditions | RQ3 |
| Whiteside et al. (2022) | Cross-sectional study aiming to assess the association of cognitive outcomes in Long COVID | *n* = 49 patients with Long COVID; adult patients of a Post COVID outpatient clinic in the US;  *M*(age Long COVID) = 49.65, *SD* = 12.43; 83.7% female | Experiencing significant fatigue and cognitive concerns after COVID-19 | Depression (BDI-II)  Anxiety (BAI) | There were significant correlations between mood/anxiety scores and cognitive measures | RQ2 |
| Wright et al. (2024) | Case-control observational pilot study aiming to determine if patients that develop lingering neurologic symptoms of fatigue and brain fog after initial recovery from COVID-19 have persistent low growth hormone secretion and providing data regarding depression in Long COVID patients and other COVID-19 patients | *n* = 10 patients with Long COVID and *n* = 13 patients after COVID-19 without Long COVID; Long COVID patients formerly treated at a post COVID recovery clinic in the US and individuals without Long COVID recruited via flyers and online platforms;  *M*(age Long COVID) = 48.5, *SD* = 9.9; 80% female  *M*(age no Long COVID) = 46.8, *SD* = 14.9; 69.2% female | Persistent neurologic symptoms at least 6 months after initial SARS-CoV-2 infection | Depression (BDI-II) | Individuals with Long COVID showed significantly worse depression scores than patients after COVID-19 without Long COVID and depression showed significant correlations with fatigue and quality of life | RQ1; RQ2 |
| Yaksi et al. (2022) | Retrospective cohort study aiming to evaluate Long COVID frequency and related factory in patients followed up after hospitalisation | *n* = 133 patients after COVID-19, including *n* = 86 patients with Long COVID; patients formerly hospitalised in Turkey due to acute COVID-19;  *M*(age Long COVID) = no data; 54.7% female  *M*(age whole sample) = 65.7, *SD* = 13.1; 48.1% female | The presence of at least one symptom lasting more than four weeks | Depression (self-rating from 0-100) | Depression was not significantly associated with Long COVID | RQ2 |
| Zhang et al. (2023a) | Prospective cohort study aiming to identify predictors of Long COVID | *n* = 207 patients after COVID-19, including *n* = 168 patients with Long COVID; primary care patients with comorbid physical and psychosocial conditions, aged 55 years or above, from Hong Kong, China;  *M*(age whole sample) = 70.8, *SD* = 5.7; 76.3% female | Symptoms that persisted at least 4 weeks after SARS-CoV-2-infection | Depression (PHQ-9), cut-off scores: 0-4 normal, 5-9 mild depression, 10-27 moderate/severe depression  Anxiety (GAD-7), cut-off scores: 0-4 very mild anxiety, 5-9 mild anxiety, 10-21 moderate/severe anxiety | Higher levels of depression at baseline were significantly associated with post-COVID fatigue and higher anxiety levels were significantly associated with cognitive difficulties | RQ3 |
| Zhang et al. (2023b) | Longitudinal cohort study aiming to investigate the 3-year health outcomes of COVID-19 survivors and providing data on mental health differences between patients with Long COVID and controls | *n* = 753 patients with Long COVID and *n* = 606 patients after COVID-19 without Long COVID; adult sample formerly hospitalised for confirmed COVID-19 in a hospital in Wuhan, China;  $\tilde{X}$(age Long COVID) = 57  (IQR 48-65); 51% female  $\tilde{X}$(age no Long COVID) = 57 (IQR 46-65); 41% female | Presence of at least one symptom at 2YFU | Anxiety (GAD-7), cut-off score ≥ 5  Depression (PHQ-9), cut-off score ≥ 5  PTSD (PCL-C), cut-off score ≥ 38  Anxiety or depression (EQ-5D-5L) | The proportion of survivors with Long COVID who had worse mental health was significantly higher than that of COVID-19 survivors without Long COVID | RQ1 |
| Zheng et al. (2023a) | Prospective cohort study aiming to investigate determents of recovery from dyspnoea in adults with COVID-19 and compare these to determinants of recovery from non-COVID-19 dyspnoea and providing longitudinal data on pre-existing depression or anxiety as a risk factor for Long COVID | *n* = 990 patients after COVID-19; adult patients formerly hospitalised in the UK due to COVID-19;  *M*(age Long COVID)=59.1, *SD* = 11.9; 44.7% female | A history of COVID-19 and a MRC Dyspnoea score of at least 2 at 5MFU | Pre-existing depression or anxiety (as a comorbidity) | Higher odds of worsening of dyspnoea between 5MFU and 1YFU were observed among participants with pre-existing anxiety or depression | RQ3 |
| Zheng et al. (2023b) | Retrospective cohort study aiming to examine the independent roles of a sedentary lifestyle as a modifiable risk factor in the development of acute and post-acute COVID-19 sequelae among COVID-19 survivors | *n* = 610 patients with Long COVID, *n* = 833 patients after COVID-19 without Long COVID and *n* = 2,962 healthy controls without a history of COVID-19; adult patients from Hong Kong, China, recruited via social media, e-mails, and leaflets;  *M*(age COVID-19 survivors) = 34.1, *SD* = 13.1; 63.6% female  *M*(age HC) = 33.9, *SD* = 13.1; 61.7% female | Presence of one or more symptoms beyond 1 or 2 months from the onset of COVID-19 | Physical activity (IPAQ, < 150 min per week of moderate-to-vigorous intensity physical activity)  Sedentary behaviour (Sedentary behaviour questionnaire, ≥ 10 h per day) | Prolonged sedentary behaviour (≥ 10 h/day) before COVID-19 was positively associated with an increased risk of symptoms of post-acute COVID-19 sequelae, while physical inactivity was not | RQ2 |

*Note.* $\tilde{X}$ = median; ADHD = attention deficit hyperactivity disorder; BAI = Beck Anxiety Inventory; BDI-13 = Beck Depression Inventory-13; BDI-II = Beck Depression Inventory 2; BFSI = Big Five Structure Inventory; BNF = British National Formulary; C19-YRS = Yorkshire Rehabilitation Scale; CATS = Child and Adolescent Trauma Screen; CD-RISC = Connor-Davidson Resilience Scale; CFS = chronic fatigue syndrome; CIS8R = Checklist of Individual Strength 8R; Conners CBRS-Parent = Conners Comprehensive Behavior Rating Scale-Parent; CSI = Central Sensitization Inventory; CSQ-CAT = Coping Strategies Questionnaire-Catastrophizing subscale; C-SSRS = Columbia Suicide Severity Rating Scale; DASS-21 = Depression, Anxiety and Stress Scale; DTI = diffusion tensor imaging; EPQR-S = Eysenck Personality Questionnaire Revised-Short Form; EQ-5D-3L = European Quality of Life 5 Dimensions 3 Level Version; EQ-5D-5L = European Quality of Life 5 Dimensions 5 Level Version; ERI = Effort-Reward Imbalance Questionnaire; FABQ = Fear Avoidance Beliefs Questionnaire; FAS = Fatigue Assessment Scale; FCV-19S = Fear of COVID-19 Scale; FIVE = Fear of Illness and Virus Evaluation; FMS = fibromyalgia syndrome; FU = follow-up; GAD-2 = Generalized Anxiety Disorder Scale-2; GAD-7 = Generalized Anxiety Disorder Scale-7; GADS = Goldberg Anxiety and Depression Scale; GDS = Geriatric Depression Scale; GSLTPAQ = Godin-Shephard Leisure-Time PA; HADS = Hospital Anxiety and Depression Scale; HADS-A = Hospital Anxiety and Depression Scale (Anxiety); HADS-D = Hospital Anxiety and Depression Scale (Depression); HAMA = Hamilton Anxiety Rating Scale; HAMD = Hamilton Depression Scale; HAPYS = Health Anxiety by Proxy Scale; HC = healthy controls; ICU = intensive care unit; IES-R = Impact of Event Scale – Revised; IPAQ = International Physical Activity Questionnaire; IPAQ-SF = International Physical Activity Questionnaire Short Form; IPQ-R = Illness Perception Questionnaire-Revised; IQR = interquartile range; K6 = Kessler Psychological Distress Scale; LCSS = Long COVID Stigma Scale; LTE-Q = List of Threatening Experiences Questionnaire; *M* = Mean; ME/CFS = myalgic encephalomyelitis/chronic fatigue syndrome; METs = Resting Metabolic Units; MFU = month follow-up; MINI = Mini-International Neuropsychiatric Interview; MRC Dyspnea Scale = Medical Research Council Dyspnea Scale; MSAS-7 = Multisensory Amplification Scale; MSSI = Modified Scale for Suicide Ideation; Neuro-QOL = Quality of Life in Neurological Disorders; NIHTB-EB = National Institutes of Health Toolbox Emotion Battery; OCD = obsessive-compulsive disorder; ONS = Office for National Statistics; PAI = Personality Assessment Inventory; PANAS = Positive and Negative Affect Schedule; PASC = post-acute sequelae of COVID-19; PCC = post COVID-19 condition; PCL = PTSD Checklist; PCL-5 = PTSD Checklist for DSM-5; PCL-5-K = Posttraumatic Stress Disorder Checklist-5; PCL-C = PTSD Checklist – Civilian Version; PC-PTSD = Primary Care PTSD Screen; PCS = Pain Catastrophizing Scale; PCS = post-concussion syndrome; PHQ-15 = Patient Health Questionnaire-15; PHQ-2 = Patient Health Questionnaire-2; PHQ-8 = Patient Health Questionnaire-8; PHQ-9 = Patient Health Questionnaire-9; POMS = Profile of Mood States; PROMIS = Patient-Reported Outcomes Measurement; PSC = Pediatric Symptom Checklist; PSS = Perceived Stress Scale; PSS-4 = Perceived Stress Scale 4; Psy-Flex = Contextually Sensitive Measure of Psychological Flexibility; PTSD = post-traumatic stress disorder; RQ = research question; SAI = Soma Assessment Interview; SCS-SF = Self-Compassion Scale - Short Form; SD = standard deviation; SDQ = Strengths and Difficulties Questionnaire; SOC-13 = Sense of Coherence-13 Questionnaire; STADI = State Trait Anxiety and Depression Inventory; STAI-S = State-Trait-Anxiety Inventory (State); STAI-T = State-Trait-Anxiety Inventory (Trait); TICS = Trier Inventory for Chronic Stress; TMD = total mood disturbance; TSK = Tampa Scale for Kinesiophobia; TSK-11 = Tampa Scale for Kinesiophobia-11; VAS = Visual Analogue Scale; YFU = year follow-up; ZSDS = Zung Self-Rating Depression Scale.

## **eTable 2. Overview of included studies providing cross-sectional group comparisons on psychological variables (RQ1)**

| **Author**  **(year of publication)** | **Psychological**  **variable(s)** | **Long COVID (sample size)** | **Type of control group (*n*)** | **Long COVID Mean (*SD*)**  if not stated  otherwise | | **CG Mean (*SD*)**  if not stated otherwise | **Significant**  **difference**  **(*p*-value)** | **Effect size/**  **coefficients** | **Comments** | |
| --- | --- | --- | --- | --- | --- | --- | --- | --- | --- | --- |
| Al-Hadrawi et al. (2023) | Anxiety; Depression | *n* = 120 | Healthy controls without a history of COVID-19 (*n* = 36) | *M*(Anxiety) = 20.37, *SD* = 8.19 | | *M*(Anxiety) = 7.58, *SD* = 7.74 | <.0001 | – |  |  |
|  |  |  |  | *M*(Depression) = 17.36, *SD* = 5.44 | | *M*(Depression) = 5.09, *SD* = 5.16 | <.0001 | – |  |  |
| Alghamdi et al. (2022) | Depression; Anxiety | *n* = 277 | Patients after COVID-19 without Long COVID (*n* = 227) | No. and % of patients with depressive symptoms: 83 (36.7%) | | No. and % of patients with depressive symptoms: 48 (17.3%) | <.001 | – | Unclear how depressive symptoms were assessed |  |
|  |  |  |  | No. and % of patients with anxiety symptoms: 69 (39.4%) | | No. and % of patients with anxiety symptoms: 65 (25.5%) | <.001 | – |  |  |
| Alkwai et al. (2022) | Anxiety; Depression; other psychological illness | *n* = 108 | Patients after COVID-19 without Long COVID (*n* = 105) | No. and % of patients with anxiety: 14 (13%) | | No. and % of patients with anxiety: 6 (5.7%) | .056 | – |  |  |
|  |  |  |  | No. and % of patients with depression: 8 (7.4%) | | No. and % of patients with depression: 2 (1.9%) | .055 | – |  |  |
|  |  |  |  | No. and % of patients with other psychological illness: 4 (3.7%) | | No. and % of patients with other psychological illness: 0 (0%) | .064 | – |  |  |
| Ariza et al. (2022) | Depression; Anxiety | *n* = 319 | Healthy controls without a history of COVID-19 (*n* = 109) | *M*(Depression) = 9.13, *SD* = 6.64 | | *M*(Depression) = 3.08, *SD* = 2.79 | <.001 | *d* = 1.023 |  |  |
|  |  |  |  | *M*(Anxiety) = 6.73, *SD* = 5.55 | | *M*(Anxiety) = 3.18, *SD* = 3.12 | <.001 | *d* = 0.702 |  |  |
| Azcue et al. (2022) | Depression; Anxiety; suicidal ideation | *n* = 73 | ME/CFS patients without Long COVID (*n* = 42) | *M*(Depression) = 6.97, *SD* = 3.97 | | *M*(Depression) = 9.35, *SD* = 3.21 | <.01 | – |  |  |
|  |  |  |  | *M*(Anxiety Trait) = 17.08, *SD* = 14.04 | | *M*(Anxiety Trait) = 26.97, *SD* = 17.12 | <.01 | – |  |  |
|  |  |  |  | *M*(Anxiety State) = 27.12, *SD* = 14.12 | | *M*(Anxiety State) = 31.42, *SD* = 15.32 | non sig | – |  |  |
|  |  |  |  | *M*(suicidal ideation) = 0.24, *SD* = 0.66 | | *M*(suicidal ideation) = 0.834, *SD* = 1.32 | <.01 | – |  |  |
| Becker et al. (2021) | Anxiety; Depression; PTSD; perceived stress during illness; resilience | *n* = 63 | Patients after COVID-19 without Long COVID (*n* = 27) | *M*(Anxiety) = 4.37, *SD* = 4.12 | | *M*(Anxiety) = 3.19, *SD* = 2.54 | .174 | – |  |  |
|  |  |  |  | *M*(Depression) = 2.79, *SD* = 3.63 | | *M*(Depression) = 1.93, *SD* = 2.11 | .258 | – |  |  |
|  |  |  |  | *M*(PTSD) = -2.34, *SD* = 1.68 | | *M*(PTSD) = -2.85, *SD* = 1.49 | .183 | – |  |  |
|  |  |  |  | *M*(Perceived stress during illness) = 21.91, *SD* = 8.04 | | *M*(Perceived stress during illness) = 21.86, *SD* = 6.51 | .183 | – |  |  |
|  |  |  |  | *M*(Resilience) = 31.32, *SD* = 6.45 | | *M*(Resilience) = 32.31, *SD* = 5.16 | .494 | – |  |  |
| Bellan et al. (2022) | Anxiety symptoms; depressive symptoms | *n* = 93 | Patients after COVID-19 without Long COVID (*n* = 154) | No. and % of patients with anxiety: 20 (23.0%) | | No. and % of patients with anxiety: 16 (10.5%) | .01 | – |  |  |
|  |  |  |  | No. and % of patients with depression: 28 (32.2%) | | No. and % of patients with depression: 24 (15.8%) | .005 | – |  |  |
| Benoit-Piau et al. (2023) | Pre-existing mental health disorders; psychological  distress | *n* = 622  Long COVID & FSS < 5: *n* = 387 (32.5%)  Long COVID & FSS ≥ 5: *n* = 181 (15.25) | Patients after COVID-19 without Long COVID (*n* = 727)  No Long COVID & FSS < 5: *n* = 571 (47.9%)  No Long COVID & FSS ≥ 5: 53 (4.4%) | No. and % of patients with pre-existing mental health disorders: | | No. and % of patients with pre-existing mental health disorders: | <.001 | – |  |  |
|  |  |  |  | Long COVID & FSS < 5: 82 (21.4%) | | No Long COVID & FSS < 5: 77 (13.7%) |  |  |  |  |
|  |  |  |  | Long COVID & FSS ≥ 5: 80 (44.9%) | | No Long COVID & FSS ≥ 5: 21 (40.4%) |  |  |  |  |
|  |  |  |  | No. and % of patients with psychological distress: 36 (6.1%) | | No. and % of patients with psychological distress: 8 (1.2%) | – | – |  |  |
| Buonsenso et al. (2023) | Stigma | *n* = 40 | Patients after COVID-19 without Long COVID (*n* = 184) | No. and % of patients for each item on a 5-point Likert scale from 1 (never) to 5 (always): | | No. and % of patients for each item on a 5-point Likert scale from 1 (never) to 5 (always): |  |  |  |  |
|  |  |  |  | I felt embarrassed due to Long COVID:  1: 29 (72.5%)  2: 5 (12.5%)  3: 5 (12.5%)  4: 1 (2.5%)  5: 0 (0.0%) | | I felt embarrassed due to Long COVID:  1: 164 (89.1%)  2: 11 (6.0%)  3: 5 (2.7%)  4: 3 (1.6%)  5: 1 (0.5%) | .035 | – |  |  |
|  |  |  |  | I felt embarrassed due to my physical limitation:  1: 25 (62.5%)  2: 7 (17.5%)  3: 6 (15.0%)  4: 1 (2.5%)  5: 1 (2.5%) | | I felt embarrassed due to my physical limitation:  1: 169 (91.8%)  2: 11 (6.0%)  3: 2 (1.1%)  4: 2 (1.1%)  5: 0 (0.0%) | <.001 | – |  |  |
|  |  |  |  | I felt less valued due to Long COVID:  1: 29 (72.5%)  2: 7 (17.5%)  3: 2 (5.0%)  4: 2 (5.0%) | | I felt less valued due to Long COVID:  1: 170 (92.3%)  2: 8 (4.4%)  3: 4 (2.2%)  4: 2 (1.1%) | .003 | – |  |  |
|  |  |  |  | I felt different due to Long COVID:  1: 30 (75.0%)  2: 6 (15.0%)  3: 2 (5.0%)  4: 1 (2.5%)  5: 1 (2.5%) | | I felt different due to Long COVID:  1: 169 (91.8%)  2: 7 (3.8%)  3: 5 (2.7%)  4: 2 (1.1%)  5: 1 (0.5%) | .033 | – |  |  |
|  |  |  |  | Due to Long COVID, people are uncomfortable  with me:  1: 35 (87.5%)  2: 2 (5.0%)  3: 3 (7.5%)  4: 0 (0.0%)  5: 0 (0.0%) | | Due to Long COVID, people are uncomfortable with me:  1: 168 (91.3%)  2: 11 (6.0%)  3: 2 (1.1%)  4: 1 (0.5%)  5: 2 (1.1%) | .15 | – |  |  |
|  |  |  |  | Due to Long COVID, people are rude to me:  1: 36 (90.0%)  2: 1 (2.5%)  3: 1 (2.5%)  4: 2 (5.0%)  5: 0 (0.0%) | | Due to Long COVID, people are rude to me:  1: 172 (93.5%)  2: 6 (3.3%)  3: 2 (1.1%)  4: 2 (1.1%)  5: 2 (1.1%) | .43 | – |  |  |
|  |  |  |  | Close people did not want spend time with me anymore when they knew I may have Long COVID:  1: 39 (97.5%)  2: 0 (0.0%)  3: 1 (2.5%)  5: 0 (0.0%) | | Close people did not want spend time with me anymore when they knew I may have Long COVID:  1: 176 (95.7%)  2: 7 (3.8%)  3: 0 (0.0%)  5: 1 (0.5%) | .096 | – |  |  |
|  |  |  |  | People thought I was lying about my health:  1: 35 (87.5%)  2: 1 (2.5%)  3: 2 (5.0%)  4: 2 (5.0%)  5: 0 (0.0%) | | People thought I was lying about my health:  1: 177 (96.2%)  2: 5 (2.7%)  3: 1 (0.5%)  4: 0 (0.0%)  5: 1 (0.5%) | .006 | – |  |  |
|  |  |  |  | People stopped respecting me due to Long COVID:  1: 37 (92.5%)  2: 0 (0.0%)  3: 3 (7.5%)  5: 0 (0.0%) | | People stopped respecting me due to Long COVID:  1: 177 (96.2%)  2: 5 (2.7%)  3: 1 (0.5%)  5: 1 (0.5%) | .017 | – |  |  |
|  |  |  |  | People do not think Long COVID is a real disease:  1: 25 (62.5%)  2: 6 (15.0%)  3: 3 (7.5%)  4: 2 (5.0%)  5: 4 (10.0%) | | People do not think Long COVID is a real disease:  1: 153 (83.2%)  2: 11 (6.0%)  3: 14 (7.7%)  4: 3 (1.6%)  5: 3 (1.6%) | .007 | – |  |  |
|  |  |  |  | People think having Long COVID is a sign  of weakness:  1: 31 (77.5%)  2: 2 (5.0%)  3: 3 (7.5%)  4: 3 (7.5%)  5: 1 (2.5%) | | People think having Long COVID is a sign  of weakness:  1: 168 (91.3%)  2: 7 (3.8%)  3: 3 (1.6%)  4: 1 (0.5%)  5: 5 (2.7%) | .008 | – |  |  |
|  |  |  |  | I feel I can be judged negatively due to Long COVID:  1: 30 (75.0%)  2: 2 (5.0%)  3: 6 (15.0%)  4: 1 (2.5%)  5: 1 (2.5%) | | I feel I can be judged negatively due to Long COVID:  1: 174 (94.6%)  2: 6 (3.3%)  3: 2 (1.1%)  4: 0 (0.0%)  5: 2 (1.1%) | <.001 | – |  |  |
| Cai et al. (2023) | Anxiety; Depression; PTSD; Anxiety/Depression | *n* = 450 at 6MFU | At 6MFU :  Patients after COVID-19 without Long COVID (*n* = 75)  Healthy controls without a history of COVID-19 (*n* = 979) |  | | Patients after COVID-19 without Long COVID: |  |  |  |  |
|  |  |  |  | No. and % of patients with Anxiety: 183 (40.67%) | | No. and % of patients with Anxiety: 6 (8%) | <.001 | – |  |  |
|  |  |  |  | No. and % of patients with Depression: 207 (46%) | | No. and % of patients with Depression: 5 (6.67%) | <.001 | – |  |  |
|  |  |  |  | No. and % of patients with PTSD: 87 (19.33%) | | No. and % of patients with PTSD: 1 (1.33%) | <.001 | – |  |  |
|  |  |  |  | No. and % of patients with Anxiety/Depression: 240 (53.33%) | | No. and % of patients with Anxiety/Depression: 8 (10.67%) | <.001 | – |  |  |
|  |  |  |  |  | | Healthy controls without a history of COVID-19: |  |  |  |  |
|  |  |  |  |  | | No. and % of patients with Anxiety: 370 (37.79%) | .300 | – |  |  |
|  |  |  |  |  |  | No. and % of patients with Depression: 313 (31.97%) | <.001 | – |  |  |
|  |  |  |  |  |  | No. and % of patients with PTSD: 111 (11.34%) | <.001 | – |  |  |
|  |  |  |  |  |  | No. and % of patients with Anxiety/Depression: 426 (43.51%) | .001 | – |  |  |
| Calvache-Mateo et al. (2023) | Physical activity; central sensitisation; kinesiophobia; catastrophising; Depression; Anxiety; stress; fear avoidance | *n* = 55 | Patients after COVID-19 without Long COVID (*n* = 57)  Healthy controls without a history of COVID-19 (*n* = 58) |  | | Patients after COVID-19 without Long COVID: |  |  |  |  |
|  |  |  |  | *M*(physical activity) = 812.53, *SD* = 667.98 | | *M*(physical activity) = 3138.82, *SD* = 958.98 | ≤.05 | – |  |  |
|  |  |  |  | *M*(central sensitisation) = 54.53, *SD* = 17.10 | | *M*(central sensitisation) = 18.81, *SD* = 16.24 | ≤.05 | – |  |  |
|  |  |  |  | *M*(kinesiophobia) = 25.06, *SD* = 6.67 | | *M*(kinesiophobia) = 13.81, *SD* = 7.20 | ≤.05 | – |  |  |
|  |  |  |  | *M*(catastrophising) = 22.09, *SD* = 10.64 | | *M*(catastrophising) = 6.16, *SD* = 8.89 | ≤.05 | – |  |  |
|  |  |  |  | *M*(depression) = 7.02, *SD* = 5.41 | | *M*(depression) = 2.14, *SD* = 3.76 | ≤.05 | – |  |  |
|  |  |  |  | *M*(anxiety) = 9.11, *SD* = 3.98 | | *M*(anxiety) = 2.54, *SD* = 3.45 | ≤.05 | – |  |  |
|  |  |  |  | *M*(stress) = 9.26, *SD* = 5.27 | | *M*(stress) = 4.79, *SD* = 5.12 | ≤.05 | – |  |  |
|  |  |  |  | *M*(fear avoidance) = 28.86, *SD* = 13.76 | | *M*(fear avoidance) = 7.98, *SD* = 9.34 | ≤.05 | – |  |  |
|  |  |  |  |  | | Healthy controls without a history of COVID-19: |  |  |  |  |
|  |  |  |  |  |  | *M*(physical activity) = 3250.17, *SD* = 1253.65 | ≤.05 | – |  |  |
|  |  |  |  |  |  | *M*(central sensitisation) = 17.69, *SD* = 14.30 | ≤.05 | – |  |  |
|  |  |  |  |  |  | *M*(kinesiophobia) = 16.55, *SD* = 9.44 | ≤.05 | – |  |  |
|  |  |  |  |  | | *M*(catastrophising) = 6.95, *SD* = 7.71 | ≤.05 | – |  |  |
|  |  |  |  |  |  | *M*(depression) = 2.76, *SD* = 4.00 | ≤.05 | – |  |  |
|  |  |  |  |  |  | *M*(anxiety) = 2.60, *SD* = 4.11 | ≤.05 | – |  |  |
|  |  |  |  |  |  | *M*(stress) = 4.35, *SD* = 4.41 | ≤.05 | – |  |  |
|  |  |  |  |  |  | *M*(fear avoidance) = 10.43, *SD* = 12.64 | ≤.05 | – |  |  |
| Carazo et al. (2022) | Psychological distress | *n* = 2,836 | Healthy controls without a history of COVID-19 (*n* = 4,390) | No. and % of patients with psychological distress score | | No. and % of patients with psychological distress score | .23 | – | Global score (initially comparing hospitalised Long COVID, non-hospitalised Long COVID and HC) |  |
|  |  |  |  | < 7: 1,090 (38.4%) | | < 7: 1,972 (44.9%) |  |  |  |  |
|  |  |  |  | 7-12: 1,070 (37.7%) | | 7-12: 1,779 (40.5%) |  |  |  |  |
|  |  |  |  | > 12: 1,090 (38.4%) | | > 12: 1,972 (44.9%) |  |  |  |  |
| Carter et al. (2022) | Anger; Depression;  total mood disturbance; physical activity; Depression medications; walking self-efficacy | *n* = 17 | Healthy controls without a history of COVID-19 (*n* = 15) | *M*(anger) = 6, *SD* = 5 | | *M*(anger) = 3, *SD* = 2 | .132 | – |  |  |
|  |  |  |  | $\tilde{X}$(depression) = 3 (IQR 11) | | $\tilde{X}$(depression) = 3 (IQR 3) | .262 | – |  |  |
|  |  |  |  | *M*(total mood disturbance) = 19, *SD* = 24 | | *M*(total mood disturbance) = -1, *SD* = 12 | .005 | 1.0 |  |  |
|  |  |  |  | $\tilde{X}$(physical activity) = 21 (IQR 18) | | $\tilde{X}$(physical activity) = 36 (IQR 22) | .004 | 0.27 |  |  |
|  |  |  |  | % of patients with Depression medications: 6.35% | | % of patients with Depression medications: 1.7% | .051 | – |  |  |
|  |  |  |  | $\tilde{X}$(walking self-efficacy) = 81 (IQR 44) | | $\tilde{X}$(walking self-efficacy) = 95 (IQR 18) | .274 | – |  |  |
| Chen et al. (2023b) | Depression and/or Anxiety | *n* = 904 | Patients after COVID-19 without Long COVID (*n* = 499) | No. and % of patients with pre-existing Depression and/or Anxiety: 165 (18.2%) | | No. and % of patients with pre-existing Depression and/or Anxiety: 64 (12.9%) | .01 | – |  |  |
| Cuschieri et al. (2023) | Anxiety; Depression | *n* = 50 | Patients after COVID-19 without Long COVID (*n* = 72)  Healthy controls without a history of COVID-19 (*n* = 489) |  | | Patients after COVID-19 without Long COVID: |  |  | Exact numbers not reported |  |
|  |  |  |  | Moderate and severe depression scores | | Moderate and severe depression scores | .001 | – |  |  |
|  |  |  |  |  | | Healthy controls without a history of COVID-19: |  |  |  |  |
|  |  |  |  | Moderate and severe depression scores | | Moderate and severe depression scores | <.01 | – |  |  |
|  |  |  |  | Anxiety | | Anxiety | <.01 | – |  |  |
| de Oliviera et al. (2022) | Depression and anxiety (questionnaire on Long COVID symptoms); depression and anxiety (domain of the EQ-5D-3L) | *n* = 369 | Healthy controls without a history of COVID-19 (*n* = 70) | No. and % of patients not anxious or depressed: 162 (44.9%) | | No. and % of patients not anxious or depressed: 53 (79.1%) | .000 | – |  |  |
|  |  |  |  | No. and % of patients moderately anxious or depressed: 167 (46.2%) | | No. and % of patients moderately anxious or depressed: 14 (20.9%) |  |  |  |  |
|  |  |  |  | No. and % of patients extremely anxious or depressed: 32 (8.9%) | | No. and % of patients extremely anxious or depressed: 0 (0%) |  |  |  |  |
| Delgado-Alonso et al. (2022a) | Personality (emotional stability, extraversion, openness, conscientiousness, agreeableness) | *n* = 93 | Matched healthy controls without a history of COVID-19 (*n* = 93) | *M*(agreeableness) = 47.94, *SD* = 22.47 | | *M*(agreeableness) = 55.66, *SD* = 22.40 | .02 | – |  |  |
|  |  |  |  | *M*(conscientiousness) = 50.33, *SD* = 29.05 | | *M*(conscientiousness) = 58.38, *SD* = 26.82 | .051 | – |  |  |
|  |  |  |  | *M*(extraversion) = 45.04, *SD* = 30.79 | | *M*(extraversion) = 56.38, *SD* = 29.78 | .012 | – |  |  |
|  |  |  |  | *M*(emotional stability) = 34.15, *SD* = 27.0 | | *M*(emotional stability) = 45.24, *SD* = 27.0 | .006 (sign. after FDR correction) | – |  |  |
|  |  |  |  | *M*(openness) = 40.02, *SD* = 31.45 | | *M*(openness) = 48.62, *SD* = 28.09 | .051 | – |  |  |
| Durstenfeld et al. (2023) | Anxiety; Depression; physical activity | *n* = 476 | Healthy controls without a history of COVID-19 (*n* = 1,004) | *M*(Anxiety) = 5.04, *SD* = 4.66 | | *M*(Anxiety) = 3.08, *SD* = 3.60 | <.0001 | – |  |  |
|  |  |  |  | *M*(Depression) = 5.85, *SD* = 4.91 | | *M*(Depression) = 3.31, *SD* = 3.74 | <.0001 | – |  |  |
|  |  |  |  | *M*(physical activity) = 2.20, *SD* = 1.73 | | *M*(physical activity) = 2.62, *SD* = 1.84 | .0000 | – |  |  |
| Fernández-Alonso et al. (2023) | Depression; Anxiety | *n* = 32 at 3MFU  *n* = 54 at 6MFU | Patients after COVID-19 without Long COVID (*n* = 65 at 3MFU, *n* = 43 at 6MFU) | 3MFU: | | 3MFU: |  |  |  |  |
|  |  |  |  | *M*(Depression) = 5.13, *SD* = 4.92 | | *M*(Depression) = 3.20, *SD* = 3.30 | .006 | – |  |  |
|  |  |  |  | *M*(Anxiety) = 6.50, *SD* = 4.97 | | *M*(Anxiety) = 5.12, *SD* = 4.28 | .154 | – |  |  |
|  |  |  |  | 6MFU: | | 6MFU: |  |  |  |  |
|  |  |  |  | *M*(Depression) = 4.87, *SD* = 4.33 | | *M*(Depression) = 2.53, *SD* = 3.11 | .051 | – |  |  |
|  |  |  |  | *M*(Anxiety) = 6.96, *SD* = 5.02 | | *M*(Anxiety) = 3.84, *SD* = 3.13 | .002 | – |  |  |
| Freda et al. (2024) | Maternal health anxiety; fear of SARS-CoV-2 infection | *n* = 29 | Patients after COVID-19 without Long COVID (*n* = 76)  Healthy controls without a history of COVID-19 (*n* = 93) |  | | Patients after COVID-19 without Long COVID: |  |  | Only values of significant results reported |  |
|  |  |  |  | *M*(maternal health anxiety) = 17.17, *SD* = 8.60 | | *M*(maternal health anxiety) = 12.17, *SD* = 6.14 | .001 | mean difference = 5.01, 95% CI [10.77-13.58] |  |  |
|  |  |  |  | *M*(fear of SARS-CoV-2 infection) = 7.24, *SD* = 9.47 | | *M*(fear of SARS-CoV-2 infection) = 6.11, *SD* = 7.79 | .21 | mean difference = 0.26, 95% CI [-0.08-0.62] |  |  |
|  |  |  |  |  | | Healthy controls without a history of COVID-19: |  |  |  |  |
|  |  |  |  |  |  | *M*(maternal health anxiety) = 10.76, *SD* = 5.07 | <.001 | mean difference = 6.41, 95% CI [9.72-11.81] |  |  |
|  |  |  |  |  |  | *M*(fear of SARS-CoV-2 infection) = 7.81, *SD* = 7.32 | .03 | mean difference = 0.36, 95% CI [0.02-0.69] |  |  |
| Frontera et al. (2021) | Anxiety; Depression; history of Depression, Anxiety, other mood disorder, thought disorder; Depression and Anxiety as Long COVID symptoms | *n* = 19 | Patients after COVID-19 without Long COVID (*n* = 57)  Healthy controls without a history of COVID-19 (*n* = 923) |  | | Patients after COVID-19 without Long COVID: |  |  | Individual comparisons not reported, only global comparisons between all 3 groups |  |
|  |  |  |  | $\tilde{X}$(Anxiety) = 56.8 (IQR 51.4-62.6) | | $\tilde{X}$(Anxiety) = 53.3 (IQR 45.1-56.8) | .007 | – |  |  |
|  |  |  |  | $\tilde{X}$(Depression) = 51.3 (IQR 46.8-56.7) | | $\tilde{X}$(Depression) = 46.8 (IQR 36.9-53.2) | .113 | – |  |  |
|  |  |  |  | No. and % of patients with a history of Depression: 11 (58%) | | No. and % of patients with a history of Depression: 17 (30%) | .013 | – |  |  |
|  |  |  |  | No. and % of patients with a history of  Anxiety: 8 (42%) | | No. and % of patients with a history of Anxiety: 18 (32%) | .562 | – |  |  |
|  |  |  |  | No. and % of patients with a history of other mood disorder: 5 (26%) | | No. and % of patients with a history of other mood disorder: 1 (2%) | <.001 | – |  |  |
|  |  |  |  | No. and % of patients with a history of thought disorder: 1 (5%) | | No. and % of patients with a history of thought disorder: 0 (0%) | .011 | – |  |  |
|  |  |  |  | No. and % of patients with Depression symptom: 8 (42%) | | No. and % of patients with Depression symptom: 15 (26%) | .305 | – |  |  |
|  |  |  |  | No. and % of patients with Anxiety symptom: 10 (53%) | | No. and % of patients with Anxiety symptom: 21 (37%) | .025 | – |  |  |
|  |  |  |  |  | | Healthy controls without a history of COVID-19: |  |  |  |  |
|  |  |  |  |  |  | $\tilde{X}$(Anxiety) = 51.4 (IQR 45.9-57.6) |  |  |  |  |
|  |  |  |  |  |  | $\tilde{X}$(Depression) = 47.9 (IQR 43.1-53.6) |  |  |  |  |
|  |  |  |  |  |  | No. and % of patients with a history of Depression: 252 (27%) |  |  |  |  |
|  |  |  |  |  |  | No. and % of patients with a history of Anxiety: 283 (31%) |  |  |  |  |
|  |  |  |  |  |  | No. and % of patients with a history of other mood disorder: 47 (5%) |  |  |  |  |
|  |  |  |  |  |  | No. and % of patients with a history of thought disorder: 4 (0.4%) |  |  |  |  |
|  |  |  |  |  |  | No. and % of patients with Depression symptom: 243 (26%) |  |  |  |  |
|  |  |  |  |  |  | No. and % of patients with Anxiety symptom: 258 (28%) |  |  |  |  |
| Fry et al. (2023) | Anxiety; Anxiety related disorders; Depression; Mania; Paranoia; Schizophrenia; Borderline features; Antisocial features; Alcohol problems; Drug problems; Aggression; Suicidal ideation; Nonsupport; Stress; Treatment rejection; Dominance; Warmth | *n* = 201 | Patients with post-concussion syndrome (*n* = 102) | *M*(Anxiety) = 59.65, *SD* = 12.46 | | *M*(Anxiety) = 63.36, *SD* = 15.17 | non sig | *d* = 0.35 |  |  |
|  |  |  |  | *M*(Anxiety related disorders) = 57.61, *SD* = 13.55 | | *M*(Anxiety related disorders) = 61.68, *SD* = 15.69 | non sig | *d* = 0.3 |  |  |
|  |  |  |  | *M*(Depression) = 64.46, *SD* = 13.36 | | *M*(Depression) = 68.31, *SD* = 15.69 | non sig | *d* = 0.27 |  |  |
|  |  |  |  | *M*(Mania) = 46.23, *SD* = 9.20 | | *M*(Mania) = 48.12, *SD* = 10.18 | non sig | *d* = 0.19 |  |  |
|  |  |  |  | *M*(Paranoia) = 49.16, *SD* = 9.55 | | *M*(Paranoia) = 54.64, *SD* = 13.01 | <.01 | *d* = 0.50 |  |  |
|  |  |  |  | *M*(Schizophrenia) = 56.74, *SD* = 10.47 | | *M*(Schizophrenia) = 61.18, *SD* = 14.20 | non sig | *d* = 0.37 |  |  |
|  |  |  |  | *M*(Borderline feat ures) = 52.83, *SD* = 10.28 | | *M*(Borderline feat ures) = 58.09, *SD* = 12.90 | non sig | *d* = 0.46 |  |  |
|  |  |  |  | *M*(Antisocial features) = 46.76, *SD* = 7.49 | | *M*(Antisocial features) = 50.02, *SD* = 10.43 | non sig | *d* = 0.38 |  |  |
|  |  |  |  | *M*(Alcohol problems) = 46.71, *SD* = 7.46 | | *M*(Alcohol problems) = 48.90, *SD* = 9.72 | non sig | *d* = 0.26 |  |  |
|  |  |  |  | *M*(Drug problems) = 48.37, *SD* = 6.69 | | *M*(Drug problems) = 52.34, *SD* = 11.54 | non sig | *d* = 0.46 |  |  |
|  |  |  |  | *M*(Aggression) = 45.33, *SD* = 8.71 | | *M*(Aggression) = 49.32, *SD* = 11.67 | non sig | *d* = 0.47 |  |  |
|  |  |  |  | *M*(Suicidal ideation) = 52.16, *SD* = 11.50 | | *M*(Suicidal ideation) = 54.75, *SD* = 14.78 | non sig | *d* = 0.20 |  |  |
|  |  |  |  | *M*(Nonsupport) = 51.20, *SD* = 10.93 | | *M*(Nonsupport) = 54.16, *SD* = 12.22 | non sig | *d* = 0.38 |  |  |
|  |  |  |  | *M*(Stress) = 52.46, *SD* = 9.94 | | *M*(Stress) = 56.44, *SD* = 11.77 | non sig | *d* = 0.26 |  |  |
|  |  |  |  | *M*(Treatment rejection) = 48.94, *SD* = 9.56 | | *M*(Treatment rejection) = 44.83, *SD* = 11.40 | non sig | *d* = 0.36 |  |  |
|  |  |  |  | *M*(Dominance) = 48.04, *SD* = 10.75 | | *M*(Dominance) = 46.02, *SD* = 10.28 | non sig | *d* = 0.19 |  |  |
|  |  |  |  | *M*(Warmth) = 50.03, *SD* = 10.25 | | *M*(Warmth) = 46.07, *SD* = 11.30) | non sig | *d* = 0.31 |  |  |
| Gaspar et al. (2023) | Pre-existing Depression/Anxiety | At 3MFU: *n* = 101  At 6MFU: *n* = 73  At 9MFU: *n* = 59 | Patients after COVID-19 without Long COVID  At 3MFU: *n* = 51  At 6MFU: *n* = 44  At 9MFU: *n* = 51 | No. and % of patients with pre-existing Depression/Anxiety at 3MFU: 21 (20.8%) | | No. and % of patients with pre-existing Depression/Anxiety at 3MFU: 9 (17.7%) | .646 | – |  |  |
|  |  |  |  | No. and % of patients with pre-existing Depression/Anxiety at 6MFU: 15 (20.6%) | | No. and % of patients with pre-existing Depression/Anxiety at 6MFU: 10 (22.7%) | .781 | – |  |  |
|  |  |  |  | No. and % of patients with pre-existing Depression/Anxiety at 9MFU: 11 (18.6%) | | No. and % of patients with pre-existing Depression/Anxiety at 9MFU: 11 (21.6%) | .702 | – |  |  |
| Goodman et al. (2023) | Depression; Anxiety; PTSD; suicide ideation | *n* = 126 | Patients after COVID-19 without Long COVID (*n* = 495) | % of patients with Depression: 28.33% | | % of patients with Depression: 14.97% | .001 | – |  |  |
|  |  |  |  | % of patients with Anxiety: 47.06% | | % of patients with Anxiety: 19.17% | <.001 | – |  |  |
|  |  |  |  | % of patients with PTSD: 38.70% | | % of patients with PTSD: 19.0% | <.001 | – |  |  |
|  |  |  |  | % of patients with positive suicide ideation screen: 21.19% | | % of patients with positive suicide ideation screen: 11.43% | .006 | – |  |  |
| Haider et al. (2023) | Depression; Anxiety; Catastrophising; Kinesiophobia; Multisensory sensitivity/ Somatosensory amplification | *n* = 203 | Patients with fibromyalgia syndrome (FMS; *n* = 99) or chronic fatigue syndrome (CFS; *n* = 87) |  | | FMS patients: |  |  |  |  |
|  |  |  |  | *M*(Depression PHQ-2) = 2.2, *SD* = 1.7 | | *M*(Depression PHQ-2) = 2.3, *SD* = 1.4 | non sig | – |  |  |
|  |  |  |  | *M*(Depression HADS) = 8.8, *SD* = 4.1 | | *M*(Depression HADS) = 9.0, *SD* = 3.4 | non sig | – |  |  |
|  |  |  |  | *M*(Anxiety HADS) = 9.6, *SD* = 4.1 | | *M*(Anxiety HADS) = 9.6, *SD* = 3.5 | non sig | – |  |  |
|  |  |  |  | *M*(Catastrophising) = 12.4, *SD* = 8.1 | | *M*(Catastrophising) = 14.8, *SD* = 7.4 | .052 | – |  |  |
|  |  |  |  | *M*(Kinesiophobia) = 25.0, *SD* = 6.8 | | *M*(Kinesiophobia) = 27.5, *SD* = 5.9 | <.01 | – |  |  |
|  |  |  |  | *M*(Multisensory sensitivity/ Somatosensory amplification) = 20.3, *SD* = 5.4 | | *M*(Multisensory sensitivity/ Somatosensory amplification) = 23.5, *SD* = 5.6 | <.001 | – |  |  |
|  |  |  |  |  | | CFS patients: |  |  |  |  |
|  |  |  |  |  |  | *M*(Depression PHQ-2) = 2.3, *SD* = 1.5 | non sig | – |  |  |
|  |  |  |  |  |  | *M*(Depression HADS) = 8.5, *SD* = 3.4 | non sig | – |  |  |
|  |  |  |  |  | | *M*(Anxiety HADS) = 9.0, *SD* = 3.6 | non sig | – |  |  |
|  |  |  |  |  |  | *M*(Catastrophising) = 15.0, *SD* = 6.3 | <.01 | – |  |  |
|  |  |  |  |  |  | *M*(Kinesiophobia) = 26.6, *SD* = 5.9 | non sig | – |  |  |
|  |  |  |  |  |  | *M*(Multisensory sensitivity/ Somatosensory amplification) = 21.7, *SD* = 5.4 | non sig | – |  |  |
| Hedberg et al. (2023) | Pre-existing mental health disorders (comorbidities) | Non-hospitalised: *n* = 1,963  Hospitalised: *n* = 693  ICU-treated: *n* = 410 | Non-hospitalised patients after COVID-19 without Long COVID (*n* = 189,496)  Hospitalised patients after COVID-19 without Long COVID (*n* = 11,377)  ICU-treated patients after COVID-19 without Long COVID (*n* = 866) | No. and % of patients with mental health disorder: 720 (36.7%) | | No. and % of patients with mental health disorder: 36,579 (19.3%) | – | – |  |  |
|  |  |  |  | No. and % of patients with mental health disorder: 195 (28.1%) | | No. and % of patients with mental health disorder: 2,322 (20.4%) | – | – |  |  |
|  |  |  |  | No. and % of patients with mental health disorder: 89 (21.7%) | | No. and % of patients with mental health disorder: 154 (17.8%) | – | – |  |  |
| Heine et al. (2023) | Depression; Anxiety | *n* = 47 | Matched healthy controls without a history of COVID-19 (*n* = 47) | *M*(Depression) = 17.0, *SD* = 7.8 | | *M*(Depression) = 4.8, *SD* = 5.4 | <.0001 | g [95% CI] = 1.11 [0.73-1.51] |  |  |
|  |  |  |  | *M*(Anxiety) = 14.6, *SD* = 7.5 | | *M*(Anxiety) = 3.9, *SD* = 4.8 | <.0001 | g = 1.06 [0.68-1.45] |  |  |
| Huang et al. (2022) | Depression; Anxiety; PTSD | *n* = 650 | Patients after COVID-19 without Long COVID at 2 years (*n* = 540) | No. and % of patients with Depression: 70/649 (11%) | | No. and % of patients with Depression: 5 (1%) | <.0001 | OR: 11.43, 95% CI [4.55-28.72] |  |  |
|  |  |  |  | No. and % of patients with Anxiety: 83 (13%) | | No. and % of patients with Anxiety: 15/536 (3%) | <.0001 | OR: 4.63, 95% CI [2.53-8.50] |  |  |
|  |  |  |  | No. and % of patients PTSD: 27 (4%) | | No. and % of patients with PTSD: 0/538 (0%) | non sig | – |  |  |
| Jacobs et al. (2023) | Pre-existing Depression/Anxiety | *n* = 518 | Patients after COVID-19 without Long COVID (*n* = 706) | No. and % of patients with pre-existing Depression/Anxiety: 71 (13.7%) | | No. and % of patients with pre-existing Depression/Anxiety: 56 (7.9%) | .001 | – |  |  |
| Kim et al. (2022) | Anxiety/Depression; PTSD | *n* = 129 | Patients after COVID-19 without Long COVID (*n* = 41) | No. and % of patients with Anxiety/Depression: 54 (41.86%) | | No. and % of patients with Anxiety/Depression: 6 (14.63%) | .015 | – |  |  |
|  |  |  |  | $\tilde{X}$(PTSD) = 6 (IQR 2.0-14.0) | | $\tilde{X}$(PTSD) = 2 (IQR 0.0-5.0) | <.001 | – |  |  |
| König et al. (2023) | Neuroticism; Life events; Resilience | *n* = 49 | Patients after COVID-19 without Long COVID (*n* = 93) | *M*(Neuroticism) = 5.2, *SD* = 4.0 | | *M*(Neuroticism) = 2.7, *SD* = 2.9 | <.05 | OR: 1.24, 95% CI [1.11-1.38] |  |  |
|  |  |  |  | *M*(Life events past 12 months) = 1.4, *SD* = 1.6 | | *M*(Life events past 12 months) = 0.9, *SD* = 1.2 | <.05 | OR: 1.32, 95% CI [1.01-1.73] |  |  |
|  |  |  |  | *M*(Life events earlier) = 4.1, *SD* = 2.3 | | *M*(Life events earlier) = 3.7, *SD* = 2.5 | non sig | OR: 1.08, 95% CI [0.93-1.25] |  |  |
|  |  |  |  | *M*(Resilience) = 63.6, *SD* = 15.4 | | *M*(Resilience) = 72.9, *SD* = 11.2 | <.05 | OR: 0.95, 95% CI [0.92-0.98] |  |  |
| Kostev et al. (2022b) | Pre-existing Anxiety disorders; pre-existing reaction to severe stress and adjustment disorders; pre-existing disorders of psychological development | *n* = 114 | Patients after COVID-19 without Long COVID (*n* = 6,454) | No. and % of patients with Anxiety disorders: 6 (5.3%) | | No. and % of patients with Anxiety disorders: 77 (1.2%) | .001 | – |  |  |
|  |  |  |  | No. and % of patients with reaction to severe stress and adjustment disorders: 3 (2.6%) | | No. and % of patients with reaction to severe stress and adjustment disorders: 122 (1.9%) | .566 | – |  |  |
|  |  |  |  | No. and % of patients with disorders of psychological development: 16 (14.0%) | | No. and % of patients with disorders of psychological development: 1,016 (15.7%) | .620 | – |  |  |
| Kozik et al. (2023) | Depression | *n* = 282 | Healthy controls without a history of COVID-19 (*n* = 52) | *M*(Depression) = 10.69, *SD* = 5.57 | | *M*(Depression) = 3.92, *SD* = 2.93 | <.001 | – |  |  |
| Lhuillier et al. (2022) | Depression | Long COVID: *n* = 366  Post-acute COVID-19 Respiratory Sequelae: *n* = 206  Post-acute COVID-19 Central Nervous System Sequelae: *n* = 134  Post-acute COVID-19 Fatigue Sequelae: *n* = 108  Post-acute COVID-19 Muscular Sequelae: *n* = 34 | Patients after COVID-19 without Long COVID (*n* = 853)  Patients after COVID-19 without Post-acute COVID-19 Respiratory Sequelae (*n* = 1,074)  Patients after COVID-19 without Post-acute COVID-19 Central Nervous System Sequelae (*n* = 1,146)  Patients after COVID-19 without Post-acute COVID-19 Fatigue Sequelae (*n* = 1,172)  Patients after COVID-19 without Post-acute COVID-19 Muscular Sequelae (*n* = 1,246) | Long COVID:  *M*(Depression) = 4.23, *SD* = 4.71 | | No Long COVID:  *M*(Depression) = 3.06, *SD* = 4.23 | <.001 | – |  |  |
|  |  |  |  | Post-acute COVID-19 Respiratory Sequelae:  *M*(Depression) = 4.82, *SD* = 5.0 | | No Post-acute COVID-19 Respiratory Sequelae:  *M*(Depression) = 3.15, *SD* = 4.3 | <.001 | – |  |  |
|  |  |  |  | Post-acute COVID-19 Central Nervous System Sequelae:  *M*(Depression) = 4.43, *SD* = 5.5 | | No Post-acute COVID-19 Central Nervous System Sequelae:  *M*(Depression) = 3.30, *SD* = 4.3 | .085 | – |  |  |
|  |  |  |  | Post-acute COVID-19 Fatigue Sequelae:  *M*(Depression) = 5.09, *SD* = 4.9 | | No Post-acute COVID-19 Fatigue Sequelae:  *M*(Depression) = 3.26, *SD* = 4.4 | .001 | – |  |  |
|  |  |  |  | Post-acute COVID-19 Muscular Sequelae:  *M*(Depression) = 5.47, *SD* = 6.5 | | No Post-acute COVID-19 Muscular Sequelae:  *M*(Depression) = 3.36, *SD* = 4.4 | .209 | – |  |  |
| Liang et al. (2023) | History of depression; history of anxiety disorders; self-reported depression; self-reported anxiety; perceived stress | *n* = 23 | Healthy controls without a history of COVID-19 (*n* = 24) | | % of patients with a history of depression: 21.7% | % of patients with a history of depression: 12.5% | .400 | – |  |  |
|  |  |  |  |  | % of patients with a history of anxiety: 26.1% | % of patients with a history of anxiety: 8.3% | .106 | – |  |  |
|  |  |  |  |  | *M*(depression) = 53.27, *SD* = 9.11 | *M*(depression) = 45.25, *SD* = 6.86) | .001 | – |  |  |
|  |  |  |  |  | *M*(anxiety) = 56.55, *SD* = 10.45 | *M*(anxiety) = 46.74, *SD* = 8.65 | .001 | – |  |  |
|  |  |  |  |  | *M*(perceived stress) = 57.74, *SD* = 11.28 | *M*(perceived stress) = 46.04, *SD* = 11.56 | .001 | – |  |  |
| Liu et al. (2023) | History of depression; history of anxiety disorder | *n* = 223 | Patients after COVID-19 without Long COVID (*n* = 543) | % of patients with a history of depression: 26.5% | | % of patients with a history of depression: 17.3% | – | OR: 1.72, 95% CI [1.19-2.49] |  |  |
|  |  |  |  | % of patients with a history of anxiety disorder: 32.3% | | % of patients with a history of anxiety disorder: 26.0% | – | OR: 1.36, 95% CI [0.97-1.91] |  |  |
| Magel et al. (2023) | Depression | *n* = 58 at 3MFU  *n* = 47 at 6MFU | At 3MFU:  Patients after COVID-19 without Long COVID (*n* = 29)  At 6MFU:  Patients after COVID-19 without Long COVID (*n* = 32) | *M*(Depression) = 5.8, *SD* = 5.2 | | *M*(Depression) = 0.3, *SD* = 0.8 | <.0001 | – |  |  |
|  |  |  |  | *M*(Depression) = 5.8, *SD* = 5.2 | | *M*(Depression) = 0.3, *SD* = 0.8 | <.0001 | – |  |  |
| Magnavita et al. (2023) | Stress; Anxiety; Depression | *n* = 115 | Patients after COVID-19 without Long COVID (*n* = 49) | *M*(stress) = 0.86, *SD* = 0.37 | | *M*(stress) = 0.89, *SD* = 0.37 | .745 | – |  |  |
|  |  |  |  | *M*(anxiety) = 3.00, *SD* = 2.83 | | *M*(anxiety) = 1.59, *SD* = 2.26 | .001 | – |  |  |
|  |  |  |  | *M*(depression) = 2.07, *SD* = 2.13 | | *M*(depression) = 1.10, *SD* = 1.74 | .002 | – |  |  |
| Margalit et al. (2022) | Recreational physical activity; Depression | *n* = 66 | Patients after COVID-19 without Long COVID (*n* = 75) | *M*(recreational physical activity prior to COVID-19) = 140.15, *SD* = 114.36 | | *M*(recreational physical activity prior to COVID-19) = 144.80, *SD* = 218.91 | .379 | – |  |  |
|  |  |  |  | *M*(recreational physical activity following COVID-19) = 49.28, *SD* = 121.13 | | *M*(recreational physical activity following COVID-19) = 49.18, *SD* = 84.69 | .457 | – |  |  |
|  |  |  |  | *M*(depression) = 9.71, *SD* = 4.53 | | *M*(depression) = 3.32, *SD* = 3.46 | <.001 | – |  |  |
| Marinkovic et al. (2023) | Stress; Anxiety; Depression | *n* = 18 | Healthy controls without a history of COVID-19 (*n* = 20) | *M*(stress) = 19.56, *SD* = 4.57 | | *M*(stress) = 18.80, *SD* = 4.77 | .622 | – |  |  |
|  |  |  |  | *M*(Anxiety) = 4.50, *SD* = 3.13 | | *M*(Anxiety) = 3.28, *SD* = 3.01 | .240 | – |  |  |
|  |  |  |  | *M*(Depression) = 4.33, *SD* = 3.45 | | *M*(Depression) = 2.89, *SD* = 2.89 | .145 | – |  |  |
| Martin et al. (2023) | Depression | *n* = 40 | Healthy controls without a history of COVID-19 (*n* = 40) | *M*(Depression) = 6.85, *SD* = 3.98 | | *M*(Depression) = 2.72, *SD* = 4.55 | .006 | – |  |  |
| Mazza et al. (2022) | Positive psychiatric history; Depression (BDI-13 ≥ 9 = yes); Depression (ZSDS index ≥ 50 = yes) | *n* = 59 (1MFU)  *n* = 32 (3MFU)  *n* = 56 (6MFU)  *n* = 54 (12MFU) | Patients after COVID-19 without Long COVID (*n* = 206 at 1MFU, *n* = 88 at 3MFU, *n* = 132 at 6MFU, *n* = 104 at 12MFU) | 1MFU: | | 1MFU: |  |  |  |  |
|  |  |  |  | No. and % of patients with positive psychiatric history: 41 (18%) | | No. and % of patients with positive psychiatric history: 187 (19%) | <.001 | – |  |  |
|  |  |  |  | No. and % of patients with depression (BDI-13): 41 (18%) | | No. and % of patients with depression (BDI-13): 195 (11%) | <.001 | – |  |  |
|  |  |  |  | No. and % of patients with depression (ZSDS index): 31 (28%) | | No. and % of patients with depression (ZSDS index): 168 (38%) | <.001 | – |  |  |
|  |  |  |  | 3MFU: | | 3MFU: |  |  |  |  |
|  |  |  |  | No. and % of patients with positive psychiatric history: 24 (8%) | | No. and % of patients with positive psychiatric history: 77 (11%) | .188 | – |  |  |
|  |  |  |  | No. and % of patients with depression (BDI-13): 24 (8%) | | No. and % of patients with depression (BDI-13): 82 (6%) | .018 | – |  |  |
|  |  |  |  | No. and % of patients with depression (ZSDS index): 13 (19%) | | No. and % of patients with depression (ZSDS index): 72 (16%) | <.001 | – |  |  |
|  |  |  |  | 6MFU: | | 6MFU: |  |  |  |  |
|  |  |  |  | No. and % of patients with positive psychiatric history: 40 (16%) | | No. and % of patients with positive psychiatric history: 108 (24%) | .238 | – |  |  |
|  |  |  |  | No. and % of patients with depression (BDI-13): 45 (11%) | | No. and % of patients with depression (BDI-13): 123 (9%) | <.001 | – |  |  |
|  |  |  |  | No. and % of patients with depression (ZSDS index): 31 (25%) | | No. and % of patients with depression (ZSDS index): 105 (27%) | <.001 | – |  |  |
|  |  |  |  | 12MFU: | | 12MFU: |  |  |  |  |
|  |  |  |  | No. and % of patients with positive psychiatric history: 33 (21%) | | No. and % of patients with positive psychiatric history: 81 (23%) | .065 | – |  |  |
|  |  |  |  | No. and % of patients with depression (BDI-13): 42 (12%) | | No. and % of patients with depression (BDI-13): 97 (7%) | .019 | – |  |  |
|  |  |  |  | No. and % of patients with depression (ZSDS index): 33 (21%) | | No. and % of patients with depression (ZSDS index): 80 (24%) | .069 | – |  |  |
| Messin et al. (2021) | Anxiety | *n* = 53 | Patients after COVID-19 without Long COVID (*n* = 21) | % of patients not anxious: 49.1% | | % of patients not anxious: 71.4% | .0585 | – |  |  |
|  |  |  |  | % of patients slightly or moderately anxious: 32.1% | | % of patients slightly or moderately anxious: 28.6% |  |  |  |  |
|  |  |  |  | % of patients highly or very highly anxious: 18.9% | | % of patients highly or very highly anxious: 0% |  |  |  |  |
| Meza-Torres et al. (2022) | History of depression; history of anxiety | *n* = 7,623 | Patients after COVID-19 without Long COVID (*n* = 408,882) | % of patients with a history of depression: 36.2% | | % of patients with a history of depression: 22.8% | <.001 | – |  |  |
|  |  |  |  | % of patients with a history of anxiety: 34.8% | | % of patients with a history of anxiety: 23.3% | <.001 |  |  |  |
| Mikuteit et al. (2023) | Depression; Anxiety | *n* = 124 | Healthy controls without a history of COVID-19 (*n* = 24) | *M*(Depression) = 2.76, *SD* = 2.79 | | *M*(Depression) = 0.57, *SD* = 1.08 | .001 | – |  |  |
|  |  |  |  | *M*(Anxiety) = 1.88, *SD* = 2.37 | | *M*(Anxiety) = 0.14, *SD* = 0.47 | <.001 | – |  |  |
| Mora et al. (2023) | Depression; Anxiety | *n* = 94 | Patients after COVID-19 without Long COVID (*n* = 58)  Patients after covert COVID-19 without Long COVID (*n* = 41)  Healthy controls without a history of COVID-19 (*n* = 104) |  | | Patients after COVID-19 without Long COVID: |  |  |  |  |
|  |  |  |  | *M*(Depression) = 0.9, 95% CI [0.6-1.1] | | *M*(Depression) = 0.4, 95% CI [0.1-0.8] | <.05 | – |  |  |
|  |  |  |  | *M*(Anxiety) = 1.3, 95% CI [1.0-1.6] | | *M*(Anxiety) = 0.6, 95% CI [0.2-0.9] | <.05 | – |  |  |
|  |  |  |  |  | | Patients after covert COVID-19 without Long COVID: |  |  |  |  |
|  |  |  |  |  |  | *M*(Depression) = 0.3, 95% CI [0.0-0.7] |  |  |  |  |
|  |  |  |  |  |  | *M*(Anxiety) = 0.5, 95% CI [0.1-0.9] |  |  |  |  |
|  |  |  |  |  |  | Healthy controls without a history of COVID-19: |  |  |  |  |
|  |  |  |  |  | | *M*(Depression) = 0.5, 95% CI [0.2-0.7] |  |  |  |  |
|  |  |  |  |  |  | *M*(Anxiety) = 0.6, 95% CI [0.3-0.9] |  |  |  |  |
| Navas-Otero et al. (2023) | Physical activity; depressed mood | *n* = 68 | Patients after COVID-19 without Long COVID (*n* = 68)  Healthy controls without a history of COVID-19 (*n* = 68) | *M*(physical activity) = 1037.47, *SD* = 960.78  No. and % of patients with depressed mood: 30 (46.1%) | | Patients after COVID-19 without Long COVID:  *M*(physical activity) = 4732.45, *SD* = 3592.17  No. and % of patients with depressed mood: 20 (30.7%) | <.001 | – | Global F-test for depressed mood (comparison between all three groups) |  |
|  |  |  |  |  |  | Healthy controls without a history of COVID-19:  *M*(physical activity) = 7455.03, *SD* = 4736.34  No. and % of patients with depressed mood: 16 (24.6%) | .002 | – |  |  |
| O’Sullivan et al. (2023) | Depression; Anxiety; PTSD | *n* = 34 | Healthy controls without a history of COVID-19 (*n* = 26) | $\tilde{X}$(Depression) = 8 (IQR 5-12) | | $\tilde{X}$(Depression) = 1 (IQR 0-3) | <.001 | – |  |  |
|  |  |  |  | $\tilde{X}$(Anxiety) = 5 (IQR 2-7) | | $\tilde{X}$(Anxiety) = 2 (IQR 0-3) | <.05 | – |  |  |
|  |  |  |  | $\tilde{X}$(PTSD) = 9 (IQR 6-19) | | $\tilde{X}$(PTSD) = 1 (IQR 0-5) | <.001 | – |  |  |
| Peters et al. (2022) | Depressive symptoms; anxiety symptoms | *n* = 1,406 | Patients after COVID-19 without Long COVID (*n* = 524) | % of patients with none/low depression: 80.9% | | % of patients with none/low depression: 95.5% | <.001 | – |  |  |
|  |  |  |  | % of patients with moderate depression: 14.1% | | % of patients with moderate depression: 2.9% |  |  |  |  |
|  |  |  |  | % of patients with strong depression: 5.0% | | % of patients with strong depression: 1.6% |  |  |  |  |
|  |  |  |  | % of patients with depression (≥ 3/6 points): 22.3% | | % of patients with depression (≥ 3/6 points): 5.6% | <.001 | – |  |  |
|  |  |  |  | % of patients with anxiety (≥ 3/6 points): 22.2% | | % of patients with anxiety (≥ 3/6 points): 6.2% | <.001 | – |  |  |
| Phu et al. (2023) | Depression; Anxiety; stress | *n* = 745 | Patients after COVID-19 without Long COVID (*n* = 194) | No. and % of patients with Depression: 97 (13%) | | No. and % of patients with Depression: 7 (3.6%) | – | – |  |  |
|  |  |  |  | No. and % of patients with Anxiety: 171 (23%) | | No. and % of patients with Anxiety: 8 (4.1%) | – | – |  |  |
|  |  |  |  | No. and % of patients with stress: 40 (5.4%) | | No. and % of patients with stress: 2 (1%) | – | – |  |  |
| Pływaczewska-Jakubowska et al. (2022) | physical activity; stress | *n* = 1,013 | Patients after COVID-19 without Long COVID (*n* = 504) | % of patients with regular physical activity: 12.1% | | % of patients with regular physical activity: 52.8% | .42 | – |  |  |
|  |  |  |  | % of patients with stress: 33.3% | | % of patients with stress: 31.6% | .47 | – |  |  |
| Rastogi et al. (2023) | Prior mental health diagnosis; physical activity level not meeting guidelines | *n* = 19,862 | Patients after COVID-19 without Long COVID (*n* = 44,652) | % of patients with prior mental health diagnosis: 34.8% | | % of patients with prior mental health diagnosis: 45.4% | – | – |  |  |
|  |  |  |  | % of patients with physical activity level not meeting guidelines: 27.9% | | % of patients with physical activity level not meeting guidelines: 41.3% | – | – |  |  |
| Roessler et al. (2022) | Anxiety disorders; depression; adjustment disorders | *n* = 157,134 (*n* = 11,950 children; *n* = 145,184 adults) | Matched healthy controls without a history of COVID-19 (*n* = 51,825 children; *n* = 557,287 adults) | Children/adolescents only: | | Children/adolescents only: |  |  |  |  |
|  |  |  |  | Incidence rate of anxiety disorders: IR = 16.70 | | Incidence rate of anxiety disorders: IR = 10.87 | <.01 | – |  |  |
|  |  |  |  | Incidence rate of depression: IR = 12.05 | | Incidence rate of depression: IR = 8.32 | <.01 | – |  |  |
|  |  |  |  | Incidence rate of adjustment disorders: IR = 26.37 | | Incidence rate of adjustment disorders: IR = 15.40 | <.01 | – |  |  |
| Román-Montes et al. (2023) | Anxiety/depression | *n* = 187 | Patients after COVID-19 without Long COVID (*n* = 59) | % of patients with  no anxiety/depression: 42% | | % of patients with  no anxiety/depression: 63% | .02 | – |  |  |
|  |  |  |  | % of patients with  moderate anxiety/depression: 54% | | % of patients with  moderate anxiety/depression: 34% |  |  |  |  |
|  |  |  |  | % of patients with  extreme anxiety/depression: 4% | | % of patients with  extreme anxiety/depression: 3% |  |  |  |  |
| Salve et al. (2023) | Depression | *n* = 77 | Patients after COVID-19 without Long COVID (*n* = 133) | *M*(Depression) = 5.3, *SD* = 2.6 | | *M*(Depression) = 3.6, *SD* = 2.9 | <.001 | – |  |  |
| Shachar-Lavie (2023) | Connection with friends; physical activity; depression and anxiety; avoidance; negative cognitions | *n* = 87 | Healthy controls without a history of COVID-19 (*n* = 101) | *M*(connection with friends) = 0.39, *SD* = 0.63 | | *M*(connection with friends) = 0.36, *SD* = 0.66 | .746 | 0.0 (partial eta) |  |  |
|  |  |  |  | *M*(physical activity) = 1.09, *SD* = 0.74 | | *M*(physical activity) = 0.81, *SD* = 0.77 | .064 | 0.016 (partial eta) |  |  |
|  |  |  |  | *M*(depression and anxiety) = 2.51, *SD* = 2.54 | | *M*(depression and anxiety) = 2.79, *SD* = 2.75 | .140 | 0.012 (partial eta) |  |  |
|  |  |  |  | *M*(avoidance) = 0.09, *SD* = 0.43 | | *M*(avoidance) = 0.17, *SD* = 0.52 | .921 | 0.000 (partial eta) |  |  |
|  |  |  |  | *M*(negative cognitions) = 0.65, *SD* = 1.14 | | *M*(negative cognitions) = 1.29, *SD* = 1.81 | .063 | 0.027 (partial eta) |  |  |
| Song et al. (2023) | Depression; alcohol abuse | *n* = 8,329 | Patients after COVID-19 without Long COVID (*n* = 600,161) | % of patients with depression: 21.1% | | % of patients with depression: 12.8% | <.001 | – |  |  |
|  |  |  |  | % of patients with  alcohol abuse: 1.3% | | % of patients with alcohol abuse = 1.2% | 1.0 | – |  |  |
| Staples et al. (2023) | Depression; Anxiety | *n* = 1,873 | Healthy controls without a history of COVID-19 (*n* = 6,151)  Patients with acute COVID-19 (*n* = 2,417)  Patients after COVID-19 without Long COVID (*n* = 7,468) |  | | Healthy controls without a history of COVID-19: |  |  |  |  |
|  |  |  |  | *M*(Depression) = 16.0, *SD* = 6.2 | | *M*(Depression) = 14.6, *SD* = 6.5 | <.001 | F = 65.7 |  |  |
|  |  |  |  | *M*(Anxiety) = 13.3, *SD* = 5.2 | | *M*(Anxiety) = 12.2, *SD* = 5.5 | <.001 | F = 39.3 |  |  |
|  |  |  |  |  | | Patients with acute COVID-19: |  |  |  |  |
|  |  |  |  |  |  | *M*(Depression) = 14.1, *SD* = 6.3 |  |  |  |  |
|  |  |  |  |  |  | *M*(Anxiety) = 12.1, *SD* = 5.3 |  |  |  |  |
|  |  |  |  |  |  | Patients after COVID-19 without Long COVID: |  |  |  |  |
|  |  |  |  |  |  | *M*(Depression) = 13.8, *SD* = 6.3  *M*(Anxiety) = 11.8, *SD* = 5.4 |  |  |  |  |
| Tebeka et al. (2023) | Depression; Anxiety | *n* = 1,095 | Patients after COVID-19 without Long COVID (*n* = 1,021) | *M*(Depression) = 3.55 | | *M*(Depression) = 3.17 | <.0001 | – |  |  |
|  |  |  |  | *M*(Anxiety) = 3.65 | | *M*(Anxiety) = 3.28 | <.0001 | – |  |  |
|  |  |  |  | % of patients with Depression: 22.2%, 95% CI [19.7-24.6] | | % of patients with Depression: 18.0%, 95% CI [15.6-20.4] |  |  |  |  |
|  |  |  |  | % of patients with Anxiety: 23.3%, 95% CI [20.8-25.8] | | % of patients with Anxiety: 17.6%, 95% CI [15.2-19.2] |  |  |  |  |
|  |  |  |  | % of patients with chronic depression: 10.7% | | % of patients with chronic depression: 9.2% |  |  |  |  |
|  |  |  |  | % of patients with chronic anxiety: 18.4% | | % of patients with chronic anxiety: 14.8% |  |  |  |  |
| Terai et al. (2023) | Anxiety;  Depression;  Fear of COVID-19 | *n* = 433 at 3MFU  *n* = 350 at 6MFU  *n* = 239 at 12MFU | Patients after COVID-19 without Long COVID (*n* = 502 at 3MFU; *n* = 515 at 6MFU; *n* = 485 at 12MFU) | 3MFU: | |  |  |  | No mean values reported, only significant differences between groups for each FU |  |
|  |  |  |  | Anxiety | |  | < .0001 | – |  |  |
|  |  |  |  | Depression | |  | < .0001 | – |  |  |
|  |  |  |  | Fear of COVID-19 | |  | < .0001 | – |  |  |
|  |  |  |  | 6MFU: | |  |  |  |  |  |
|  |  |  |  | Anxiety | |  | < .0001 | – |  |  |
|  |  |  |  | Depression | |  | < .0001 | – |  |  |
|  |  |  |  | Fear of COVID-19 | |  | < .0001 | – |  |  |
|  |  |  |  | 12MFU: | |  |  |  |  |  |
|  |  |  |  | Anxiety | |  | < .0001 | – |  |  |
|  |  |  |  | Depression | |  | < .0001 | – |  |  |
|  |  |  |  | Fear of COVID-19 | |  | < .0001 | – |  |  |
| Uniyal et al. (2022) | Alcoholism | *n* = 18 | Patients after COVID-19 without Long COVID (*n* = 226) | No. and % of patients with alcoholism: 2 (11.1%) | | No. and % of patients with alcoholism: 38 (16.8%) | .53 | – |  |  |
| Wright et al. (2024) | Depression | *n* = 10 | Patients after COVID-19 without Long COVID (*n* = 13) | *M*(Depression) = 16.8, *SD* = 10.7 | | *M*(Depression) = 2.2, *SD* = 5.4 | .003 | – |  |  |
| Zhang et al. (2023b) | Anxiety; Depression; PTSD; Anxiety or depression | *n* = 753 | Patients after COVID-19 without Long COVID (*n* = 606) | 2YFU: | | 2YFU: | – | – | *P*-values not reported |  |
|  |  |  |  | No. and % of patients with anxiety: 98 (13.0%) | | No. and % of patients with anxiety: 13 (2.0%) |  |  |  |  |
|  |  |  |  | No. and % of patients with depression: 81 (11.0%) | | No. and % of patients with depression: 6 (1.0%) |  |  |  |  |
|  |  |  |  | No. and % of patients with PTSD: 28 (4.0%) | | No. and % of patients with PTSD: 1 (0%) |  |  |  |  |
|  |  |  |  | No. and % of patients with anxiety or depression: 160 (21.0%) | | No. and % of patients with anxiety or depression: 25 (4.0%) |  |  |  |  |
|  |  |  |  | 3YFU: | | 3YFU: |  |  |  |  |
|  |  |  |  | No. and % of patients with anxiety: 156 (21.0%) | | No. and % of patients with anxie ty: 41 (7.0%) |  |  |  |  |
|  |  |  |  | No. and % of patients with depression: 278 (37.0%) | | No. and % of patients with depression: 74 (12.0%) |  |  |  |  |
|  |  |  |  | No. and % of patients with PTSD: 120 (16.0%) | | No. and % of patients with PTSD: 37 (6.0%) |  |  |  |  |
|  |  |  |  | No. and % of patients with anxiety or depression: 291/752 (52.0%) | | No. and % of patients with anxiety or depression: 107 (18.0%) |  |  |  |  |

*Note.* $\tilde{X}$ = median; BDI-13 = Beck Depression Inventory-13; CI = confidence interval; EQ-5D-3L = European Quality of Life 5 Dimensions 3 Level Version; FMS = fibromyalgia syndrome; FSS = Fatigue Severity Scale; FU = follow-up; HADS = Hospital Anxiety and Depression Scale; HC = healthy controls; ICU = intensive care unit; IQR = interquartile range; *M* = Mean; ME/CFS = myalgic encephalomyelitis/chronic fatigue syndrome; MFU = month follow-up; No. = number; PHQ-2 = Patient Health Questionnaire-2; PROMIS = Patient-Reported Outcomes Measurement; PTSD = post-traumatic stress disorder; RQ = research question; *SD* = standard deviation; YFU = year follow-up; ZSDS = Zung Self-Rating Depression Scale.

## **eTable 3. Overview of included studies providing cross-sectional associations with psychological variables (RQ2)**

| **Author (year of**  **publication)** | **Long COVID (sample size)** | **Type of control group (*n*)** | **Psychological**  **variables(s) Mean (*SD*)**  if not stated otherwise | **Condition-relevant**  **outcome(s) (measuring instrument; Mean (*SD*))**  if not stated otherwise | **Psychological variable(s)**  **(correlation/ regression)**  if not stated otherwise | **Significant**  **difference (*p*-value)** | **Related results (short summary)** | **Comments** |
| --- | --- | --- | --- | --- | --- | --- | --- | --- |
| Azcue et al. (2022) | *n* = 73 | ME/CFS patients without Long COVID (*n* = 42) | Long COVID:  Depression *M*(Depression) = 6.97, *SD* = 3.97  Suicidal ideation  *M*(suicidal ideation) = 0.24, *SD* = 0.66  Anxiety  *M*(Anxiety Trait) = 17.08, *SD* = 14.04  *M*(Anxiety State) = 27.12, *SD* = 14.12  ME/CFS:  Depression  *M*(Depression) = 9.35, *SD* = 3.21  Suicidal ideation  *M*(suicidal ideation) = 0.834, *SD* = 1.32  Anxiety  *M*(Anxiety Trait) = 26.97, *SD* = 17.12  *M*(Anxiety State) = 31.42, *SD* = 15.32 | Physical problems (SF-36) = 49.21 (24.71)  *M Control* = 32.14 (21.24)  Limitations due to physical problems (SF-36) = 22.22 (29.23)  *M Control* = 9.22 (14.16)  Pain (SF-36) = 33.28 (24.64)  *M Control* = 27.67 (30.18)  Social role (SF-36) = 32.46 (30.45)  *M Control* = 13.27 (22.27)  Mental health (SF-36) = 60.63 (25.04)  *M Control* = 49.64 (24.99)  Limitations due to mood problems (SF-36) = 73.13 (29.85)  *M Control* = 61.50 (33.58)  Fatigue/energy (SF-36) = 15.90 (15.81)  *M Control* = 9.37 (12.51)  Own's health perception (SF-36) = 38.13 (21.29)  *M Control* = 23.39 (16.34)  Fatigue (MFIS) = 62.68 (15.41)  *M Control* = 68.09 (12.74) | Depression & Fatigue (MFIS):  \|r\| = 0.47 | <.01 | The correlation analysis revealed significant negative correlations between the psychological variables depression, anxiety, and suicidal ideation with several quality of life indices and positive correlations with fatigue |  |
|  |  |  |  |  | Depression & Limitations due to physical problems (SF-36):  \|r\| = -0.28 | <.05 |  |  |
|  |  |  |  |  | Depression & Pain (SF-36):  \|r\| = -0.35 | <.01 |  |  |
|  |  |  |  |  | Suicidal ideation & Fatigue (MFIS):  \|r\| = 0.38 | <.01 |  |  |
|  |  |  |  |  | Suicidal ideation & Limitations due to physical problems (SF-36):  \|r\| = 0.26 | <.05 |  |  |
|  |  |  |  |  | Suicidal ideation & Pain (SF-36):  \|r\| = -0.26 | <.05 |  |  |
|  |  |  |  |  | Anxiety (State) & Fatigue (MFIS):  \|r\| = 0.31 | <.01 |  |  |
|  |  |  |  |  | Anxiety (State) & Limitations due to physical problems (SF-36): non sig | non sig |  |  |
|  |  |  |  |  | Anxiety (State) &  Pain (SF-36):  \|r\| = -0.26 | <.05 |  |  |
|  |  |  |  |  | Anxiety (Trait) & Fatigue (MFIS), Limitations due to physical problems (SF-36), Pain (SF-36): all non sig | non sig |  |  |
| Benoit-Piau et al. (2023) | *n* = 622  Long COVID & FSS < 5: *n* = 387 (32.5%)  Long COVID & FSS ≥ 5: *n* = 181 (15.25) | Patients after COVID-19 without Long COVID (*n* = 727)  No Long COVID & FSS < 5: *n* = 571 (47.9%)  No Long COVID & FSS ≥ 5: 53 (4.4%) | Pre-existing mental health disorders  No. and % of patients with pre-existing mental health disorder:  Long COVID & FSS < 5: 82 (21.4%)  Long COVID & FSS ≥ 5: 80 (44.9%)  No Long COVID & FSS < 5: 77 (13.7%)  No Long COVID & FSS ≥ 5: 21 (40.4%) | Fatigue (FSS): 31.9% ≥ 5  Fatigue (SOFA/GP) : 37.8% ≥ 2 | Pre-existing mental health disorder & Long COVID (FSS < 5):  *OR*: 1.715, 95% *CI* [1.218-2.415] | .002 | A diagnosis of mental health disorder was significantly associated with Long COVID |  |
|  |  |  |  |  | Pre-existing mental health disorder & Long COVID (FSS ≥ 5):  *OR*: 5.155, 95% *CI* [3.509-7.519] | <.001 |  |  |
| Beyer et al. (2023) | *n* = 69 | – | Depression  *M*(Depression) = 6.0, *SD* = 7.0  Anxiety  *M*(Anxiety) = 6.5, *SD* = 8.0 | Fatigue (FAS) = 35.0 (7.4)  HRQoL (SF-36)  physical score = 35.2 (9.0); mental score = 40.9 (12.8) | Depression & Fatigue:  \|r\| = 0.331 | .009 | Depression and Anxiety significantly correlated with fatigue |  |
|  |  |  |  |  | Anxiety & Fatigue:  \|r\| = 0.290 | .022 |  |  |
| Bierbauer et al. (2022) | *n* = 246 | – | Personal control  *M*(personal control) = 2.89, *SD* = 1.12)  Treatment control  *M*(treatment control) = 2.06, *SD* = 0.98  Coherence  *M*(coherence) = 1.89, *SD* = 1.17  Emotional representation  *M*(emotional representation) = 3.40, *SD* = 1.09  Illness identity  *M*(illness identity) = 15.06, *SD* = 5.54  Depressive symptoms  *M*(Depressive symptoms) = 8.45, *SD* = 3.97)  Anxiety  *M*(Anxiety) = 7.83, *SD* = 3.98 | Fatigue (CIS20) = 56.65 (12.35)  QoL (SF-12, PCS) = 35.75 (9.96) | Personal control & Fatigue:  \|r\| = -0.18 | <.01 | The correlation analysis revealed significant correlations between aspects of illness identity, depression, and anxiety with quality of life and fatigue |  |
|  |  |  |  |  | Treatment control & Fatigue:  \|r\| = -0.20 | <.01 |  |  |
|  |  |  |  |  | Coherence & Fatigue:  \|r\| = -0.19 | <.01 |  |  |
|  |  |  |  |  | Emotional representation & Fatigue:  \|r\| = 0.28 | <.01 |  |  |
|  |  |  |  |  | Illness identity & Fatigue:  \|r\| = -0.48 | <.01 |  |  |
|  |  |  |  |  | Depressive symptoms & Fatigue:  \|r\| = 0.56 | <.01 |  |  |
|  |  |  |  |  | Anxiety & Fatigue:  \|r\| = -0.26 | <.01 |  |  |
|  |  |  |  |  | Personal control & QoL:  \|r\| = -0.02 | non sig |  |  |
|  |  |  |  |  | Treatment control & QoL:  \|r\| = 0.10 | non sig |  |  |
|  |  |  |  |  | Coherence & QoL:  \|r\| = -0.02 | non sig |  |  |
|  |  |  |  |  | Emotional representation & QoL:  \|r\| = 0.02 | non sig |  |  |
|  |  |  |  |  | Illness identity & QoL:  \|r\| = -0.30 | <.01 |  |  |
|  |  |  |  |  | Depressive symptoms & QoL:  \|r\| = -0.14 | <.05 |  |  |
|  |  |  |  |  | Anxiety & QoL:  \|r\| = -0.13 | non sig |  |  |
| Bonner et al. (2024) | *n* = 7,183 (whole sample) | Healthy controls without a history of COVID-19 | Anxiety/Depression  No. and % of Long COVID patients with Anxiety or Depression: 1,289 (38.5%) | Disability (broad spectrum of limitations) | Anxiety/Depression & Long COVID:  *OR*: 1.38, 95% *CI* [1.19-1.61] | <.05 | Anxiety disorders or depression were significantly associated with increased odds of Long COVID and disability |  |
|  | *n* = 18,752 (whole sample) |  |  |  | Anxiety/Depression & disability:  *OR*: 3.97, 95% *CI* [3.61-4.36] | <.05 |  |  |
| Bungenberg et al. (2022) | *n* = 50 | – | Affective Symptoms (HADS)  *M*(Depression) = 5.08, *SD* = 3.45  *M*(Anxiety) = 6.88, *SD* = 4.41 | Fatigue (FSMC) = 61.8 (19.03) | Hospitalised patients:  Affective symptoms & Fatigue:  \|r\| = 0.65 | <.05 | In the correlation analysis, affective symptoms were shown to be positively associated with fatigue severity |  |
|  |  |  |  |  | Non-Hospitalised patients:  Affective symptoms & Fatigue:  \|r\| = 0.59 | <.01 |  |  |
| Burton et al. (2023) | *n* = 74 | – | Anxiousness  $\tilde{X}$(anxiousness) = 23 (IQR 4-37)  Physical activity  $\tilde{X}$(physical activity) = 30 (IQR 24.3-36.7) | Baseline:  HRQoL (EQ5D-5L, EQ-VAS)  $\tilde{X}$(EQ5D) = 0.64 (IQR 0.37-0.75)  $\tilde{X}$(EQ-VAS) = 40 (IQR 30-50)  Social participation (PROMIS P8a)  $\tilde{X}$= 11 (IQR 8-18)  Fatigue (FACIT-Fatigue)  $\tilde{X}$= 15 (IQR 8-20)  Physical symptoms (PHQ-15)  $\tilde{X}$= 15 (IQR 13-18)  Post-Exertional Malaise (Post-Exertional Malaise scale)  $\tilde{X}$= 15 (IQR 10-18)  Overall unwellness (VAS)  $\tilde{X}$ = 59 (IQR 52-66)  Physical demand  Combined physical, emotional and mental demand  (each 4-point Likert scale) | *Within-person associations, mean correlation coefficient*  Anxious & Overall unwellness (*n* = 57):  \|r\| = 0.25  Anxious & Fatigue (*n* = 57):  \|r\| = 0.21  Anxious & Breathing (*n* = 49):  \|r\| = 0.16  Anxious & Lightheaded (*n* = 46):  \|r\| = 0.2  Anxious & Taste (*n* = 26):  \|r\| = 0.08  Anxious & Pain (*n* = 49):  \|r\| = 0.19  Anxious & Thinking (*n* = 52):  \|r\| = 0.32 | – | Pooled within-subject correlations showed anxiety correlated with cognitive difficulty, but not with fatigue |  |
|  |  |  |  |  | *Pooled between-person analysis, partial correlations*  Anxious & Pain  Partial coefficient = 0.12  Anxious & Breathing  Partial coefficient = 0.15  Anxious & Thinking  Partial coefficient = 0.16  *Pooled within-person contemporaneous analysis*  Anxious & Pain  Partial coefficient = 0.02  Anxious & Breathing  Partial coefficient = 0.04  Anxious & Thinking  Partial coefficient = 0.16  Anxious & Fatigue  Partial coefficient = 0.02 |  |  |  |
|  |  |  |  |  | *Within-person correlation, mean correlation coefficient*  Mean activity & physical demand:  \|r\| = 0.45  Mean activity & combined demand:  \|r\| = 0.34  Mean activity (last 3 hours) & Fatigue:  \|r\| = -0.09  Mean activity (most recent hour) & Fatigue:  \|r\| = 0.02  Mean activity (next 3 hours) & Fatigue:  \|r\| = -0.11 |  |  |  |
| Busatto et al. (2022) | *n* = 749 | – | PTSD  Depression  Anxiety  Mean values or %s not reported | Presence of Long COVID | Discrimination coefficient:  Coefficient (PTSD) = 2.110, 95% *CI* [1.618-2.602]  Coefficient (Depression) = 2.052, 95% *CI* [1.603-2.502] | Global p-value for psychiatric/cognitive symptoms <.001 | Different symptoms that persist for several months after moderate or severe COVID-19 were dominated by fatigue and psychiatric symptoms | Latent variable of PASC symptoms was generated using two-parameter logistic IRT modelling |
|  |  |  |  |  | Coefficient (Anxiety) = 1.961, 95% *CI* [1.548-2.373] |  |  |  |
| Delgado-Alonso et al. (2022a) | *n* = 93 | Matched healthy controls without a history of COVID-19 (*n* = 93) | Long COVID:  Agreeableness  *M*(agreeableness) = 47.94, *SD* = 22.47  Conscientiousness  *M*(conscientiousness) = 50.33, *SD* = 29.05  Extraversion  *M*(extraversion) = 45.04, *SD* = 30.79  Emotional stability  *M*(emotional stability) = 34.15, *SD* = 27.0  Openness  *M*(openness) = 40.02, *SD* = 31.45  No Long COVID:  Agreeableness  *M*(agreeableness) = 55.66, *SD* = 22.40  Conscientiousness  *M*(conscientiousness) = 58.38, *SD* = 26.82  Extraversion  *M*(extraversion) = 56.38, *SD* = 29.78  Emotional stability  *M*(emotional stability) = 45.24, *SD* = 27.0  Openness  *M*(openness) = 48.62, *SD* = 28.09 | Fatigue (MFIS) = 52.96 (15.14) | Openness & Fatigue:  \|r\| = 0.07 | non sig | No statistically significant correlations were identified between the five personality  factors and fatigue |  |
|  |  |  |  |  | Emotional stability & Fatigue:  \|r\| = -0.21 | non sig |  |  |
|  |  |  |  |  | Extraversion & Fatigue:  \|r\| = -0.19 | non sig |  |  |
|  |  |  |  |  | Conscientiousness & Fatigue:  \|r\| = 0.03 | non sig |  |  |
|  |  |  |  |  | Agreeableness & Fatigue:  \|r\| = 0.24 | non sig |  |  |
| Delgado-Alonso et al. (2022b) | *n* = 50 | Matched healthy controls without a history of COVID-19 (*n* = 50) | Anxiety  *M*(Anxiety Trait) = 49.34, *SD* = 11.47  *M*(Anxiety State) = 41.08, *SD* = 12.29  Depression  *M*(Depression) = 16.00, *SD* = 8.86 | Global cognitive composite score derived as the mean of z-scores of all cognitive tests: forward and backward digit span, (CBT), (SDMT), (BNT), (JLO),  (ROCF), (FCSRT), verbal fluencies, (SCWT), (VOSP) and computerised neuropsychological battery Vienna Test System  Fatigue (MFIS) = 55.15 (15.15) | Anxiety & cognitive score:  \|r\| = -0.342 | .022 | Anxiety, but not depression, significantly correlated with the global cognitive composite score |  |
|  |  |  |  |  | Depression & cognitive score:  \|r\| = -0.234 | .109 |  |  |
| Delgado-Alonso et al. (2023) | *n* = 170 | – | Depressive symptoms  Trait-Anxiety  State-Anxiety | Brain fog (FLEI)  Attention = 24.86 (7.97)  Memory = 25.71 (7.24)  Executive function = 18.36 (8.85)  Mental ability = 68.94 (22.28) | Depression & brain fog:  \|r\| = 0.445 | <.001 | Depression, Trait-Anxiety and State-Anxiety significantly correlated with brain fog |  |
|  |  |  |  |  | Trait-Anxiety & brain fog:  \|r\| = 0.418 | <.001 |  |  |
|  |  |  |  |  | State-Anxiety & brain fog:  \|r\| = 0.304 | <.001 |  |  |
| Elmunzer et al. (2023) | *n* = 104 | Patients after COVID-19 without Long COVID (*n* = 12) | PTSD  No. and % of all patients with PTSD: 48 (41.4%)  Depressive symptoms  Anxiety symptoms | Presence and severity of COVID-19-related symptoms (self-developed questions)  Gastrointestinal symptom severity (GSRS) | PTSD & no. of current moderate or severe COVID-related symptoms:  \|r\| = 0.51  *β* = 0. 462, *t* = 5.474 | < .0001  < .0001 | Higher degree of psychological trauma related to the illness experience, depressive symptoms and anxiety symptoms significantly correlated with the no. of persistent COVID-related symptoms and with gastrointestinal symptom severity and higher psychological trauma score was significantly associated with the no. of moderate or severe persistent COVID-related symptoms and with gastrointestinal symptom severity |  |
|  |  |  |  |  | PTSD & gastrointestinal symptom severity:  \|r\| = 0.42  *β* = 0. 420, *t* = 4.653 | < .0001  < .0001 |  |  |
|  |  |  |  |  | Depression & no. of current COVID-related symptoms:  \|r\| = 0.62 | < .0001 |  |  |
|  |  |  |  |  | Depression & gastrointestinal symptom severity:  \|r\| = 0.56 | < .0001 |  |  |
|  |  |  |  |  | Anxiety & no. of current COVID-related symptoms:  \|r\| = 0.43 | < .0001 |  |  |
|  |  |  |  |  | Anxiety & gastrointestinal symptom severity:  \|r\| = 0.42 | < .0001 |  |  |
|  |  |  |  |  |  |  |  |  |
| Fernández-de-Las-Peñas et al. (2022) | *n* = 146 | – | Sensitisation  *M*(Sensitisation) = 33.9, *SD* = 17.25  Anxiety  *M*(Anxiety) = 5.3, *SD* = 4.2  Depression  *M*(Depression) = 5.1, *SD* = 4.3  Kinesiophobia  *M*(Kinesiophobia) = 24.1, *SD* = 8.55  Pain Catastrophising  *M*(Pain Catastrophising) = 12.15, *SD* = 11.95 | HRQoL (EQ-5D-5L) = 0.75 (0.2)  Pain intensity (numeric pain rating scale) = 5.6 (1.7) | Sensitisation & QoL:  \|r\| = -0.199 | <.05 | The study revealed significant associations between psychological variables and quality of life (sensitisation, depression and catastrophising) and sensitisation, anxiety and depression were significantly associated with pain intensity |  |
|  |  |  |  |  | Anxiety & QoL:  \|r\| = non sig | non sig |  |  |
|  |  |  |  |  | Depression & QoL:  \|r\| = -0.174 | <.05 |  |  |
|  |  |  |  |  | Kinesiophobia & QoL:  \|r\| = non sig | non sig |  |  |
|  |  |  |  |  | Catastrophising & QoL:  \|r\| = -0.21 | <.05 |  |  |
|  |  |  |  |  | Sensitisation & pain intensity:  \|r\| = 0.19 | <.05 |  |  |
|  |  |  |  |  | Anxiety & pain intensity:  \|r\| = 0.175 | <.05 |  |  |
|  |  |  |  |  | Depression & pain intensity  \|r\| = 0.225 | <.01 |  |  |
|  |  |  |  |  | Kinesiophobia & pain intensity:  \|r\| = non sig | non sig |  |  |
|  |  |  |  |  | Catastrophising & pain intensity:  \|r\| = -0.21 | non sig |  |  |
| Fujita et al. (2023) | *n* = 482 | – | Anxiety symptoms | Presence of Long COVID (medical interviews)  HRQoL (EQ-5D-5L)  $\tilde{X}$(Long COVID patients without headache) = 0.61  $\tilde{X}$(Long COVID patients with headache) = 0.54 | Anxiety & QoL:  *OR*: 1.29, 95% *CI* [0.73-2.29] | .39 | Self-reported anxiety after COVID-19 was not significantly related to quality of life |  |
| Giurgi-Oncu et al. (2021) | *n* = 143 | – | Depressive symptoms  $\tilde{X}$(64 hospitalised patients) = 10 (IQR 10-19)  $\tilde{X}$(79 outpatients) = 8 (IQR 6-11)  Anxiety symptoms  $\tilde{X}$(64 hospitalised patients) = 10 (IQR 7-16)  $\tilde{X}$(79 outpatients) = 9 (IQR 7-13) | No. of persisting symptoms  $\tilde{X}$(64 hospitalised patients) = 5 (IQR 4-6)  $\tilde{X}$(79 outpatients) = 3 (IQR 2-4)  QoL (EQ-5D-5L)  $\tilde{X}$ VAS score(64 hospitalised patients) = 62 (IQR 48-74.75)  $\tilde{X}$ VAS score(79 outpatients) = 66 (IQR 60-75)  Impairment (PCFS)  $\tilde{X}$(64 hospitalised patients) = 2 (IQR 2-3)  $\tilde{X}$(79 outpatients) = 1 (IQR 1-2) | Depression & no. of persisting symptoms:  \|r\| = 0.726 [0.630; 0.802] | <.001 | There were strong statistically significant correlations between depression as well as anxiety symptoms and the no. of persisting symptoms; other significant correlations were evidenced between depression as well as anxiety symptoms and quality of life scores |  |
|  |  |  |  |  | Anxiety & no. of persisting symptoms:  \|r\| = 0.440 [0.290; 0.577] | <.001 |  |  |
|  |  |  |  |  | Depression & QoL VAS score:  \|r\| = 0.652 [0.767;0.505] | <.001 |  |  |
|  |  |  |  |  | Anxiety & QoL VAS score:  \|r\| = 0.702 [0.783;0.583] | <.001 |  |  |
| Gouraud et al. (2021) | *n* = 29 | Patients after COVID-19 without Long COVID (*n* = 71) | Psychological distress  $\tilde{X}$(all patients) = 10 (IQR 6-14) | Presence of Long COVID (presence of cognitive complaints yes/no; self-developed questions)  (SVFT)  $\tilde{X}$ All patients = 18 (14-21)  (DSST)  $\tilde{X}$ All patients = 50 (38-62)  (MMSE)  $\tilde{X}$All patients = 28 (26-30) | Psychological distress & presence of cognitive complaints:  Adjusted *OR*: 1.96, 95% *CI* [1.08-3.57] | .028 | There was a significant association between higher psychological distress and the presence of persistent cognitive complaints |  |
| Harenwall et al. (2022) | *n* = 154 | – | PTSD  *M*(PTSD) = 1.29, *SD* = 0.80 | Long COVID symptom severity (fatigue; adapted C19-YRS) = 7.04 (2.18)  HRQoL (EuroQol EQ-5D-5L) = 0.54 (0.24) | PTSD symptoms & persistent fatigue severity:  *β* = 0.15, *t* = 1.72 | .088 | Baseline PTSD symptoms were not significantly associated with baseline persistent fatigue |  |
| Hastie et al. (2022) | *n* = 1,856 (no recovery) | – | Pre-existing Depression or Anxiety  No. and % of all patients after symptomatic COVID-19: 13,501 (42.88%) | Self-reported recovery status after COVID-19 (self-reported categories: full, partial or none)  Long COVID symptoms (self-developed questions)  Limitations in daily activities (self-developed questions)  QoL (EQ-5D) $\tilde{X}$(whole sample) = 75 (60-89) | Pre-existing Depression or Anxiety & status of no recovery:  *OR*: 2.29, 95% *CI* [2.06-2.55] | – | Following previous symptomatic infection, pre-existing Depression or Anxiety was associated with lack of complete recovery |  |
|  | *n* = 13,350 (partial recovery) | – |  |  |  |  |  |  |
|  |  |  |  |  | Pre-existing Depression or Anxiety & status of partial recovery:  *OR*: 1.66, 95% *CI* [1.58-1.75] | – |  |  |
| Hedberg et al. (2023) | Non-hospitalised: *n* = 1,963 | Non-hospitalised patients after COVID-19 without Long COVID (*n* = 189,496) | Pre-existing mental health disorders (comorbidities)  No. and % of Long COVID patients with mental health disorders:  Non-hospitalised patients: 720 (36.7%)  Hospitalised patients: 195 (28.1%)  ICU-treated patients: 89 (21.7%)  No. and % of patients without Long COVID with mental health disorders:  Non-hospitalised patients: 36,579 (19.3%)  Hospitalised patients: 2,322 (20.4%)  ICU-treated patients: 154 (17.8%) | Presence of Long COVID (diagnosis)  Long COVID symptoms (symptom-based  diagnosis codes)  Healthcare use | Pre-existing mental health disorders & Long COVID diagnosis in formerly non-hospitalised patients:  *OR*: 2.18, 95% *CI* [1.98-2.39] | – | Pre-existing mental health disorders were significantly associated with a PCC diagnosis among non-hospitalised and hospitalised individuals |  |
|  |  |  |  |  | Pre-existing mental health disorders & Long COVID diagnosis in formerly hospitalised patients:  *OR*: 1.38, 95% *CI* [1.17-1.64] | – |  |  |
|  | Hospitalised: *n* = 693 | Hospitali sed patients after COVID-19 without Long COVID (*n* = 11,377) |  |  |  |  |  |  |
|  |  |  |  |  | Pre-existing mental health disorders & Long COVID diagnosis in formerly ICU-treated patients:  *OR*: 1.11, 95% *CI* [0.88-1.42] | – |  |  |
|  | ICU-treated: *n* = 410 | ICU-treated patients after COVID-19 without Long COVID (*n* = 866) |  |  |  |  |  |  |
| Heine et al. (2023) | *n* = 47 | Matched healthy controls without a history of COVID-19 (*n* = 47) | Long COVID:  Depressive symptoms  *M*(Depression) = 17.0, *SD* = 7.8  Anxiety symptoms  *M*(Anxiety) = 14.6, *SD* = 7.5  No Long COVID:  Depressive symptoms  *M*(Depression) = 4.8, *SD* = 5.4  Anxiety symptoms *M*(Anxiety) = 3.9, *SD* = 4.8 | Presence of Long COVID  Fatigue severity (FSMC)  Overall = 77.0 (10.1)  *Control* = 33.2 (11.1)  Physical fatigue = 38.2 (5.6)  *Control* = 16.9 (5.6)  Cognitive fatigue = 38.9 (6.1)  *Control* = 16.3 (6.0) | Depressive symptoms & post-COVID fatigue:  \|r\| = 0.577  *β* = 0.52 | <.0001  .049 | Higher levels of depressiveness and anxiety significantly correlated with fatigue severity and post-acute depressiveness was significantly associated with post-COVID fatigue |  |
|  |  |  |  |  | Anxiety symptoms & post-COVID fatigue:  \|r\| = 0.430 | .0050 |  |  |
| Hirahata et al. (2022) | *n* = 1,891 | – | No. and % of patients with Depressive symptoms: 1,535 (81.2%) | Presence of self-reported Long COVID symptoms (obtained from electronic medical records)  No. of symptoms = 8.4 (3.2)  Impairment in activities of daily living (PS Score) = 3.1 (2.4) | Depressive symptoms & performance status:  *β* = 0.47, 95% *CI* [0.21-0.73] | <.05 | Depressive symptoms were significantly associated with lower performance status |  |
| Hirschtick et al. (2023) | *n* = 1,913 (whole sample) | – | Pre-existing psychological condition  % of all patients with a pre-existing psychological condition: 12.6% | Presence of Long COVID: 40.0% | Psychological condition & Long COVID:  Adjusted *PR*: 1.24, 95% *CI* [1.06-1.44] | <.01 | A pre-existing psychological condition was significantly associated with Long COVID |  |
| Jung et al. (2022) | *n* = 447 | Patients < 4 weeks after COVID-19 (*n* = 675) | Long COVID:  Depression  *M*(Depression HADS) = 7.5, *SD* = 4.7  Anxiety  *M*(Anxiety HADS) = 6.1, *SD* = 4.7  No Long COVID:  Depression  *M*(Depression HADS) = 7.4, *SD* = 4.6  Anxiety  *M*(Anxiety HADS) = 6.2, *SD* = 4.8 | Long COVID symptoms (self-developed questionnaire) = 8.1 (5.2) symptoms  *Control* = 7.6 (4.6) symptoms | Depressive symptoms & no. of Long COVID symptoms:  B = 0.163, *SE* = 0.064 | .012 | Depressive and Anxiety symptoms assessed with the HADS were significantly associated with the no. of Long COVID symptoms |  |
|  |  |  |  |  | Anxiety symptoms & no. of Long COVID symptoms:  B = 0.282, *SE* = 0.062 | <.001 |  |  |
| Jung et al. (2023) | *n* = 440 | – | Depression  *M*(Depression HADS) = 7.5, *SD* = 4.7  Anxiety  *M*(Anxiety HADS) = 6.0, *SD* = 4.7 | Long COVID symptoms (self-developed questionnaire)  Brain fog: 38.6% (*n* = 170)  Headache: 31.1% (*n* = 137)  Dizziness: 29% (*n* = 128)  Memory impairment: 23.6% (*n* = 104) | Depressive symptoms & brain fog:  *OR*: 1.05, 95% *CI* [0.97-1.15] | .230 | Anxiety symptoms assessed with the HADS were significantly associated with brain fog; Anxiety (HADS-Anxiety score ≥ 8), severe Anxiety (HADS-Anxiety score ≥ 11), Depression (HADS-Depression score ≥ 8) and severe Depression (HADS- Depression score ≥ 11) were significantly associated with brain fog, headache, dizziness and memory impairment |  |
|  |  |  |  |  | Anxiety symptoms & brain fog:  *OR*: 1.09, 95% *CI* [1.01-1.18] | .037 |  |  |
|  |  |  |  |  | Depressive symptoms & headache:  *OR*: 1.00, 95% *CI* [0.93-1.09] | .921 |  |  |
|  |  |  |  |  | Anxiety symptoms & headache:  *OR*: 1.04, 95% *CI* [0.97-1.12]  Depressive symptoms & dizziness:  *OR*: 0.98, 95% *CI* [0.90-1.07]  Anxiety symptoms & dizziness:  *OR*: 1.06, 95% *CI* [0.99-1.15]  Depressive symptoms & memory impairment:  *OR*: 1.04, 95% *CI* [0.95-1.14]  Anxiety symptoms & memory impairment:  *OR*: 1.02, 95% *CI* [0.94-1.11]  Depression (cut-off score ≥ 8) & brain fog:  Adjusted *OR*: 4.64, 95% *CI* [2.94-7.33]  Anxiety (cut-off score ≥ 8) & brain fog:  Adjusted *OR*: 3.36, 95% *CI* [2.18-5.52]  Depression (cut-off score ≥ 8) & headache:  Adjusted *OR*: 1.82, 95% *CI* [1.17-2.82]  Anxiety (cut-off score ≥ 8) & headache:  Adjusted *OR*: 1.94, 95% *CI* [1.24-3.04]  Depression (cut-off score ≥ 8) & dizziness:  Adjusted *OR*: 2.22, 95% *CI* [1.41-3.49]  Anxiety (cut-off score ≥ 8) & dizziness:  Adjusted *OR*: 2.24, 95% *CI* [1.42-3.53]  Depression (cut-off score ≥ 8) & memory impairment:  Adjusted *OR*: 3.37, 95% *CI* [2.13-5.97]  Anxiety (cut-off score ≥ 8) & memory impairment:  Adjusted *OR*: 2.11, 95% *CI* [1.27-3.52]  Severe Depression (cut-off score ≥ 11) & brain fog:  Adjusted *OR*: 4.27, 95% *CI* [2.62-6.95]  Severe Anxiety (cut-off score ≥ 11) & brain fog:  Adjusted *OR*: 2.50, 95% *CI* [1.42-4.40]  Severe Depression (cut-off score ≥ 11) & headache:  Adjusted *OR*: 2.00, 95% *CI* [1.26-3.18] | .295 |  |  |
|  |  |  |  |  | Depressive symptoms & dizziness:  *OR*: 0.98, 95% *CI* [0.90-1.07] | .652 |  |  |
|  |  |  |  |  | Anxiety symptoms & dizziness:  *OR*: 1.06, 95% *CI* [0.99-1.15] | .117 |  |  |
|  |  |  |  |  | Depressive symptoms & memory impairment:  *OR*: 1.04, 95% *CI* [0.95-1.14] | .373 |  |  |
|  |  |  |  |  | Anxiety symptoms & memory impairment:  *OR*: 1.02, 95% *CI* [0.94-1.11] | .579 |  |  |
|  |  |  |  |  | Depression (cut-off score ≥ 8) & brain fog:  Adjusted *OR*: 4.64, 95% *CI* [2.94-7.33] | <.001 |  |  |
|  |  |  |  |  | Anxiety (cut-off score ≥ 8) & brain fog:  Adjusted *OR*: 3.36, 95% *CI* [2.18-5.52] | <.001 |  |  |
|  |  |  |  |  | Depression (cut-off score ≥ 8) & headache:  Adjusted *OR*: 1.82, 95% *CI* [1.17-2.82] | .008 |  |  |
|  |  |  |  |  | Anxiety (cut-off score ≥ 8) & headache:  Adjusted *OR*: 1.94, 95% *CI* [1.24-3.04] | .004 |  |  |
|  |  |  |  |  | Depression (cut-off score ≥ 8) & dizziness:  Adjusted *OR*: 2.22, 95% *CI* [1.41-3.49] | .001 |  |  |
|  |  |  |  |  | Anxiety (cut-off score ≥ 8) & dizziness:  Adjusted *OR*: 2.24, 95% *CI* [1.42-3.53] | .001 |  |  |
|  |  |  |  |  | Depression (cut-off score ≥ 8) & memory impairment:  Adjusted *OR*: 3.37, 95% *CI* [2.13-5.97] | <.001 |  |  |
|  |  |  |  |  | Anxiety (cut-off score ≥ 8) & memory impairment:  Adjusted *OR*: 2.11, 95% *CI* [1.27-3.52] | .004 |  |  |
|  |  |  |  |  | Severe Depression (cut-off score ≥ 11) & brain fog:  Adjusted *OR*: 4.27, 95% *CI* [2.62-6.95] | <.001 |  |  |
|  |  |  |  |  | Severe Anxiety (cut-off score ≥ 11) & brain fog:  Adjusted *OR*: 2.50, 95% *CI* [1.42-4.40] | .002 |  |  |
|  |  |  |  |  | Severe Depression (cut-off score ≥ 11) & headache:  Adjusted *OR*: 2.00, 95% *CI* [1.26-3.18] | .003 |  |  |
|  |  |  |  |  | Severe Anxiety (cut-off score ≥ 11) & headache:  Adjusted *OR*: 2.29, 95% *CI* [1.34-3.91] | .003 |  |  |
|  |  |  |  |  | Severe Depression (cut-off score ≥ 11) & dizziness:  Adjusted *OR*: 2.16, 95% *CI* [1.35-3.44] | .001 |  |  |
|  |  |  |  |  | Severe Anxiety (cut-off score ≥ 11) & dizziness:  Adjusted *OR*: 3.05, 95% *CI* [1.78-5.24] | <.001 |  |  |
|  |  |  |  |  | Severe Depression (cut-off score ≥ 11) & memory impairment:  Adjusted *OR*: 2.72, 95% *CI* [1.63-4.54] | <.001 |  |  |
|  |  |  |  |  | Severe Anxiety (cut-off score ≥ 11) & memory impairment:  Adjusted *OR*: 2.50, 95% *CI* [1.41-4.40] | <.001 |  |  |
| Lhuillier et al. (2022) | Long COVID: *n* = 366 | Patients after COVID-19 without Long COVID (*n* = 853) | Depression  Long COVID:  *M*(Depression) = 4.23, *SD* = 4.71  No Long COVID:  *M*(Depression) = 3.06, *SD* = 4.23  Post-acute COVID-19 Respiratory Sequelae:  *M*(Depression) = 4.82, *SD* = 5.0  No Post-acute COVID-19 Respiratory Sequelae:  *M*(Depression) = 3.15, *SD* = 4.3  Post-acute COVID-19 Central Nervous System Sequelae:  *M*(Depression) = 4.43, *SD* = 5.5  No Post-acute COVID-19 Central Nervous System Sequelae:  *M*(Depression) = 3.30, *SD* = 4.3  Post-acute COVID-19 Fatigue Sequelae:  *M*(Depression) = 5.09, *SD* = 4.9  No Post-acute COVID-19 Fatigue Sequelae:  *M*(Depression) = 3.26, *SD* = 4.4  Post-acute COVID-19 Muscular Sequelae:  *M*(Depression) = 5.47, *SD* = 6.5  No Post-acute COVID-19 Muscular Sequelae:  *M*(Depression) = 3.36, *SD* = 4.4 | Presence of Post-Acute COVID-19 Syndrome (self-reported symptom severity scales): *n* = 366  Presence of Post-acute COVID-19 Respiratory Sequelae (self-reported symptom severity scales): *n* = 206  Presence of Post-acute COVID-19 Central Nervous System Sequelae (self-reported symptom severity scales): *n* = 134  Presence of Post-acute COVID-19 Fatigue Sequelae (self-reported symptom severity scales): *n* = 108  Presence of Post-acute COVID-19 Muscular Sequelae (self-reported symptom severity scales): *n* = 34 | Depressive symptoms & presence of Long COVID:  Adjusted *RR*: 1.07, 95% *CI* [0.99-1.16] | .259 | Higher depressive symptoms were significantly associated with persistent fatigue, but not overall Long COVID, persistent respiratory symptoms, persistent cognitive symptoms or persistent muscular symptoms |  |
|  |  |  |  |  | Depressive symptoms & presence of Post-acute COVID-19 Respiratory Sequelae:  Adjusted *RR*: 1.13, 95% *CI* [1.02-1.26] | .079 |  |  |
|  | Post-acute COVID-19 Respiratory Sequelae: *n* = 206 | Patients after COVID-19 without Post-acute COVID-19 Respiratory Sequelae (*n* = 1,074) |  |  |  |  |  |  |
|  |  |  |  |  | Depressive symptoms & presence of Post-acute COVID-19 Central Nervous System Sequelae:  Adjusted *RR*: 1.09, 95% *CI* [0.93-1.28] | .752 |  |  |
|  | Post-acute COVID-19 Central Nervous System Sequelae: *n* = 134 | Patients after COVID-19 without Post-acute COVID-19 Central Nervous System Sequelae (*n* = 1,146) |  |  |  |  |  |  |
|  |  |  |  |  | Depressive symptoms & presence of Post-acute COVID-19 Fatigue Sequelae:  Adjusted *RR*: 1.21, 95% *CI* [1.06-1.38] | .029 |  |  |
|  |  |  |  |  | Depressive symptoms & presence of Post-acute COVID-19 Muscular Sequelae:  Adjusted *RR*: 1.26, 95% *CI* [0.95-1.66] | .259 |  |  |
|  | Post-acute COVID-19 Fatigue Sequelae: *n* = 108 | Patients after COVID-19 without Post-acute COVID-19 Fatigue Sequelae (*n* = 1,172) |  |  |  |  |  |  |
|  |  |  |  |  |  |  |  |  |
|  | Post-acute COVID-19 Muscular Sequelae: *n* = 34 | Patients after COVID-19 without Post-acute COVID-19 Muscular Sequelae (*n* = 1,246) |  |  |  |  |  |  |
| Lier et al. (2022) | *n* = 219 | – | Depression  $\tilde{X}$(Depression) = 8 (4 IQR -12)  Anxiety  $\tilde{X}$(Anxiety) = 6 (IQR 3-9) | Functional impairment (PCFS) $\tilde{X}$ = 2 (IQR 1-2) | Depression & functional impairment:  \|r\| = 0.59 | <.001 | There was a strong significant association of depression and anxiety with functional impairment |  |
|  |  |  |  |  | Anxiety & functional impairment:  \|r\| = 0.4 | <.001 |  |  |
| Loosen et al. (2022) | *n* = 1,708 | Patients after COVID-19 without Long COVID (*n* = 48,694) | Depression | Presence of Long COVID | Depression & Long COVID:  *OR*: 1.21, 95% *CI* [1.07-1.37] | .002 | Depression  in women was significantly associated with an increased likelihood of developing Long COVID |  |
| Luedke et al. (2023) | *n* = 34 | – | Depressive symptoms  *M*(Depression) = 59.25, *SD* = 7.0  Anxiety symptoms  *M*(Anxiety) = 70.70, *SD* = 17.79 | Functional impairment (IRS) = 2.41 (1.31) | Depression & functional impairment:  \|r\| = 0.51 | .006 | Low mood and anxiety significantly correlated with functional impairment and anxiety was significantly associated with functional impairment |  |
|  |  |  |  |  | Anxiety & functional impairment:  \|r\| = 0.67  β = 0.63 | <.001  .02 |  |  |
| Magel et al. (2023) | *n* = 58 | Patients after COVID-19 without Long COVID (*n* = 29) | Depression  Long COVID:  *M*(Depression) = 5.8, *SD* = 5.2  No Long COVID:  *M*(Depression) = 0.3, *SD* = 0.8  Anxiety/depression | Substantial fatigue at 3 months | Depression & Fatigue:  *OR*: 1.43, 95% *CI* [1.23-1.78] | <.001 | There were significant associations between depression as well as pre-existing anxiety/depression with fatigue at 3-month follow-up |  |
|  |  |  |  |  | Anxiety/depression & Fatigue:  *OR*: 3.70, 95% *CI* [1.96-8.04] | <.001 |  |  |
| Magnavita et al. (2023) | *n* = 115 | Patients after COVID-19 without Long COVID (*n* = 49) | Long COVID:  Anxiety  *M*(anxiety) = 3.00, *SD* = 2.83  Depression  *M*(depression) = 2.07, *SD* = 2.13  No Long COVID:  Anxiety  *M*(anxiety) = 1.59, *SD* = 2.26  Depression  *M*(depression) = 1.10, *SD* = 1.74 | Work ability (WAI) = 8.56 (2.01)  *Control* = 9.31 (1.36) | Anxiety & no. of Long COVID symptoms: *β* = 0.337, t = 0.641 | .005 | Anxiety was significantly associated with the no. of post-COVID symptoms, while depression was not |  |
|  |  |  |  |  | Depression & no. of Long COVID symptoms:  *β* = 0.111, t = 0.320 | .749 |  |  |
| Meza-Torres et al. (2022) | *n* = 7,623 | Patients after COVID-19 without Long COVID (*n* = 408,882) | Long COVID:  History of depression  % of patients with a history of depression: 36.2%  History of anxiety  % of patients with a history of anxiety: 34.8%  No Long COVID:  History of depression  % of patients with a history of depression: 22.8%  History of anxiety  % of patients with a history of anxiety: 23.3% | Presence of Long COVID | Depression & Long COVID:  *OR*: 1.55, 95% *CI* [1.47-1.64] | <.001 | Depression and anxiety were significantly associated with Long COVID |  |
|  |  |  |  |  | Anxiety & Long COVID:  *OR*: 1.35, 95% *CI* [1.28-1.43] | <.001 |  |  |
| Navas-Otero et al. (2023) | *n* = 68 | Patients after COVID-19 without Long COVID (*n* = 68)  Healthy controls without a history of COVID-19 (*n* = 68) | Physical activity  Long COVID:  *M*(physical activity) = 1037.47, *SD* = 960.78  Recovered:  *M*(physical activity) = 4732.45, *SD* = 3592.17  Healthy controls:  *M*(physical activity) = 7455.03, *SD* = 4736.34 | Symptom severity (C19-YRS) = 42.7 (0.36)  *Recovered* = 2.7 (11.8)  Functional disability (C19-YRS) = 20.8 (15.2)  *Recovered* = 5.1 (3.7)  Overall health (C19-YRS) = 4.6 (2.1)  *Recovered* = 1.7 (1.4)  Fatigue (fatigue-subscale of PROMIS-29) = 11.82 (5.03)  *Recovered* = 14.23 (3.58)  *HC* = 15.93 (3.05)  Weakness (5STS) = 14.23 (5.98)  *Recovered* = 8.01 (2.58)  *HC* = 7.73 (2.86) | Physical activity & symptom severity:  \|r\| = -0.189 | <.001 | Significant correlations between physical activity and symptom severity, functional disability, and overall health were found |  |
|  |  |  |  |  | Physical activity & functional disability:  \|r\| = -0.229 | <.001 |  |  |
|  |  |  |  |  | Physical activity & overall health:  \|r\| = 0.592 | <.001 |  |  |
| Nishimi et al. (2023) | *n* = 81 | Patients after COVID-19 without Long COVID (*n* = 447) | Psychological resilience | Presence of Long COVID (yes/no) | Resilience & Long COVID:  *RR*: 0.79, 95% *CI* [0.58-1.07] | .130 | Resilience was not significantly associated with the risk for reporting Long COVID |  |
| Pacho-Hernández et al. (2022) | *n* = 146 | – | Anxiety  *M*(Anxiety) = 5.3, *SD* = 4.2  Depression  *M*(Depression) = 5.0, *SD* = 4.3  Sensitisation  *M*(sensitisation) = 33.9, *SD* = 17.25 | HRQoL (EuroQol-5D) = 0.8 (0.2)  Sleep quality (PSQI) = 8.1 (4.3) | Anxiety & QoL:  *β* = -0.14 | .08 | Mediation models revealed that sensitisation-associated symptoms and depressive levels directly affected health-related quality of life; however, these effects were not statistically significant when sleep quality was included |  |
|  |  |  |  |  | Depression & QoL:  *β* = -0.17 | .03 |  |  |
|  |  |  |  |  | Sensitisation & QoL:  *β* = -0.19 | .01 |  |  |
| Pérez-López et al. (2023) | *n* = 164 | Patients after COVID-19 without Long COVID (*n* = 183) | Use of antidepressants  Use of anxiolytics  Resilience  Fear of COVID-19  Long COVID:  No. and % of patients with use of antidepressants: 26 (15.9%)  No. and % of patients with use of anxiolytics: 19 (11.6%)  No. and % of patients with resilience: 90 (54.9%)  No. and % of patients with fear of COVID-19: 90 (54.9%)  No Long COVID:  No. and % of patients with use of antidepressants: 25 (13.7%)  No. and % of patients with use of anxiolytics: 9 (4.9%)  No. and % of patients with resilience: 88 (48.1%)  No. and % of patients with fear of COVID-19: 90 (49.2%) | Presence of Long COVID (yes/no) | Use of antidepressants & Long COVID:  *OR*: 1.19, 95% *CI* [0.66-2.16] | – | In the logistic regression analysis, use of anxiolytics showed to be significantly associated with Long COVID whereas use of antidepressants, resilience, and fear of COVID-19 did not |  |
|  |  |  |  |  | Use of anxiolytics & Long COVID:  *OR*: 1.71, 95% *CI* [1.03-2.83] | – |  |  |
|  |  |  |  |  | Resilience & Long COVID:  *OR*: 1.31, 95% *CI* [0.86-2.00] | – |  |  |
|  |  |  |  |  | Fear of COVID-19 & Long COVID:  *OR*: 1.26, 95% *CI* [0.82-1.92] | - |  |  |
|  |  |  |  |  |  |  |  |  |
| Pływaczewska-Jakubowska et al. (2022) | *n* = 1,013 | Patients after COVID-19 without Long COVID (*n* = 504) | Physical activity  % of patients with regular physical activity: 52.8% of the Long COVID group | Presence of Long COVID | Regular physical activity & Long COVID signs and symptoms:  *β* = 0.86 (0.66 to 1.11) | .24 | Regular physical activity was not significantly related to Long COVID |  |
|  |  |  |  |  |  |  |  |  |
| Rocha et al. (2023) | *n =* 1,410 | Patients after COVID-19 without Long COVID (*n* = 1,509) | Physical activity  no data | Presence of Long COVID (yes/no) | Physical activity (became inactive) & Long COVID:  *PR*: 0.97, 95% *CI* [0.80-1.17] | – | In the poisson regression model, the likelihood of experiencing Long COVID symptoms was significantly reduced in those patients who remained active |  |
|  |  |  |  |  | Physical activity (remained active) & Long COVID:  *PR*: 0.80, 95% *CI* [0.63-0.99] | – |  |  |
|  |  |  |  |  |  |  |  |  |
| Ruggeri et al. (2023) | *n* = 18 | – | Mood disorders  *M(*Mood disorders) = 25.22, *SD* = 11.37 | Memory (summary score through a 4-point Likert scale; 0: normal, 1: mild deficit, 2: moderate deficit, 3: severe deficit) = 1.6 (0.9) | Mood disorders & memory:  *β* = 0.17, *t =* 0.77 | non sig | In the multivariate regression analysis, mood disorders were not significantly associated with cognitive dysfunctions |  |
| Salci et al. (2024) | *n* = 403 | – | Practice of physical activity  % of patients:  No = 62.5%  Yes = 36.0%  missing = 1.5%  Depressive mood  % of patients:  No = 84.1%  Yes = 15.9%  Anxiety symptoms  % of patients:  No = 85.9%  Yes = 14.1% | Perceived QoL (single-item visual analogue scale ranging from 1 – lowest possible QoL to 7 – highest possible QoL) = 5.2 (1.2); participants were divided into three categories: low (scores of 1-4), average (score of 5), and high (scores of 6 and 7); most participants (79.6%) had high QoL, followed by average QoL (10.7%) and low QoL (9.7%) | Practice of physical activity:  No & high QoL: 61.6%  No & moderate QoL: 67.4%  No & low QoL: 74.4%  Yes & high QoL: 38.4%  Yes & moderate QoL: 32.6%  Yes & low QoL= 25.6% | .2505 | Individuals with depressive mood and anxiety symptoms  were more likely to show lower quality of life |  |
|  |  |  |  |  | Depressive mood:  No & high QoL: 68.6%  No & moderate QoL: 74.4%  No & low QoL: 74.4%  Yes & high QoL: 13.4%  Yes & moderate QoL: 25.6%  Yes & low QoL: 25.6% | .0261 |  |  |
|  |  |  |  |  | Anxiety symptoms:  No & high QoL: 88.5%  No & moderate QoL: 76.7%  No & low QoL: 74.4%  Yes & high QoL: 11.5%  Yes & moderate QoL: 23.3%  Yes & low QoL: 25.6% | .0111 |  |  |
| Salve et al. (2023) | *n* = 77 | Patients after COVID-19 without Long COVID (*n* = 133) | Long COVID:  Depression  *M(*Depression) = 5.3, *SD* = 2.6  No Long COVID:  Depression  *M(*Depression) = 3.6, *SD* = 2.9 | Presence of Long COVID (yes/no) | Depression & Long COVID:  *OR*: 1.25, 95% *CI* [1.11-1.40] | .002 | In the multivariate model, depression showed to be significantly associated with Long COVID |  |
|  |  |  |  |  |  |  |  |  |
| Samper-Pardo et al. (2022) | *n* = 100 | – | Physical activity  *M*(physical activity) = 338.9, *SD* = 349.24    Depression and anxiety *M*(depression and anxiety) = 17.61, *SD* = 8.31 | QoL (SF-36) physical health = 32.19 (16.61); mental health = 34.77 (19.3) | Physical activity & physical QoL:  \|r\| = 0.139 | .168 | Physical activity significantly correlated with mental quality of life and depression and anxiety significantly correlated with mental and physical quality of life |  |
|  |  |  |  |  | Physical activity & mental QoL:  \|r\| = 0.203 | .042 |  |  |
|  |  |  |  |  | Depression and anxiety & physical QoL:  \|r\| = -0.472 | <.001 |  |  |
|  |  |  |  |  | Depression and anxiety & mental QoL:  \|r\| = -0.723 | <.001 |  |  |
| Satar et al. (2023) | *n* = 173 | – | Depression  *M*(Depression) = 6.83, *SD* = 3.41  Anxiety  *M*(Anxiety) = 7.20, *SD* = 3.87 | QoL (SF-36) physical health = 29.69 (12.61); mental health = 29.96 (14.51)  Fatigue (FSS) = 4.51 (1.78) | Depression & physical QoL:  \|r\| = -0.42  *β* = -0.21, *t* = -2.31 | <.001  .022 | In the regression analysis, depression and anxiety showed to be significantly related to quality of life |  |
|  |  |  |  |  | Depression & mental QoL:  \|r\| = -0.51  *β* = -0.25, *t* = -3.10 | <.001  .002 |  |  |
|  |  |  |  |  | Anxiety & physical QoL:  \|r\| = -0.45  *β* = -0.20, *t* = -2.14 | <.001  .034 |  |  |
|  |  |  |  |  | Anxiety & mental QoL:  \|r\| = -0.57  *β* = -0.37, *t* = -4.54 | <.001  <.001 |  |  |
| Scholz et al. (2023) | *n* = 253 | – | Depressive symptoms *M*(Depression) = 8.58; *SD* = 3.99  Anxiety *M*(Anxiety) = 7.92, *SD* = 4.09  Perceived stress  *M*(perceived stress) = 2.16, *SD* = 0.81 | SF-12  physical component score = 37.37 (9.31); mental component score = 35.54 (9.98) | Depressive symptoms & physical QoL:  \|r\| = -0.14 | <.05 | Depressive symptoms were significantly correlated with physical quality of life and mental quality of life, anxiety was significantly correlated with physical quality of life and mental quality of life, and stress was significantly correlated with mental quality of life |  |
|  |  |  |  |  | Depressive symptoms & mental QoL:  \|r\| = -0.58 | <.01 |  |  |
|  |  |  |  |  | Anxiety & physical QoL:  \|r\| = 0.15 | <.05 |  |  |
|  |  |  |  |  | Anxiety & mental QoL:  \|r\| = -0.53 | <.01 |  |  |
|  |  |  |  |  | Perceived stress & physical QoL:  \| r\| = -0.07 | Non sig |  |  |
|  |  |  |  |  | Perceived stress & mental QoL:  \|r\| = -0.42 | <.01 |  |  |
| Shachar-Lavie et al. (2023) | *n* = 87 | Healthy controls without a history of COVID-19 (*n* = 101) | Connection with friends  Long COVID:  *M*(connection with friends) = 0.39, *SD* = 0.63  No Long COVID:  *M*(connection with friends) = 0.36, *SD* = 0.66 | Functional impairment (CATS) = 1.75 (1.85)  *Control* = 2.08 (1.81) | Connection with friends & Long COVID:  *β* = 0.02 | non sig | Connection with friends was not significantly associated with Long COVID or functional impairment |  |
|  |  |  |  |  | Connection with friends & functional impairment:  *β* = -0.00 | non sig |  |  |
| Tavares-Júnior et al. (2022) | *n* = 141 | – | Depression  No. and % of patients with Depression: 57 (40%) | (ACE-R) = 82.5 (15.1)  (MMSE) = 27.4 (3.7)  Functional and cognitive impairment (Pfeffer Scale) = 1.7 (6.7)  Dyspnoea (MRC) = 0.6 (0.9)  Cognitive impairment (cognitive complaint confirmed by screening tests): *n* = 25 (34%)  Subjective cognitive decline (cognitive complaints in absence of objective impairment in screening tests): *n* = 68 (48.2%) | Depression & Cognition (SCD, CI, and normal):  X²-Test | .046 | A Chi-square test showed a significant difference regarding the proportions of depression between patients with SCD, CI, and unimpaired cognition (normal) |  |
| Tebeka et al. (2023) | *n* = 1,095 | Patients after COVID-19 without Long COVID (*n* = 1,021) | Long COVID:  Mean depression  *M*(Depression) = 3.55  Mean anxiety  *M*(Anxiety) = 3.65  Depression  % of patients with Depression: 22.2%, 95% CI [19.7-24.6]  Anxiety  % of patients with Anxiety: 23.3%, 95% CI [20.8-25.8]  Chronic depression  % of patients with chronic depression: 10.7%  Chronic anxiety  % of patients with chronic anxiety: 18.4%  No Long COVID:  Mean depression  *M*(Depression) = 3.17  Mean anxiety  *M*(Anxiety) = 3.28  Depression  % of patients with Depression: 18.0%, 95% CI [15.6-20.4]  Anxiety  % of patients with Anxiety: 17.6%, 95% CI [15.2-19.2]  Chronic depression  % of patients with chronic depression: 9.2%  Chronic anxiety  % of patients with chronic anxiety: 14.8% | Long COVID symptoms | Mean depression & no. of Long COVID symptoms:  \|r\| = 0.27 | <.001 | Levels of depression and anxiety were both strongly correlated with the no. of Long COVID symptoms and measured anxiety and chronic anxiety were significantly associated with Long COVID, while measured depression and chronic depression were not |  |
|  |  |  |  |  | Mean anxiety & no. of Long COVID symptoms:  \|r\| = 0.24 | <.001 |  |  |
|  |  |  |  |  | Depression & Long COVID | non sig |  |  |
|  |  |  |  |  | Anxiety & Long COVID:  Adjusted *OR*: 1.34, 95% *CI* [1.08-1.67] | sig |  |  |
|  |  |  |  |  | Chronic depression & Long COVID | non sig |  |  |
|  |  |  |  |  | Chronic anxiety & Long COVID:  Adjusted *OR*: 1.27, 95% *CI* [1.00-1.61] | .04 |  |  |
|  |  |  |  |  |  |  |  |  |
|  |  |  |  |  |  |  |  |  |
|  |  |  |  |  |  |  |  |  |
| Thronicke et al. (2022) | *n* = 99 | Patients after COVID-19 without Long COVID (*n* = 174) | COVID-19 Anxiety  no data | No. of Long COVID symptoms (in Long COVID group):  1-3 symptoms: 75%  4-6 symptoms: 21%  ≥7 symptoms: 4% | COVID-19-related anxiety & Long COVID:  *OR*: 8.28, 95% *CI* [1.43-47.85] | <.01 | Individuals reporting COVID-19 related anxiety were more likely to develop Long COVID |  |
| Tudor et al. (2023) | *n* = 105 | – | Self-compassion M(self-compassion) = 2.94, SD = 0.85  Psychological flexibility  M(psychological flexibility) = 19.7, SD = 5.32 | Symptom load (Symptom tool) = 28.8 (10.26)  Psychosocial impact (Impact tool) = 39.15 (14.31) | Self-compassion & symptom load:  \|r\| = -0.216 | <.05 | The correlation analysis revealed significant negative correlations of self-compassion and psychological flexibility with symptom load and psychosocial impact |  |
|  |  |  |  |  | Self-compassion & psychosocial impact:  \|r\| = -0.434 | <.001 |  |  |
|  |  |  |  |  | Psychological flexibility & symptom load:  \|r\| = -0.237 | <.05 |  |  |
|  |  |  |  |  | Psychological flexibility & psychosocial impact:  \|r\| = -0.401 | <.001 |  |  |
| Walker et al. (2023) | *n* = 3,754 | – | Depression *M*(Depression) = 11.8, *SD* = 6.0  Anxiety  *M*(Anxiety) = 9.0, *SD* = 5.9 | Functional limitation (WSAS) = 20.6 (9.9)  HRQoL (EQ-5D-5L) = 0.54 (0.27) | Depression & functional limitation:  *OR*: 1.05, 95% *CI* [1.03-1.08] | <.001 | There was a significant association between depression and functional limitation as well as significant associations of depression and anxiety with health-related quality of life |  |
|  |  |  |  |  | Anxiety & HRQoL:  *β* = -0.01 (-0.01 to -0.01) | <.0001 |  |  |
|  |  |  |  |  | Depression & HRQoL:  *β* = -0.01 (-0.01 to -0.01) | <.0001 |  |  |
| Whiteside et al. (2022) | *n* = 49 | – | Depression  *M*(Depression) = 19.88, *SD* = 9.82  Anxiety  *M*(Anxiety) = 14.94, *SD* = 10.77 | Word Reading (WRAT-4)  WAIS-IV  TMT  HVLT-R  WMS-IV  RCFT  NAB  Verbal Fluency (COWAT-FAS)  Animal Fluency  Stroop Test  WCST  Grooved Pegboard | Depression & no. of impaired cognitive scores per participant:  \|r\| = 0.48 | <.01 | There were significant correlations between mood/anxiety scores and cognitive measures |  |
|  |  |  |  |  | Anxiety & no. of impaired cognitive scores per participant:  \|r\| = 0.43 | <.01 |  |  |
|  |  |  |  | No. of impaired scores per participant = 4.92 (1.53) |  |  |  |  |
| Wright et al. (2024) | *n* = 10 | Patients after COVID-19 without Long COVID (n = 13) | Depression  Long COVID:  M(Depression) = 16.8, SD = 10.7  No Long COVID:  M(Depression) = 2.2, SD = 5.4 | Fatigue (MFSI-SF) = 31.0 (17.3)  M Control = -14.0 (7.4)  Quality of Life (QoL-AGHDA) = 13.6 (7.0)  M Control = 2.4 (5.1) | Depression & Fatigue:  \|r\| = -0.80 | .009 | The correlation analysis revealed significant correlations of depression with QoL and fatigue |  |
|  |  |  |  |  | Depression & QoL:  \|r\| = 0.73 | .012 |  |  |
| Yaksi et al. (2022) | *n* = 133 | – | Depression  no data for the whole sample | Presence of Long COVID (yes/no) | Depression score & Long COVID:  *OR*: 1.01, 95% *CI* [1.00-1.03] | .05 | In the logistic regression analysis, depression was not significantly associated with Long COVID |  |
| Zheng et al. (2023b) | *n* = 610 | Patients after COVID-19 without Long COVID (n = 833)  Healthy controls without a history of COVID-19 (n = 2,962) | Physical activity  Sedentary behaviour | Presence of Long COVID  Fatigue (yes/no) | Symptoms lasting >1 months:  Physical inactivity & Long COVID:  OR: 1.15, 95% CI [0.93-1.43]  Sedentary behaviour & Long COVID:  OR: 1.39, 95% CI [1.11-1.73]  Sedentary behaviour & Fatigue: | – | Prolonged sedentary behaviour (≥ 10 h/day) before COVID-19 was positively associated with an increased risk of symptoms of post-acute COVID-19 sequelae, while physical inactivity was not |  |
|  |  |  |  |  | OR: 1.34, 95% CI [1.07-1.67]  Symptoms lasting >2 months:  Physical inactivity & Long COVID:  OR: 1.05, 95% CI [0.85-1.30]  Sedentary behaviour & Long COVID:  OR: 1.25, 95% CI [1.01-1.55]  Sedentary behaviour & Fatigue:  OR: 1.05, 95% CI [0.81-1.36] |  |  |  |

*Note.* $\tilde{X}$ = median; 5STS = Five Sit-to-Stand Test; ACE-R = Addenbrooke’s Cognitive Examination-Revised; BNT = Boston Naming Test; C19-YRS = Yorkshire Rehabilitation Scale; CATS = Child and Adolescent Trauma Screen; CBT = Corsi Blocktapping Test; CI = confidence interval; CIS20 = Checklist Individual Strength; COWAT-FAS = Controlled Oral Word Association Test-FAS; DSST = Digit Symbol Substitution Test; EQ-5D-5L = European Quality of Life 5 Dimensions 5 Level Version; EuroQol EQ-5D-5L = European Quality of Life 5 Dimensions 5 Level Version; FACIT-Fatigue = Functional Assessment of Chronic Illness Therapy – Fatigue; FAS = Fatigue Assessment Scale; FCSRT = Free and Cued Selective Reminding Test; FLEI = Questionnaire for Complaints of Cognitive Disturbances; FSMC = Fatigue Scale for Motor and Cognitive Function; FSS = Fatigue Severity Score; GSRS = Gastrointestinal Symptom Rating Scale; HADS = Hospital Anxiety and Depression Scale; HRQoL = health-related quality of life; HVLT-R = Hopkins Verbal Learning Test-Revised; ICU = intensive care unit; IQR = interquartile range; IRS = Impairment Rating Scale; IRT = Item Response Theory; JLO = Judgment Line Orientation; *M* = Mean; ME/CFS = myalgic encephalomyelitis/chronic fatigue syndrome; MFIS = Modified Fatigue Impact Scale; MFSI-SF = Multidimensional Fatigue Symptom Inventory - Short Form; MMSE = Mini Mental State Examination; MRC = Medical Research Council - Dyspnea Scale; NAB = Neuropsychological Assessment Battery; No. = number; OR = odds ratio; PASC = post-acute sequelae of COVID-19; PCC = post COVID-19 condition; PCFS = Post-COVID-19 Functional Scale; PCS = Physical Component Scale; PHQ-15 = Patient Health Questionnaire-15; PROMIS = Patient-Reported Outcomes Measurement; PS Score = Performance Status Score; PSQI = Pittsburgh Sleep Quality index; PTSD = post-traumatic stress disorder; QoL = quality of life; QoL-AGHDA = Quality of Life-Assessment of Growth Hormone Deficiency in Adults; RCFT = Rey Complex Figure Test; ROCF = Rey-Osterrieth Complex Figure; RQ = research question; RR = risk ratio; SCD = subjective cognitive decline; SCWT = Stroop Color and Word Test; *SD* = standard deviation; SDMT = Symbol Digit Modalities Test; SF-12 = 12-Item Short Form Survey; SF-36 = 36-Item Short Form Health Survey; SOFA/GP = Schedule of Fatigue and Anergia/General Practice; SVFT = Semantic Verbal Fluency Test; TMT = Trail Making Test; VAS = Visual Analogue Scale; VOSP = Visual Object and Space Perception Battery; WAI = Work Ability Index; WAIS-IV = Wechsler Adult Intelligence Scale-4; WCST = Wisconsin Card Sorting Test; WMS-IV = Wechsler Memory Scale-4; WRAT-4 = Wide Range Achievement Test-4; WSAS = Work and Social Adjustment Scale.

## **eTable 4. Overview of included studies providing longitudinal data on psychological variables (RQ3)**

| **Author**  **(year of**  **publication)** | **Psychological**  **variable(s)** | **Condition-relevant**  **outcome(s)**  **(measuring instrument; Mean (*SD*))**  if not stated otherwise | **Long COVID**  **(sample size)** | **Baseline**  **Long COVID Mean (*SD*)**  if not stated otherwise | **Main results**  **(regression coefficients)**  if not stated otherwise | **Significant**  **difference**  **(*p*-value)** | **Comments** |
| --- | --- | --- | --- | --- | --- | --- | --- |
| Antony et al. (2023) | Pre-existing depression | Presence of Long COVID | *n* = 17,036 (No Long COVID: *n* = 2,173,543) | % of patients with pre-existing depression: 11.63% | Pre-existing depression significantly contributed to the prediction of Long COVID with an importance rank of 7 out of 37 features;  Iteration 1: 0.18239  Iteration 2: 0.15788  Iteration 3: 0.12069  Iteration 4: 0.14649  Iteration 5: 0.16802  Iteration 6: 0.16599  Iteration 7: 0.15110  Iteration 8: 0.14434  Iteration 9: 0.14115  Iteration 10: 0.12718 | sig | Importance of depression for the prediction of Long COVID computed using the SHAP (SHapely Additive exPlanations) algorithm for a machine learning model (logistic regression model) in ten iterations |
| Binka et al. (2022) | Depressive disorders | Presence of Long COVID | *n* = 2,430 (No Long COVID: *n* = 24,300) | Long COVID:  No. and % of patients with depressive disorders: 1,055 (43.4%)  No Long COVID:  No. and % of patients with depressive disorders: 6,191 (25.5%) | Depressive disorders & Long COVID:  *OR*: 2.02 (95% *CI* not reported) | – |  |
| Bottemanne et al. (2021) | Depression; Anxiety | Fatigue (clinical examination): *n* = 38 (IQR 46.3)  Dyspnoea (clinical examination): *n* = 39 (IQR 46.4)  Pain complaints (clinical examination): *n* = 26 (IQR 31.3) | *n* = 84 (all patients after COVID-19) | Anxiety  $\tilde{X}$(all patients) = 6 (IQR 4-8)  Depression  $\tilde{X}$(all patients) = 4 (IQR 2-6.5) | Depression & Fatigue:  *OR*: 0.84, 95% *CI* [0.60-1.17]  Depression & Dyspnoea:  *OR*: 0.80, 95% *CI* [0.57-1.12]  Depression & pain complaints:  *OR*: 0.93, 95% *CI* [0.77-1.13]  Anxiety & Fatigue:  *OR*: 0.81, 95% *CI* [0.57-1.15]  Anxiety & Dyspnoea:  *OR*: 0.93, 95% *CI* [1.69-1.27]  Anxiety & pain complaints:  *OR*: 0.99, 95% *CI* [0.84-1.17] | .307  .199  .466  .250  .664  .925 |  |
| Chen et al. (2023a) | Pre-existing history of Depression; pre-existing history of Anxiety; pre-existing history of OCD; pre-existing history of ADHD | Long COVID symptoms (standardised intake surveys and physician documentation) = 15.8 (7.0)  Fatigue: 91 (98%)  Sleep disturbances: 62 (67%)  Cognitive difficulties: 83 (89%)  Dizziness/ light-headedness: 69  (74%) | *n* = 93 | No. and % of patients with pre-existing history of Depression: 12 (13%)  No. and % of patients with pre-existing history of Anxiety: 27 (29%)  No. and % of patients with pre-existing history of  OCD: no exact data reported  No. and % of patients with pre-existing history of  ADHD: 16 (17%) | Pre-existing mood disorders (Depression, Anxiety or OCD) & fatigue:  *OR*: 1.1, 95% *CI* [0.08-inf]  Pre-existing mood disorders (Depression, Anxiety or OCD) & change in appetite:  *OR*: 2.7, 95% *CI* [1.02-7.6]  Pre-existing mood disorders (Depression, Anxiety or OCD) & sleep disturbance:  *OR*: 3.0, 95% *CI* [0.96-11.5]  Pre-existing mood disorders (Depression, Anxiety or OCD) & concentration difficulties/brain fog/memory loss:  *OR*: 1.8, 95% *CI* [0.33-18.7]  Pre-existing mood disorders (Depression, Anxiety or OCD) &  dizziness/light-headedness/vertigo:  *OR*: 6.1, 95% *CI* [1.3-57.4]  Pre-existing ADHD & concentration difficulties/brain fog/memory loss:  *OR*: 1.7, 95% *CI* [0.20-81.9] | .97  .04  .06  .70  .02  1 |  |
| Durstenfeld et al. (2023) | Anxiety; Depression | Presence of at least one Long COVID symptom | *n* = 476 (No Long COVID: *n* = 1,004) | Long COVID:  *M*(Anxiety) = 5.04, *SD* = 4.66  *M*(Depression) = 5.85, *SD* = 4.91  No Long COVID:  *M*(Anxiety) = 3.08, *SD* = 3.60  *M*(Depression) = 3.31, *SD* = 3.74 | Anxiety & Long COVID:  *OR*: 1.04, 95% *CI* [0.97-1.12]  Depression & Long COVID:  *OR*: 1.08, 95% *CI* [1.01-2.16] | .25  .03 |  |
| Gaspar et al. (2023) | Pre-existing Depression/Anxiety | Presence of at least one persistent symptom (self-developed questionnaire): 66.5% (3MFU), 62.4% (6MFU) and 53.6% (9MFU)  HRQoL (EQ-5D-3L): 65.8% at least some impairment in some domain at 3MFU, 69.2% at 6MFU and 55.4% at 9MFU  HRQoL (EQ-VAS) | At 3MFU: *n* = 101 (No Long COVID: *n* = 51)  At 6MFU: *n* = 73 (No Long COVID: *n* = 44)  At 9MFU: *n* = 59 (No Long COVID: *n* = 51) | Long COVID:  No. and % of patients with pre-existing Depression/Anxiety at 3MFU: 21 (20.8%)  No. and % of patients with pre-existing Depression/Anxiety at 6MFU: 15 (20.6%)  No. and % of patients with pre-existing Depression/Anxiety at 9MFU: 11 (18.6%)  No Long COVID:  No. and % of patients with pre-existing Depression/Anxiety at 3MFU: 9 (17.7%)  No. and % of patients with pre-existing Depression/Anxiety at 6MFU: 10 (22.7%)  No. and % of patients with pre-existing Depression/Anxiety at 9MFU: 11 (21.6%) | Depression/Anxiety at 3MFU & Long COVID:  *OR*: 1.23, 95% *CI* [0.52-2.92]  Depression/Anxiety at 6MFU & Long COVID:  *OR*: 0.88, 95% *CI* [0.36-2.17]  Depression/Anxiety at 9MFU & Long COVID:  *OR*: 0.83, 95% *CI* [0.33-2.12] | .646  .781  .702 |  |
| Hill et al. (2023) | Depression; psychosis; substance abuse; tobacco smoker | Presence of Long COVID (diagnosis) | *n* = 7,512 (No Long COVID: *n* = 37,560) | Long COVID:  No. and % of patients with Depression: 1,946 (25.9%)  No. and % of patients with psychosis: 61 (0.8%)  No. and % of patients with substance abuse: 196 (2.6%)  No. and % of tobacco smokers: 504 (6.7%)  No Long COVID:  No. and % of patients with Depression: 7,430 (19.8%)  No. and % of patients with psychosis: 435 (1.2%)  No. and % of patients with substance abuse: 1,424 (3.8%)  No. and % of tobacco smokers: 3,052 (8.1%) | Depression & Long COVID:  *OR*: 1.28, 95% *CI* [1.20-1.37]  Psychosis & Long COVID:  *OR*: 0.56, 95% *CI* [0.42-0.76]  Substance abuse & Long COVID:  *OR*: 0.65, 95% *CI* [0.55-0.76]  Tobacco smokers & Long COVID:  *OR*: 0.69, 95% *CI* [0.62-0.76] | –  –  –  – |  |
| Jacobs et al. (2023) | Pre-existing Depression/Anxiety | Presence of Long COVID (self-developed symptom questions) | *n* = 518 (No Long COVID: *n* = 706) | Long COVID:  No. and % of patients with pre-existing Depression/Anxiety: 71 (13.7%)  No Long COVID:  No. and % of patients with pre-existing Depression/Anxiety: 56 (7.9%) | Depression/Anxiety & Long COVID:  Adjusted *OR*: 1.72, 95% *CI* [1.17-2.52] | <.05 |  |
| Kostev et al. (2022a) | Pre-existing Depression; pre-existing Anxiety disorders; pre-existing reaction to severe stress and adjustment disorders; pre-existing nicotine dependence | Presence of Long COVID (diagnosis): 8.3% | *n* = 4,699  (No Long COVID: *n* = 46,931)  *n* = 5,783  (No Long COVID: *n* = 45,847)  *n* = 5,835  (No Long COVID: *n* = 45,795)  *n* = 5,318  (No Long COVID: *n* = 46,312) | % of patients with pre-existing Depression: 18.6% of all patients after COVID-19  % of patients with pre-existing Anxiety disorders: 7.2% of all patients after COVID-19  % of patients with pre-existing reaction to severe stress and adjustment disorders: 13.6% of all patients after COVID-19  % of patients with pre-existing nicotine dependence: 4.5% of all patients after COVID-19 | Depression & Long COVID:  Adjusted *OR*: 0.95, 95% *CI* [0.84-1.08]  Anxiety & Long COVID:  Adjusted *OR*: 1.13, 95% *CI* [0.96-1.33]  Reaction to severe stress and adjustment disorders & Long COVID:  Adjusted *OR*: 1.24, 95% *CI* [1.10-1.41]  Nicotine dependence & Long COVID:  Adjusted *OR*: 0.96, 95% *CI* [0.78-1.19] | .433  .152  <.001  .705 |  |
| Kostev et al. (2022b) | Pre-existing Anxiety disorders; pre-existing reaction to severe stress and adjustment disorders; pre-existing disorders of psychological development | Presence of Long COVID (diagnosis): 1.7% | *n* = 114 (No Long COVID: *n* = 6,454) | Long COVID:  No. and % of patients with Anxiety disorders: 6 (5.3%)  No. and % of patients with reaction to severe stress and adjustment disorders: 3 (2.6%)  No. and % of patients with disorders of psychological development: 16 (14.0%)  No Long COVID:  No. and % of patients with Anxiety disorders: 77 (1.2%)  No. and % of patients with reaction to severe stress and adjustment disorders: 122 (1.9%)  No. and % of patients with disorders of psychological development: 1,016 (15.7%) | Anxiety & Long COVID:  Adjusted *RR*: 2.53, 95% *CI* [1.05-6.11]  Reaction to severe stress and adjustment disorders & Long COVID:  Adjusted *RR*: 0.82, 95% *CI* [0.25-2.74]  Disorders of psychological development & Long COVID:  Adjusted *RR*: 0.83, 95% *CI* [0.29-2.41] | .038  .752  .729 |  |
| Liu et al. (2023) | History of depression; history of anxiety disorder | Baseline functional activity status Moderate = 45.7%  *Control* = 49.0%  Able to climb 1 flight stairs or walk 1 block = 22.4%  *Control* = 21.5%  Able to carry groceries, bathe, or dress = 5.4%  *Control* = 4.6%  Perceived cognitive deficit score (PDQ) 0 = 47.1%  *Control* = 70.9%  >0 to 1.5 = 28.7%  *Control* = 18.4%  >1.5 to 4 = 24.2%  *Control* = 10.7% | *n* = 223 (No Long COVID: *n* = 543) | Long COVID:  % of patients with a history of depression: 26.5%  % of patients with a history of anxiety disorder: 32.3%  No Long COVID:  % of patients with a history of depression: 17.3%  % of patients with a history of anxiety disorder: 26.0% | Depression & Long COVID:  *OR*: 1.12, 95% *CI* [0.70-1.79]  Anxiety & Long COVID:  *OR*: 1.25, 95% *CI* [0.82-1.88] | –  – |  |
| Mazza et al. (2022) | Depression (BDI-13 mean score at 1MFU); Depression (ZSDS index mean score at 1MFU); positive psychiatric history | Presence of Long COVID | *n* = 101 (No Long COVID: *n* = 199) | Long COVID:  *M*(Depression BDI-13) = 4.713, *SD* = 4.64  *M*(Depression ZSDS index) = 48.084, *SD* = 12.985  % of patients with positive psychiatric history: 33%  No Long COVID:  *M*(Depression BDI-13) = 2.533, *SD* = 3.078  *M*(Depression ZSDS index) = 41.866, *SD* = 9.467)  % of patients with positive psychiatric history: 19% | Elastic net coefficients:  0.2010 (0.1175)  0.1659 (0.1065)  0.0307 (0.0310) | Variable inclusion probability  94.1 (= yes)  92.0 (= yes)  73.1 (= no) |  |
| Milde et al. (2023) | Fear of COVID-related health consequences; positive and negative trait affect; anxiety and depression; chronic stress | Long COVID symptom impairment | *n* = 91 | – | Fear of COVID-related health consequences was related to a higher magnitude of COVID-related symptom impairment at T0 (*b* = 0.25) and remained high at T1 (*b* = 0.3); however, the influence of fear of COVID-related health consequences dropped at T2 (*b* = 0.17);  fear of COVID-related health consequences predicted higher odds of any reporting COVID-related symptoms at T1 (*OR:* 9.01)  and T2 (*OR:* 9.26) but not reliably at  T0 (*OR*: 1.33);  explorative:  Depression (*b* = 0.25) and  chronic stress (*b* = 0.20) were related to higher COVID-related symptom impairment, whereas  trait positive affect was related to lower COVID-related symptom impairment (*b* = −0.17);  Depression (*OR:* 3.03) and  chronic stress (*OR:* 2.50) were related to higher odds of reporting any COVID symptoms, whereas  trait positive affect was related to lower odds of reporting any COVID symptoms (*OR:* 0.47) | 95% *CI* = 0.11 to 0.39  95% *CI* = 0.13 to 0.51  95% *CI* = -0.06 to 0.41  95% *CI* = 3.08 to 34.91  95% *CI* = 2.15 to 82.63  95% *CI* = 0.41 to 4.88  95% *CI* = 0.12 to 0.38  95% *CI* = 0.08 to 0.32  95% *CI* = -0.29 to -0.05  95% *CI* = 1.37 to 7.69  95% *CI* = 1.23 to 5.88  95% *CI* = 0.20 to 0.96 |  |
| Selvakumar et al. (2023) | Physical activity prior to infection; loneliness; negative life events prior to last year | QoL (PedsQL; range 0-100) SARS-CoV-2 positives = 78.3 (66.3-88.0); SARS-CoV negatives = 76.1 (67.9-86.4) at 6MFU | *n* = 191 (Postinfective fatigue syndrome: *n* = 48; No Long COVID: *n* = 85) | – | Physical activity & Long COVID:  *RR*: 0.96, 95% *CI* [0.92-1.00]  Loneliness & Long COVID:  *RR*: 1.01, 95% *CI* [1.00-1.02]  Negative life events & postinfective fatigue syndrome:  *RR*: 0.88, 95% *CI* [0.80-0.96] | .03  .01  .004 |  |
| Subramanian et al. (2022) | Depression; Anxiety | Presence of Long COVID | *n* = 29,869 (No Long COVID: *n* = 354,268) | Long COVID:  No. and % of patients with Depression: 11,222 (37.6%)  No. and % of patients with Anxiety: 10,481 (35.1%)  No Long COVID:  No. and % of patients with Depression: 72,681 (20.5%)  No. and % of patients with Anxiety: 67,272 (19.0%) | Depression & Long COVID:  Adjusted *HR*: 1.31, 95% *CI* [1.27-1.34]  Anxiety & Long COVID:  Adjusted *HR*: 1.35, 95% *CI* [1.31-1.39] | sig  sig |  |
| Wang et al. (2022) | Probable depression; probable anxiety; worry about COVID-19; perceived stress; loneliness | Presence of Long COVID (yes/no):  *n* = 1403 (43.9%)  Any post-COVID-19 related daily life impairment (yes/no):  *n* = 783 (55.8%) | *n* = 1,403 (No Long COVID: *n* = 1,790) | Long COVID:  No. and % of patients with probable depression: 212 (15.1%)  No. and % of patients with probable anxiety: 345 (24.6%)  No. and % of patients with worry about COVID-19: 264 (18.8%)  No. and % of patients with perceived stress Q1: 188 (13.4%)  No. and % of patients with perceived stress Q2: 166 (11.8%)  No. and % of patients with perceived stress Q3: 135 (9.6%)  No. and % of patients with perceived stress Q4: 184 (13.1%)  No. and % of patients with loneliness: 231 (16.5%)  No Long COVID:  No. and % of patients with probable depression: 397 (22.2%)  No. and % of patients with probable anxiety: 651 (36.4%)  No. and % of patients with worry about COVID-19: 496 (27.7%)  No. and % of patients with perceived stress Q1: 514 (36.6%)  No. and % of patients with perceived stress Q2: 432 (24.1%)  No. and % of patients with perceived stress Q3: 315 (17.6%)  No. and % of patients with perceived stress Q4: 358 (20.0%)  No. and % of patients with loneliness: 456 (25.5%) | Probable depression & Long COVID:  *RR*: 1.32, 95% *CI* [1.12-1.55]  Probable anxiety & Long COVID:  *RR*: 1.42, 95% *CI* [1.23-1.65]  Worry about COVID-19 & Long COVID:  *RR*: 1.37, 95% *CI* [1.71-1.16]  Perceived stress & Long COVID:  *RR*: 1.46, 95% *CI* [1.18-1.81]  Loneliness & Long COVID:  *RR*: 1.32, 95% *CI* [1.08-1.61]  Probable depression & daily life impairment:  *RR*: 1.51, 95% *CI* [1.23-1.85]  Probable anxiety & daily life impairment:  *RR*: 1.44, 95% *CI* [1.18-1.75]  Worry about COVID-19 & daily life impairment:  *RR*: 1.25, 95% *CI* [1.01-1.54]  Perceived stress & daily life impairment:  *RR*: 1.38, 95% *CI* [1.05-1.83]  Loneliness & daily life impairment:  *RR*: 1.15, 95% *CI* [0.89-1.28] | –  –  –  –  –  –  –  –  –  – |  |
| Zhang et al. (2023a) | Depression; Anxiety | Fatigue (yes/no): 81.2%  Breathlessness (yes/no): 60.9%  Cognitive difficulty (yes/no): 56%  Other new symptoms (yes/no): 46.1% | *n* = 168 (No Long COVID: *n* = 39) | Long COVID:  % of patients with moderate/severe depression: 24.2%  % of patients with moderate/severe anxiety: 19.3% | Moderate/severe depression & fatigue:  *OR*: 2.97, 95% *CI* [1.06-8.29]  Moderate/severe anxiety & cognitive difficulty:  *OR*: 3.30, 95% *CI* [1.16-9.40] | .038  .026 | Only significant associations reported |
| Zheng et al. (2023a) | Pre-existing depression or anxiety | Dyspnoea (MRC Dyspnoea score) at 5MFU:  2: 34.9%  3: 30.8%  4: 20.9%  5: 13.4% | *n* = 990 | % of patients with depression or anxiety at 1YFU: 19.7% | Depression or anxiety & worsening of dyspnoea at 1YFU:  *OR*: 1.51, 95% *CI* [1.05-2.17] | – |  |

*Note.* $\tilde{X}$ = median; ADHD = attention deficit hyperactivity disorder; BDI-13 = Beck Depression Inventory-13; CI = confidence interval; EQ-5D-3L = European Quality of Life 5 Dimensions 3 Level Version; EQ-VAS = EuroQoL-Visual Analogue Scale; HR = hazard ratio; HRQoL = health-related quality of life; IQR = interquartile range; *M* = Mean; MFU = month follow-up; MRC Dyspnoea Scale = Medical Research Council Dyspnoea Scale; No. = number; OCD = obsessive-compulsive disorder; OR = odds ratio; PDQ = Perceived Deficits Questionnaire; PedsQL = Pediatric Quality of Life Inventory; QoL = quality of life; RQ = research question; RR = risk ratio; *SD* = standard deviation; YFU = year follow-up; ZSDS = Zung Self-Rating Depression Scale.

## **eTable 5. Psychological variables, respective category, and number of studies in which they were investigated by RQ**

| **Psychological variable** | **Number RQ1** | **Number RQ2** | **Number RQ3** | **Category** |
| --- | --- | --- | --- | --- |
| Depression | 45 | 28 | 12 | Psychopathology |
| Anxiety | 35 | 23 | 8 | Psychopathology |
| Physical activity | 8 | 7 | 1 | Behavioural |
| Depression/Anxiety | 9 | 4 | 3 | Psychopathology |
| PTSD | 7 | 3 |  | Psychopathology |
| Stress | 8 | 1 | 2 | Psychophysiological |
| History of mental health disorders | 5 | 3 | 1 | Psychopathology |
| Alcohol abuse/dependence | 3 |  |  | Psychopathology |
| Suicidal ideation | 3 | 1 |  | Psychopathology |
| Mood disorders | 2 | 1 | 1 | Psychopathology |
| Reaction to severe stress and adjustment disorders | 2 |  | 2 | Psychopathology |
| Mania | 1 |  |  | Psychopathology |
| Schizophrenia/Psychosis/Paranoia | 1 |  | 1 | Psychopathology |
| ADHD |  |  | 1 | Psychopathology |
| Disorders of psychological development | 1 |  | 1 | Psychopathology |
| Nicotine abuse/dependence |  |  | 2 | Psychopathology |
| Drug problems/substance abuse | 1 |  | 1 | Psychopathology |
| Borderline features | 1 |  |  | Psychopathology |
| Antisocial features | 1 |  |  | Psychopathology |
| Thought disorder | 1 |  |  | Psychopathology |
| Depression medications | 1 | 1 |  | Psychopathology |
| Anxiety medications |  | 1 |  | Psychopathology |
| Loneliness |  |  | 2 | Affective |
| Anger | 1 |  |  | Affective |
| Positive trait affect |  |  | 1 | Affective |
| Fear of COVID-19 | 2 | 2 | 1 | Affective |
| Maternal health anxiety | 1 |  |  | Affective |
| Worry about COVID-19 |  |  | 1 | Cognitive |
| Catastrophising | 2 | 1 |  | Cognitive |
| Negative cognitions | 1 |  |  | Cognitive |
| Personal control |  | 1 |  | Cognitive |
| Treatment control |  | 1 |  | Cognitive |
| Treatment rejection | 1 |  |  | Cognitive |
| Coherence |  | 1 |  | Cognitive |
| Self-compassion |  | 1 |  | Cognitive |
| Emotional representation |  | 1 |  | Cognitive |
| Illness identity |  | 1 |  | Cognitive |
| Walking self-efficacy | 1 |  |  | Cognitive |
| Fear avoidance/Kinesiophobia | 2 | 1 |  | Behavioural |
| Avoidance | 1 |  |  | Behavioural |
| Connection with friends | 1 | 1 |  | Behavioural |
| Sedentary behaviour |  | 1 |  | Behavioural |
| Neuroticism/emotional instability | 2 | 1 |  | Personality & Interpersonal factors |
| Extraversion | 1 | 1 |  | Personality & Interpersonal factors |
| Openness | 1 | 1 |  | Personality & Interpersonal factors |
| Conscientiousness | 1 | 1 |  | Personality & Interpersonal factors |
| Agreeableness | 1 | 1 |  | Personality & Interpersonal factors |
| Resilience | 2 | 2 |  | Personality & Interpersonal factors |
| Psychological flexibility |  | 1 |  | Personality & Interpersonal factors |
| Aggression | 1 |  |  | Personality & Interpersonal factors |
| Dominance | 1 |  |  | Personality & Interpersonal factors |
| Warmth | 1 |  |  | Personality & Interpersonal factors |
| Stigma | 1 |  |  | Personality & Interpersonal factors |
| Life events | 1 |  | 1 | Prior experiences |
| Nonsupport | 1 |  |  | Prior experiences |
| Psychological distress | 2 | 1 |  | Psychophysiological |
| Central sensitisation | 1 | 2 |  | Psychophysiological |
| Multisensory sensitivity/somatosensory amplification | 1 |  |  | Psychophysiological |

*Note.* Variables with at least five observations are shaded grey; ADHD = attention deficit hyperactivity disorder; PTSD = post-traumatic stress disorder; RQ = research question.

## **eTables 6-12. Operationalisation of psychological variables with at least five observations in studies answering RQ1 and respective control groups**

**eTable 6. Operationalisation of depression in studies answering RQ1 and respective control groups**

| **Instrument** | **Reference(s)** | **Control group(s)** |
| --- | --- | --- |
| No. or % of patients with depression based on cut-off score |  |  |
| PHQ-2 | ^40^ | CO |
|  | ^79^ | CO |
|  | ^98^ | CO |
| PHQ-9 | ^19^ | CO, HC |
|  | ^25^ | CO, HC |
|  | ^50^ | CO |
|  | ^109^ | CO |
| BDI-13 | ^112^ | CO |
| ZSDS index | ^112^ | CO |
| DASS-21 | ^80^ | CO |
| No. or % of patients with pre-existing depression (diagnosis) | ^62^ | CO |
|  | ^94^ | CO |
| No. or % of patients with pre-existing depression (self-report yes/no) | ^3^ | CO |
|  | ^35^ | CO, HC |
|  | ^60^ | HC |
|  | ^71^ | CO |
|  | ^98^ | CO |
| No. or % of patients with self-reported symptoms of depression (yes/no) | ^2^ | CO |
|  | ^8^ | CO |
|  | ^35^ | CO, HC |
|  | ^74^ | CO, HC |
| PHQ-9 | ^5^ | HC |
|  | ^58^ | HC |
|  | ^59^ | CO |
|  | ^65^ | CO |
|  | ^67^ | CO |
|  | ^68^ | HC |
|  | ^76^ | HC |
|  | ^88^ | CO |
|  | ^95^ | CO, HC, acute |
| HADS | ^7^ | CO |
|  | ^32^ | CO |
|  | ^42^ | CFS, FMS |
|  | ^69^ | HC |
|  | ^99^ | CO |
| PHQ-2 | ^42^ | CFS, FMS |
|  | ^73^ | CO, HC |
|  | ^98^ | CO |
| BDI-II | ^46^ | HC |
|  | ^106^ | CO |
| HAMD | ^1^ | HC |
| PHQ-8 | ^30^ | HC |
| GADS | ^66^ | CO |
| DASS-21 | ^20^ | CO, HC |
| GDS | ^6^ | ME/CFS |
| Neuro-QoL | ^35^ | CO, HC |
| POMS | ^22^ | HC |
| PROMIS | ^60^ | HC |
| Incidence rate | ^84^ | HC |
| Self-perceived intensity | ^72^ | HC |
| PAI | ^36^ | PCS |

*Note.* acute = patients with acute COVID-19; BDI-13 = Beck Depression Inventory-13; CO = patients after COVID-19 without Long COVID; DASS-21 = Depression, Anxiety and Stress Scale; FMS = patients with fibromyalgia syndrome; GADS = Goldberg Anxiety and Depression Scale; GDS = Geriatric Depression Scale; HADS = Hospital Anxiety and Depression Scale; HAMD = Hamilton Depression Scale; HC = healthy controls without a history of COVID-19; ME/CFS = patients with myalgic encephalomyelitis/chronic fatigue syndrome; Neuro-QOL = Quality of Life in Neurological Disorders; No. = number; PAI = Personality Assessment Inventory; PCS = patients with post-concussion syndrome; PHQ-2 = Patient Health Questionnaire-2; PHQ-8 = Patient Health Questionnaire-8; PHQ-9 = Patient Health Questionnaire-9; POMS = Profile of Mood States; PROMIS = Patient-Reported Outcomes Measurement; RQ = research question; ZSDS = Zung Self-Rating Depression Scale.

**eTable 7. Operationalisation of anxiety in studies answering RQ1 and respective control groups**

| **Instrument** | **Reference(s)** | **Control group(s)** |
| --- | --- | --- |
| No. or % of patients with anxiety based on cut-off score |  |  |
| GAD-2 | ^40^ | CO |
|  | ^79^ | CO |
|  | ^98^ | CO |
| GAD-7 | ^19^ | CO, HC |
|  | ^25^ | CO, HC |
|  | ^50^ | CO |
|  | ^109^ | CO |
| DASS-21 | ^80^ | CO |
| No. or % of patients with pre-existing anxiety (diagnosis) | ^57^ | CO |
|  | ^62^ | CO |
| No. or % of patients with pre-existing anxiety (self-report yes/no) | ^3^ | CO |
|  | ^35^ | CO, HC |
|  | ^60^ | HC |
|  | ^71^ | CO |
|  | ^98^ | CO |
| No. or % of patients with self-reported anxiety symptoms (yes/no) | ^2^ | CO |
|  | ^8^ | CO |
|  | ^35^ | CO, HC |
| GAD-7 | ^5^ | HC |
|  | ^30^ | HC |
|  | ^68^ | HC |
|  | ^76^ | HC |
|  | ^95^ | CO, HC, acute |
| HADS | ^7^ | CO |
|  | ^32^ | CO |
|  | ^42^ | CFS, FMS |
|  | ^99^ | CO |
| GAD-2 | ^73^ | CO, HC |
|  | ^98^ | CO |
| HAMA | ^1^ | HC |
| BAI | ^46^ | HC |
| GADS | ^66^ | CO |
| DASS-21 | ^20^ | CO, HC |
| STAI | ^6^ | ME/CFS |
| Neuro-QoL | ^35^ | CO, HC |
| PROMIS | ^60^ | HC |
| Incidence rate | ^84^ | HC |
| Ordinal scale | ^70^ | CO |
| Self-perceived intensity | ^72^ | HC |
| PAI | ^36^ | PCS |

*Note.* acute = patients with acute COVID-19; BAI = Beck Anxiety Inventory; CO = patients after COVID-19 without Long COVID; DASS-21 = Depression, Anxiety and Stress Scale; FMS = patients with fibromyalgia syndrome; GAD-2 = Generalized Anxiety Disorder Scale-2; GAD-7 = Generalized Anxiety Disorder Scale-7; GADS = Goldberg Anxiety and Depression Scale; HADS = Hospital Anxiety and Depression Scale; HAMA = Hamilton Anxiety Rating Scale; HC = healthy controls without a history of COVID-19; ME/CFS = patients with myalgic encephalomyelitis/chronic fatigue syndrome; Neuro-QOL = Quality of Life in Neurological Disorders; No. = number; PAI = Personality Assessment Inventory; PCS = patients with post-concussion syndrome; PROMIS = Patient-Reported Outcomes Measurement; RQ = research question; STAI = State-Trait-Anxiety Inventory.

**eTable 8. Operationalisation of physical activity in studies answering RQ1 and respective control groups**

| **Instrument** | **Reference(s)** | **Control group(s)** |
| --- | --- | --- |
| No. or % of patients with regular physical activity (defined as at least 150-300 min per week of moderate-intensity activity or 75-150 min per week of high-intensity activity) | ^81^ | CO |
| No. or % of patients with physical activity level not meeting guidelines (self-report yes/no) | ^82^ | CO |
| IPAQ | ^20^ | CO, HC |
|  | ^74^ | CO, HC |
| GSLTPAQ | ^22^ | HC |
| Mean (*SD*), minutes/week | ^67^ | CO |
| Mean (*SD*), days/week | ^30^ | HC |
| Single item | ^93^ | HC |

*Note.* CO = patients after COVID-19 without Long COVID; GSLTPAQ = Godin-Shephard Leisure-Time PA; HC = healthy controls without a history of COVID-19; IPAQ = International Physical Activity Questionnaire; No. = number; RQ = research question; *SD* = standard deviation.

**eTable 9. Operationalisation of depression/anxiety in studies answering RQ1 and respective control groups**

| **Instrument** | **Reference(s)** | **Control group(s)** |
| --- | --- | --- |
| No. or % of patients with depression/anxiety based on cut-off score |  |  |
| EQ-5D-5L | ^19^ | CO, HC |
|  | ^54^ | CO |
|  | ^85^ | CO |
|  | ^109^ | CO |
| EQ-5D-3L | ^26^ | HC |
| No. or % of patients with pre-existing depression/anxiety (diagnosis) | ^38^ | CO |
| No. or % of patients with pre-existing depression/anxiety (self-report yes/no) | ^24^ | CO |
|  | ^51^ | CO |
| PSC internalising subscale | ^93^ | HC |

*Note.* CO = patients after COVID-19 without Long COVID; EQ-5D-3L = European Quality of Life 5 Dimensions 3 Level Version; EQ-5D-5L = Euro-pean Quality of Life 5 Dimensions 5 Level Version; HC = healthy controls without a history of COVID-19; No. = number; PSC = Pediatric Symptom Checklist; RQ = research question.

**eTable 10. Operationalisation of posttraumatic stress disorder in studies answering RQ1 and respective control groups**

| **Instrument** | **Reference(s)** | **Control group(s)** |
| --- | --- | --- |
| No. or % of patients with PTSD based on cut-off score |  |  |
| PCL-C | ^19^ | CO, HC |
|  | ^50^ | CO |
|  | ^109^ | CO |
| PC-PTSD | ^40^ | CO |
| PCL-5 | ^54^ | CO |
|  | ^76^ | HC |
| IES-R | ^7^ | CO |

*Note.* CO = patients after COVID-19 without Long COVID; HC = healthy controls without a history of COVID-19; IES-R = Impact of Event Scale – Revised; No. = number; PCL-5 = PTSD Checklist for DSM-5; PCL-C = PTSD Checklist – Civilian Version; PC-PTSD = Primary Care PTSD Screen; RQ = research question.

**eTable 11. Operationalisation of stress in studies answering RQ1 and respective control groups**

| **Instrument** | **Reference(s)** | **Control group(s)** |
| --- | --- | --- |
| No. or % of patients with stress based on cut-off score: DASS-21 | ^80^ | CO |
| No. or % of patients with pre-existing stress (self-report yes/no) | ^81^ | CO |
| DASS-21 | ^20^ | CO, HC |
| PSS | ^7^ | CO |
|  | ^68^ | HC |
| NIHTB-EB | ^60^ | HC |
| ERI - short version | ^66^ | CO |
| PAI | ^36^ | PCS |

*Note.* CO = patients after COVID-19 without Long COVID; DASS-21 = Depression, Anxiety and Stress Scale; ERI = Effort-Reward Imbalance Questionnaire; HC = healthy controls without a history of COVID-19; NIHTB-EB = National Institutes of Health Toolbox Emotion Battery; No. = number; PAI = Personality Assessment Inventory; PCS = patients with post-concussion syndrome; PSS = Perceived Stress Scale; RQ = research question.

**eTable 12. Operationalisation of history of mental health disorders in studies answering RQ1 and respective control groups**

| **Instrument** | **Reference(s)** | **Control group(s)** |
| --- | --- | --- |
| No. or % of patients with a history of mental health disorder (diagnosis) | ^45^ | CO |
| No. or % of patients with a history of mental health disorder (self-report yes/no) | ^3^ | CO |
|  | ^9^ | CO |
|  | ^112^ | CO |
|  | ^82^ | CO |

*Note.* CO = patients after COVID-19 without Long COVID; No. = number; RQ = research question.

## **eTables 13-15. Operationalisation of psychological variables with at least five observations in studies answering RQ2 and respective outcomes variables**

**eTable 13. Operationalisation of depression in studies answering RQ2 and respective outcomes variables**

| **Instrument** | **Reference(s)** | **Outcome variable(s)** |
| --- | --- | --- |
| Depression based on cut-off score |  |  |
| HADS | ^10^ | fatigue |
|  | ^18^ | LC |
|  | ^53^ | number of symptoms, cognitive deficits, pain, dizziness |
| BDI | ^97^ | cognitive deficits |
| PHQ-2 | ^98^ | LC symptoms |
| GDS | ^97^ | cognitive deficits |
| Pre-existing depression (diagnosis) | ^63^  ^71^ | LC  LC |
| Pre-existing depression (self-report yes/no) | ^98^ | LC symptoms |
| Self-reported symptoms of depression (yes/no) | ^48^ | impairment |
|  | ^87^ | QoL |
| HADS | ^11^ | QoL, fatigue |
|  | ^33^ | QoL, pain |
|  | ^39^ | number of symptoms, QoL |
|  | ^52^ | number of symptoms |
|  | ^53^ | number of symptoms, cognitive deficits, pain, dizziness |
|  | ^77^ | QoL |
|  | ^90^ | QoL |
|  | ^91^ | QoL |
| PHQ-9 | ^31^ | number of symptoms, gastrointestinal symptoms |
|  | ^59^ | LC, fatigue, respiratory symptoms, muscular symptoms, CNS symptoms |
|  | ^61^ | impairment |
|  | ^65^ | fatigue |
|  | ^88^ | LC |
| BDI-II | ^29^ | cognitive deficits |
|  | ^27^ | cognitive deficits |
|  | ^46^ | fatigue |
|  | ^105^ | cognitive deficits |
|  | ^106^ | QoL, fatigue |
| PHQ-8 | ^103^ | impairment, QoL |
| PHQ-2 | ^98^ | LC symptoms |
| GADS | ^66^ | number of symptoms |
| GDS | ^6^ | impairment, fatigue, pain |
| Conners CBRS-Parent | ^64^ | impairment |
| VAS scale | ^107^ | LC |

*Note.* BDI = Beck Depression Inventory; CNS = central nervous system; Conners CBRS-Parent = Conners Comprehensive Behavior Rating Scale-Parent; GADS = Goldberg Anxiety and Depression Scale; GDS = Geriatric Depression Scale; HADS = Hospital Anxiety and Depression Scale; LC = Long COVID; No. = number; PHQ-2 = Patient Health Questionnaire-2; PHQ-8 = Patient Health Questionnaire-8; PHQ-9 = Patient Health Questionnaire-9; QoL = quality of life; RQ = research question; VAS = Visual Analogue Scale.

**eTable 14. Operationalisation of anxiety in studies answering RQ2 and respective outcomes variables**

| **Instrument** | **Reference(s)** | **Outcome variable(s)** |
| --- | --- | --- |
| Anxiety based on cut-off score |  |  |
| HADS | ^10^ | fatigue |
|  | ^18^ | LC |
|  | ^53^ | number of symptoms, cognitive deficits, pain, dizziness |
| GAD-2 | ^98^ | LC symptoms |
| Pre-existing anxiety (diagnosis) | ^71^ | LC |
| Pre-existing anxiety (self-report yes/no) | ^98^ | LC symptoms |
| Self-reported anxiety symptoms (yes/no) | ^37^ | QoL |
|  | ^87^ | QoL |
| HADS | ^11^ | QoL, fatigue |
|  | ^33^ | QoL, pain |
|  | ^39^ | number of symptoms, QoL |
|  | ^52^ | number of symptoms |
|  | ^53^ | number of symptoms, cognitive deficits, pain, dizziness |
|  | ^77^ | QoL |
|  | ^90^ | QoL |
|  | ^91^ | QoL |
| GAD-7 | ^31^ | number of symptoms, gastrointestinal symptoms |
|  | ^61^ | impairment |
|  | ^103^ | impairment, QoL |
| STAI-S | ^6^ | impairment, fatigue, pain |
|  | ^29^ | cognitive deficits |
|  | ^27^ | cognitive deficits |
| STAI-T | ^6^ | impairment, fatigue, pain |
|  | ^29^ | cognitive deficits |
|  | ^27^ | cognitive deficits |
| BAI | ^46^ | fatigue |
|  | ^105^ | cognitive deficits |
| GAD-2 | ^98^ | LC symptoms |
| GADS | ^66^ | number of symptoms |
| Conners CBRS-Parent | ^64^ | impairment |
| VAS scale | ^17^ | pain, respiratory symptoms |

*Note.* BAI = Beck Anxiety Inventory; Conners CBRS-Parent = Conners Comprehensive Behavior Rating Scale-Parent; GAD-2 = Generalized Anxiety Disorder Scale-2; GAD-7 = Generalized Anxiety Disorder Scale-7; GADS = Goldberg Anxiety and Depression Scale; HADS = Hospital Anxiety and Depression Scale; LC = Long COVID; QoL = quality of life; RQ = research question; STAI-S = State-Trait-Anxiety Inventory (State); STAI-T = State-Trait-Anxiety Inventory (Trait); VAS = Visual Analogue Scale.

**eTable 15. Operationalisation of physical activity in studies answering RQ2 and respective outcomes variables**

| **Instrument** | **Reference(s)** | **Outcome variable(s)** |
| --- | --- | --- |
| Physical activity based on cut-off score (IPAQ) | ^111^ | LC, fatigue |
| Regular physical activity (defined as at least 150-300 min per week of moderate-intensity activity or 75-150 min per week of high-intensity activity) | ^81^ | LC symptoms |
| Categories of physical activity | ^83^ | LC |
| Practice of physical activity (yes/no) | ^87^ | QoL |
| IPAQ | ^74^ | symptom severity, impairment, overall health |
| IPAQ-SF | ^89^ | QoL |
| Accelerometer | ^17^ | pain, respiratory symptoms |

*Note.* IPAQ = International Physical Activity Questionnaire; IPAQ-SF = International Physical Activity Questionnaire Short Form; LC = Long COVID; QoL = quality of life; RQ = research question.

## **eTables 16-17. Operationalisation of psychological variables with at least five observations in studies answering RQ3 and respective outcomes variables**

**eTable 16. Operationalisation of depression in studies answering RQ3 and respective outcomes variables**

| **Instrument** | **Reference(s)** | **Outcome variable(s)** |
| --- | --- | --- |
| Depression based on cut-off score |  |  |
| PHQ-9 | ^108^ | fatigue, cognitive deficits |
| PHQ-2 | ^104^ | LC, impairment |
| Pre-existing depression (diagnosis) | ^4^ | LC |
|  | ^12^ | LC |
|  | ^47^ | LC |
|  | ^56^ | LC |
|  | ^62^ | LC |
|  | ^96^ | LC |
| HADS | ^14^ | fatigue, pain, respiratory symptoms |
| PHQ-8 | ^30^ | LC |
| STADI | ^113^ | LC, impairment |
| BDI-13 | ^112^ | LC |
| ZSDS index | ^112^ | LC |

*Note.* BDI-13 = Beck Depression Inventory-13; HADS = Hospital Anxiety and Depression Scale; LC = Long COVID; PHQ-2 = Patient Health Ques-tionnaire-2; PHQ-8 = Patient Health Questionnaire-8; PHQ-9 = Patient Health Questionnaire-9; RQ = research question; STADI = State Trait Anxiety and Depression Inventory; ZSDS = Zung Self-Rating Depression Scale.

**eTable 17. Operationalisation of anxiety in studies answering RQ3 and respective outcomes variables**

| **Instrument** | **Reference(s)** | **Outcome variable(s)** |
| --- | --- | --- |
| Anxiety based on cut-off score |  |  |
| GAD-7 | ^108^ | fatigue, cognitive deficits |
| GAD-2 | ^104^ | LC, impairment |
|  | ^56^ | LC |
|  | ^57^ | LC |
|  | ^62^ | LC |
|  | ^96^ | LC |
| HADS | ^14^ | fatigue, pain, respiratory symptoms |
| GAD-7 | ^30^ | LC |

*Note.* GAD-2 = Generalized Anxiety Disorder Scale-2; GAD-7 = Generalized Anxiety Disorder Scale-7; HADS = Hospital Anxiety and Depression Scale; LC = Long COVID; RQ = research question.

## **eTable 18. Sensitivity analyses for meta-analyses of cross-sectional studies with control groups**

| **Categorical data** | **Analysis** | ***z*** | ***p*** | **OR (95% CI)** | **Heterogeneity (*I^2^*)** |
| --- | --- | --- | --- | --- | --- |
| Depression | Main Analysis | 3.69 | < .001 | 2.35 (1.49-3.70) | 87% |
|  | Studies with greatest weight excl. | 3.32 | < .001 | 2.44 (1.44-4.14) | 88% |
|  | Studies with smallest sample size excl. | 3.40 | < .001 | 2.30 (1.42-3.70) | 88% |
|  | Studies with poor quality rating excl. | 3.57 | < .001 | 2.42 (1.49-3.92) | 88% |
| Anxiety | Main Analysis | 5.07 | < .001 | 2.53 (1.76-3.61) | 88% |
|  | Studies with greatest weight excl. | 4.89 | < .001 | 2.62 (1.78-3.85) | 89% |
|  | Studies with smallest sample size excl. | 4.96 | < .001 | 2.59 (1.78-3.78) | 89% |
|  | Studies with poor quality rating excl. | 5.76 | < .001 | 2.75 (1.95-3.87) | 88% |
| **Continuous data** | **Analysis** | ***z*** | ***p*** | **SMD (95% CI)** | **Heterogeneity (*I^2^*)** |
| Depression | Main Analysis | 7.73 | < .001 | 0.88 (0.66-1.11) | 93% |
|  | Studies with greatest weight excl. | 7.96 | < .001 | 0.97 (0.73-1.21) | 90% |
|  | Studies with smallest sample size excl. | 7.22 | < .001 | 0.91 (0.66-1.16) | 94% |
|  | Studies with poor quality rating excl. | 7.33 | < .001 | 0.91 (0.66-1.15) | 93% |
| Anxiety | Main Analysis | 5.98 | < .001 | 0.74 (0.50-0.99) | 92% |
|  | Studies with greatest weight excl. | 6.41 | < .001 | 0.83 (0.58-1.08) | 85% |
|  | Studies with smallest sample size excl. | 5.50 | < .001 | 0.76 (0.49-1.03) | 93% |
|  | Studies with poor quality rating excl. | 5.35 | < .001 | 0.76 (0.48-1.04) | 93% |
| Physical activity | Main Analysis | -1.79 | .07 | -0.75 (-1.58-0.07) | 97% |
|  | Studies with greatest weight excl. | -1.72 | .08 | -0.86 (-1.84-0.12) | 97% |
|  | Studies with smallest sample size excl. | -1.43 | .15 | -0.72 (-1.71-0.27) | 98% |
|  | Studies with poor quality rating excl. | – | – | – | – |
| Stress | Main Analysis | 1.66 | .10 | 0.40 (-0.07-0.87) | 84% |
|  | Studies with greatest weight excl. | 1.20 | .23 | 0.37 (-0.23-0.98) | 71% |
|  | Studies with smallest sample size excl. | 1.54 | .12 | 0.45 (-0.12-1.03) | 88% |
|  | Studies with poor quality rating excl. | 1.54 | .12 | 0.45 (-0.12-1.03) | 88% |

*Note.* OR = odds ratio, SMD = standardised mean difference; studies with smallest sample size: n < 20 per group.

## **eFigure 1. Forest plot depicting meta-analysis of cross-sectional studies with control groups investigating depression continuously**


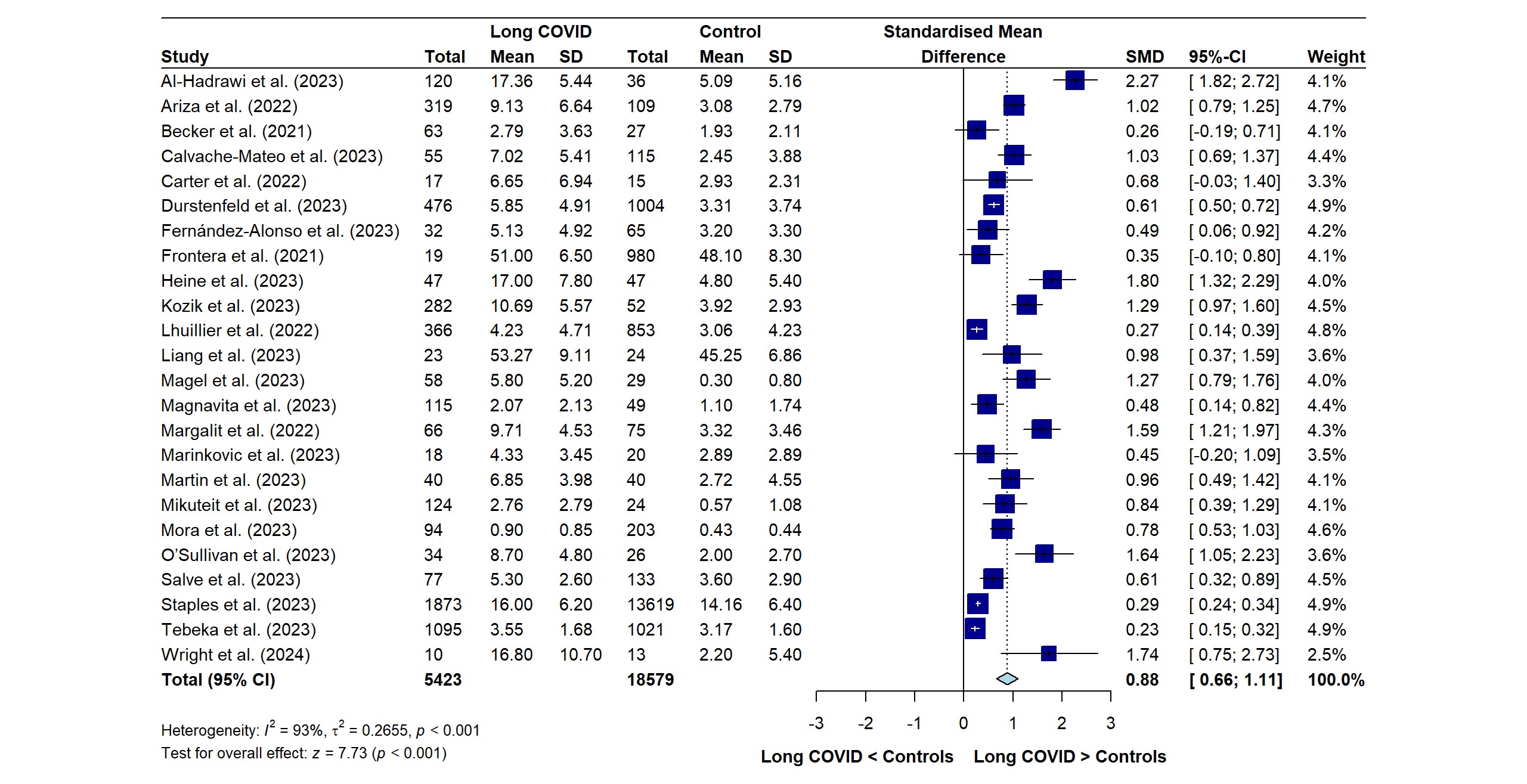


## **eFigure 2. Forest plot depicting meta-analysis of cross-sectional studies with control groups investigating depression categorically**


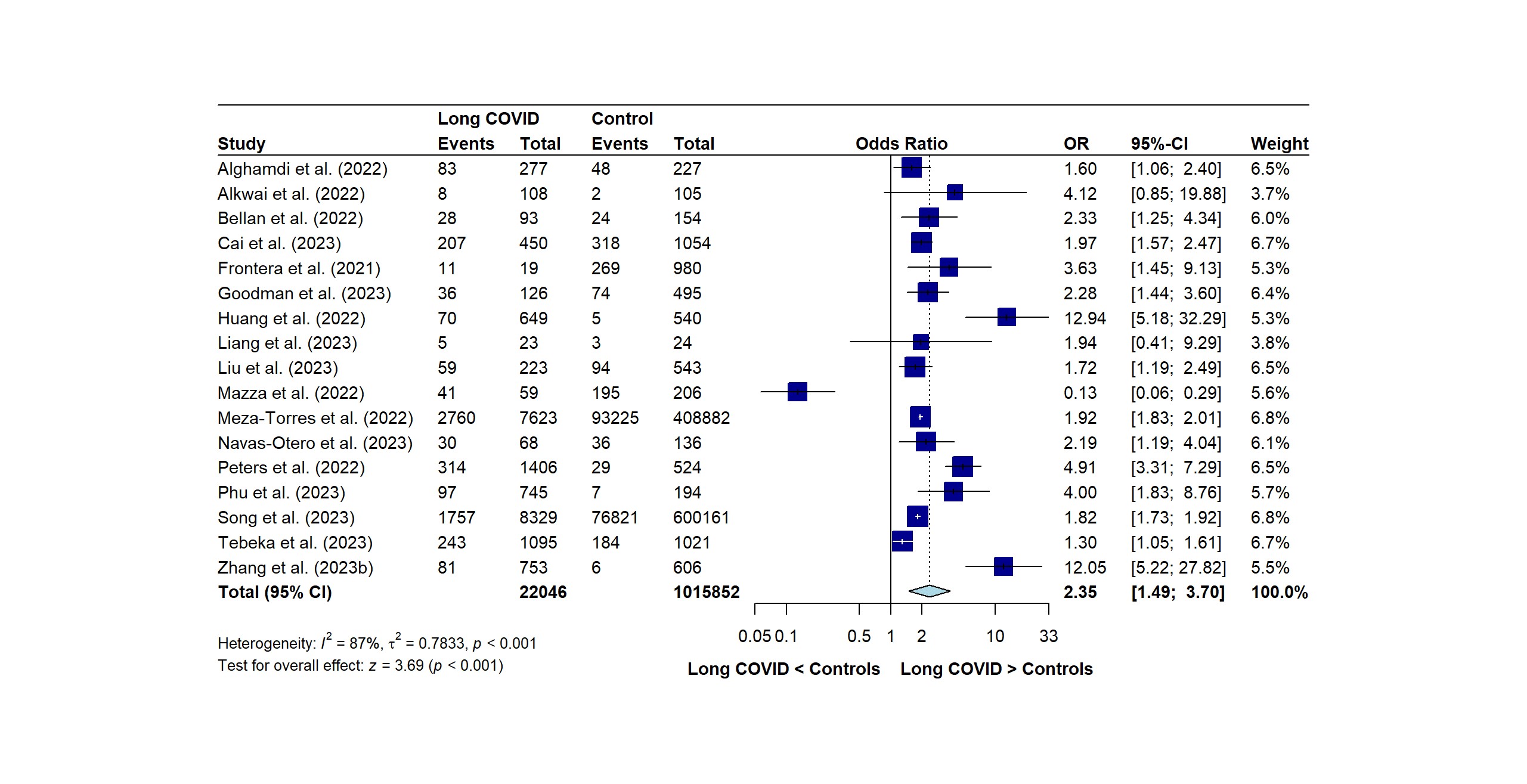


## **eFigure 3. Forest plot depicting meta-analysis of cross-sectional studies with control groups investigating anxiety continuously**


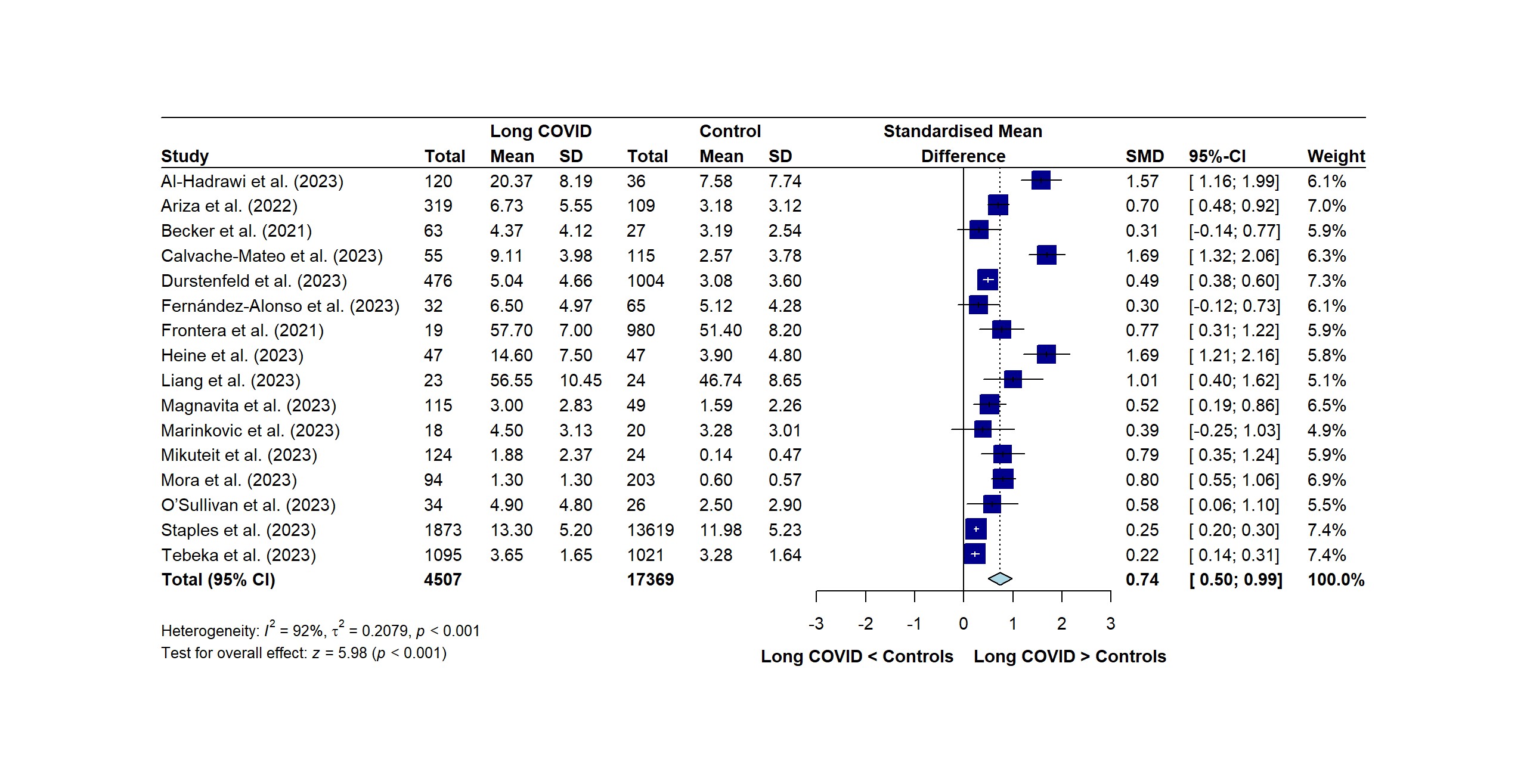


## **eFigure 4. Forest plot depicting meta-analysis of cross-sectional studies with control groups investigating anxiety categorically**


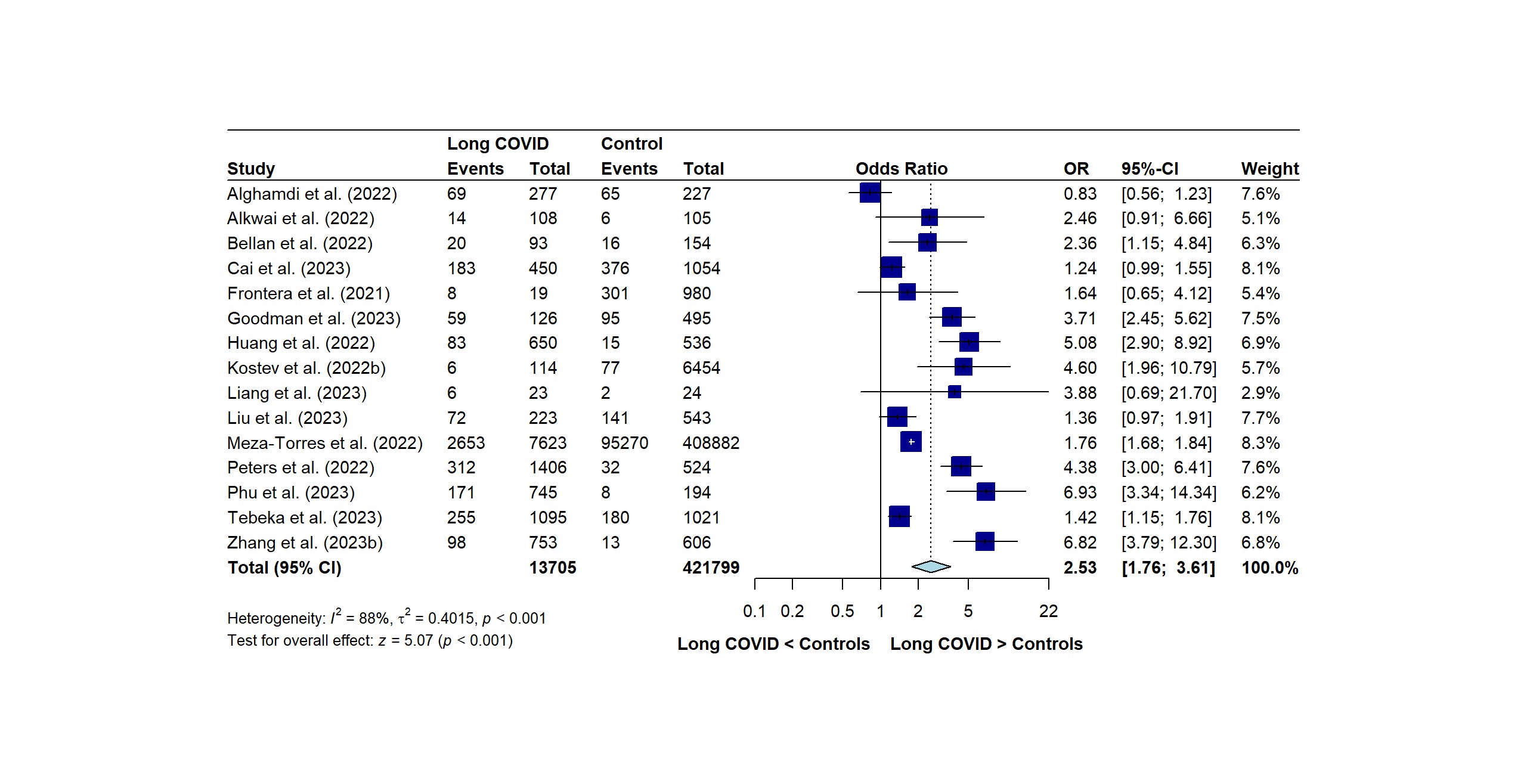


## **eFigure 5. Forest plot depicting meta-analysis of cross-sectional studies with control groups investigating physical activity**


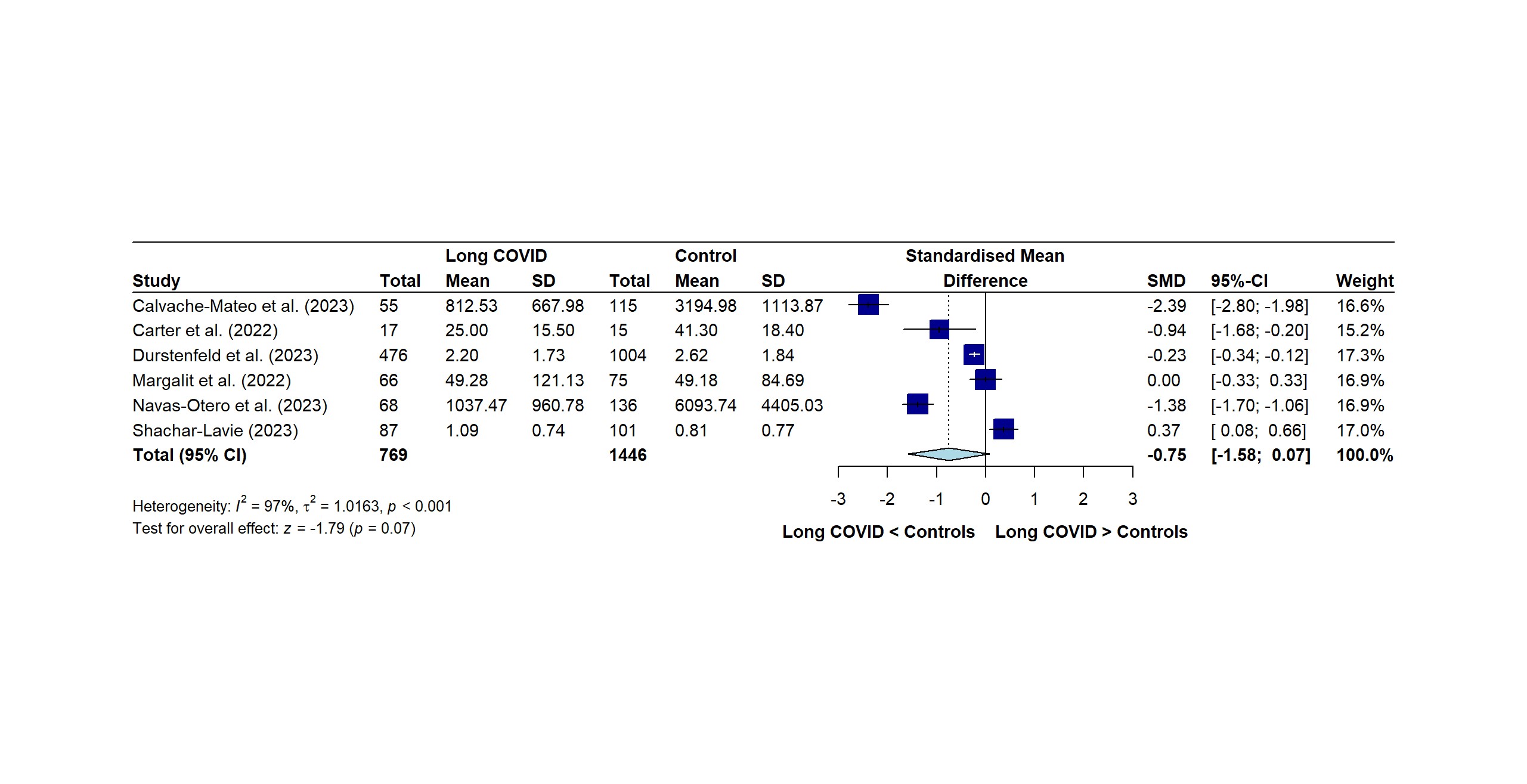


## **eFigure 6. Forest plot depicting meta-analysis of cross-sectional studies with control groups investigating stress**


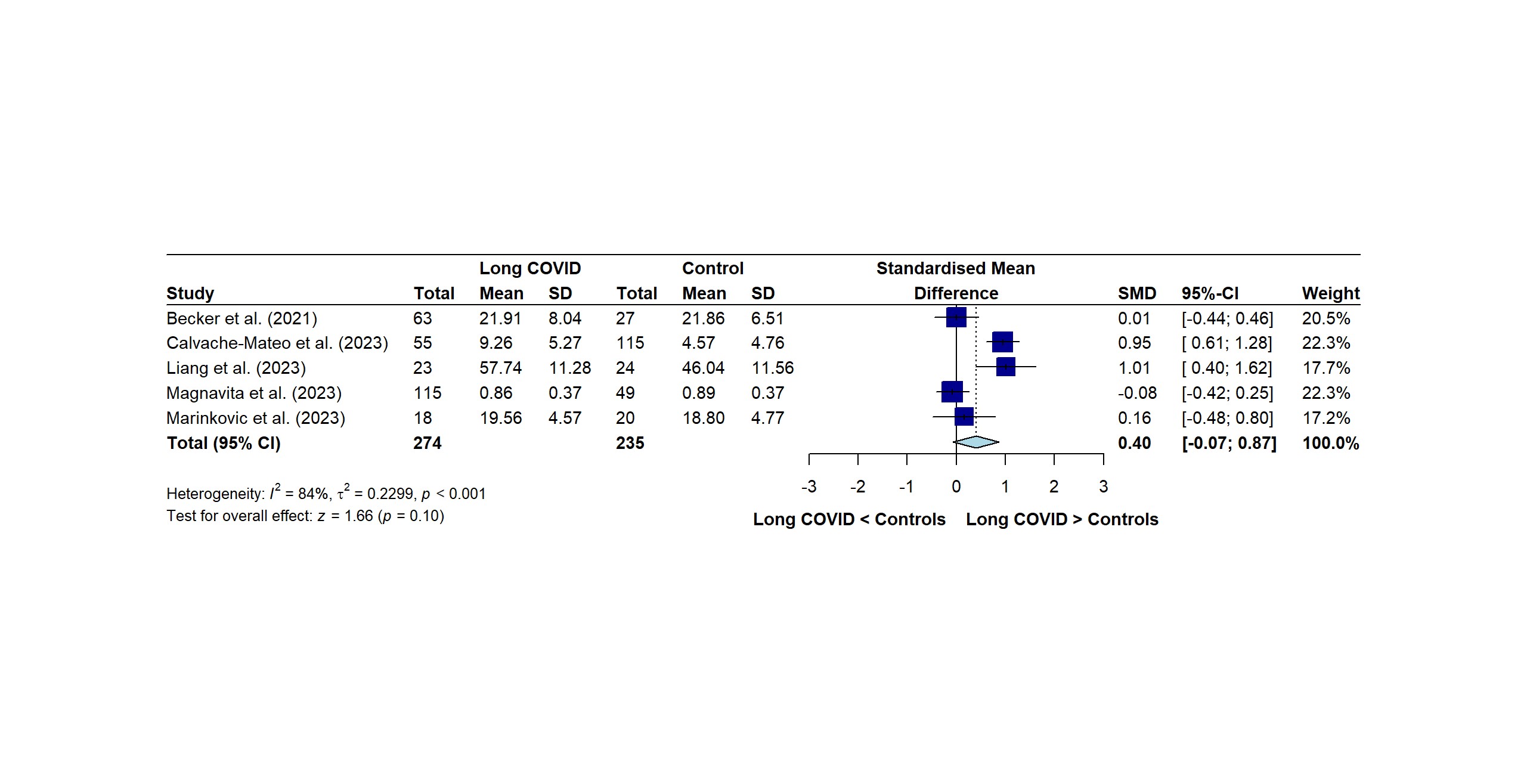


## **eFigures 7-12. Funnel plots**

**eFigure 7. Funnel plot for meta-analysis of cross-sectional studies with control groups investigating depression continuously**


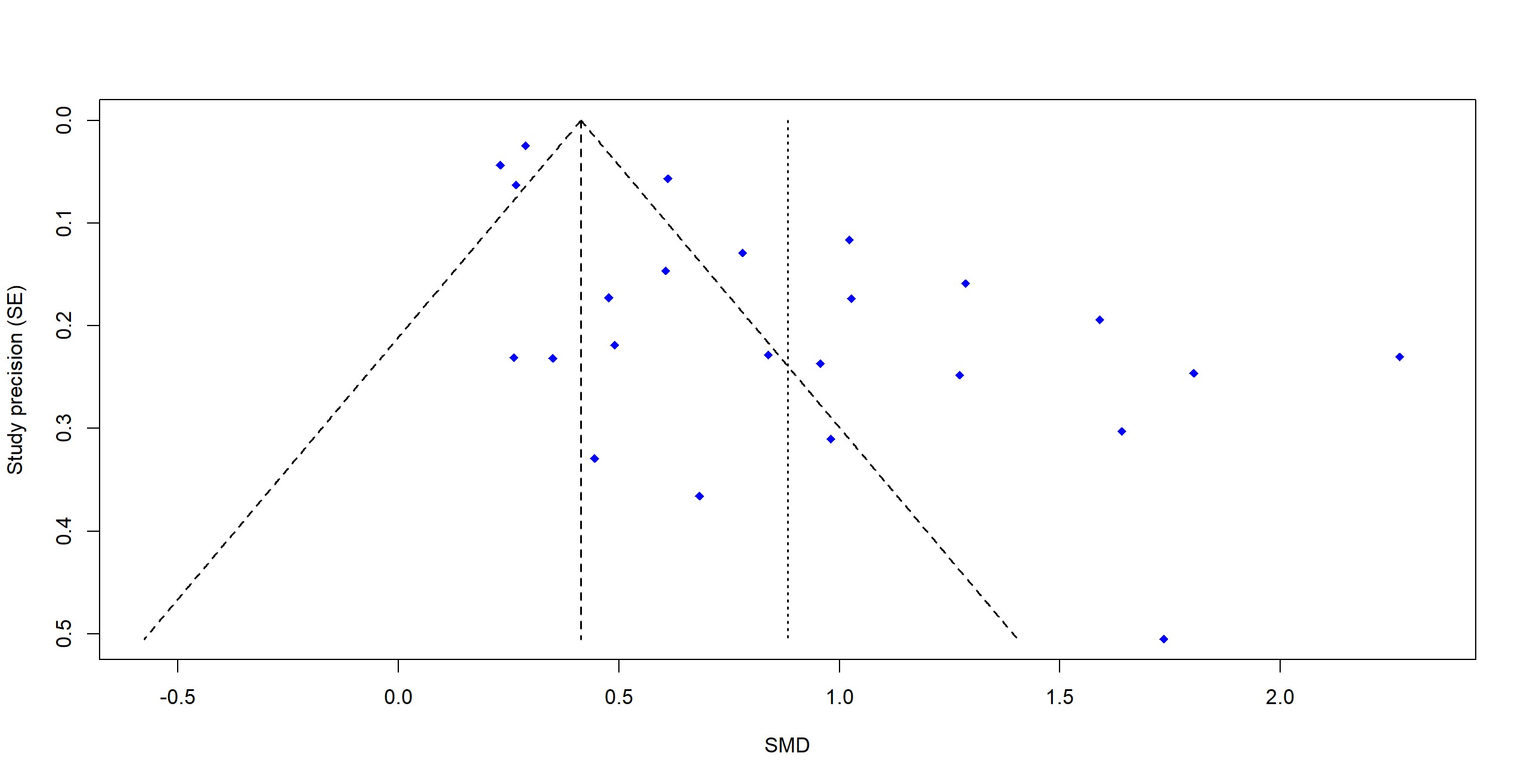


**eFigure 8. Funnel plot for meta-analysis of cross-sectional studies with control groups investigating depression categorically**


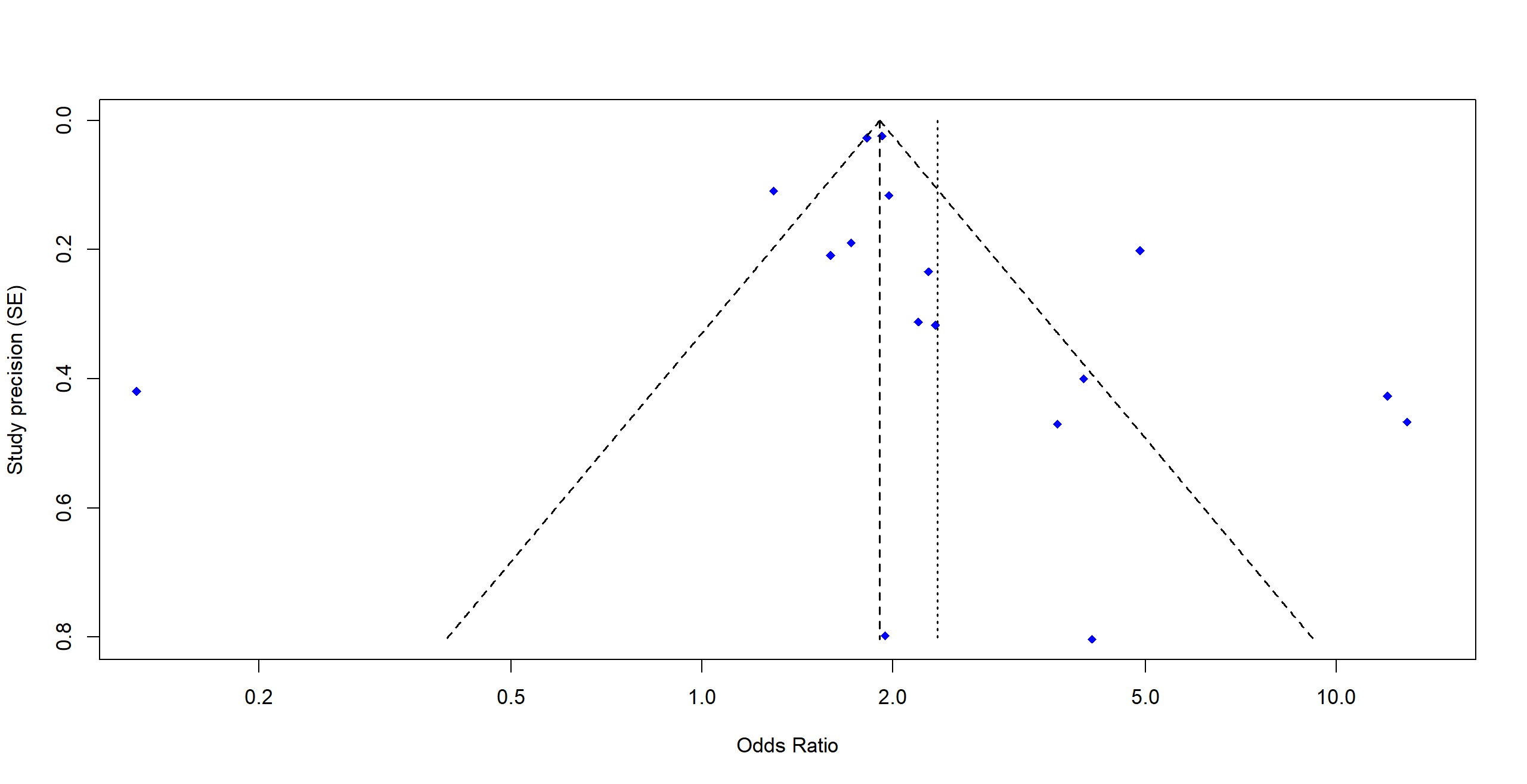


**eFigure 9. Funnel plot for meta-analysis of cross-sectional studies with control groups investigating anxiety continuously**


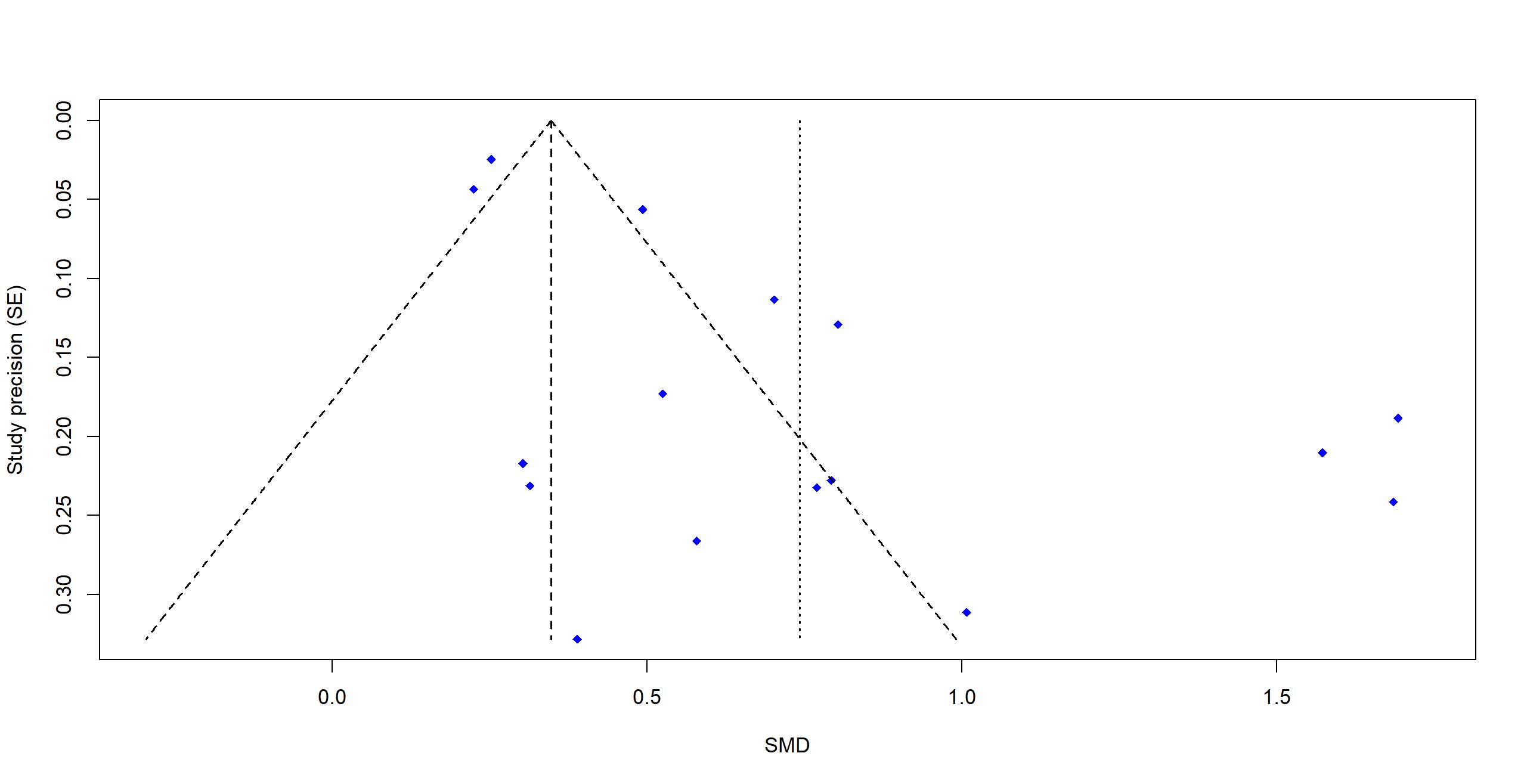


**eFigure 10. Funnel plot for meta-analysis of cross-sectional studies with control groups investigating anxiety categorically**


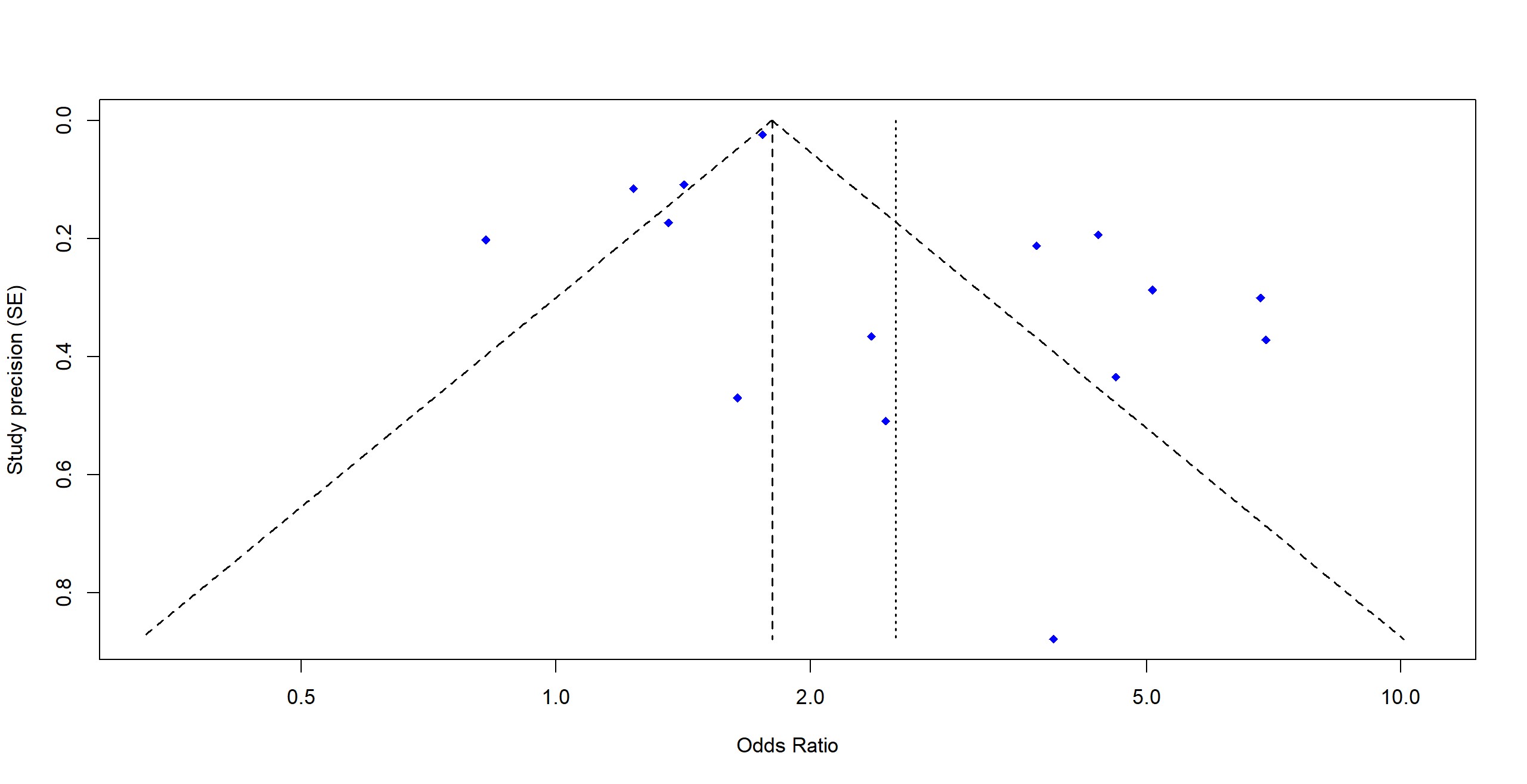


**eFigure 11. Funnel plot for meta-analysis of cross-sectional studies with control groups investigating physical activity**


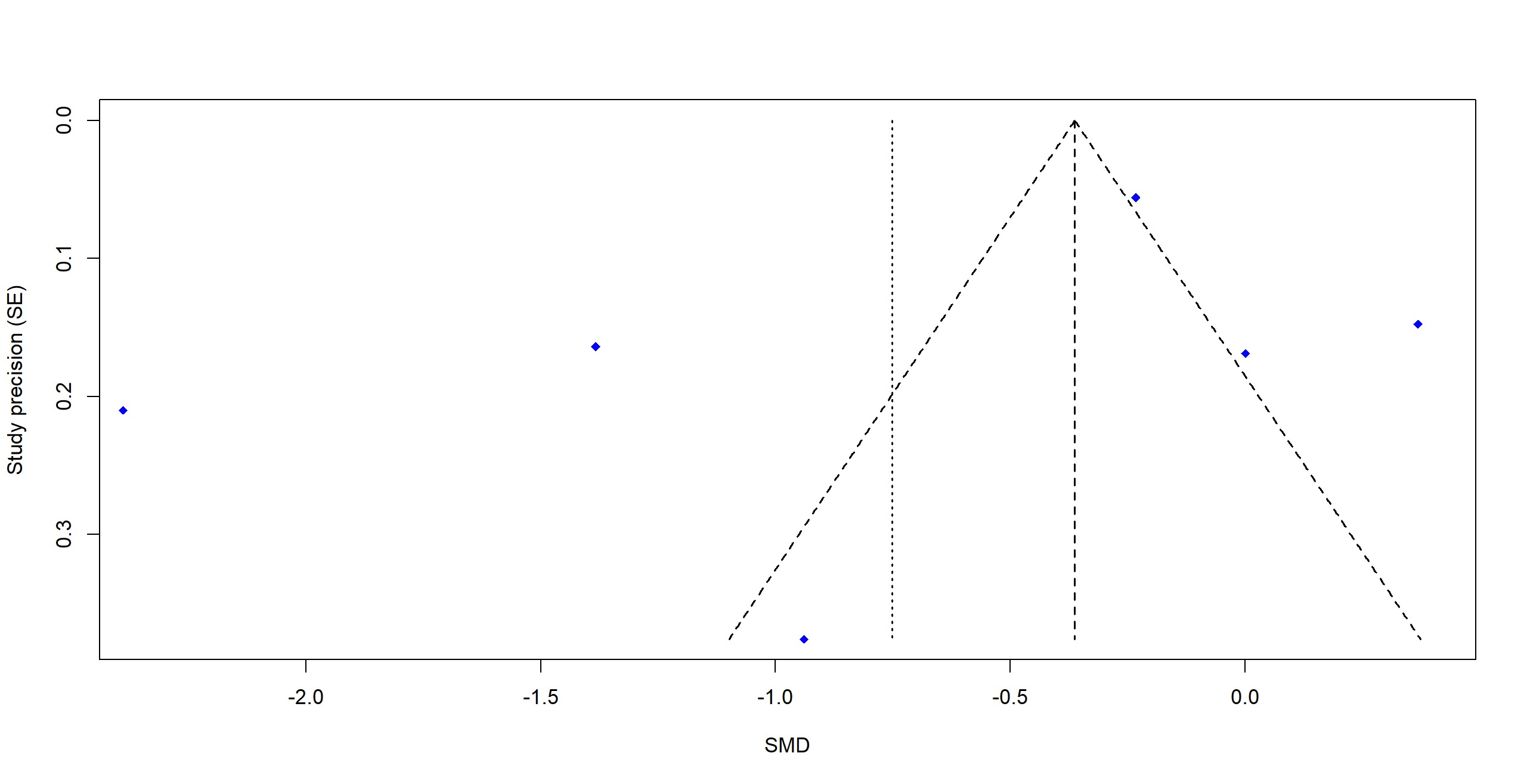


**eFigure 12. Funnel plot for meta-analysis of cross-sectional studies with control groups investigating stress**


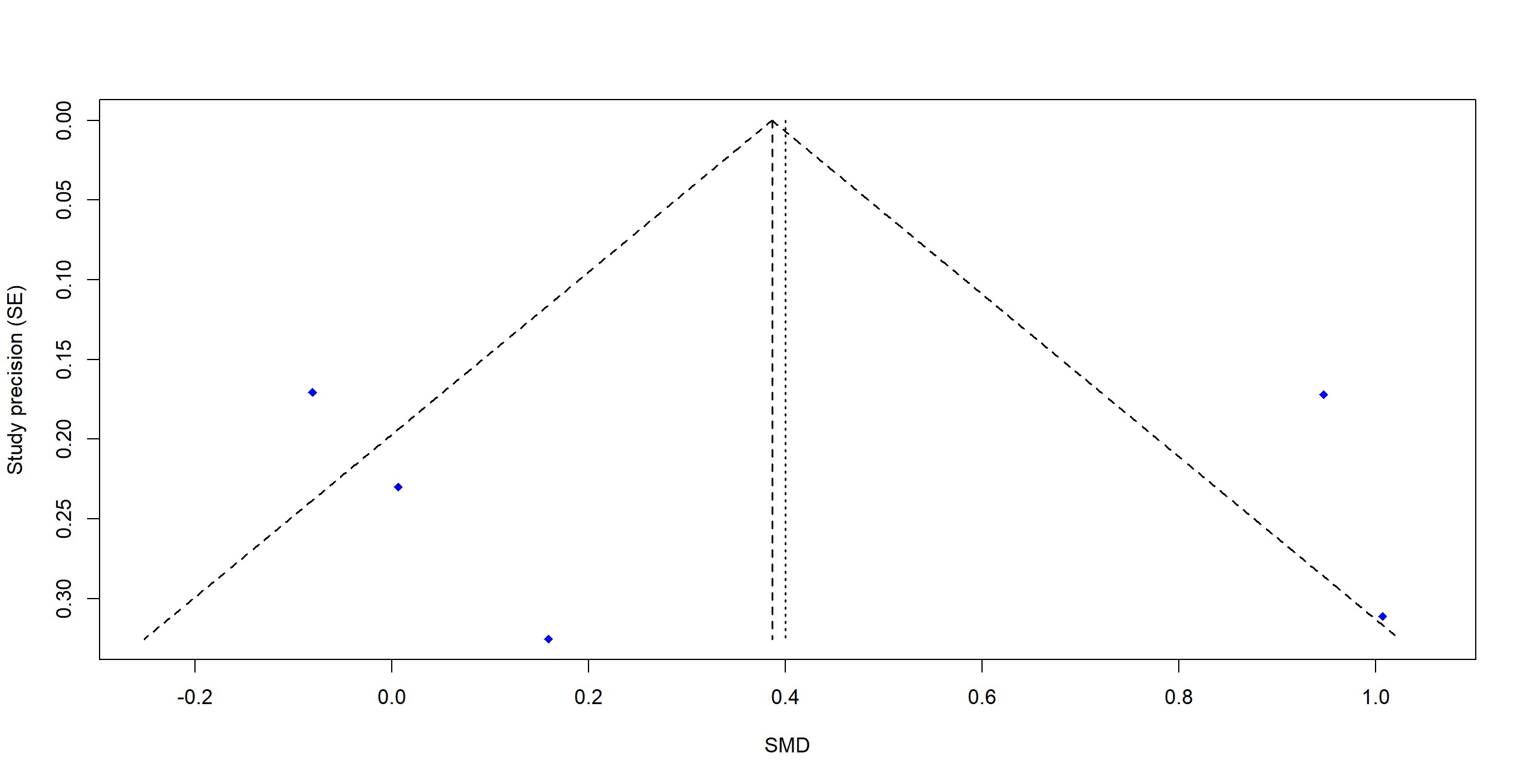


## **eReferences**

1. Al-Hadrawi DS, Al-Rubaye HT, Almulla AF, Al-Hakeim HK, Maes M. Lowered oxygen saturation and increased body temperature in acute COVID-19 largely predict chronic fatigue syndrome and affective symptoms due to Long COVID: A precision nomothetic approach. *Acta Neuropsychiatr*. 2023-4 2023;35(2):76-87. doi:doi:10.1017/neu.2022.21

2. Alghamdi SA, Alfares MA, Alsulami RA, et al. Post-COVID-19 Syndrome: Incidence, Risk Factor, and the Most Common Persisting Symptoms. *Cureus*. 2022-11 2022;14(11):e32058. doi:doi:10.7759/cureus.32058

3. Alkwai HM, Khalifa AM, Ahmed AM, et al. Persistence of COVID-19 symptoms beyond 3 months and the delayed return to the usual state of health in Saudi Arabia: A cross-sectional study. *SAGE Open Med*. 2022 2022;10:20503121221129918. doi:doi:10.1177/20503121221129918

4. Antony B, Blau H, Casiraghi E, et al. Predictive models of long COVID. *EBioMedicine*. 2023-10 2023;96:104777. doi:doi:10.1016/j.ebiom.2023.104777

5. Ariza M, Cano N, Segura B, et al. Neuropsychological impairment in post-COVID condition individuals with and without cognitive complaints. *Frontiers in Aging Neuroscience Vol 14 2022, ArtID 1029842*. 2022-10 2022;14doi:doi:<https://dx.doi.org/10.3389/fnagi.2022.1029842>

6. Azcue N, Gómez-Esteban JC, Acera M, et al. Brain fog of post-COVID-19 condition and Chronic Fatigue Syndrome, same medical disorder? *J Transl Med*. 2022-12-6 2022;20(1):569. doi:doi:10.1186/s12967-022-03764-2

7. Becker C, Beck K, Zumbrunn S, et al. Long COVID 1 year after hospitalisation for COVID-19: a prospective bicentric cohort study. *Swiss Med Wkly*. 2021-10-11 2021;151:w30091. doi:doi:10.4414/smw.2021.w30091

8. Bellan M, Apostolo D, Albè A, et al. Determinants of long COVID among adults hospitalized for SARS-CoV-2 infection: A prospective cohort study. *Front Immunol*. 2022 2022;13:1038227. doi:doi:10.3389/fimmu.2022.1038227

9. Benoit-Piau J, Tremblay K, Piché A, et al. Long-Term Consequences of COVID-19 in Predominantly Immunonaive Patients: A Canadian Prospective Population-Based Study. *J Clin Med*. 2023-9-13 2023;12(18)doi:doi:10.3390/jcm12185939

10. Beyer S, Haufe S, Meike D, et al. Post-COVID-19 syndrome: Physical capacity, fatigue and quality of life. *PLoS One*. 2023 2023;18(10):e0292928. doi:doi:10.1371/journal.pone.0292928

11. Bierbauer W, Luscher J, Scholz U. Illness perceptions in long-COVID: A cross-sectional analysis in adults. *Cogent Psychology Vol 9(1), 2022, ArtID 2105007*. 2022-1 2022;9(1)doi:doi:<https://dx.doi.org/10.1080/23311908.2022.2105007>

12. Binka M, Klaver B, Cua G, et al. An Elastic Net Regression Model for Identifying Long COVID Patients Using Health Administrative Data: A Population-Based Study. *Open Forum Infect Dis*. 2022-12 2022;9(12):ofac640. doi:doi:10.1093/ofid/ofac640

13. Bonner C, Ghouralal SL. Long COVID and Chronic Conditions in the U.S. Workforce: Prevalence, Productivity Loss, and Disability. *J Occup Environ Med*. 2024-1-2 2024;doi:doi:10.1097/jom.0000000000003026

14. Bottemanne H, Gouraud C, Hulot JS, et al. Do Anxiety and Depression Predict Persistent Physical Symptoms After a Severe COVID-19 Episode? A Prospective Study. *Front Psychiatry*. 2021 2021;12:757685. doi:doi:10.3389/fpsyt.2021.757685

15. Bungenberg J, Humkamp K, Hohenfeld C, et al. Long COVID-19: Objectifying most self-reported neurological symptoms. *Ann Clin Transl Neurol*. 2022-2 2022;9(2):141-154. doi:doi:10.1002/acn3.51496

16. Buonsenso D, Camporesi A, Morello R, et al. Social Stigma in Children with Long COVID. *Children (Basel)*. 2023-9-7 2023;10(9)doi:doi:10.3390/children10091518

17. Burton C, Dawes H, Goodwill S, Thelwell M, Dalton C. Within and between-day variation and associations of symptoms in Long Covid: Intensive longitudinal study. *PLoS One*. 2023 2023;18(1):e0280343. doi:doi:10.1371/journal.pone.0280343

18. Busatto GF, de Araujo AL, Castaldelli-Maia JM, et al. Post-acute sequelae of SARS-CoV-2 infection: Relationship of central nervous system manifestations with physical disability and systemic inflammation. *Psychological Medicine*. 2022-9 2022;52(12):2387-2398. doi:doi:<https://dx.doi.org/10.1017/S0033291722001374>

19. Cai J, Lin K, Zhang H, et al. A one-year follow-up study of systematic impact of long COVID symptoms among patients post SARS-CoV-2 omicron variants infection in Shanghai, China. *Emerg Microbes Infect*. 2023-12 2023;12(2):2220578. doi:doi:10.1080/22221751.2023.2220578

20. Calvache-Mateo A, Navas-Otero A, Heredia-Ciuró A, et al. Post-COVID Patients With New-Onset Chronic Pain 2 Years After Infection: Cross-Sectional Study. *Pain Manag Nurs*. 2023-5-22 2023;doi:doi:10.1016/j.pmn.2023.04.010

21. Carazo S, Skowronski DM, Laforce R, Jr., et al. Physical, Psychological, and Cognitive Profile of Post-COVID Conditions in Healthcare Workers, Quebec, Canada. *Open Forum Infect Dis*. 2022-8 2022;9(8):ofac386. doi:doi:10.1093/ofid/ofac386

22. Carter SJ, Baranauskas MN, Raglin JS, Pescosolido BA, Perry BL. Functional status, mood state, and physical activity among women with post-acute COVID-19 syndrome. *International Journal of Public Health Vol 67 2022, ArtID 1604589*. 2022-6 2022;67doi:doi:<https://dx.doi.org/10.3389/ijph.2022.1604589>

23. Chen EY, Morrow AK, Malone LA. Exploring the Influence of Pre-Existing Conditions and Infection Factors on Pediatric Long COVID Symptoms and Quality of Life. *Am J Phys Med Rehabil*. 2023-10-30 2023;doi:doi:10.1097/phm.0000000000002363

24. Chen SJ, Morin CM, Ivers H, et al. The association of insomnia with long COVID: An international collaborative study (ICOSS-II). *Sleep Med*. 2023-12 2023;112:216-222. doi:doi:10.1016/j.sleep.2023.09.034

25. Cuschieri S, Grech S, Grech V. A glimpse into Long COVID characteristics and the mental health impact within a highly vaccinated population: a Malta observational study. *Ann Ist Super Sanita*. 2023-4 2023;59(2):101-107. doi:doi:10.4415/ann_23_02_02

26. de Oliveira JF, de Ávila RE, de Oliveira NR, et al. Persistent symptoms, quality of life, and risk factors in long COVID: a cross-sectional study of hospitalized patients in Brazil. *Int J Infect Dis*. 2022-9 2022;122:1044-1051. doi:doi:10.1016/j.ijid.2022.07.063

27. Delgado-Alonso C, Díez-Cirarda M, Pagán J, et al. Unraveling brain fog in post-COVID syndrome: Relationship between subjective cognitive complaints and cognitive function, fatigue, and neuropsychiatric symptoms. *Eur J Neurol*. 2023-10-5 2023;doi:doi:10.1111/ene.16084

28. Delgado-Alonso C, Valles-Salgado M, Delgado-Álvarez A, et al. Examining Association of Personality Characteristics and Neuropsychiatric Symptoms in Post-COVID Syndrome. *Brain Sci*. 2022-2-14 2022;12(2)doi:doi:10.3390/brainsci12020265

29. Delgado-Alonso C, Valles-Salgado M, Delgado-Álvarez A, et al. Cognitive dysfunction associated with COVID-19: A comprehensive neuropsychological study. *J Psychiatr Res*. 2022-6 2022;150:40-46. doi:doi:10.1016/j.jpsychires.2022.03.033

30. Durstenfeld MS, Peluso MJ, Peyser ND, et al. Factors Associated With Long COVID Symptoms in an Online Cohort Study. *Open Forum Infect Dis*. 2023-2 2023;10(2):ofad047. doi:doi:10.1093/ofid/ofad047

31. Elmunzer BJ, Palsson OS, Forbes N, et al. Prolonged Gastrointestinal Manifestations After Recovery From COVID-19. *Clin Gastroenterol Hepatol*. 2023-11-22 2023;doi:doi:10.1016/j.cgh.2023.11.009

32. Fernández-Alonso V, Rodríguez-Fernández S, Secadas-Rincón L, Pérez-Gómez M, Moro-Tejedor MN, Salcedo M. Resilience After COVID-19: A Descriptive, Cross-Sectional Study. *Clin Nurs Res*. 2023-3 2023;32(3):618-628. doi:doi:10.1177/10547738231154326

33. Fernández-de-Las-Peñas C, Valera-Calero JA, Herrero-Montes M, et al. The Self-Reported Leeds Assessment of Neuropathic Symptoms and Signs (S-LANSS) and PainDETECT Questionnaires in COVID-19 Survivors with Post-COVID Pain. *Viruses*. 2022-7-7 2022;14(7)doi:doi:10.3390/v14071486

34. Freda MF, Scandurra C, Auriemma E, et al. Long-COVID in children: An exploratory case-control study from a bio-psycho-social perspective. *J Psychosom Res*. 2024-1 2024;176:111564. doi:doi:10.1016/j.jpsychores.2023.111564

35. Frontera JA, Lewis A, Melmed K, et al. Prevalence and Predictors of Prolonged Cognitive and Psychological Symptoms Following COVID-19 in the United States. *Front Aging Neurosci*. 2021 2021;13:690383. doi:doi:10.3389/fnagi.2021.690383

36. Fry L, Logemann A, Waldron E, et al. Emotional functioning in long COVID: Comparison to post-concussion syndrome using the Personality Assessment Inventory. *Clin Neuropsychol*. 2023-10-15 2023:1-21. doi:doi:10.1080/13854046.2023.2264546

37. Fujita K, Otsuka Y, Sunada N, et al. Manifestation of Headache Affecting Quality of Life in Long COVID Patients. *J Clin Med*. 2023-5-18 2023;12(10)doi:doi:10.3390/jcm12103533

38. Gaspar P, Dias M, Parreira I, et al. Predictors of Long-COVID-19 and its Impact on Quality of Life: Longitudinal Analysis at 3, 6 and 9 Months after Discharge from a Portuguese Centre. *Acta Med Port*. 2023-2-24 2023;doi:doi:10.20344/amp.19047

39. Giurgi-Oncu C, Tudoran C, Pop GN, et al. Cardiovascular Abnormalities and Mental Health Difficulties Result in a Reduced Quality of Life in the Post-Acute COVID-19 Syndrome. *Brain Sci*. 2021-11-2 2021;11(11)doi:doi:10.3390/brainsci11111456

40. Goodman ML, Molldrem S, Elliott A, Robertson D, Keiser P. Long COVID and mental health correlates: A new chronic condition fits existing patterns. *Health Psychology and Behavioral Medicine Vol 11(1), 2023, ArtID 2164498*. 2023-12 2023;11(1)doi:doi:<https://dx.doi.org/10.1080/21642850.2022.2164498>

41. Gouraud C, Bottemanne H, Lahlou-Laforêt K, et al. Association Between Psychological Distress, Cognitive Complaints, and Neuropsychological Status After a Severe COVID-19 Episode: A Cross-Sectional Study. *Front Psychiatry*. 2021 2021;12:725861. doi:doi:10.3389/fpsyt.2021.725861

42. Haider S, Janowski AJ, Lesnak JB, et al. A comparison of pain, fatigue, and function between post-COVID-19 condition, fibromyalgia, and chronic fatigue syndrome: A survey study. *Pain*. 2023-2 2023;164(2):385-401. doi:doi:<https://dx.doi.org/10.1097/j.pain.0000000000002711>

43. Harenwall S, Heywood-Everett S, Henderson R, Smith J, McEnery R, Bland AR. The Interactive Effects of Post-Traumatic Stress Symptoms and Breathlessness on Fatigue Severity in Post-COVID-19 Syndrome. *J Clin Med*. 2022-10-21 2022;11(20)doi:doi:10.3390/jcm11206214

44. Hastie CE, Lowe DJ, McAuley A, et al. Outcomes among confirmed cases and a matched comparison group in the Long-COVID in Scotland study. *Nat Commun*. 2022-10-12 2022;13(1):5663. doi:doi:10.1038/s41467-022-33415-5

45. Hedberg P, Granath F, Bruchfeld J, et al. Post COVID-19 condition diagnosis: A population-based cohort study of occurrence, associated factors, and healthcare use by severity of acute infection. *J Intern Med*. 2023-2 2023;293(2):246-258. doi:doi:10.1111/joim.13584

46. Heine J, Schwichtenberg K, Hartung TJ, et al. Structural brain changes in patients with post-COVID fatigue: a prospective observational study. *EClinicalMedicine*. 2023-4 2023;58:101874. doi:doi:10.1016/j.eclinm.2023.101874

47. Hill EL, Mehta HB, Sharma S, et al. Risk factors associated with post-acute sequelae of SARS-CoV-2: an N3C and NIH RECOVER study. *BMC Public Health*. 2023-10-25 2023;23(1):2103. doi:doi:10.1186/s12889-023-16916-w

48. Hirahata K, Nawa N, Fujiwara T. Characteristics of Long COVID: Cases from the First to the Fifth Wave in Greater Tokyo, Japan. *J Clin Med*. 2022-10-31 2022;11(21)doi:doi:10.3390/jcm11216457

49. Hirschtick JL, Xie Y, Slocum E, et al. A statewide population-based approach to examining Long COVID symptom prevalence and predictors in Michigan. *Prev Med*. 2023-12 2023;177:107752. doi:doi:10.1016/j.ypmed.2023.107752

50. Huang L, Li X, Gu X, et al. Health outcomes in people 2 years after surviving hospitalisation with COVID-19: a longitudinal cohort study. *Lancet Respir Med*. 2022-9 2022;10(9):863-876. doi:doi:10.1016/s2213-2600(22)00126-6

51. Jacobs ET, Catalfamo CJ, Colombo PM, et al. Pre-existing conditions associated with post-acute sequelae of COVID-19. *J Autoimmun*. 2023-2 2023;135:102991. doi:doi:10.1016/j.jaut.2022.102991

52. Jung YH, Ha EH, Choe KW, Lee S, Jo DH, Lee WJ. Persistent Symptoms After Acute COVID-19 Infection in Omicron Era. *J Korean Med Sci*. 2022-7-11 2022;37(27):e213. doi:doi:10.3346/jkms.2022.37.e213

53. Jung YH, Ha EH, Park J, Choe KW, Lee WJ, Jo DH. Neurological and Psychiatric Manifestations of Post-COVID-19 Conditions. *J Korean Med Sci*. 2023-3-20 2023;38(11):e83. doi:doi:10.3346/jkms.2023.38.e83

54. Kim Y, Kim SW, Chang HH, Kwon KT, Hwang S, Bae S. One Year Follow-Up of COVID-19 Related Symptoms and Patient Quality of Life: A Prospective Cohort Study. *Yonsei Med J*. 2022-6 2022;63(6):499-510. doi:doi:10.3349/ymj.2022.63.6.499

55. König BH, van Jaarsveld CH, Bischoff EW, Schers HJ, Lucassen PL, Olde Hartman TC. Prognostic factors for persistent fatigue after COVID-19: a prospective matched cohort study in primary care. *Br J Gen Pract*. 2023-5 2023;73(730):e340-e347. doi:doi:10.3399/bjgp.2022.0158

56. Kostev K, Smith L, Koyanagi A, Jacob L. Prevalence of and Factors Associated With Post-Coronavirus Disease 2019 (COVID-19) Condition in the 12 Months After the Diagnosis of COVID-19 in Adults Followed in General Practices in Germany. *Open Forum Infect Dis*. 2022-7 2022;9(7):ofac333. doi:doi:10.1093/ofid/ofac333

57. Kostev K, Smith L, Koyanagi A, Konrad M, Jacob L. Post-COVID-19 conditions in children and adolescents diagnosed with COVID-19. *Pediatr Res*. 2022-5-14 2022:1-6. doi:doi:10.1038/s41390-022-02111-x

58. Kozik V, Reuken P, Utech I, et al. Characterization of neurocognitive deficits in patients with post-COVID-19 syndrome: persistence, patients' complaints, and clinical predictors. *Front Psychol*. 2023 2023;14:1233144. doi:doi:10.3389/fpsyg.2023.1233144

59. Lhuillier E, Yang Y, Morozova O, et al. The Impact of World Trade Center Related Medical Conditions on the Severity of COVID-19 Disease and Its Long-Term Sequelae. *Int J Environ Res Public Health*. 2022-6-7 2022;19(12)doi:doi:10.3390/ijerph19126963

60. Liang H, Ernst T, Oishi K, et al. Abnormal brain diffusivity in participants with persistent neuropsychiatric symptoms after COVID-19. *NeuroImmune Pharm Ther*. 2023-3-25 2023;2(1):37-48. doi:doi:10.1515/nipt-2022-0016

61. Lier J, Stoll K, Obrig H, et al. Neuropsychiatric phenotype of post COVID-19 syndrome in non-hospitalized patients. *Front Neurol*. 2022 2022;13:988359. doi:doi:10.3389/fneur.2022.988359

62. Liu TC, Yoo SM, Sim MS, Motwani Y, Viswanathan N, Wenger NS. Perceived Cognitive Deficits in Patients With Symptomatic SARS-CoV-2 and Their Association With Post-COVID-19 Condition. *JAMA Netw Open*. 2023-5-1 2023;6(5):e2311974. doi:doi:10.1001/jamanetworkopen.2023.11974

63. Loosen SH, Jensen BO, Tanislav C, Luedde T, Roderburg C, Kostev K. Obesity and lipid metabolism disorders determine the risk for development of long COVID syndrome: a cross-sectional study from 50,402 COVID-19 patients. *Infection*. 2022-10 2022;50(5):1165-1170. doi:doi:10.1007/s15010-022-01784-0

64. Luedke JC, Vargas G, Jashar DT, Morrow A, Malone LA, Ng R. Cognitive disengagement syndrome in pediatric patients with long covid: Associations with mood, anxiety, and functional impairment. *Child Neuropsychology*. 2023-9 2023:No Pagination Specified. doi:doi:<https://dx.doi.org/10.1080/09297049.2023.2252967>

65. Magel T, Meagher E, Boulter T, et al. Fatigue presentation, severity, and related outcomes in a prospective cohort following post-COVID-19 hospitalization in British Columbia, Canada. *Front Med (Lausanne)*. 2023 2023;10:1179783. doi:doi:10.3389/fmed.2023.1179783

66. Magnavita N, Arnesano G, Di Prinzio RR, et al. Post-COVID Symptoms in Occupational Cohorts: Effects on Health and Work Ability. *Int J Environ Res Public Health*. 2023-4-25 2023;20(9)doi:doi:10.3390/ijerph20095638

67. Margalit I, Yelin D, Sagi M, et al. Risk Factors and Multidimensional Assessment of Long Coronavirus Disease Fatigue: A Nested Case-Control Study. *Clin Infect Dis*. 2022-11-14 2022;75(10):1688-1697. doi:doi:10.1093/cid/ciac283

68. Marinkovic K, White DR, Alderson Myers A, Parker KS, Arienzo D, Mason GF. Cortical GABA Levels Are Reduced in Post-Acute COVID-19 Syndrome. *Brain Sci*. 2023-12-1 2023;13(12)doi:doi:10.3390/brainsci13121666

69. Martin EM, Rupprecht S, Schrenk S, et al. A hypoarousal model of neurological post-COVID syndrome: the relation between mental fatigue, the level of central nervous activation and cognitive processing speed. *J Neurol*. 2023-10 2023;270(10):4647-4660. doi:doi:10.1007/s00415-023-11819-7

70. Messin L, Puyraveau M, Benabdallah Y, et al. COVEVOL: Natural Evolution at 6 Months of COVID-19. *Viruses*. 2021-10-25 2021;13(11)doi:doi:10.3390/v13112151

71. Meza-Torres B, Delanerolle G, Okusi C, et al. Differences in Clinical Presentation With Long COVID After Community and Hospital Infection and Associations With All-Cause Mortality: English Sentinel Network Database Study. *JMIR Public Health Surveill*. 2022-8-16 2022;8(8):e37668. doi:doi:10.2196/37668

72. Mikuteit M, Baskal S, Klawitter S, et al. Amino acids, post-translational modifications, nitric oxide, and oxidative stress in serum and urine of long COVID and ex COVID human subjects. *Amino Acids*. 2023-9 2023;55(9):1173-1188. doi:doi:10.1007/s00726-023-03305-1

73. Mora AM, Kogut K, Sandhu NK, et al. SARS-CoV-2 infection and long COVID among California farmworkers. *J Rural Health*. 2023-9-16 2023;doi:doi:10.1111/jrh.12796

74. Navas-Otero A, Calvache-Mateo A, Martín-Núñez J, et al. Characteristics of Frailty in Perimenopausal Women with Long COVID-19. *Healthcare (Basel)*. 2023-5-18 2023;11(10)doi:doi:10.3390/healthcare11101468

75. Nishimi K, Tan J, Scoglio A, et al. Psychological Resilience to Trauma and Risk for COVID-19 Infection and Somatic Symptoms Over Two Years. *Psychosom Med*. 2023-5-9 2023;doi:doi:10.1097/psy.0000000000001215

76. O'Sullivan O, Holdsworth DA, Ladlow P, et al. Cardiopulmonary, Functional, Cognitive and Mental Health Outcomes Post-COVID-19, Across the Range of Severity of Acute Illness, in a Physically Active, Working-Age Population. *Sports Med Open*. 2023-2-2 2023;9(1):7. doi:doi:10.1186/s40798-023-00552-0

77. Pacho-Hernández JC, Fernández-de-Las-Peñas C, Fuensalida-Novo S, Jiménez-Antona C, Ortega-Santiago R, Cigarán-Mendez M. Sleep Quality Mediates the Effect of Sensitization-Associated Symptoms, Anxiety, and Depression on Quality of Life in Individuals with Post-COVID-19 Pain. *Brain Sci*. 2022-10-8 2022;12(10)doi:doi:10.3390/brainsci12101363

78. Pérez-López FR, Blümel JE, Vallejo MS, et al. Anxiety but not menopausal status influences the risk of long-COVID-19 syndrome in women living in Latin America. *Maturitas*. 2023-11-2 2023;180:107873. doi:doi:10.1016/j.maturitas.2023.107873

79. Peters C, Dulon M, Westermann C, Kozak A, Nienhaus A. Long-Term Effects of COVID-19 on Workers in Health and Social Services in Germany. *Int J Environ Res Public Health*. 2022-6-7 2022;19(12)doi:doi:10.3390/ijerph19126983

80. Phu DH, Maneerattanasak S, Shohaimi S, et al. Prevalence and factors associated with long COVID and mental health status among recovered COVID-19 patients in southern Thailand. *PLoS One*. 2023 2023;18(7):e0289382. doi:doi:10.1371/journal.pone.0289382

81. Pływaczewska-Jakubowska M, Chudzik M, Babicki M, Kapusta J, Jankowski P. Lifestyle, course of COVID-19, and risk of Long-COVID in non-hospitalized patients. *Front Med (Lausanne)*. 2022 2022;9:1036556. doi:doi:10.3389/fmed.2022.1036556

82. Rastogi R, Cerda IH, Ibrahim A, Chen JA, Stevens C, Liu CH. Long COVID and psychological distress in young adults: Potential protective effect of a prior mental health diagnosis. *Journal of Affective Disorders*. 2023-11 2023;340:639-648. doi:doi:<https://dx.doi.org/10.1016/j.jad.2023.08.031>

83. Rocha JQS, Caputo EL, Vieira YP, Afonso MDS, Duro SMS, de Oliveira Saes M. Physical activity status prevents symptoms of long covid: Sulcovid-19 survey. *BMC Sports Sci Med Rehabil*. 2023-12-14 2023;15(1):170. doi:doi:10.1186/s13102-023-00782-5

84. Roessler M, Tesch F, Batram M, et al. Post-COVID-19-associated morbidity in children, adolescents, and adults: A matched cohort study including more than 157,000 individuals with COVID-19 in Germany. *PLoS Med*. 2022-11 2022;19(11):e1004122. doi:doi:10.1371/journal.pmed.1004122

85. Román-Montes CM, Flores-Soto Y, Guaracha-Basañez GA, et al. Post-COVID-19 syndrome and quality of life impairment in severe COVID-19 Mexican patients. *Front Public Health*. 2023 2023;11:1155951. doi:doi:10.3389/fpubh.2023.1155951

86. Ruggeri M, Ricci M, Pagliaro M, Gerace C. Anosmia predicts memory impairment in post-covid-19 syndrome: Results of a neuropsychological cohort study. *European Archives of Psychiatry and Clinical Neuroscience*. 2023-8 2023:No Pagination Specified. doi:doi:<https://dx.doi.org/10.1007/s00406-023-01670-2>

87. Salci MA, Carreira L, Baccon WC, et al. Perceived quality of life and associated factors in long COVID syndrome among older Brazilians: A cross-sectional study. *J Clin Nurs*. 2024;33(1):178-191. doi:10.1111/jocn.16618

88. Salve HR, Daniel RA, Kumar A, Kumar R, Misra P. Prevalence and Determinants of Long COVID Among Patients Attending the Outpatient Department of a Subdistrict Hospital in Haryana. *Cureus*. 2023-9 2023;15(9):e46007. doi:doi:10.7759/cureus.46007

89. Samper-Pardo M, León-Herrera S, Oliván-Blázquez B, Gascón-Santos S, Sánchez-Recio R. Clinical characterization and factors associated with quality of life in Long COVID patients: Secondary data analysis from a randomized clinical trial. *PLoS One*. 2023 2023;18(5):e0278728. doi:doi:10.1371/journal.pone.0278728

90. Satar S, Şahin ME, Ergün P. Health related quality of life and its determinants in COVID-19 patients. *Tuberk Toraks*. 2023-9 2023;71(3):250-260. doi:doi:10.5578/tt.20239706

91. Scholz U, Bierbauer W, Lüscher J. Social Stigma, Mental Health, Stress, and Health-Related Quality of Life in People with Long COVID. *Int J Environ Res Public Health*. 2023-2-22 2023;20(5)doi:doi:10.3390/ijerph20053927

92. Selvakumar J, Havdal LB, Drevvatne M, et al. Prevalence and Characteristics Associated With Post-COVID-19 Condition Among Nonhospitalized Adolescents and Young Adults. *JAMA Netw Open*. 2023-3-1 2023;6(3):e235763. doi:doi:10.1001/jamanetworkopen.2023.5763

93. Shachar-Lavie I, Shorer M, Segal H, Fennig S, Ashkenazi-Hoffnung L. Mental health among children with long COVID during the COVID-19 pandemic. *Eur J Pediatr*. 2023-4 2023;182(4):1793-1801. doi:doi:10.1007/s00431-023-04854-z

94. Song Z, Giuriato M. Demographic And Clinical Factors Associated With Long COVID. *Health Aff (Millwood)*. 2023-3 2023;42(3):433-442. doi:doi:10.1377/hlthaff.2022.00991

95. Staples LG, Nielssen O, Dear BF, et al. Prevalence and Predictors of Long COVID in Patients Accessing a National Digital Mental Health Service. *Int J Environ Res Public Health*. 2023-9-13 2023;20(18)doi:doi:10.3390/ijerph20186756

96. Subramanian A, Nirantharakumar K, Hughes S, et al. Symptoms and risk factors for long COVID in non-hospitalized adults. *Nat Med*. 2022-8 2022;28(8):1706-1714. doi:doi:10.1038/s41591-022-01909-w

97. Tavares-Júnior JWL, Oliveira DN, da Silva JBS, et al. Long-covid cognitive impairment: Cognitive assessment and apolipoprotein E (APOE) genotyping correlation in a Brazilian cohort. *Front Psychiatry*. 2022 2022;13:947583. doi:doi:10.3389/fpsyt.2022.947583

98. Tebeka S, Carcaillon-Bentata L, Decio V, et al. Complex association between post-COVID-19 condition and anxiety and depression symptoms. *Eur Psychiatry*. 2023-12-13 2023:1-36. doi:doi:10.1192/j.eurpsy.2023.2473

99. Terai H, Ishii M, Takemura R, et al. Comprehensive analysis of long COVID in a Japanese nationwide prospective cohort study. *Respir Investig*. 2023-11 2023;61(6):802-814. doi:doi:10.1016/j.resinv.2023.08.008

100. Thronicke A, Hinse M, Weinert S, Jakubowski A, Grieb G, Matthes H. Factors Associated with Self-Reported Post/Long-COVID-A Real-World Data Study. *Int J Environ Res Public Health*. 2022-12-2 2022;19(23)doi:doi:10.3390/ijerph192316124

101. Tudor L, Harenwall S, Henderson R, Bland AR. Post-covid-19 syndrome: Self-compassion and psychological flexibility moderate the relationship between physical symptom load and psychosocial impact. *Acta Psychol (Amst)*. 2023-11 2023;241:104093. doi:doi:10.1016/j.actpsy.2023.104093

102. Uniyal N, Sethi Y, Sharma PC, et al. Post-COVID Syndrome and Severity of COVID-19: A Cross-Sectional Epidemiological Evaluation From North India. *Cureus*. 2022-7 2022;14(7):e27345. doi:doi:10.7759/cureus.27345

103. Walker S, Goodfellow H, Pookarnjanamorakot P, et al. Impact of fatigue as the primary determinant of functional limitations among patients with post-COVID-19 syndrome: a cross-sectional observational study. *BMJ Open*. 2023-6-7 2023;13(6):e069217. doi:doi:10.1136/bmjopen-2022-069217

104. Wang S, Quan L, Chavarro JE, et al. Associations of depression, anxiety, worry, perceived stress, and loneliness prior to infection with risk of post-COVID-19 conditions. *JAMA Psychiatry*. 2022-11 2022;79(11):1081-1091. doi:doi:<https://dx.doi.org/10.1001/jamapsychiatry.2022.2640>

105. Whiteside DM, Basso MR, Naini SM, et al. Outcomes in post-acute sequelae of COVID-19 (PASC) at 6 months post-infection Part 1: Cognitive functioning. *The Clinical Neuropsychologist*. 2022-5 2022;36(4):806-828. doi:doi:<https://dx.doi.org/10.1080/13854046.2022.2030412>

106. Wright TJ, Pyles RB, Sheffield-Moore M, et al. Low growth hormone secretion associated with post-acute sequelae SARS-CoV-2 infection (PASC) neurologic symptoms: A case-control pilot study. *Mol Cell Endocrinol*. 2024-1-1 2024;579:112071. doi:doi:10.1016/j.mce.2023.112071

107. Yaksi N, Teker AG, Imre A. Long COVID in Hospitalized COVID-19 Patients: A Retrospective Cohort Study. *Iran J Public Health*. 2022-1 2022;51(1):88-95. doi:doi:10.18502/ijph.v51i1.8297

108. Zhang D, Chung VC, Chan DC, et al. Determinants of post-COVID-19 symptoms among adults aged 55 or above with chronic conditions in primary care: data from a prospective cohort in Hong Kong. *Front Public Health*. 2023 2023;11:1138147. doi:doi:10.3389/fpubh.2023.1138147

109. Zhang H, Huang C, Gu X, et al. 3-year outcomes of discharged survivors of COVID-19 following the SARS-CoV-2 omicron (B.1.1.529) wave in 2022 in China: a longitudinal cohort study. *Lancet Respir Med*. 2023-11-21 2023;doi:doi:10.1016/s2213-2600(23)00387-9

110. Zheng B, Vivaldi G, Daines L, et al. Determinants of recovery from post-COVID-19 dyspnoea: analysis of UK prospective cohorts of hospitalised COVID-19 patients and community-based controls. *Lancet Reg Health Eur*. 2023-6 2023;29:100635. doi:doi:10.1016/j.lanepe.2023.100635

111. Zheng C, Huang WY, Sun FH, et al. Association of Sedentary Lifestyle with Risk of Acute and Post-Acute COVID-19 Sequelae: A Retrospective Cohort Study. *Am J Med*. 2023-12-16 2023;doi:doi:10.1016/j.amjmed.2023.12.002

112. Mazza MG, Palladini M, Villa G, De Lorenzo R, Rovere Querini P, Benedetti F. Prevalence, trajectory over time, and risk factor of post-COVID-19 fatigue. *J Psychiatr Res*. Nov 2022;155:112-119. doi:10.1016/j.jpsychires.2022.08.008

113. Milde C, Glombiewski JA, Wilhelm M, Schemer L. Psychological Factors Predict Higher Odds and Impairment of Post-COVID Symptoms: A Prospective Study. *Psychosom Med*. Jul-Aug 01 2023;85(6):479-487. doi:10.1097/PSY.0000000000001214
